# Exploiting Configurational Lability in Aza‐Sulfur Compounds for the Organocatalytic Enantioselective Synthesis of Sulfonimidamides

**DOI:** 10.1002/anie.202109160

**Published:** 2021-11-02

**Authors:** Michael J. Tilby, Damien F. Dewez, Adrian Hall, Carolina Martínez Lamenca, Michael C. Willis

**Affiliations:** ^1^ Department of Chemistry University of Oxford Chemistry Research Laboratory Mansfield Road Oxford OX1 3TA UK; ^2^ UCB Biopharma 1420 Braine-l'Alleud Belgium; ^3^ Medicinal Chemistry Janssen Research & Development 2340 Beerse Belgium

**Keywords:** alkylation, cinchona alkaloid, enantioselective catalysis, phase-transfer catalysis, sulfonimidamides

## Abstract

Methods for establishing the absolute configuration of sulfur‐stereogenic aza‐sulfur derivatives are scarce, often relying on cumbersome protocols and a limited pool of enantioenriched starting materials. We have addressed this by exploiting, for the first time, a feature of sulfonimidamides in which it is possible for tautomeric structures to also be enantiomeric. Such sulfonimidamides can readily generate prochiral ions, which we have exploited in an enantioselective alkylation process. Selectivity is achieved using a readily prepared bis‐quaternized phase‐transfer catalyst. The overall process establishes the capability of configurationally labile aza‐sulfur species to be used in asymmetric catalysis.

## Graphical abstract

The unusual stereochemical feature of certain sulfonimidamides, in which tautomeric forms are also enantiomeric, has been exploited to develop a catalytic enantioselective synthesis of alkylated sulfonimidamides. The reactions proceed through a prochiral anionic intermediate to deliver enantiomerically enriched products. A double‐alkylated, cinchona alkaloid‐derived catalyst is used as a phase‐transfer catalyst.

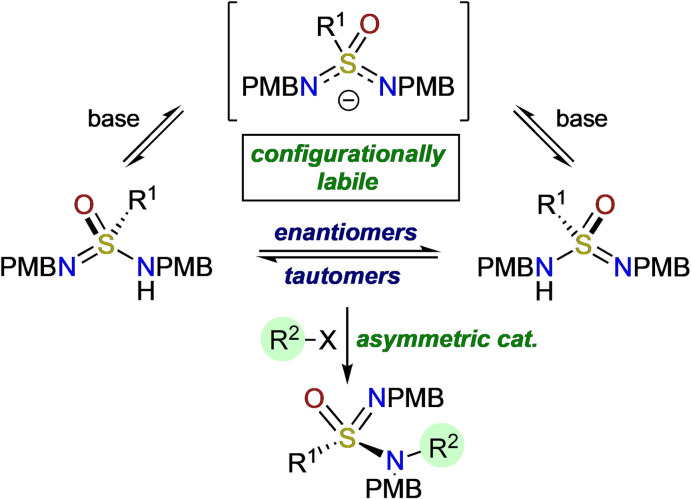

Several aza‐sulfur containing functional groups have established utility in a range of applications (Scheme 1 a). Sulfinamides (**A**) are the most used aza‐sulfur(IV) species,^[1]^ and Ellman's sulfinamide chiral auxiliary provides a compelling example of how the stereocentre at sulfur can be effectively used in synthesis.^[2]^ Aza‐sulfur(VI) compounds have also gained significant attention, demonstrated by sulfonamides (**B**) and sulfoximines (**C**) being exploited as pharmacophores^[3]^ in many commercial pharmaceuticals^[4]^ and agrochemicals.^[5]^ The success of these molecules is often ascribed to properties such as metabolic stability, polarity, and hydrogen bonding ability.^[3b]^

**Scheme 1:**
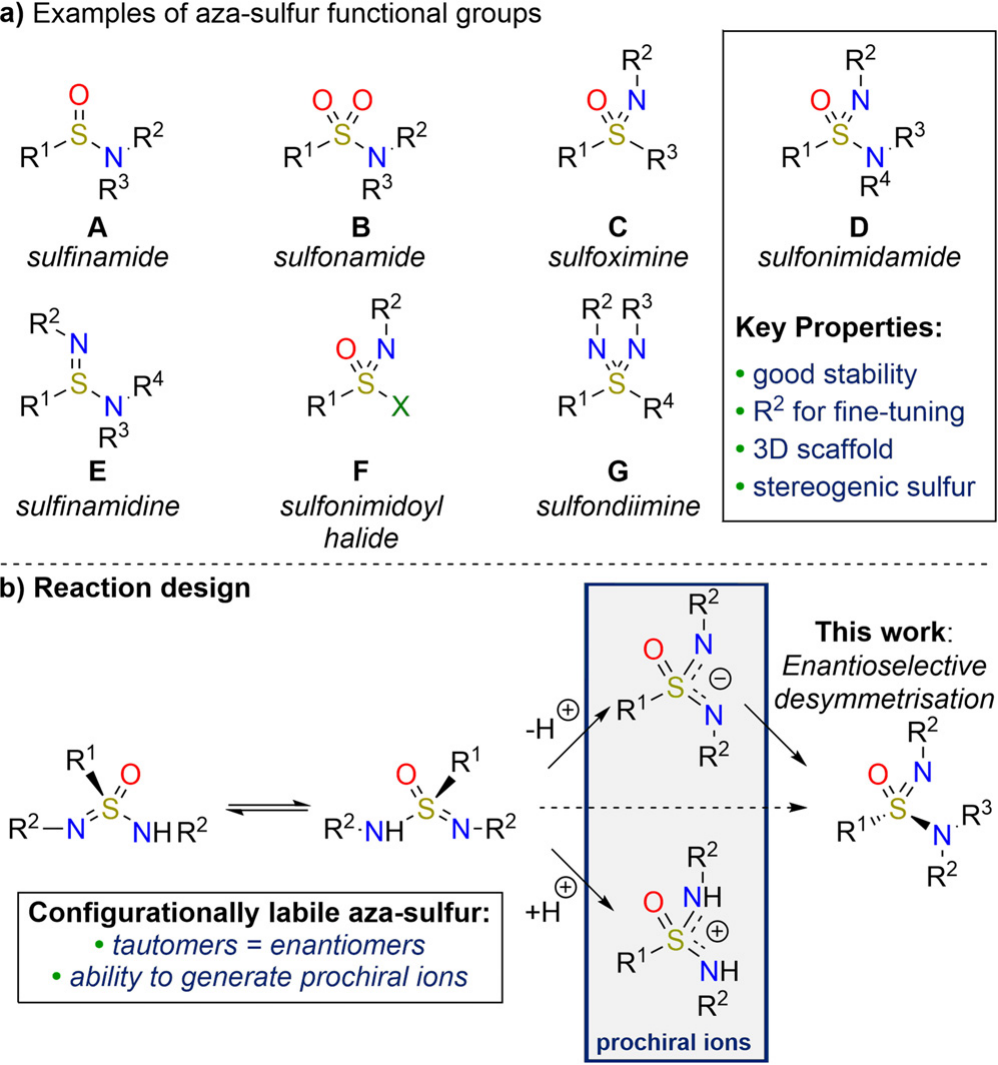
a) Examples of aza‐sulfur compounds, including sulfonimidamides. b) Proposed enantioselective synthesis of aza‐sulfur compounds by exploiting configurational lability.

The aza‐analogues of sulfonamides, sulfonimidamides (**D**), although first reported in the 1930s,^[6]^ have been considerably less explored, but contain an array of advantageous features.^[7]^ For example, sulfonimidamides form a three‐dimensional scaffold with a central stereogenic sulfur atom, and the presence of the imidic nitrogen provides the potential for an extra hydrogen bond‐donor site. Alternatively, an additional vector can be accessed through incorporation of substituents on this nitrogen atom, allowing the fine‐tuning of physiochemical properties. These attributes have led to sulfonimidamides gaining traction in biological settings,^[8]^ and as catalysts.^[9]^ Consequently, there have been considerable efforts to develop new synthetic methods towards this functional group,^[10]^ as well as routes to other underexplored aza‐sulfur derivatives,[^10h^, ^11^] including sulfinamidines (**E**),^[12]^ sulfonimidoyl halides (**F**)[^10c^, ^13^] and sulfondiimines (**G**).^[14]^

There have been a number of approaches to achieve enantioselective syntheses of aza‐sulfur functional groups.^[15]^ For sulfinamides, asymmetric syntheses have traditionally relied on a chiral auxiliary approach,[^2^, ^16^] and only in recent years have catalytic methods emerged.^[17]^ The ability to prepare a limited pool of enantioenriched sulfinamides has been capitalised upon, with these undergoing stereoselective conversion to several other aza‐sulfur motifs, notably sulfonimidoyl halides,[^9b^, ^13^, ^18a^, ^18b^] and recently sulfoximines.^[19]^

Recent breakthroughs have exploited prochiral sulfoximines (**C**) in enantioselective desymmetrisation reactions,^[20]^ although the extension of these concepts to other aza‐sulfur functional groups is yet to be developed. An intriguing stereochemical feature of sulfinamidines (**E**) and sulfonimidamides (**D**) is based on their ability to exist in two tautomeric forms; for variants with identical *N*‐substituents, rapid equilibration between tautomers would lead to configurational lability at sulfur (shown for sulfonimidamides in Scheme 1 b). Deprotonation of such a sulfonimidamide would lead to a prochiral anion intermediate, which, when followed by a desymmetrising transformation would lead to enantioenriched products via a unique pathway. Protonation of the starting sulfonimidamide would also generate a prochiral intermediate, this time being cationic.

As there has been minimal investigation into aza‐sulfur systems that demonstrate tautomerisation,^[21]^ we first chose to probe this feature in more detail. *N*,*N*′‐Sulfonimidamides **2** were selected as candidates for this investigation, in part due to recent advances in their ease of synthesis[^10b^, ^10i^] and also their good stability profile. We began by examining the chirality of compound **2 a** in the solid state with X‐ray crystallographic data (Scheme 2 a). The crystal structure shows **2 a** with two distinct sulfur‐nitrogen bond lengths of 1.631(2) and 1.508(2) Å, indicating the presence of distinguishable amidic (S−N) and imidic (S=N) bonds, respectively. The crystal was also shown to exist as a racemate of two enantiomeric units, with a single asymmetric unit containing the *para*‐methoxybenzyl (PMB) groups in chemically distinguishable environments.

**Scheme 2:**
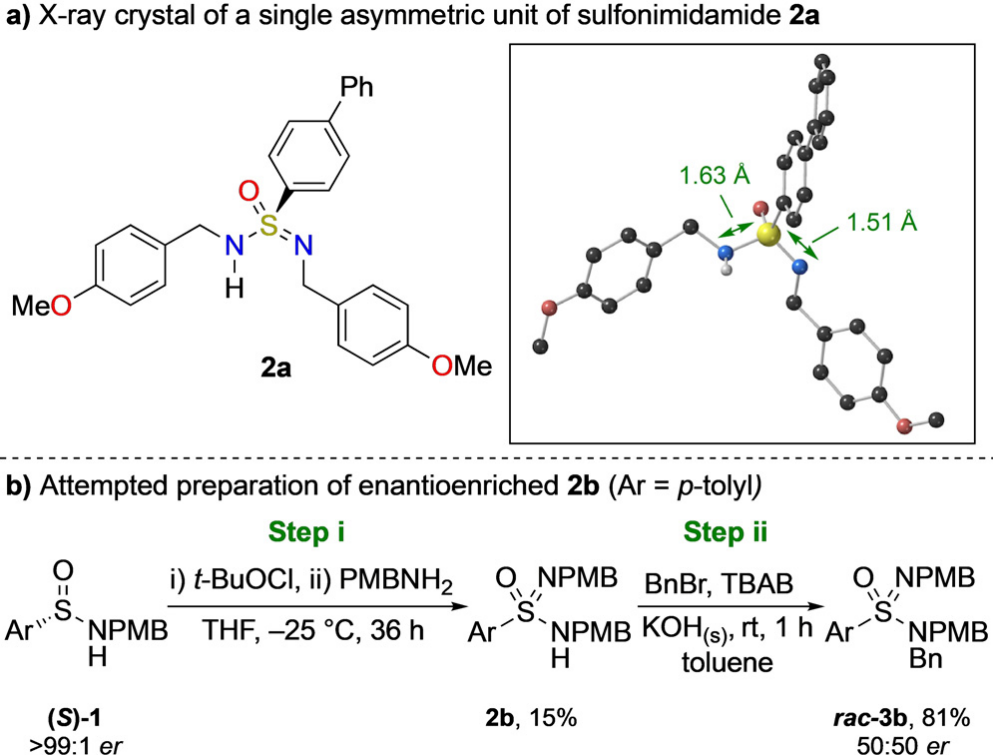
a) X‐ray crystallographic data for sulfonimidamide **2 a**. The majority of H‐atoms have been removed for clarity. b) Attempt to synthesise and react enantiopure sulfonimidamide **2 b**. Step i: chlorination and amine displacement, step ii: alkylation with benzyl bromide.

As the X‐ray structure of **2 a** displays the presence of chirality, we attempted to prepare compound **2 b** via a series of known stereoselective transformations, from a derivative of one of the few commercially available enantiopure sulfinamides (**(*S*)‐1**, Scheme 2 b, see Supporting information).[^9b^, ^18a^, ^18b^] From these experiments, the resulting **2 b** showed no significant optical rotation. Furthermore, compounds of this type could not be separated by chiral‐phase HPLC, as would be expected for a configurationally labile motif. In order to consolidate this evidence, **2 b** was alkylated in the presence of base, which resulted in ***rac*** **‐3 b**. This indicates that at some stage in this sequence, racemisation must be occurring. As we were able to show that step i is stereoselective with an alternative amine (see Supporting Information), this racemisation is likely due to rapid tautomerisation of **2 b**, or from formation of a symmetric anion from addition of base in step ii. Regardless, this indicates that the stereochemical information in this process is readily lost, priming this class of aza‐sulfur motif for asymmetric catalysis.

To gain an understanding of the dynamics of tautomerisation and racemisation of *N*,*N*′‐sulfonimidamides (**2**), a series of ^1^H and ^13^C NMR experiments were conducted (Scheme 3, and Supporting Information). Examination of the ^1^H NMR spectrum of **2 b** in several solvents including the non‐polar aprotic solvent [D_8_]toluene, at room temperature (Scheme 3 a), indicates that the two PMB groups appear to be chemically and magnetically equivalent. This observed chemical equivalence is evident from a single environment of the H^3^, H^4^ and H^5^ protons, as well as the diastereotopic protons H^2^ and H^2^′ appearing as two discrete doublets (*J*=14.0 Hz). That this ^1^H spectrum contains no observable N*H* proton (H^1^), in conjunction with the diastereotopic protons only showing coupling to each other, implies that the proposed tautomerisation is rapid on the NMR‐timescale in toluene. In turn, this suggests that epimerisation at sulfur would also be occurring at a fast rate.

**Scheme 3:**
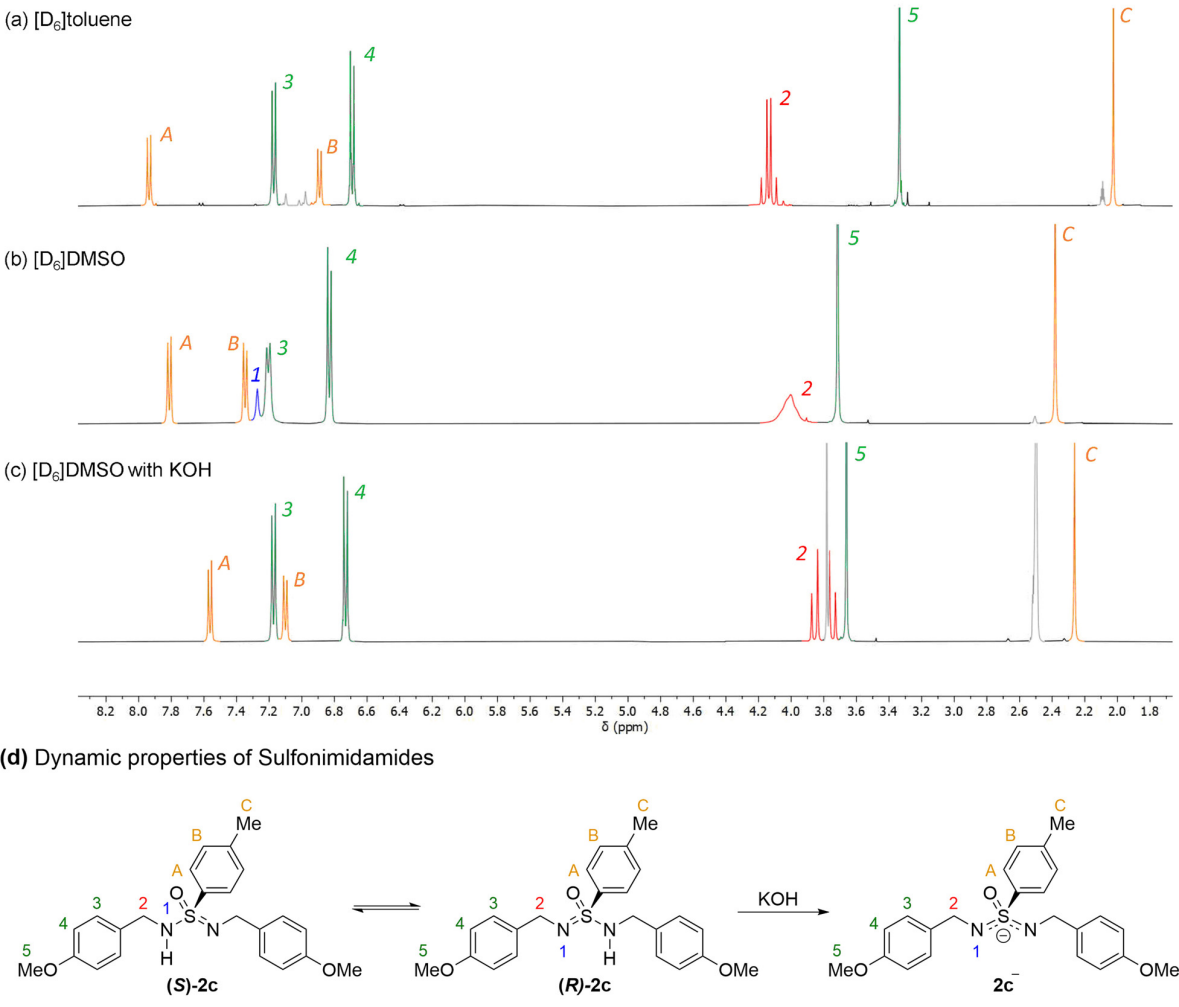
^1^H NMR spectra of compound **2 b** at 400 MHz and 295 K, analysing the tautomerization process a) [D_8_]toluene as solvent. b) [D_6_]DMSO as solvent. c) [D_6_]DMSO as solvent in the presence of excess potassium hydroxide. d) Representation of tautomerisation with racemisation of sulfonimidamide **2 b** followed by deprotonation with potassium hydroxide to the anion.

The ^1^H NMR spectra of **2 b** in toluene conducted at cryogenic temperatures (see Supporting Information), indicate that, although slowed, tautomerization persists even at temperatures as low as −40 °C. In order to decelerate the rate of epimerisation sufficiently at room temperature, we employed [D_6_]DMSO as solvent, as this has previously been used to examine prototropic tautomerism,^[22]^ owing to its polar aprotic nature. Analysis of the [D_6_]DMSO ^1^H spectrum (Scheme 3 b), shows that the N*H* proton is observable as H^1^, a broad singlet at 7.27 ppm. In addition, [D_6_]DMSO appears to cause significant broadening for certain environments of **2 b**, in particular those close to the nitrogen atoms such as H^3^ and H^2^, which would arise from multiple peaks coalescing in a slow chemical exchange process. From these experiments, it is conceivable that *N*,*N*′‐sulfonimidamide **2 b** can be deprotonated to form an achiral anion in solution. In order to test the viability of this hypothesis, excess potassium hydroxide was added to **2 b** in [D_6_]DMSO (Scheme 3 c). The most noticeable change between this spectrum and the previous one is that no broad peaks are present. Specifically, the H^2^ environment now consists of two well resolved doublets, indicative of diastereotopic protons which only couple to each other (*J*=14.5 Hz), hence, once again causing the two PMB groups to behave as chemically and magnetically equivalent. This observation can either arise from potassium hydroxide considerably accelerating the rate of tautomerization in [D_6_]DMSO, or from the generation of the symmetric anion **2 b^−^** (Scheme 3 d). The ^1^H NMR spectrum suggests considerable formation of **2 b^−^**, as several resonances are significantly shifted upfield. This is most prominent for protons positioned close to the nitrogen atoms, such as H^A^ and H^2^.

The investigations described above, concerning anion formation, suggested that *protonation* of the sulfonimidamide could also lead to a symmetric system, this time a cation. Pleasingly, we found that addition of TFA to neutral sulfonimidamide **2 b** in [D_6_]DMSO supports this idea (see Supporting Information). To further verify that the ions are in fact inherently symmetric, X‐ray crystallography of the protonated sulfonimidamide **4** was obtained (Scheme 4). When examining the sulfur‐nitrogen bond lengths in **4** it can be seen they are effectively identical, with a value of 1.58 Å. Intriguingly, this is also near the averaged value between the previously discussed amidic (S−N) and imidic (S=N) bonds, indicating a partial double bond. Therefore, when free rotation about the S−N bonds is possible, the cationic species **4** contains a plane of symmetry through the sulfur atom. Overall, these experiments indicate that aza‐sulfur motifs with the potential for tautomerisation can exhibit rapid configurational lability at the stereogenic centre in solution. In addition, protonation or deprotonation allows ready access to prochiral anions and cations.

**Scheme 4:**
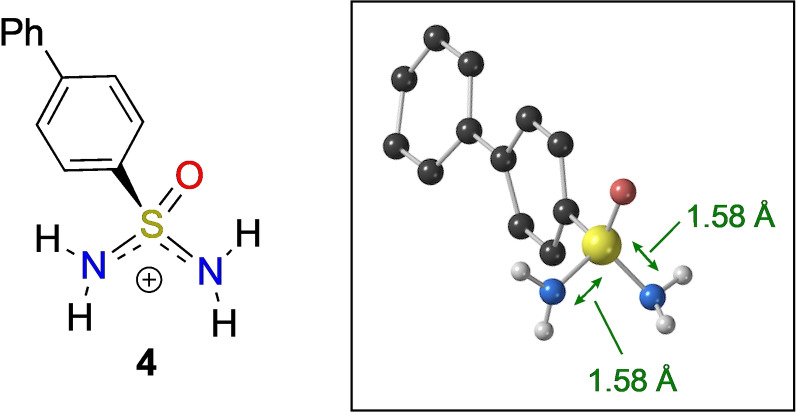
a) X‐ray crystallographic data for sulfonimidamide **4**. The majority of H‐atoms have been removed for clarity, as have the chloride counterion and water molecule in the structure.

Having established the configuration lability in sulfonimidamides such as **2**, these molecules were now primed for the development of catalytic dynamic kinetic resolution processes, or desymmetrisation of their corresponding ions. Accordingly, we targeted their use in a catalytic desymmetrisation route to enantiomerically enriched sulfonimidamides. The majority of existing methods for the synthesis of enantioenriched sulfonimidamides rely on the addition of an enantiopure amine into a racemic sulfonimidoyl halide (**F**), which commonly results in a poor diastereomeric ratio of products.[^8b^, ^10c^, ^24a^] Alternative routes often proceed through the formation of a handful of enantioenriched sulfonimidoyl halides, followed by a stereoselective displacement, typically with inversion of stereochemistry.[^13^, ^18a^, ^24b^] In these sequences the use of sulfonimidoyl chlorides is often undesirable on account of their chemical instability.^[25]^ Whilst Boc‐protected sulfonimidoyl fluorides have recently been shown to be a more stable substitute,^[13]^ both species are prone to significant racemisation if not handled with care, and hence loss of stereochemical information can result.[^18a^, ^26^] Limited reports have also used more specific, stable enantioenriched precursors.[^10g^, ^27^] Although the *N*‐based reactivity of sulfonimidamides is well documented,^[28]^ there are only a handful of methods that exploit this in asymmetric synthesis. Examples to date use a limited selection of simple sulfonimidamide starting materials and rely on the formation of diastereomeric products, demonstrating that there is no general method for the direct control of configuration at the sulfur centre.^[29]^ We reiterate, at present there are no reported catalytic enantioselective syntheses of sulfonimidamides, i.e., that set the configuration of the sulfur stereocentre.

We selected phase‐transfer catalysed enantioselective alkylation as our initial reaction to study. Although related alkylation reactions using catalysts that incorporate a chiral counterion have been extended from C−C bond formation to include C−X bond formation,[^30^, ^31^] the only examples involving C−S bond formation are used in sulfoxide syntheses.^[32]^ To the best of our knowledge there is no known asymmetric reaction to directly control a S(VI) stereocentre using a phase‐transfer catalyst.

Initially, sulfonimidamide **2 a** was reacted with one equivalent of benzyl bromide in the presence of base and a phase‐transfer catalyst, using toluene as solvent (Scheme 5 a). Notably, in our control reactions, we were pleased to observe that **2 a** showed no background reaction in the absence of base or catalyst. To evaluate the possibility of a chiral catalyst inducing enantioenrichment, a variety of cinchona alkaloid derived phase‐transfer catalysts were explored (Scheme 5 b). Pleasingly, many of these catalysts showed good reactivity, and provided proof‐of‐concept with varying levels of enantioinduction, with the optimal catalyst identified as **CAT‐1** (see Supporting Information for details). To show that this desymmetrisation strategy is suitable in producing both high yields and enantiomeric ratios, we lowered the temperature of the reaction to −20 °C and, as expected, the selectivity did improve, but the conversion was drastically depleted even after 24 h (Scheme 5 c, entry ii).

**Scheme 5:**
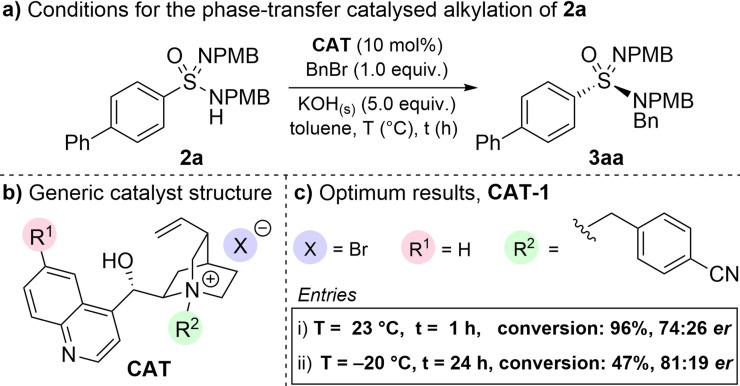
Initial evaluation of cinchona alkaloid derived phase transfer catalysts. **2 a** (1.0 equiv) at a concentration of 0.1 M in toluene and conversions determined by analysis of crude ^1^H NMR spectra.

As the singly alkylated cinchona alkaloid scaffold did not deliver an effective catalyst, our attention turned to *bis*‐quaternized phase‐transfer catalysts,^[33]^ as reports indicated that they could deliver increased activity.^[33b]^ Chiral catalysts of this nature were developed by Merck, and have effectively been used for a C−C bond forming spirocyclisation reaction. The modular nature of these catalysts allows multiple substituents to be placed on the alkaloid core, providing an opportunity for further fine‐tuning of the catalyst structure. We began our investigation of these new catalysts with **CAT‐2** (Table 1); however, when using our earlier conditions, we were surprised to see a significant reduction in both conversion and enantioselectivity (entry 1). In contrast to the mono‐substituted catalysts, we noted that the reactivity and selectivity had a high dependence on the catalyst loading and equivalents of base used (entry 2, see Supporting Information for further details). This observation arises from the efficiency of *bis*‐quaternized catalysts being dependent on the rate of catalyst degradation in comparison to the rate of product formation, which will likely be influenced by catalyst solubility and degree of mass‐transfer.^[33d]^ We then took advantage of the additional variation possible with the second catalyst substituent, which is attached to the quinoline nitrogen. Due in part to the success of **CAT‐2** and the *N‐para*‐nitrile benzyl substituent in the original catalyst evaluation, we decided to predominantly use this backbone for further catalyst design (see Supporting Information). Incorporation of a nitrile substituent (**CAT‐3** and **CAT‐4**, entries 3,4) or an extended aromatic system (**CAT‐5**, entry 5) retained reactivity, but resulted in reduced stereoselectivity and the catalysts performed similarly to the parent catalyst **CAT‐1**. Fluorinated aromatic substituents were identified as beneficial for enantioselectivity (entries 6,7). With **CAT‐7** showing good operation and robustness at room temperature, the reaction was then conducted at −20 °C, and gratifyingly, excellent conversion was observed paired with high enantioselectivity (entry 8).

**Table 1:** Further optimisation of the phase‐transfer catalysed alkylation reaction with *bis*‐quaternized phase‐transfer catalyst.^[a]^ 
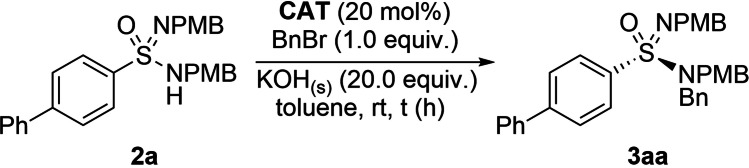

entry	catalyst	*t* [h]	conversion [%]	*er*
1^[b]^	**CAT‐2**	7	61	68:32
2	**CAT‐2**	1	100	81:19
3	**CAT‐3**	1	100	75.5:24.5
4	**CAT‐4**	1	100	76.5:23.5
5	**CAT‐5**	1	100	73.5:26.5
6	**CAT‐6**	1	100	81.5:18.5
7	**CAT‐7**	1	100	85.5:14.5
8^[c]^	**CAT‐7**	24	84	90.5:9.5
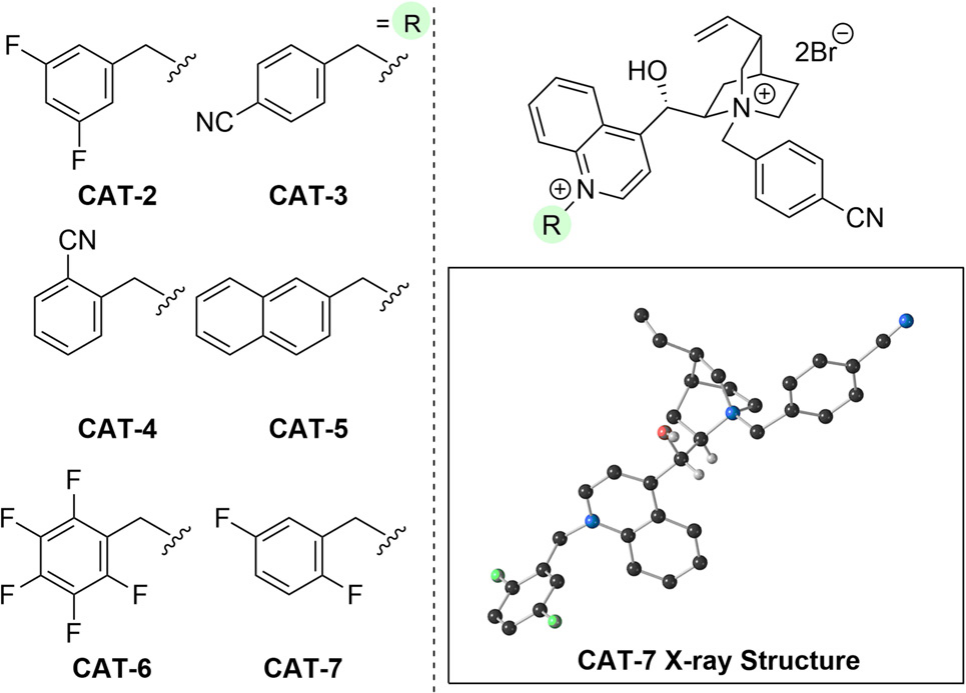				

[a] Sulfonimidamide (1.0 equiv) at a concentration of 0.1 M in toluene and conversions determined by analysis of crude ^1^H NMR spectrum. [b] 10 mol % of catalyst and 5.0 equiv of KOH_(s)_. [c] −20 °C in toluene (0.05 M). In the X‐ray structure, the Br anions, and the majority of H‐atoms have been removed for clarity.^[23]^

We next explored the generality of the developed system using a variety of electrophiles (Scheme 6). A range of simple benzylic systems with varying electronic properties were examined, with all producing the desired products in good to excellent yields. The *para*‐fluorine substituent (**3 ab**) provided an enantiomeric ratio similar to the model system. Changing the *para* substituent to a sulfide resulted in a mixed valence sulfur derivative being produced (**3 ac**). Electron withdrawing substituents were also tolerated as evident with the strongly withdrawing cyano group of species **3 ad**. Of note, example **3 ae** contains a reactive aldehyde functionality and provides a good enantiomeric ratio. A naphthyl group was effectively introduced (**3 af**). We next turned our attention to non‐benzylic electrophiles, and although allyl bromide has drastically different topology it still provided an excellent result (**3 ag**). Increasing the steric bulk on the alkene resulted in a reduction in the rate of reaction (**3 ah**) while maintaining good selectivity. Finally, we were pleased to note that although substrate **3 ai** was produced in a low yield owing to poor reactivity, a substantial enantiomeric ratio was obtained, showing the possibility of the extension of a desymmetrisation reaction to alkyl electrophiles.

**Scheme 6:**
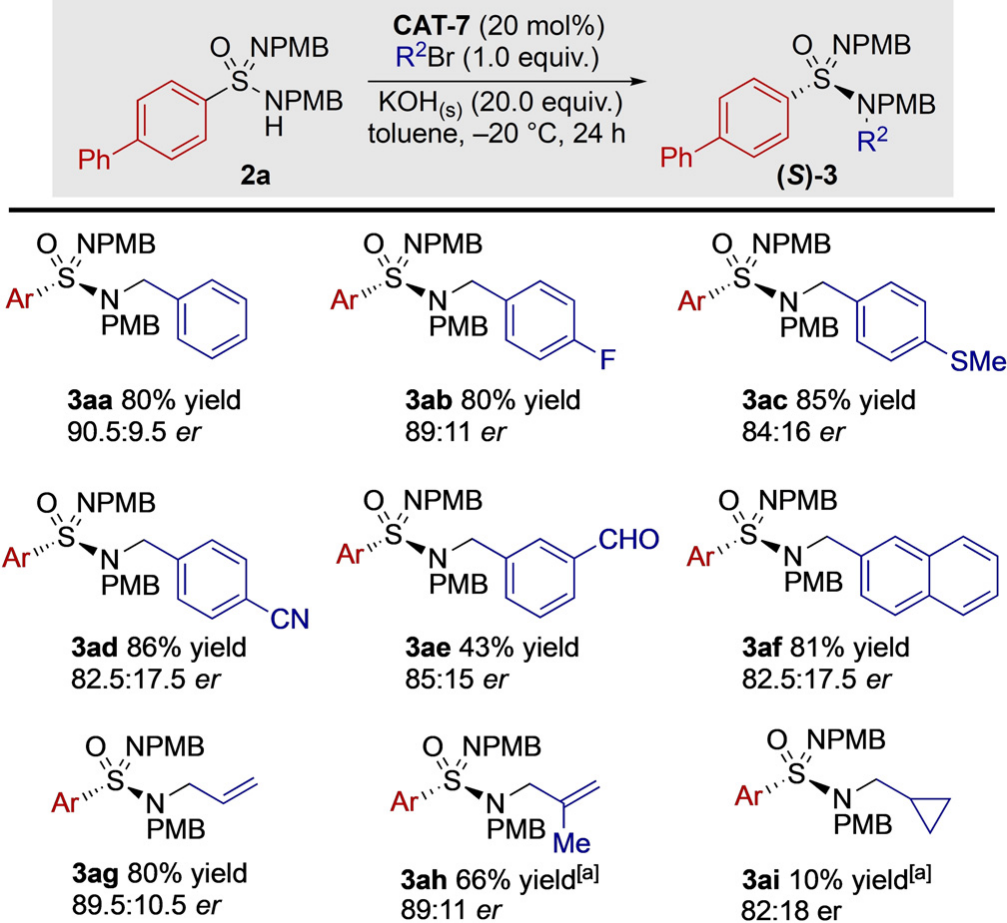
Exploration of the generality of the organocatalysed alkylation by examining the scope of the electrophile. Reaction conducted with sulfonimidamide **2 a** (1.0 equiv) at a concentration of 0.05 M in toluene with stirring at 1000 rpm. [a] Reaction time 48 h. All *er* measurements taken on the bulk sample. Isolated yields.

We then explored variation of the sulfonamidamide substrates (Scheme 7). Pleasingly, tolyl‐derived **3 b** performed similarly to our model system. Furthermore, we were able to synthesise an authentic sample of **(*R*)‐3 b** from sulfinamide **(*S*)‐1** via a series of known transformations involving sulfonimidoyl chlorides (see Supporting Information).^[18a]^ From these experiments we were able to determine the absolute configuration of sulfonimidamide **3 b** produced by our asymmetric catalysis, and hence, by analogy, the sulfonimidamides reported here are assigned as the *S*‐enantiomers. Several other sulfonimidamides with simple aromatic backbones were explored; the influence of the *para*‐phenyl substituent in **2 a** in comparison to the unsubstituted phenyl ring (**3 c**), should be noted, with **3 c** being produced in similar yield, but lower selectivity. A methyl group substitution at the *meta*‐position (**3 d**) gave similar results to substrate **3 b**; however, a methyl group in the *ortho*‐position (**3 e**) resulted in a reduction in both selectivity and reactivity, with the reaction requiring 48 hours to obtain a moderate yield. We attributed this poor reactivity to the low solubility of substrate **2 e** in toluene. Pleasingly, a *para*‐fluoro substituent (**3 f**) gave a high enantiomeric ratio and excellent yield. Thiophene heterocycles are commonplace in medicinal and materials chemistry,^[34]^ hence we were pleased that derivative **3 g** gave a near identical result to our model system **3 a**. To further exemplify the potential application to medicinal chemistry, the pyrazole containing **3 h**, which is a chiral at sulfur analogue of the commercial sulfonamide drug Celecoxib, was evaluated. To date, enantioenriched sulfonimidamides have primarily been reported with aromatic backbones, where the few alternatives present are predominantly in the patent literature,[^8a^, ^8d^, ^8e^, ^8f^, ^35^] and rely on diastereomer formation to allow separation by preparative HPLC. Accordingly, we were interested in the synthesis of enantioenriched sulfonimidamides with non‐aromatic backbones; alkenyl sulfonimidamide **3 i** was obtained with no alkene isomerisation being observed. Finally, methyl‐sulfonimidamide **3 j** was obtained in moderate yield and selectivity. The substrate specificity exhibited with catalyst **CAT‐7** suggests that to expand this methodology to substrates with sp^3^‐hybridised backbones it would be necessary to further optimize the phase‐transfer catalyst, or evaluate alternative catalyst designs. Overall, this methodology has the potential to significantly diversify the variety of enantioenriched sulfonimidamides which are currently available.

**Scheme 7:**
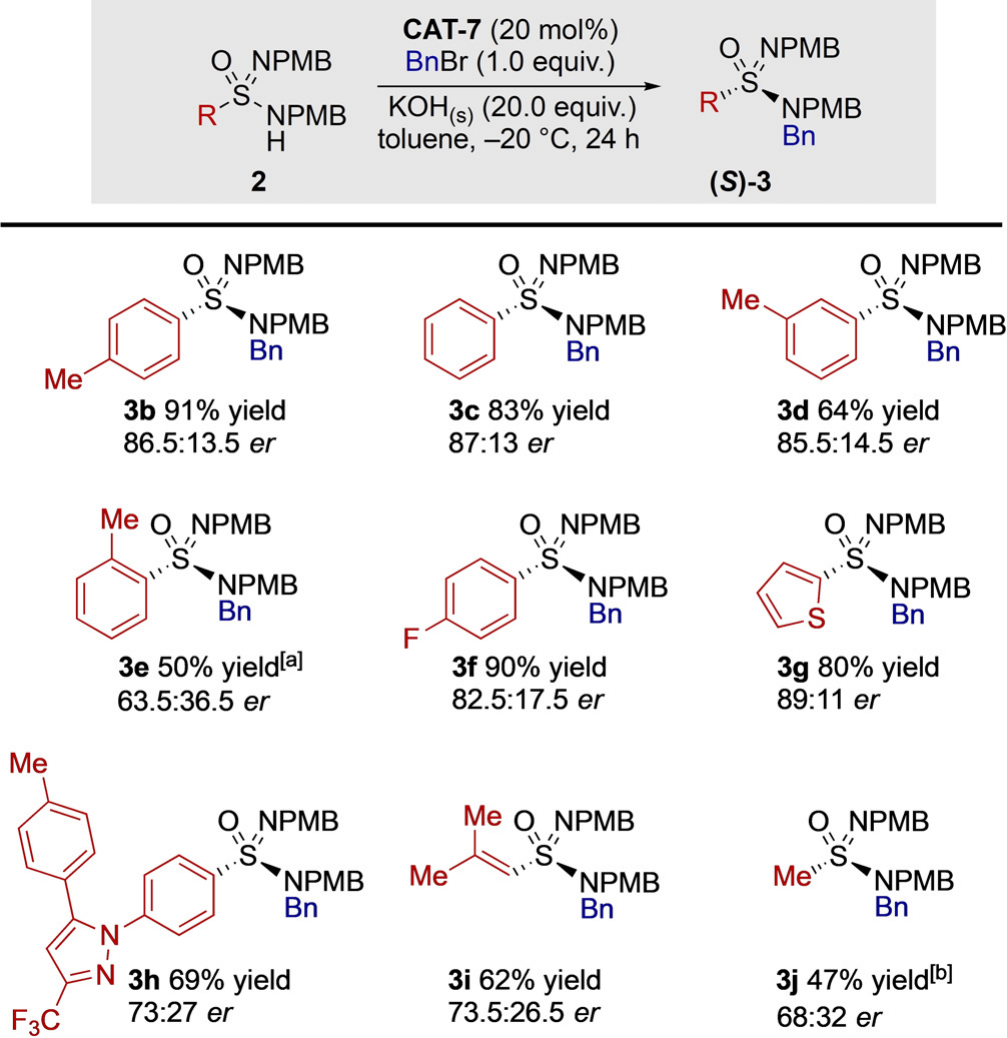
Exploration of the generality of the organocatalysed alkylation by examining the scope of the sulfonimidamide. Reaction conducted with sulfonimidamide (1.0 equiv) at a concentration of 0.05 M in toluene with stirring at 1000 rpm. [a] Reaction time 48 h. [b] Reaction time 30 h. All *er* measurements taken on the bulk sample. Isolated yields.

As our reaction design requires two identical substituents on the two sulfonimidamide N‐atoms, we set out to showcase the full utility of our methodology by developing conditions to effectively remove the PMB protecting groups (Scheme 8). The imidic PMB group could be selectively removed using DDQ as an oxidant; the reaction proceeded with an excellent yield to provide **(*R*)‐4** with no loss of enantiomeric purity. From comparison to a similar deprotection sequence,^[23]^ it can be assumed that these reactions occur with complete retention of configuration. Removal of both PMB groups could be achieved in a single step by treatment with TFA at elevated temperatures. Again, the reaction occurred with no loss of stereochemical information and validates the configurational stability of the molecules in acidic conditions.

**Scheme 8:**
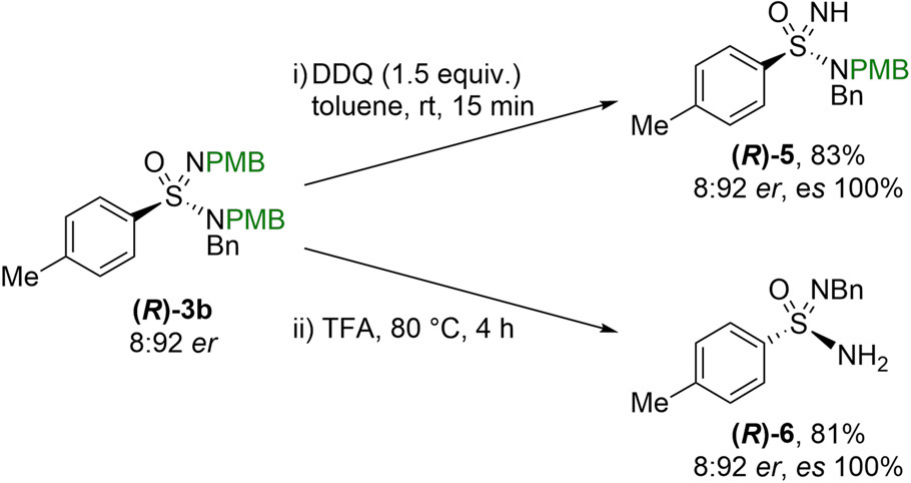
Deprotection strategy to access di‐ and mono‐ substituted sulfonimidamides.

A variety of enantioenriched aza‐S(IV) compounds have been reported to undergo racemisation at the sulfur stereocentre, including sulfinamides^[35]^ and sulfilimines.^[36]^ However, S(VI) compounds are known to have a greater degree of configurational stability, although examples of epimerisation at the sulfur of sulfonimidamides have been reported.^[37]^ Therefore, we were pleased to note that for the sulfonimidamides synthesised here, no degradation in enantiomeric ratio was observed even when subjected to several stress tests (see Supporting Information).

Within this report we have highlighted a unique class of aza‐sulfur compounds in which the two tautomeric forms are also enantiomeric. Using sulfonimidamides of this type we have established that epimerisation is rapid under a variety of conditions, and that these species can readily produce symmetric ions, creating opportunities for asymmetric catalysis. We have exploited this phenomenon to develop the first catalytic enantioselective desymmetrisation of sulfonimidamides. The process uses substoichiometric amounts of a highly modular chiral catalyst to control the configuration at the sulfur stereocentre, circumventing previous methods that rely on enantioenriched starting materials or adjacent stereocentres to form diastereomers. The developed method has delivered the most diverse range of enantiomerically enriched sulfonimidamides to date. However, most significantly, we anticipate that this fundamental principle will lead to further developments in the synthesis of asymmetric aza‐sulfur compounds.

## Conflict of interest

The authors declare no conflict of interest.

## Supporting information

As a service to our authors and readers, this journal provides supporting information supplied by the authors. Such materials are peer reviewed and may be re‐organized for online delivery, but are not copy‐edited or typeset. Technical support issues arising from supporting information (other than missing files) should be addressed to the authors.

Supporting Information
