# Supplementary material for: Exploiting Configurational Lability in Aza‐Sulfur Compounds for the Organocatalytic Enantioselective Synthesis of Sulfonimidamides
Source: Angew Chem Int Ed Engl. 2021 Nov 2;60(49):25680–7. doi: 10.1002/anie.202109160 (PMC9298307; doi:10.1002/anie.202109160)

Angewandte Chemie
Eine Zeitschrift der Gesellschaft Deutscher Chemiker

Supporting Information

Exploiting Configurational Lability in Aza-Sulfur Compounds for the Organocatalytic Enantioselective Synthesis of Sulfonimidamides

*Michael J. Tilby, Damien F. Dewez, Adrian Hall, Carolina Martínez Lamenca, and Michael C. Willis**

anie_202109160_sm_miscellaneous_information.pdf

Supporting Information for:

Exploiting Configurational Lability in Aza-Sulfur Compounds with the Organocatalytic Enantioselective Synthesis of Sulfonimidamides

Michael J. Tilby,[a] Damien F. Dewez,[a] Adrian Hall,[b] Carolina Martínez-Lamenca,[c] Michael C. Willis*,[a]

[a] Department of Chemistry, Chemistry Research Laboratories, University of Oxford, Oxford OX1 3TA, *Email:* Michael.Willis@chem.ox.ac.uk

[b] UCB Biopharma SPRL, 1420 Braine-l'Alleud, Belgium

[c] Neuroscience Medicinal Chemistry, Janssen Research & Development, 2340 Beerse, Belgium

Table of Contents

1. General Information S3
2. ^{1}H & ^{13}C NMR Studies on Tautomerization S5
    2.1. Solvent Effects on Tautomerization S5
    2.2. Temperature Effects on Tautomerization S9
    2.3. Additive Effects on Tautomerization S11
3. Optimisation of the Enantioselective Alkylation S17
    3.1. Optimisation with Singly-Quaternized Catalysts S17
    3.2. Optimisation with Doubly-Quaternized Catalysts S22
    3.3. Control Reactions S24
4. Determination of Configuration S25
5. Configuration Stress Test S26
6. Self-Disproportionation of Enantiomers (SDE) Evaluation S27
7. Experimental Procedures S28
    7.1. Preparation of Reagents S28
    7.2. Synthesis of an Unsubstituted Sulfonimidamide S31
    7.3. Sulfonimidamide Synthesis from a Sulfinamide S33
    7.4. Representative Examples of Catalyst Synthesis S34
    7.5. Synthesis of Mono-Substituted Sulfonimidamides S37
    7.6. Synthesis of Di-Substituted *N,N'*-Sulfonimidamides S45
    7.7. Enantioselective Alkylation of Sulfonimidamides S52
    7.8. Authentic Sample Synthesis S70
    7.9. Deprotections S71
8. Crystallography S73
9. References S74
10. HPLC-Data S75
11. NMR-Spectra S97

1. General Information

Reactions were conducted under an inert atmosphere of nitrogen, with anhydrous solvents, unless otherwise stated. Anhydrous THF, CH_2Cl_2 and toluene were obtained by passing through alumina columns using an Innovative Technology Inc. PS-400-7 solvent purification system, these solvents were then degassed with nitrogen prior to use, all other solvents were purchased as anhydrous from a commercial source. All glassware was oven dried (> 80 °C) for at least 16 h prior to use and allowed to cool to room temperature under a positive pressure of nitrogen. When low temperature (< 0 °C) had to be maintained for long periods of time an Easymax 102 Advanced Thermostat system was used or a FT902 FT immersion cooler depending on the reaction in question, otherwise an ice bath or acetone/dry ice bath was used. The reagent thionyl chloride was distilled under a nitrogen atmosphere prior to use. The reagent *t*-butyl hypochlorite was prepared according to the procedure outlined by Mintz-Walling.[1] Organometallic reagents in solution were bought from a commercial source and titrated prior to use, the volume used was then adjusted to ensure the equivalents used was constant on repeat experiments. 4-Methoxybenzaldehyde was distilled prior to use via short path distillation with a Kugelrohr. Potassium hydroxide was ground to a fine powder with a pestle and mortar before use as a base in reactions. *p*-Toluenesulfinic acid sodium salt was dried under vacuum at 100 °C for 4 h prior to use. All other reagents were purchased from Sigma-Aldrich Chemical Co. Ltd., Alfa Aesar, Acros Organic Ltd., Fluorochem Ltd., Strem Chemicals Inc. or Insight Biotechnology Ltd. and were used as supplied.

^{1}H NMR spectra were recorded on a Bruker AVIII400 (400 MHz) or AVII500 (700 MHz) spectrometer, ^{13}C NMR were recorded on a Bruker AVIII400 (101 MHz), AVII 500 (126 MHz) or AVIII HD 500 (126 MHz) spectrometer and ^{19}F NMR were recorded on a Bruker AVIII400 (377 MHz). Chemical shifts (δ_H, δ_C and δ_F) are quoted in ppm and relative to tetramethylsilane (δ_H = 0 ppm) and referenced to the residual solvent peaks. Coupling constants (*J*) are quoted in Hz and rounded to the nearest 0.5 Hz, with the following abbreviations used: s - singlet, br. - broad, app. - apparent, d - doublet, t - triplet, q - quartet, p - pentet, m - multiplet, dd - doublet of doublets, etc. Spectra assignments were determined with the use of chemical shifts, coupling constants, two-dimensional (COSY, HSQC and HMBC) NMR spectroscopy and by comparison to spectra of other compounds.

Low resolution mass spectra were recorded on a Waters LCT Premier mass spectrometer. High resolution mass spectra were recorded by the mass spectrometry service at the

Chemistry Research Laboratory, University of Oxford, using a Bruker Daltonic µTOF spectrometer (ESI). m/z values are reported in Daltons (Da) and high-resolution values are calculated to four decimal places from the molecular formula, with all found values in a tolerance of 5 ppm.

Infrared spectra (IR) were recorded using a Bruker Tensor 27 Fourier Transform spectrometer using a diamond ATR module. Absorption maximum v_{max} are given in wavenumbers (cm^{-1}).

Melting points (MP) are recorded in degrees Celsius (°C) using a Leica Galen III hot-stage microscope apparatus, with the crystallisation solvent reported in parenthesis.

Thin layer chromatography (TLC) was performed on Merk aluminium silica gel 60 F254 precoated plates with visualisation under a UV lamp (λ_{max} = 254 or 365 nm) or by staining with potassium permanganate solution. The retention factors (R_f) are reported with the solvent system used in parenthesis. Reactions were followed by TLC when practical. Flash column chromatography (FCC) was carried out on Merk silica gel 60 (230-400 mesh) and the solvent system used reported in parenthesis, for select examples a Biotage was used for this process. Petrol refers to the fraction of light petroleum ether boiling in the range of 40-60 °C.

Optical rotations were measured on a Schmidt Haensch UniPol L2000 polarimeter at 589 nm, 25 °C. $[\alpha]_D^{25}$ is expressed in deg cm^3 g^{-1} dm^{-1} and *c* is expressed in g 100 cm^{-3}. The enantiomeric ratio (*er*) was determined by chiral stationary phase HPLC in a Dionex P680 chromatogram with a Dionex UVD170U detector (λ_{max} = 225, 250, 275 or 300 nm) using a flow rate of 1.0 mL min^{-1} with either a Daicel Chiralpack AD-H, OD-H or IA-3 column. The eluent used and retention times (τ_{major} and τ_{minor}) are given in parentheses. In an analogous procedure compound **3b** was also analysed on a Dionex UltiMate 3000 chromatogram and compounds **4** and **5** analysed on an Agilent 1260 Series chromatogram.

Compound names are generated by CambridgeSoft ChemBioDraw Ultra 12.0, whilst phase-transfer catalyst names are based on the parent alkaloid, alkylating agent and nature of the anion.

2. ^{1}H & ^{13}C NMR Studies on Tautomerization

2.1. Solvent Effects of Tautomerization

The ^{1}H and ^{13}C NMR spectrum of compound **2b** was evaluated in multiple solvents at room temperature ($CDCl_3$, CD_2Cl_2, C_6D_6, toluene-d_8 and DMSO-d_6).

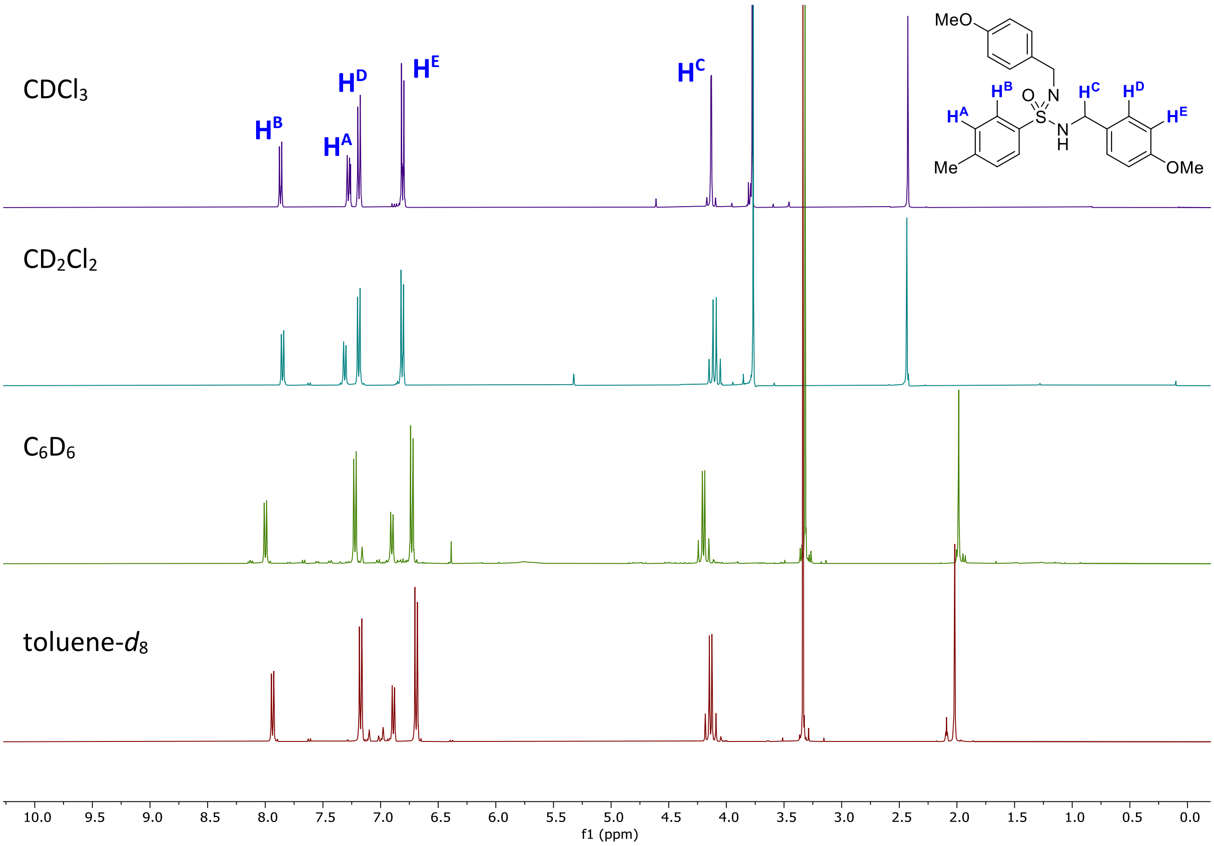

Figure S1. ^{1}H NMR spectrum of compound **2b** at 400 MHz and 295 K analysing the tautomerization process in various solvents.

The ^{1}H NMR spectrum of **2b** in $CDCl_3$, CD_2Cl_2, C_6D_6 and toluene-d_8 showed 8 distinct protons environments (Figure S1), indicating that the two PMB groups are observed to be chemically and magnetically equivalent on the NMR timescale. A key feature of all these spectra is that there is no observable N*H* environment, presumably due to rapid tautomerization. This is supported by the peaks at ~4.15 ppm that correspond to two distinct diastereotopic protons (H^C, NCH_2), with all spectra providing the same coupling constant of J = 14.0 Hz, the fact that these protons only couple to each other and are well resolved implies that coupling to an N*H* environment is not observed and that the chemical exchange process is fast.

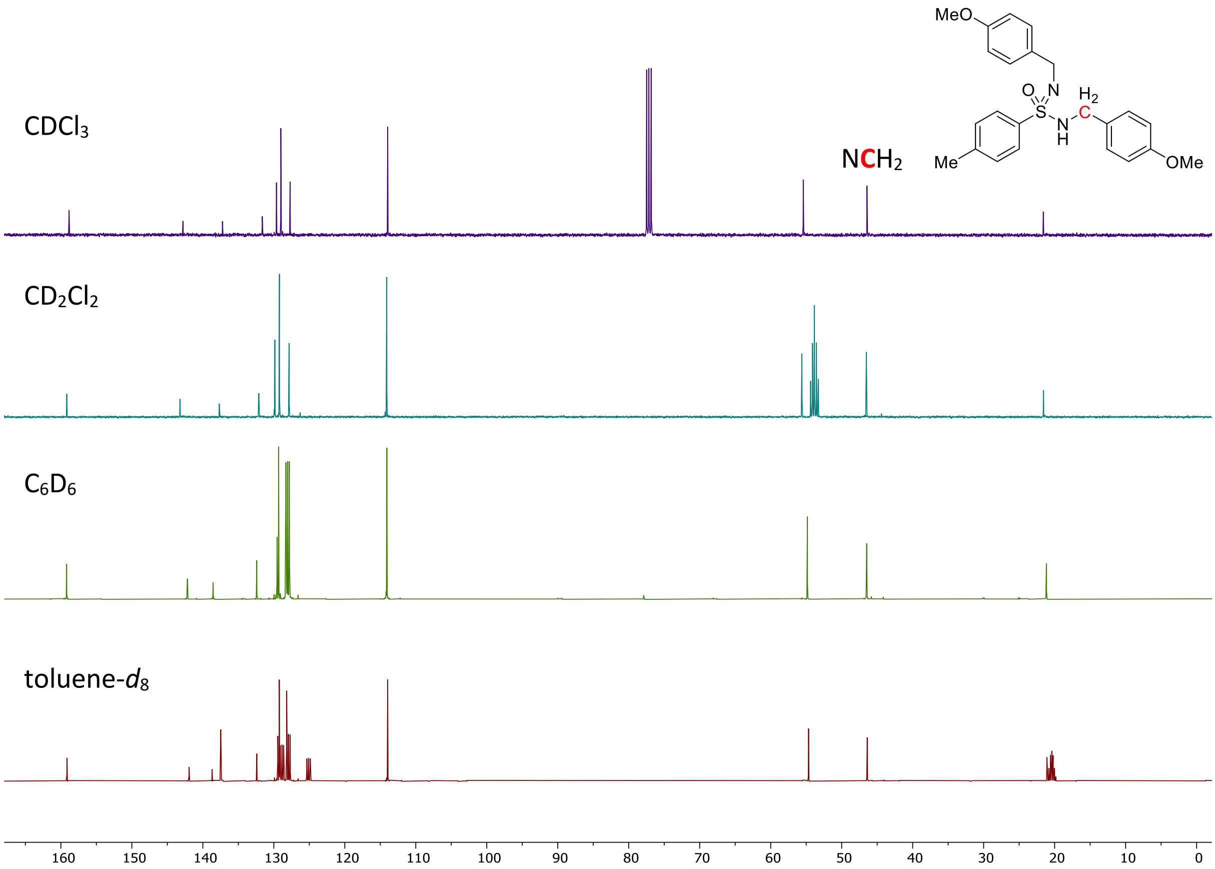

Figure S2. ^{13}C NMR spectrum of compound **2b** at 101 MHz and 295 K analysing the tautomerization process in various solvents.

The ^{13}C NMR spectrum of **2b** is near identical regardless of solvent (Figure S2). The observation that there is only one sharp carbon peak at 46.5 ppm, corresponding to a single methylene carbon environment rather than two, supports the previous conclusion that an interconversion between the S–N and S=N is occurring in each solvent.

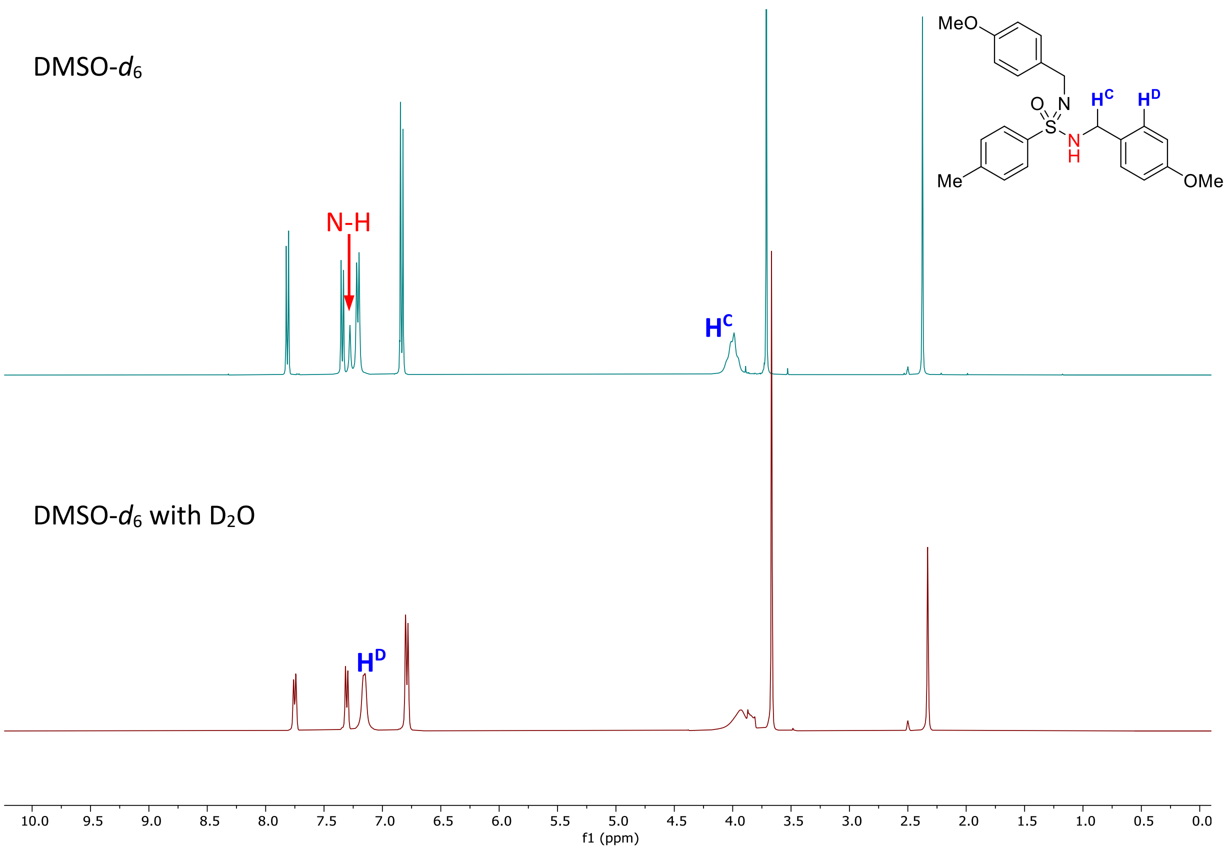

Figure S3. ^{1}H NMR spectrum of compound **2b** at 400 MHz and 295 K analysing the tautomerization process in DMSO-d_6, and subsequent performing of a D_2O-shake experiment.

Intriguingly the ^{1}H NMR spectrum in DMSO-d_6 now showed an additional broad peak at 7.28 ppm which integrated to 1 proton, furthermore this peak has a similar chemical shift reported for sulfonimidamide N*H* protons in the literature.[2] We confirmed this proton to be an exchangeable proton by performing a "D_2O shake" experiment, as indicated by its disappearance in the spectra (Figure S3). In addition, in DMSO-d_6 the NCH_2 protons (H^C) now produced a broad multiplet due to the rate of proton exchange being sufficiently slowed and potential coupling to the N*H*. We were also able to see a significant degree of broadening for one of the aryl protons on the PMB group (H^D) further supporting the conclusion that in DMSO-d_6 the PMB groups are observed to be chemically and magnetically inequivalent on the NMR timescale.

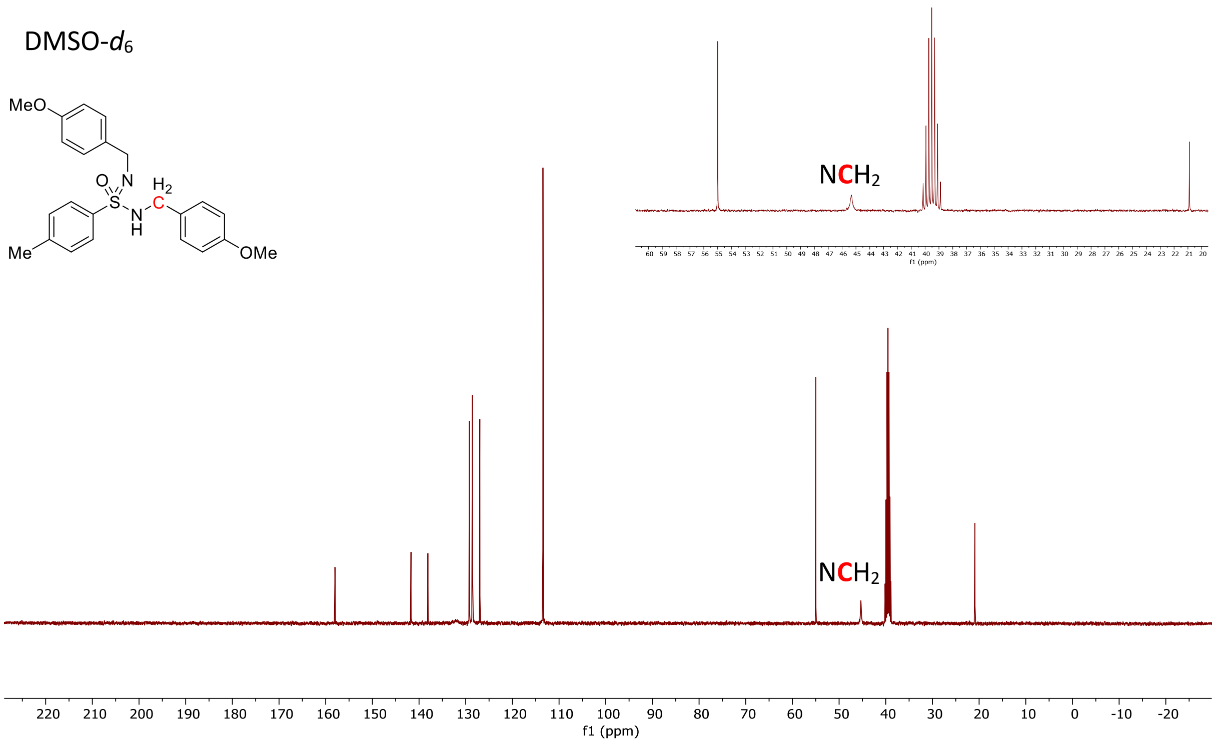

Figure S4. ^{13}C NMR spectra of compound **2b** at 101 MHz and 295 K analysing the tautomerization process in DMSO-d_6.

Examination of the ^{13}C NMR in DMSO-d_6 (Figure S4) now showed the methylene carbon signal at 45.3 ppm to be characteristically very different to the previous spectrum (Figure S2). In particular significant broadening of the peak at 45.3 ppm arises, indicating that this carbon environment consists of multiple peaks involved in a chemical exchange process. Also, the aromatic region only shows 7 clean signals which may arise from broadening of an aromatic quaternary centre. Overall, we were able to show that by using DMSO-d_6 as solvent a sufficient reduction in the rate of tautomerization occurs, causing a broadening of peaks due to the chemical inequivalence of the environments.

2.2. Temperature Effects on Tautomerization

To further probe the tautomerization of sulfonimidamides we performed a ^{1}H NMR analysis on **2b** at the varying temperature between room temperature and –80 °C. These experiments were conducted in toluene-d_8, which proved beneficial due to the relevance in our enantioselective asymmetric alkylation.

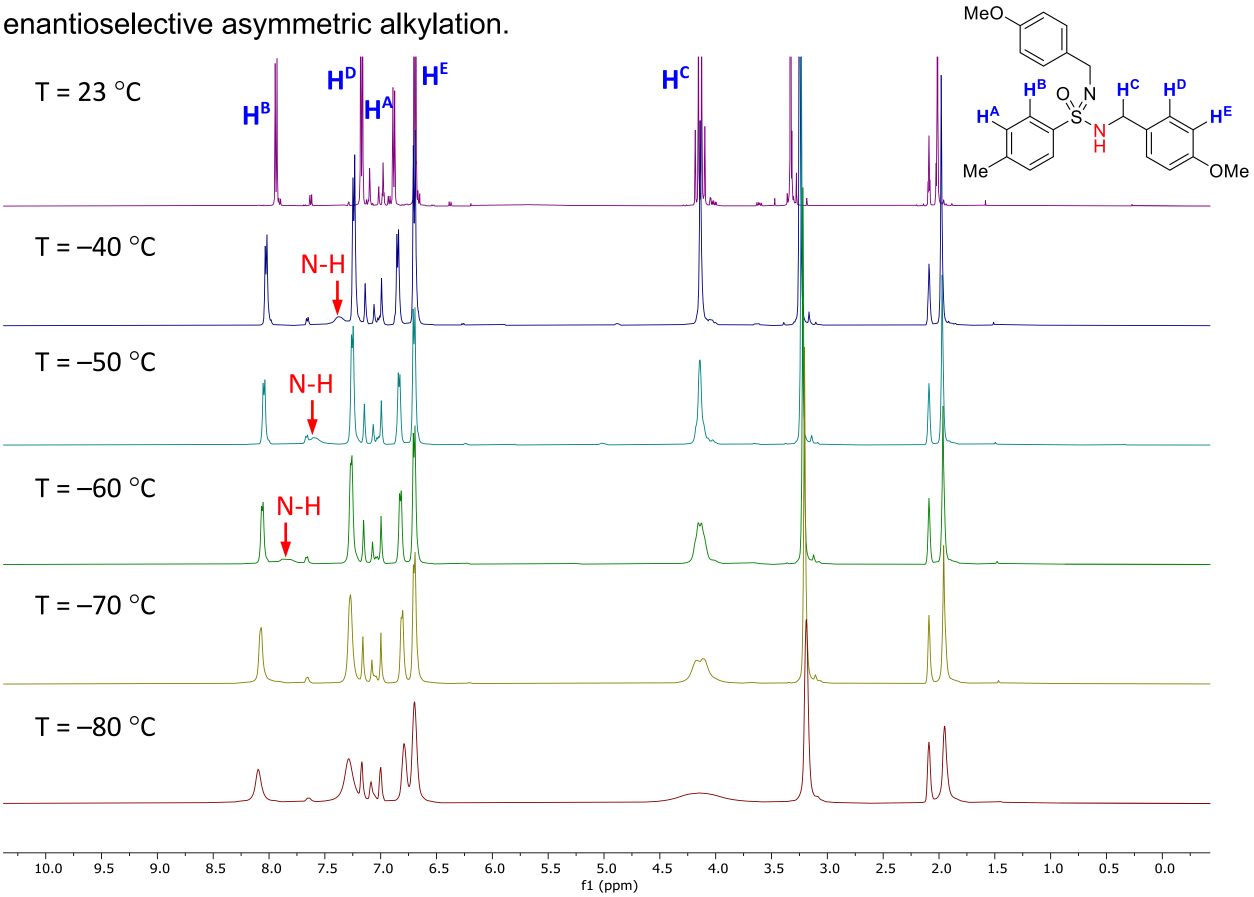

Figure S5. ^{1}H NMR spectrum of compound **2b** at 500 MHz and various temperatures analysing the tautomerization process in toluene-d_8.

Initially we compared the ^{1}H NMR spectrum of **2b** at room temperature and –40 °C, in doing so we noted that at the lower temperature there was now a broad peak observed at 7.37 ppm. This peak has tentatively been assigned as the N*H* proton of the sulfonimidamide, due to the previous results (Figure S3). The broad nature at this temperature can be assumed to be due to this proton undergoing a chemical exchange process, hence at –40 °C, tautomerization, and in turn racemisation, would still persist. At lower temperatures such as –80 °C the N*H* proton is no longer observed which may arise from a combination of the chemical exchange process, partial averaged coupling to the adjacent NCH_2 and ^{14}N (I = 1), as well as inhomogeneity in the sample.

Another key observation from these ^{1}H NMR spectrum is that the diastereotopic protons, NCH_2, at room temperature appear as two doublets with close chemical shifts (NC$H_A$$H_B$ & NC$H_A$$H_B$ with 4.17 & 4.11 ppm respectively, and J = 14.0 Hz). On reducing the temperature to

–40 °C these peaks appear to coalesce, likely due to the variation of chemical shift with temperature causing an apparent singlet (similar apparent singlets are observed at room temperature with related compounds **2g** and **2j**). The fact that this peak has no observable coupling to the N*H* proton supports our previous assumption that significant tautomerization is still occurring at this temperature. Further lowering of the temperature to –60 °C now shows the presence of two separate peaks, however it cannot be distinguished if this is from chemical inequivalence of the diastereotopic protons (NCH_AH_B & NCH_AH_B) or additionally between the protons at the imidic and amidic site ($S{=}NCH_2$ & $S{=}N(H)CH_2$). At –80 °C the signal of these peaks has become exceptionally broad, especially when compared to the other proton environments, which could imply the presence of multiple low intensity signals as would be expected from the multiple chemically inequivalent environments.

The key feature of this series of ^{1}H NMR experiments is that they provide evidence that the N*H* proton of the sulfonimidamide is able to undergo rapid chemical exchange, via tautomerization that is certainly persisting at the low temperature of –40 °C. It is also likely that significant tautomerization occurs at lower temperatures, but this is challenging to conclude from the current results. Regardless this has significance for any asymmetric catalysis occurring at cryogenic temperatures in toluene.

2.3. Additive Effects on Tautomerization

When **2b** ^{1}H & ^{13}C NMR spectrum in DMSO-d_6 were analysed, the tautomerization was sufficiently slowed to allow analysis of the N*H* proton as well as significant broadening of the methylene environment. We decided to examine the effects of additives including TBAB (a phase-transfer catalyst) and KOH (a base), as well as their effects in combination.

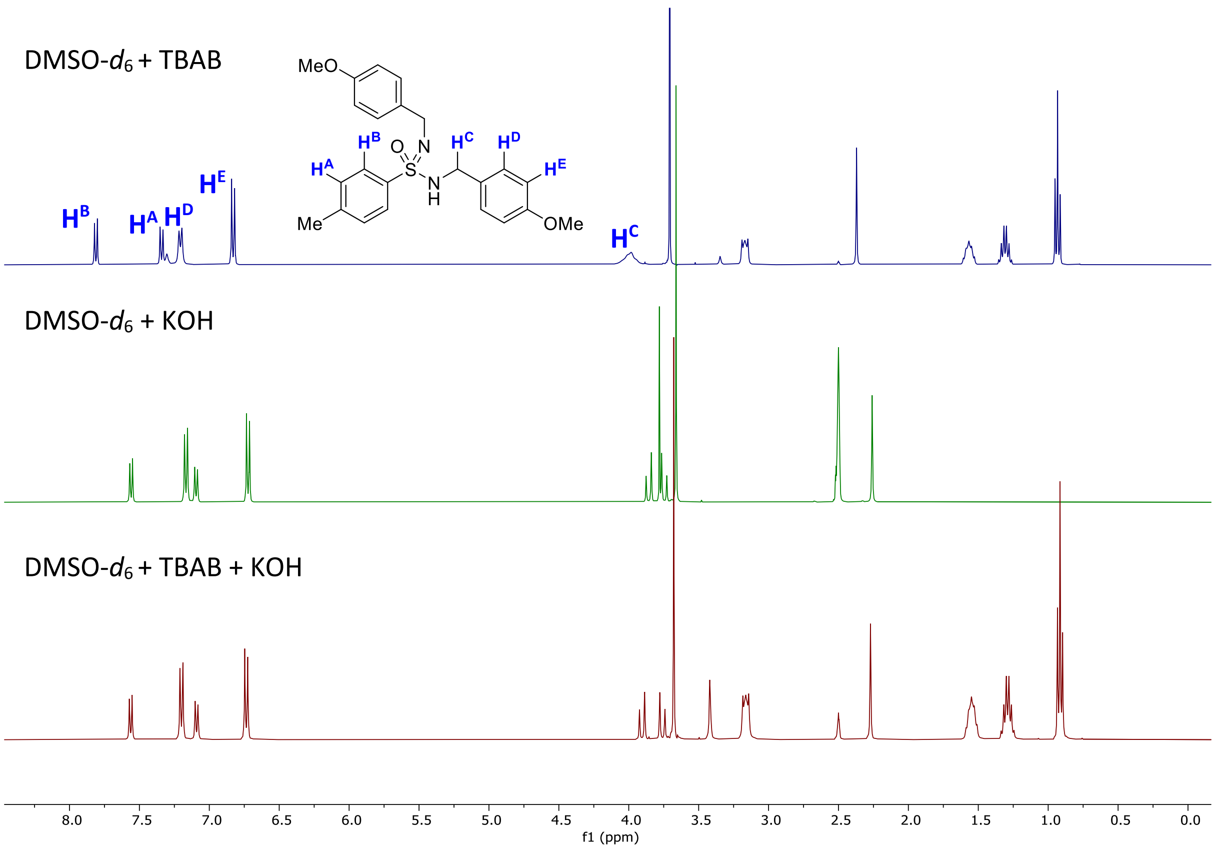

Figure S6. ^{1}H NMR spectrum of compound **2b** at 400 MHz and 295 K analysing the tautomerization process with the additives TBAB and KOH.

First of all, we noted that addition of TBAB showed no significant impact on the ^{1}H NMR spectrum of **2b** showing that the rate of tautomerization was still sufficiently slow (Figure S6). However, addition of KOH produced a variety of changes to the spectrum, firstly the N*H* proton is no longer observable and secondly the methylene peaks can now be observed as two distinct diastereotopic protons, now with J = 14.5 Hz. Finally, it is also noticeable that several of the proton environments close to the nitrogen heteroatoms are shifted upfield, with this most significant proton H^B (7.81 ppm to 7.56 ppm), which may indicate the formation of the sulfonimidamide anion.

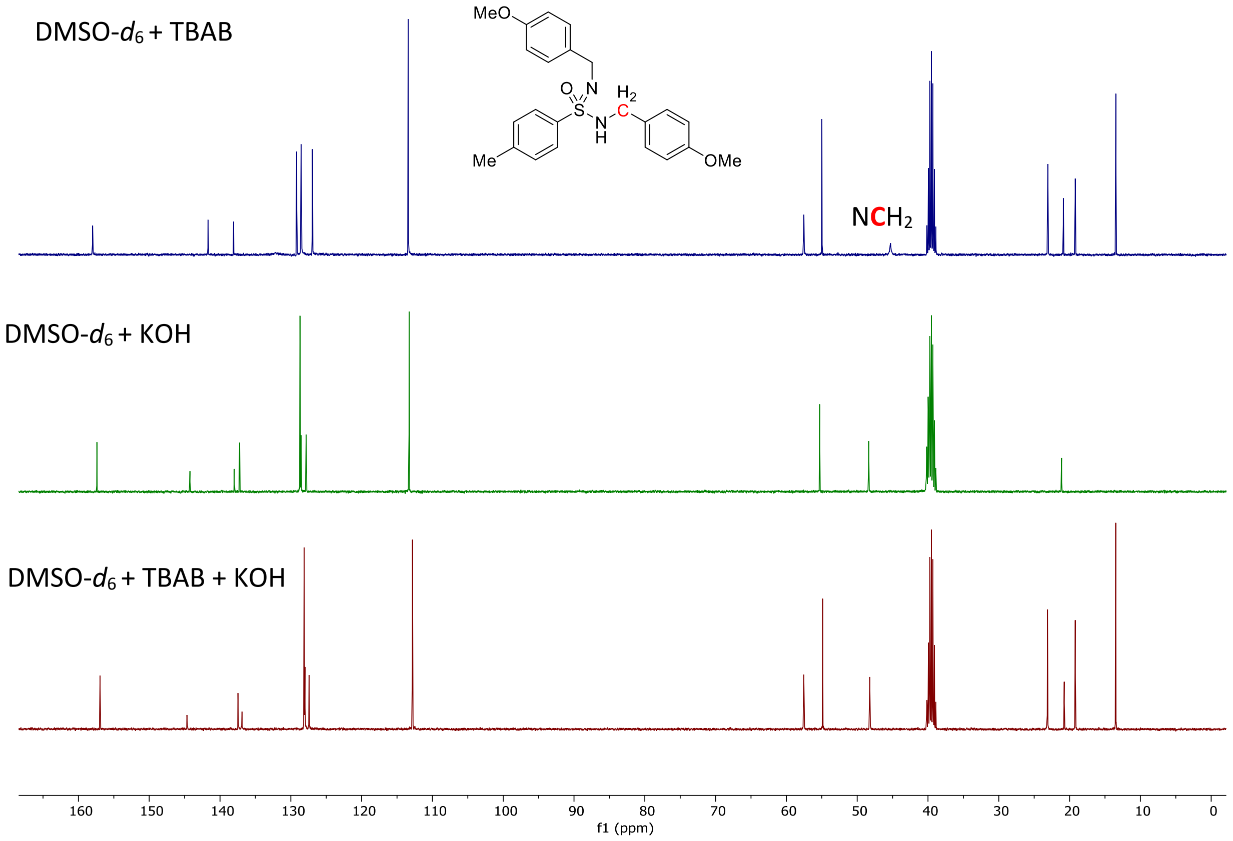

Figure S7. ^{13}C NMR spectrum of compound **2b** at 101 MHz and 295 K analysing the tautomerization process with the additives TBAB and KOH.

Examination of the ^{13}C NMR spectrum (Figure S7) again confirmed TBAB had minimal impact, but addition of potassium hydroxide reformed the sharp N*C*H_2 methylene peak. Previously the NMR signals were shown to be insensitive to the solvent (Figure S1), however now significant chemical shift difference can be observed on addition of KOH, which we ascribe to be from the formation of the symmetric sulfonimidamide anion. Therefore, the addition of KOH seems to at the very least give rise to an accelerated rate of tautomerization, but it is likely that this in fact generates the anion in solution.

To further examine whether the change in chemical shifts observed on addition of KOH was in fact a consequence of the sulfonimidamide deprotonation, we applied the same experiment to sulfonimidamide **3b**, a sulfonimidamide which does not contain any N*H* protons to deprotonate.

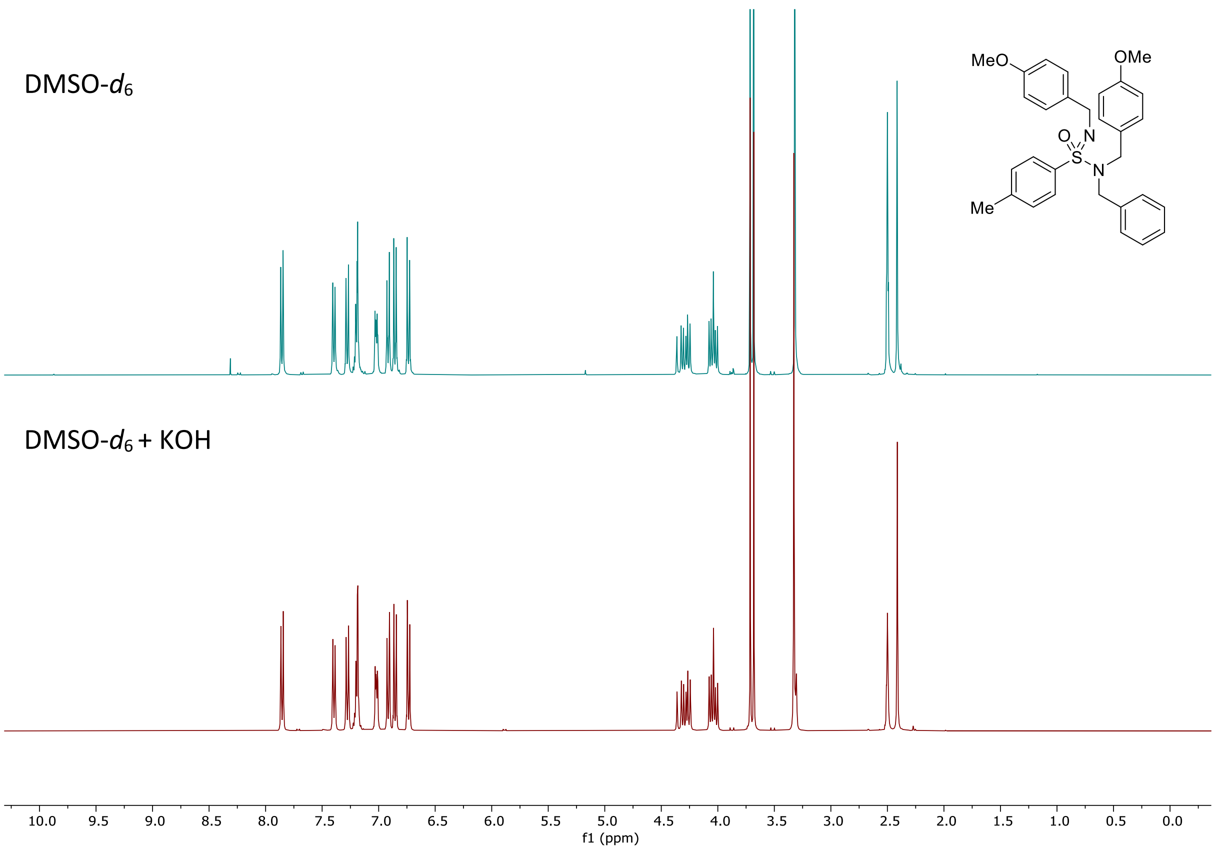

Figure S8. ^{1}H NMR spectrum of compound **3b** at 400 MHz and 295 K analysing the effect of KOH as an additive.

Comparison of the ^{1}H NMR spectrum of **3b** under neutral and basic conditions in DMSO-d_6 (Figure S8) showed no significant change in the chemical shifts or coupling constants of any of the protons. This result indicates that the presence of KOH does not significantly impact the ^{1}H NMR spectra of **3b** and the previous changes seen for **2b** (Figure S6) arise due to deprotonation.

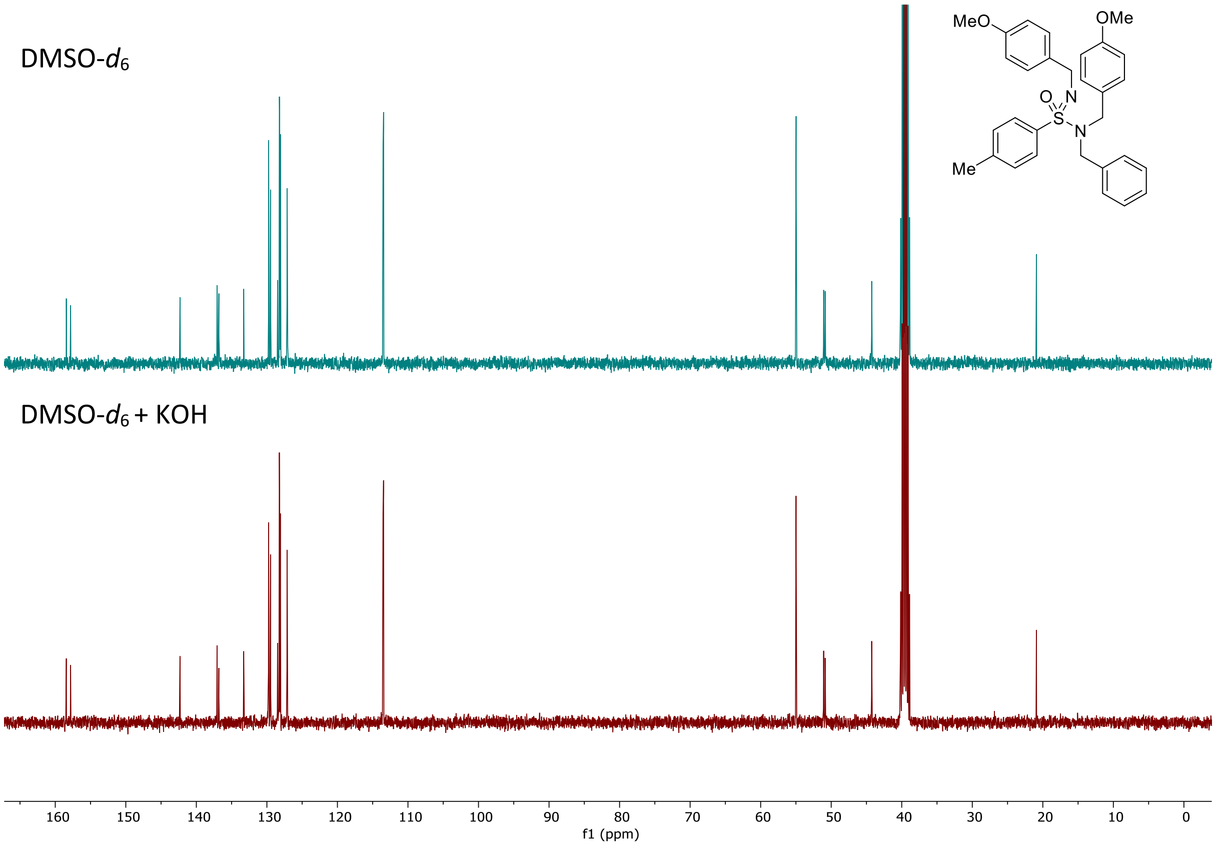

Figure S9. ^{13}C NMR spectrum of compound **3b** at 101 MHz and 295 K analysing the effect of KOH as an additive.

The comparison of the ^{13}C NMR spectrum in DMSO-d_6 under neutral and basic conditions for **3b** (Figure S9) gave an analogous conclusion to that previously discussed, with no significant change in any of the carbon chemical shifts.

Therefore, we have demonstrated that addition of base leads to the formation of a symmetric anion. Therefore, by analogy, protonation of the sulfonimidamides would also lead to symmetric cation species. To probe this, we added acid (TFA) to a sample of **2b** in DMSO-d_6 (where tautomerization is sufficiently slow).

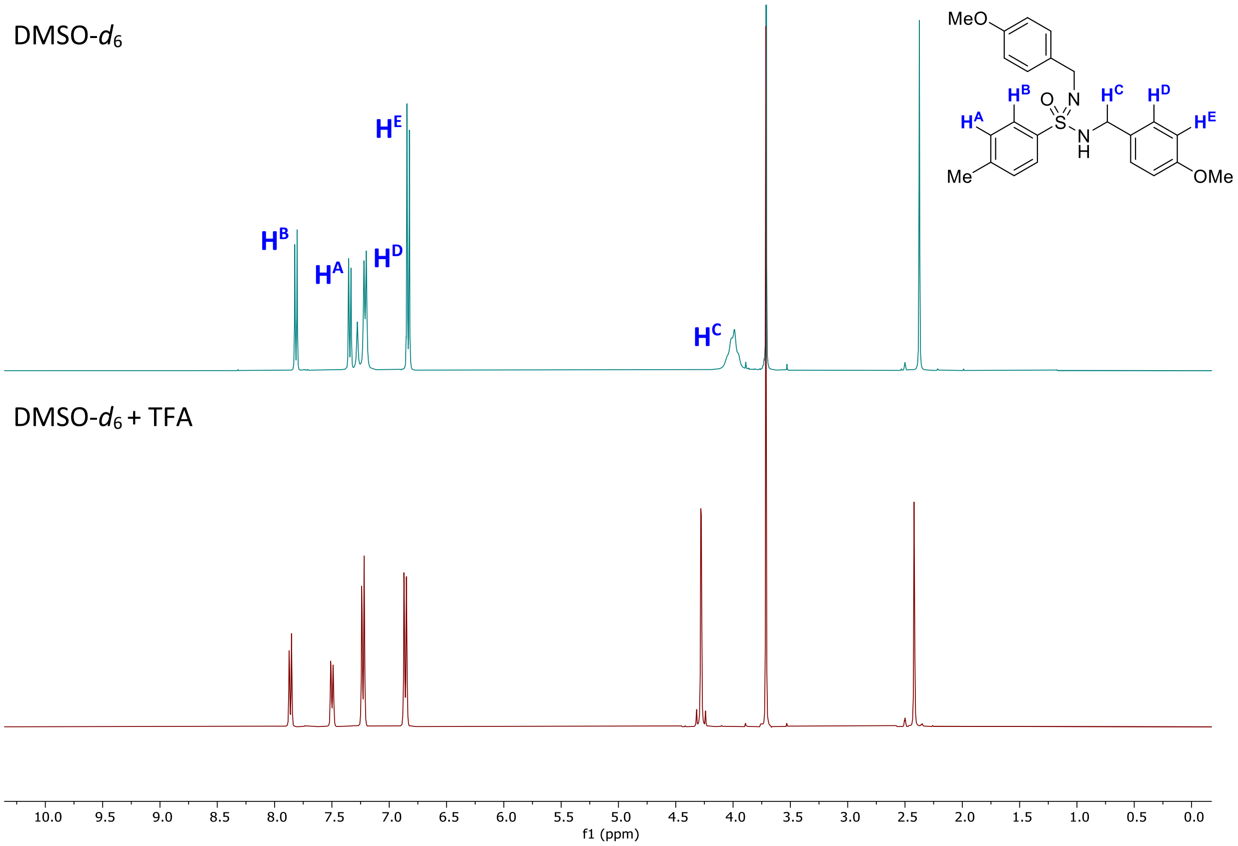

Figure S10. ^{1}H NMR spectrum of compound **2b** at 400 MHz and 295 K analysing the tautomerization process with TFA as an additive.

The addition of TFA to **2b** in DMSO-d_6 caused the N*H* signal to no longer be present (Figure S10), and the methylene proton signal to once again become sharp well resolved peaks. These observations indicate that TFA either increases the rate of tautomerization or it forms the proposed symmetric cation. Analysis of the chemical shifts indicate that most environments are not drastically affected by the addition of TFA, except H^A and H^C, which are shifted downfield, furthermore the coupling constants for the diastereotopic protons H^C has now increased (J = 14.5 Hz). Although the changes in the spectrum are not as predominant as with KOH, the differences presented may still indicate that a cationic species is present.

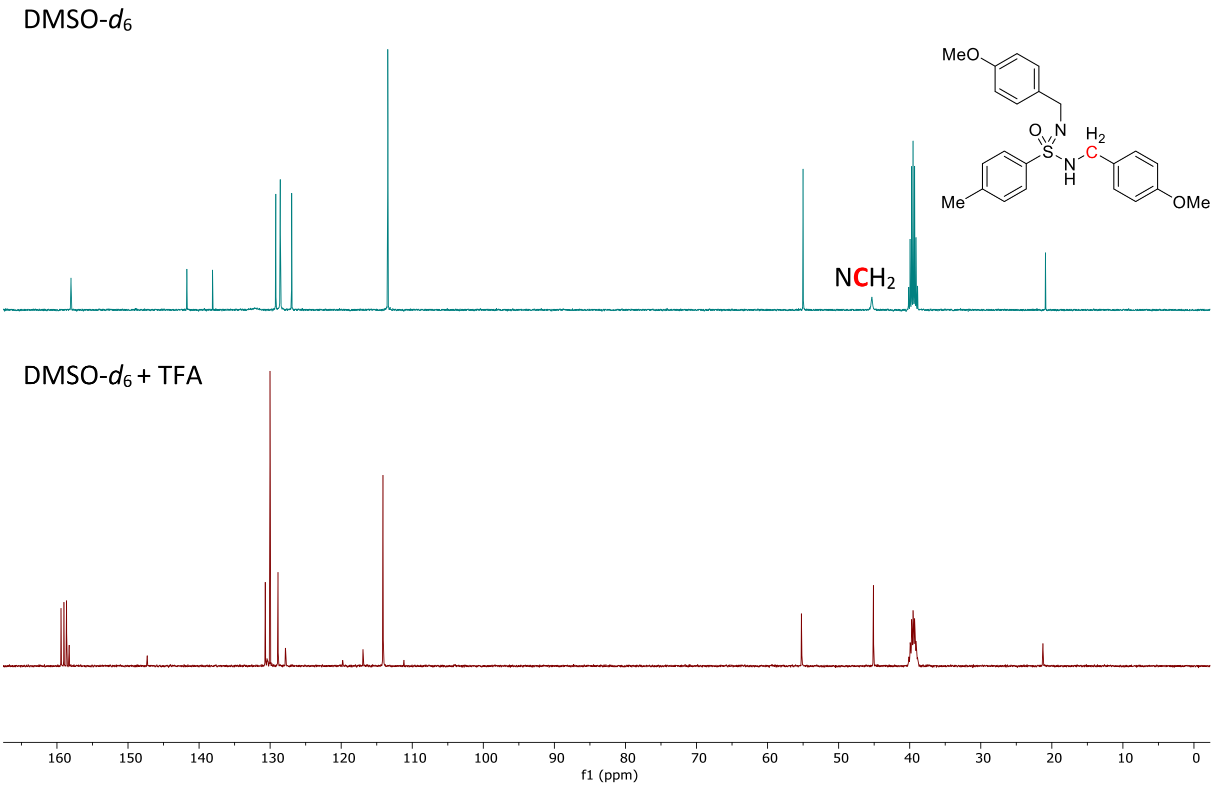

Figure S11. ^{13}C NMR spectrum of compound **2b** at 101 MHz and 295 K analysing the tautomerization process with TFA as an additive.

Examination of the ^{13}C NMR spectrum (Figure S11) now indicates that the methylene signal is no longer broad supporting the conclusion that addition of acid causes the PMB groups to appear chemically equivalent. Another major difference between the neutral and acidic ^{13}C NMR spectrum is that there is a significant change in chemical shift for signals in the aromatic region (in particular a quaternary centre is now shifted downfield to 147.3 ppm), this observation leads us to conclude that the protonated sulfonimidamide is a significant component of the sample.

3. Optimisation of the Enantioselective Alkylation

3.1. Optimisation with Singly-Quaternized Catalysts

Table S1. Initial Catalyst Screen

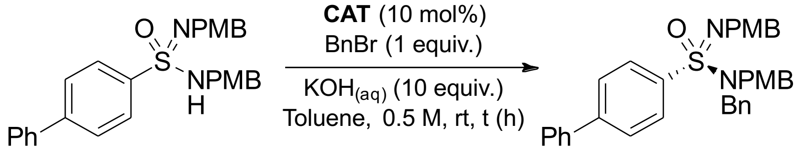

2a **3aa**

Entry	CAT	Time (h)	Conversion (%)	*er*
1	**CAT-8**	2	100	63:37
2	**CAT-9**	2	100	64.5:35.5
3	**CAT-10**	1	100	69:31

Sulfonimidamide (1 equiv.). Aqueous refers to the base as a 50% wt. solution in water, and conversions determined by analysis of crude ^{1}H NMR spectrum.

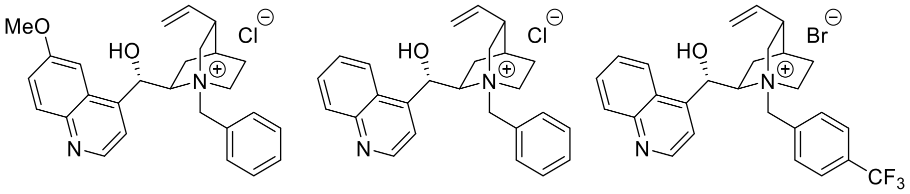

CAT-8 **CAT-9** **CAT-10**

Initial results showed that **CAT-8** produced a highly reactive system with some enantio-induction (entry 1). A minimal difference was observed between the quinidine and cinchonine derived catalysts, however due to the slight improvement with **CAT-9** (entry 2) this scaffold was selected for further catalyst screening. Pleasingly the commercially available catalyst **CAT-10** produced an increase in the enantiomeric ratio (entry 3), hence this was used for further screening of conditions.

Table S2. Initial Condition Screen: Base

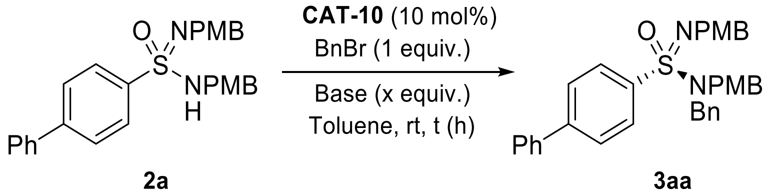

Entry	Base	Base equiv.	Conc. (M)	Time (h)	Conversion (%)	*er*
1	$KOH_{(aq)}$	10	0.50	1	100	69:31
2	$KOH_{(aq)}$	5	0.10	1	100	70:30
3[a]	$KOH_{(aq)}$	10	0.25	1	100	70:30
4[a]	$NaOH_{(aq)}$	5	0.50	1	96	72:28
5[a]	$K_2CO_{3(aq)}$	5	0.50	16	22	71:29
6[a]	$K_3PO_{4(aq)}$	5	0.50	16	44	71:29
7	$KOH_{(s)}$	5	0.10	1	100	72.5:27.5
8	$NaOH_{(s)}$	5	0.10	2	86	72:28
9	$LiOH_{(s)}$	5	0.10	16	0	N/A
10	$CsOH{\cdot}OH_{2(s)}$	5	0.10	1	98	67.5:32.5
11	$K_2CO_{3(s)}$	5	0.10	24	0	N/A
12	$K_3PO_{4(s)}$	5	0.10	24	36	77.5:22.5
12	$K_3PO_{4(s)}$	5	0.10	72	100	75:25
13	$PhONa_{(s)}$	5	0.10	16	0	N/A
14	$NaH_{(s)}$	5	0.10	24	68	66:34
15	*t*-$BuOK_{(s)}$	5	0.10	1	100	73:27

Sulfonimidamide (1 equiv.). Aqueous refers to the base as a 50**%** wt. solution in water, and conversions determined by analysis of crude ^{1}H NMR spectrum. [a] 20 mol% of **CAT-3**

A screen of bases, concentration and equivalents of base was conducted. This indicated that solid bases out-performed bases in aqueous solution. Although $K_3PO_{4(s)}$ gave increased enantioselectivity the conversion was drastically reduced (entry 12) and extension of reaction times reduced the *er* further. Overall solid KOH produced a compromise of excellent reactivity and good *er* (entry 7).

Table S3. Initial Condition Screen: Solvent

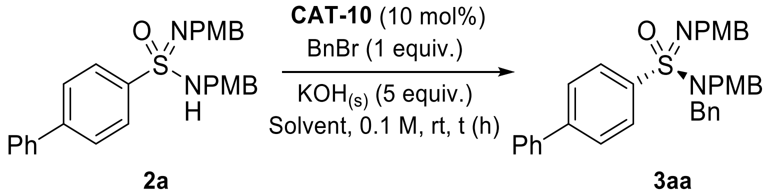

Entry	Solvent	Time (h)	Conversion (%)	*er*
1	Toluene	1	100	72.5:27.5
2	CH_2Cl_2	2	100	57:43
3	Et_2O	2	97	66:34
4	MTBE	1	97	68.5:31.5
5	*m*-Xylene	1	96	71.5:28.5
6	Chlorobenzene	1	96	68.5:31.5
7	Benzene	1	97	74:26

Sulfonimidamide (1 equiv.). Conversions determined by analysis of crude ^{1}H NMR spectrum.

A solvent screen revealed polar solvents gave a reduced *er*. Benzene did perform marginally better than toluene (entry 7), but toluene was selected as the solvent for its more beneficial properties including its higher freezing point.

Table S4. Initial Condition Screen: Electrophile

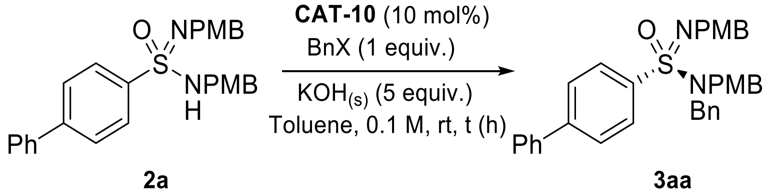

Entry	Electrophile	Time (h)	Conversion (%)	*er*
1	BnBr	1	100	72.5:27.5
2	BnCl	5	68	76:24
3	BnI	2	97	68.5:31.5
4	BnOTs	1	98	69.5:30.5

Sulfonimidamide (1 equiv.). Conversions determined by analysis of crude ^{1}H NMR spectrum.

A screen of electrophiles showed that BnCl gave a higher *er*, however this was at the determent of reactivity, hence this electrophile was deemed inappropriate for use in reactions that operate at a lower temperature.

Table S5. Catalyst Screen

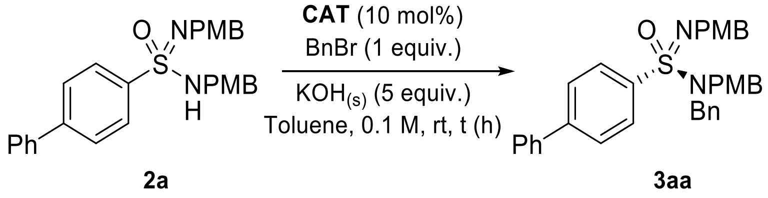

Entry	Catalyst	Time (h)	Conversion (%)	*er*
1	**CAT-1**	1	100	74:26
2[a]	**CAT-1**	24	49	78:22
3[b]	**CAT-1**	6	74	75.5:24.5
4	**CAT-9**	3	100	63.5:36.5
5	**CAT-11**	1	87	63:37
6	**CAT-12**	1	100	30.6:69.5
7	**CAT-13**	4	100	53:47
8	**CAT-14**	1	95	59:41
9	**CAT-15**	24	76	52:48
10	**CAT-16**	1	100	73:27
11[a]	**CAT-16**	24	45	76:24
12[b]	**CAT-16**	16	0	N/A
13[c]	**CAT-16**	24	0	N/A
14	**CAT-17**	6	100	56.5:43.5
15	**CAT-18**	1	95	65.5:34.5
19	**CAT-19**	1	85	54.5:45.5
20	**CAT-20**	1	96	69.5:30.5
21	**CAT-21**	8	67	57.5:42.5
22	**CAT-22**	1	56	60:40
23	**CAT-23**	5	53	53.5:46.5
24	**CAT-24**	24	47	54.5:45.5

Sulfonimidamide (1 equiv.). Conversions determined by analysis of crude ^1H NMR spectrum. [a] Base used is $K_3PO_{4(s)}$ (5 equiv.). [b] BnCl (1.5 equiv.) used in place of BnBr. [c] Reaction run at temperature of −20°C.

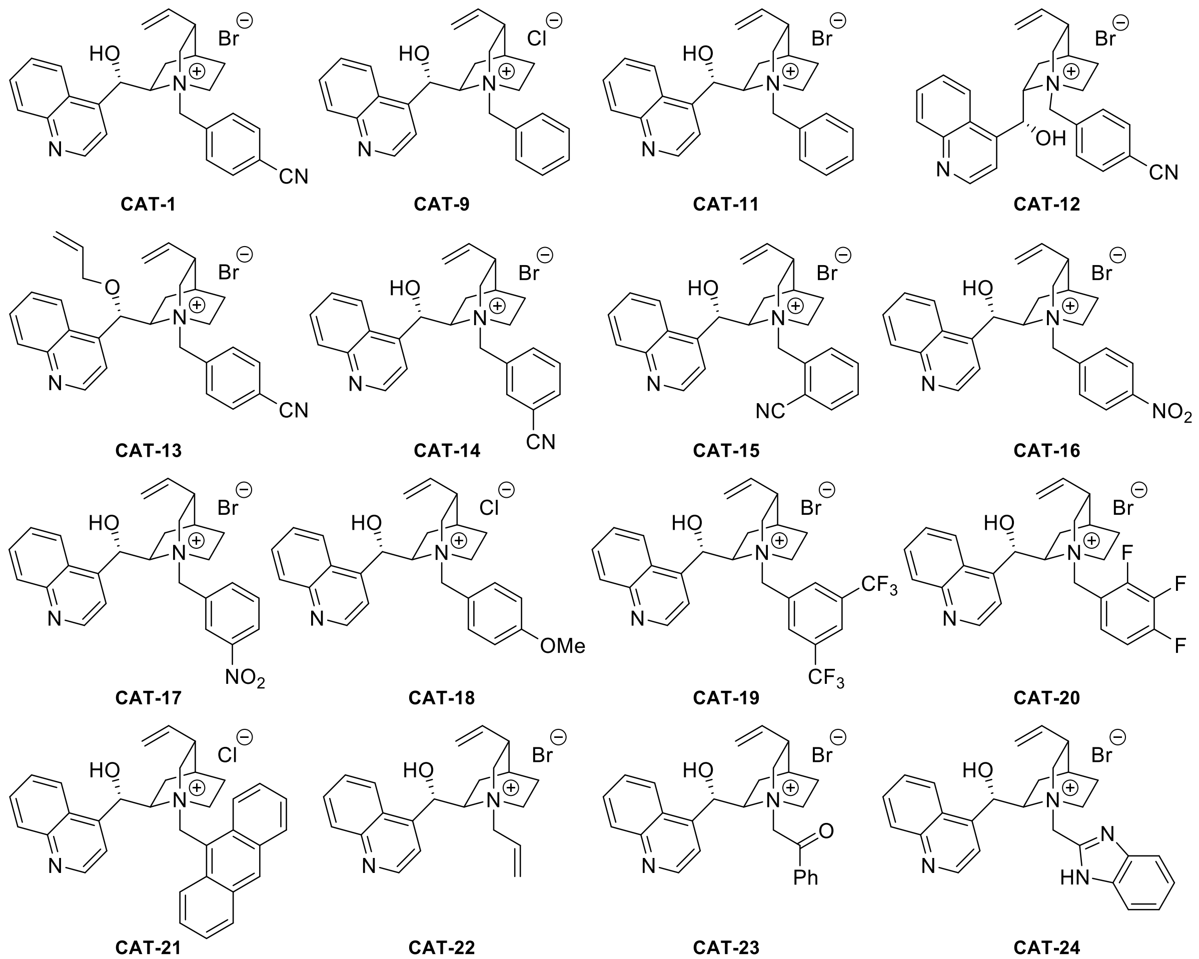

Table S5. Catalyst Screen; figure indicating the catalysts used for this screen.

The use of K_3PO_4 and BnCl were again evaluated using **CAT-1** but gave a similar conclusion to that previously seen (entries 2 & 3). Due to the modular nature of the catalysts and ease of synthesis, a library was developed and screened. The effect of the anion of the catalyst was examined by using **CAT-9** under the new conditions (entry 4) and compared with **CAT-11**, which showed no substantial effect. The pseudo-enantiomer of **CAT-1** (**CAT-12**) was also a component catalyst, now providing the opposite enantiomer albeit in a slightly reduced ratio. Another intriguing factor is that the hydroxyl group of the catalyst appeared essential for enantioinduction, as when **CAT-13** was employed good conversion was observed to a near racemic mixture of the product. All additional catalysts screened were inferior to **CAT-1**, and an electron-withdrawing para substituent was deemed essential. The closest result was **CAT-16**, therefore once again the use of K_3PO_4, BnCl and a lower temperature reaction were tested with **CAT-16** (entries 15-17) but this catalyst appeared to give reduced reactivity compared to **CAT-1**, deeming it unsuitable.

3.2. Optimisation with Doubly-Quaternized Catalysts

Table S6. Condition Screen: Catalyst Loading

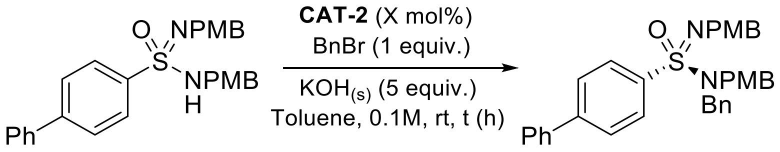

2a **3aa**

Entry	Catalyst (mol%)	Time (h)	Conversion (%)	*er*
1	10	7	61	68:32
2	1	16	76	53.5:46.5
3	20	1	94	75.5:25.5

Sulfonimidamide (1 equiv.). Conversions determined by analysis of crude ^{1}H NMR spectrum.

The catalyst loading was shown to have a dramatic effect on reactivity and *er*. Hence increasing **CAT-2** to 20 mol% gave an improved result when compared to the parent catalyst (**CAT-1**). Due to this result it was decided to further screen this motif of catalyst, in order to check for significant improvements.

Table S7. Further Catalyst Screen

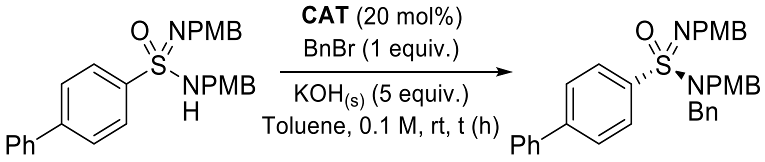

2a **3a**

Entry	Catalyst	Time (h)	Conversion (%)	*er*
1	**CAT-2**	1	94	75.5:25.5
2	**CAT-25**	4	100	69:31
3	**CAT-26**	4	100	70.5:29.5
4	**CAT-27**	1	95	68:32
5	**CAT-28**	24	70	62:38
6	**CAT-29**	6	88	50.5:49.5
7	**CAT-30**	48	100	44.5:55.5
8	**CAT-31**	48	53	49.5:50.5

Sulfonimidamide (1 equiv.). Conversions determined by analysis of crude ^{1}H NMR spectrum.

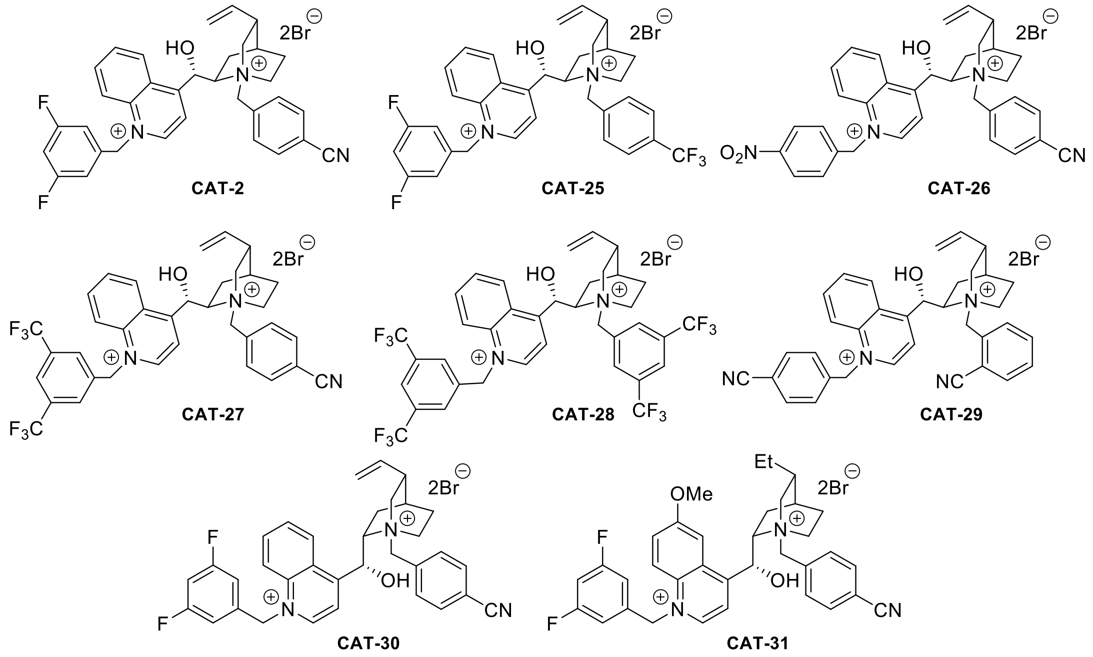

Table S7. Further Catalyst Screen; scheme indicating the catalysts used for this screen.

All catalysts screened under these conditions were inferior to **CAT-2**. However, comparison of **CAT-2**, with **CAT-29** (entries 1 & 6) exemplifies our conclusion that properties which were deemed necessary in our initial catalyst screen carried over to the *bis*-quarternized systems (i.e. compared to **CAT-15**). Interestingly the pseudo-enantiomer of **CAT-2**, **CAT-30** now has a drastic reduction in reactivity and stereoselective, which differs from our findings for the singly-quaternized system.

Table S8. Condition Screen: Base

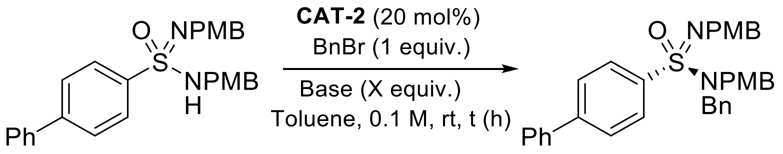

2a **3aa**

Entry	Base	Base (equiv.)	Time (h)	Conversion (%)	*er*
1	$KOH_{(aq)}$	5	24	100	66.5:33.5
2	$NaOH_{(s)}$	5	2	91	73.5:26.5
3	$K_2CO_{3(s)}$	5	96	40	60.5:39.5
4	$K_3PO_{4(s)}$	5	24	44	72:28
5	$KOH_{(s)}$	10	1	100	79.5:20.5
6	$KOH_{(s)}$	20	1	100	81:19

Sulfonimidamide (1 equiv.). Aqueous refers to the base as a 50% wt. solution in water, and conversions determined by analysis of crude ^{1}H NMR spectrum.

The bases were examined for the doubly-quaternized systems. It was shown that aqueous base was again detrimental to the enantioselectivity, while $K_2CO_{3(s)}$ and $K_3PO_{4(s)}$ also had a

reduced rate of reaction, as well as now showing a decrease in *er*. Increasing the equivalents of base showed some improvement in the stereoselectivity.

Table S9. Condition Screen: Electrophile

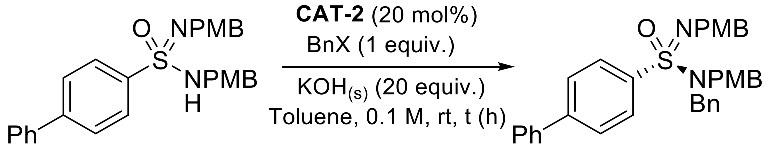

2a **3aa**

Entry	Electrophile	Time (h)	Conversion (%)	*er*
1	BnBr	1	100	81:19
2	BnCl	24	20	76:24
3[a]	BnI	4	90	66.5:33.5
4	BnOTs	1	95	71:29

Sulfonimidamide (1 equiv.). Conversions determined by analysis of crude ^{1}H NMR spectrum. [a] KOH (10 equiv.).

Re-examination of the electrophile confirmed our decision to use BnBr, as this maintained reactivity whilst producing the best *er*, overall resulting in our new set of conditions, for which further catalysts were screened resulting in **CAT-7**.

3.3. Control Reactions

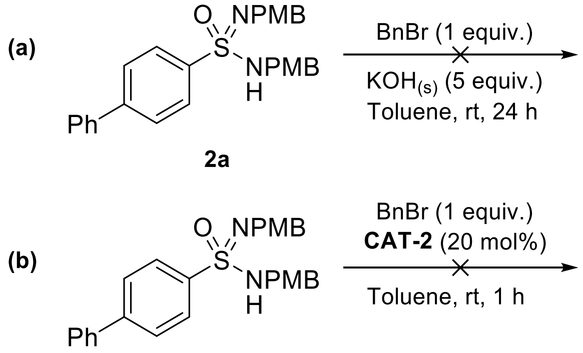

Scheme S1. Control reactions for the alkylation of sulfonimidamides. Sulfonimidamide (1 equiv.). and concentration 0.1 M. No reaction determined by analysis of crude ^{1}H NMR spectrum.

Control reactions were conducted on **2a** indicating that no background reaction was occurring in both the absence of catalyst (a) and in the absence of base (b), in agreement with the previous optimisation.

4. Determination of Configuration

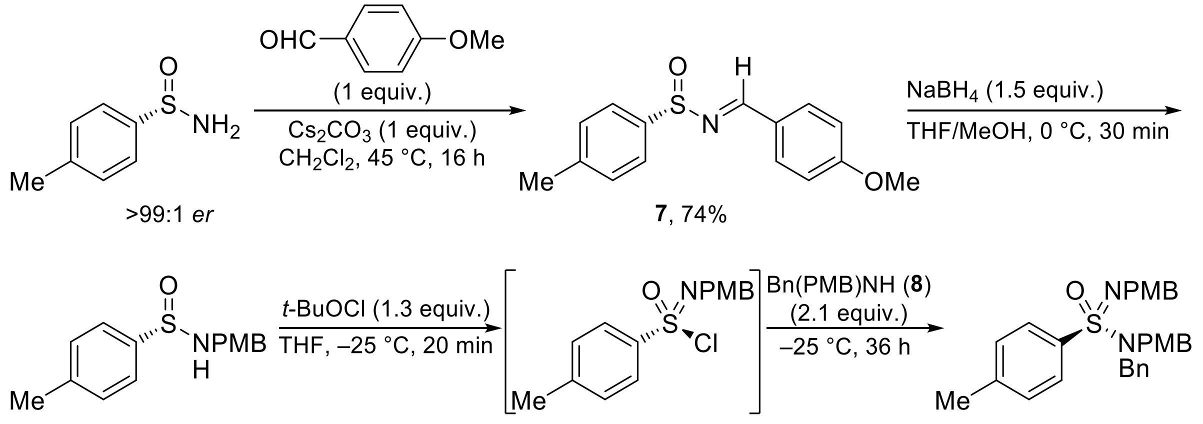

Scheme S2. Reaction sequence used to synthesis an authentic sample of **(*R*)-3b**, using known stereoselective reactions.

The absolute configuration was determined by the synthesis of an authentic sample of **(*R*)-3b**, starting from one of the few commercially available enantiopure sulfinamides (Scheme S2). The first step involved synthesis of the sulfinimine **7**, via condensation with the appropriate aldehyde,[3] followed by a reduction to sulfinamide **1**.[4] This sequence had no effect on configuration at sulfur by comparison to analogous reaction sequences.[5]

Sulfinamide **1** was then chlorinated with *t*-BuOCl to produce the sulfonimidoyl chloride *in situ*, which was subsequently displaced with the appropriate amine (**8**) to yield **3b**, in a stereoselective reaction. Previous literature has shown that the initial chlorination occurs with retention,[6] and then for related substrates the displacement occurs with inversion,[6-7] overall producing the (*R*)-configuration. During the course of these experiments, we noted that it was imperative to maintain cryogenic temperatures for extended periods of time, as when the reactions were warmed to room temperature considerable racemisation of the sulfonimidoyl chloride was observed, providing **(*R*)-3b** with an undesirable enantiomeric ratio (typically 30:70 *er*).

Comparison of the optical rotations of this authentic sample and that of the sample produced via the asymmetric phase-transfer catalysed alkylation, indicated that they were of opposite sign. The difference in configuration was further confirmed by examination of the chiral-phase HPLC data, overall indicating that our procedure provides the (*S*)-configuration.

5. Configuration Stress Test

Table S10. Attempts to racemise (*R*)-3b under various conditions

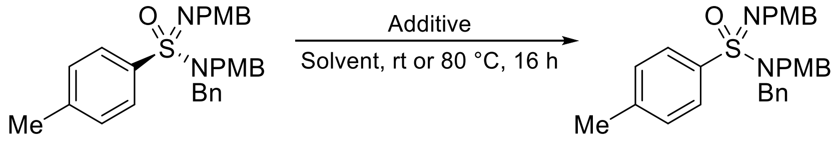

(*R*)-3b **3b**

Entry	Solvent	Temperature (°C)	Additive	*es* (%)
1	Toluene	23	N/A	100
2	Toluene	80	N/A	100
3	Acetonitrile	23	N/A	100
4	Acetonitrile	80	N/A	100
5	Ethanol	23	N/A	100
6	Ethanol	80	N/A	100
7	THF	23	N/A	100
8	THF	80	N/A	100
9	THF	23	**8** (1.1 equiv.)	100
10	THF	80	**8** (1.1 equiv.)	100
11	THF	23	NH_3 (xs.)	100
12	THF	80	NH_3 (xs.)	100

Sulfonimidamide (25 mg), was dissolved in solvent (0.5 mL) with the corresponding additive.

Sulfonimidamide **(*R*)-3b** of known enantioenrichment (37:63 *er*) was subjected to multiple stress tests by placing it in a given solvent at either room temperature or 80 °C in a sealed microwave vial. After a period of 16 h the reactions were cooled to room temperature and the solvent removed in vacuo, the crude mixture was directly dissolved in *i*-PrOH and analysed by chiral-phase HPLC, to obtain the enantiospecificity (*es*).

For this stress test multiple solvents were selected including aprotic non-polar (toluene, entries 1 & 2), aprotic polar (acetonitrile, entries 3 & 4) and protic polar (ethanol, entries 5 & 6). As observed all results showed no racemisation had occurred, resulting in enantiospecificities of 100%. Furthermore, using potentially nucleophilic species (Bn(PMB)NH (**8**) and NH_3 in THF), which could induce racemisation (entries 9-12) showed that the sulfonimidamides were configurationally stable.

6. Self-Disproportionation of Enantiomers (SDE) Evaluation

Table S11. SDEvC test on compound (*R*)-3h

Entry	Sample	Mass (mg)	Peak 1 (% area)	Peak 2 (% area)	*ee* (%)
1	Pure sample before chromatography	119	30.95	69.05	38.1
2	F1 (tubes 22-28)	7	30.36	69.64	39.3
3	F2 (tubes 29-31)	27	30.78	69.22	38.4
4	F3 (tubes 32-34)	39	30.95	69.05	38.1
5	F4 (tubes 35-37)	22	30.85	69.15	38.3
6	F5 (tail, tubes 38-56)	12	30.38	69.62	39.2

To ensure the veracity of reported enantiomeric excess (*ee*) or ratios (*er*), tests were conducted to gauge the magnitude of the self-disproportionation of enantiomers (SDE) phenomenon.[19] To rule out any SDEvS, an exact mass of sulfonimidamide **(*R*)-3h** of known enantioenrichment (31:69 *er*) was first subjected to a temperature of 60 °C and a pressure of 50 mbar for 12 h. No weight loss was observed, rulling out the possibility of self-disproportionation of enantiomers *via* sublimation.

An exact mass (119 mg, entry 1) of sulfonimidamide **(*R*)-3h** of known enantioenrichment (38.1% *ee*, 31:69 *er*) was then subjected to chromatography using the same purification procedure as used routinely and fractionated as depicted in Table S11. The *ee's* of all five fractions were then determined by chiral HPLC (Table S11, entries 2-6) using a Chiralpak IA-3 column (*n*-hexane/*i*-PrOH, 80:20), and the maximum Δ*ee* was calculated to be 1.2% *ee*. The maximum Δ*ee* being under 5% *ee*, we proceeded under the assumption that the influence of the SDEvC on the recorded *er's*/*ee's* is non-significant.

7. Experimental Procedures

7.1. Preparation of Reagents

(*S*)-4-methyl-*N*-(4-methylbenzylidene)benzenesulfinamide (7)

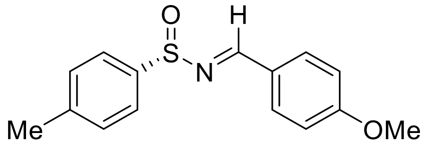

The title compound was prepared according to the literature procedure.[3]

(*S*)-4-methylbenzenesulfinamide (>99:1 *er*) (2.33 g, 15 mmol, 1.0 equiv.), cesium carbonate (4.89 g, 15 mmol, 1.0 equiv.) and 4-methoxybenzaldehyde (1.83 mL, 15 mmol, 1.0 equiv.) were added to a round bottom flask equipped with a reflux condenser, and then CH_2Cl_2 (75 mL) was added. The reaction was then heated to 45 °C and left to stir for 16 h. The reaction mixture was then cooled to room temperature and water (50 mL) was subsequently added. The aqueous layer was further extracted with CH_2Cl_2 × 3 (100 mL). The organic extracts were combined and dried with sodium sulfate, filtered and concentrated in vacuo. Purification by flash column chromatography (Petrol:EtOAc 9:1 to 4:1), afforded the title compound (3.03 g, 74%) as a white solid. The spectroscopic data is in agreement with literature.[8]

MP (Petrol/EtOAc) 131-133 °C

^{1}H NMR (400 MHz, $CDCl_3$) δ_H 8.66 (s, 1H, N*H*C), 7.78 (d, *J* = 9.0 Hz, 2H, 2 × Ar*H*), 7.62 (d, *J* = 8.0 Hz, 2H, 2 × CHAr*H*), 7.28 (d, *J* = 8.0 Hz, 2H, 2 × CHAr*H*), 6.92 (d, *J* = 9.0 Hz, 2H, 2 × Ar*H*), 3.82 (s, 3H, OC*H₃*), 2.37 (s, 3H, ArC*H₃*).

^{13}C NMR (101 MHz, $CDCl_3$) δ_C 163.3, 159.9, 142.3, 141.7, 131.6, 129.9, 127.1, 124.9, 114.4, 55.5, 21.5.

LRMS (ESI$^+$, m/z) $[M+Na]^+$ 296.0.

$[\alpha]_D^{25}$ +33.7° (*c* = 1.0, $CHCl_3$).

(*S*)-4-methyl-*N*-(4-methylbenzyl)benzenesulfinamide (1)

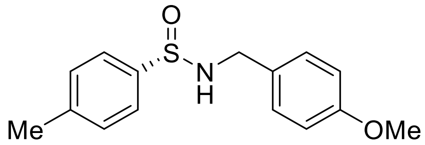

The title compound was prepared according to the modified literature procedure.[4]

Sulfinimine **6** (2.46 g, 9 mmol, 1.0 equiv.) was added to a round bottom flask and dissolved in THF (45 mL) and subsequently cooled to 0 °C. To this solution was added sodium borohydride (0.51 g, 13.5 mmol, 1.5 equiv.) dissolved in MeOH (13.5 mL). The reaction was left to stir for 30 min at 0 °C, then a saturated aqueous solution of sodium bicarbonate (20 mL) was added dropwise, and the reaction extracted with CH_2Cl_2 × 3 (100 mL). The organic extracts were combined and dried with sodium sulfate and concentrated in vacuo affording the title

compound (2.38 g, 96%) as a white solid. No further purification was required. The spectroscopic data is in agreement with literature.[9]

MP (CH_2Cl_2) 70-72 °C

^{1}H NMR (400 MHz, $CDCl_3$) δ_H 7.63 (d, *J* = 8.0 Hz, 2H, 2 × Ar*H*), 7.30 (d, *J* = 8.0 Hz, 2H, 2 × Ar*H*), 7.17 (d, *J* = 8.5 Hz, 2H, 2 × CH_2Ar*H*), 6.83 (d, *J* = 8.5 Hz, 2H, 2 × CH_2Ar*H*), 4.33 (br. dd, *J* = 6.5, 5.5 Hz, 1H, N*H*), 4.16 (dd, *J* = 13.0, 5.5 Hz, 1H, CH_AH_B), 3.81 (dd, *J* = 13.0, 6.5 Hz, 1H, CH_AH_B), 3.77 (s, 3H, OCH_3), 2.40 (s, 3H, ArCH_3).

^{13}C NMR (101 MHz, $CDCl_3$) δ_C 159.2, 141.4, 141.1, 130.0, 129.7, 129.7, 126.1, 114.1, 55.4, 44.1, 21.4.

LRMS (ESI$^+$, m/z) $[M+Na]^+$ 298.0.

er was determined by HPLC using a Chiralpak IA-3 column (*n*-hexane:*i*-PrOH, 85:15); τ_{major} = 11.72 min, τ_{minor} = 15.29 min (>99.5:0.5 *er*).

$[\alpha]_D^{25}$ +11.8° (*c* = 1.0, $CHCl_3$).

Note: To obtain a sample of the racemic material for chiral-phase HPLC analysis an alternative literature procedure was conducted.[5]

Dry *p*-toluenesulfinic acid sodium salt (1.78 g, 10.00 mmol, 1.00 equiv.) and toluene (25 mL) was added to a round bottom flask. The suspension was cooled to 0 °C and oxalyl chloride (0.89 mL, 10.52 mmol, 1.05 equiv.) was added dropwise. The reaction mixture was warmed to room temperature over 1 h to generating *p*-toluenesulfinyl chloride *in situ*. This solution was then added dropwise to a mixture of 4-methoxylbenzylamine (1.57 mL, 12.00 mmol, 1.20 equiv.) and triethylamine (2.10 mL ,15.00 mmol, 1.50 equiv.) in toluene (7 mL) at 0 °C. After stirring for 1 h at 0 °C water (20 mL) and EtOAc (50 mL) were added to the solution, the organic layer was extracted, and the aqueous phase further extracted with EtOAc × 2 (50 mL). The organic extracts were combined and washed with aqueous HCl (0.1 M), dried over magnesium sulfate, filtered and concentrated in vacuo. Puurification by flash column chromatography (Petrol:EtOAc 4:1 to 1:1), afforded the racemate of the title compound (1.80 g, 69%) as an off-white solid.

***N*-benzyl-1-(4-methoxyphenyl)methanamine (8)**

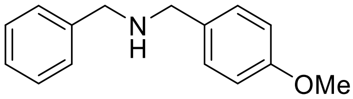

The title compound was prepared according to the literature procedure.[10]

Sodium triacetoxyborohydride (5.26 g, 24.5 mmol, 1.4 equiv.) was added to a round bottom flask and dichloroethane (62.5 mL) was added. To this suspension was added 4-methoxybenzaldehyde (2.23 mL, 17.5 mmol, 1.0 equiv.) and benzylamine (1.94 mL, 17.5 mmol, 1.0 equiv.) at room temperature and the reaction mixture was left to stir for 4 h.

The reaction was quenched by the dropwise addition of a saturated aqueous solution of sodium bicarbonate (30 mL) and the aqueous phase was extracted with CH_2Cl_2 × 3 (100 mL). The organic extracts were combined and dried with sodium sulfate, filtered and concentrated in vacuo. Purification by flash column chromatography (Petrol:EtOAc, 4:1 to 3:7), afforded the title compound (6.60 g, 88%) as a yellow oil. The spectroscopic data is in agreement with literature.[11]

^{1}H NMR (400 MHz, $CDCl_3$) δ_H 7.38 – 7.33 (m, 4H, 4 × Ph*H*), 7.31 – 7.24 (m, 3H, Ph*H*, 2 × *p*-OMeAr*H*), 6.89 (d, *J* = 8.5 Hz, 2H, 2 × *p*-OMeAr*H*), 3.85 – 3.79 (m, 5H, OCH_3, NCH_2), 3.77 (s, 2H, NCH_2), 1.74 (s, 1H, N*H*).

^{13}C NMR (101 MHz, $CDCl_3$) δ_C 158.8, 140.5, 132.5, 129.5, 128.5, 128.3, 127.1, 113.9, 55.4, 53.2, 52.7.

LRMS (ESI^+, m/z) $[M+H]^+$ 228.2.

TrNSO (9)

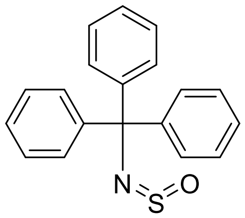

The title compound was prepared according to the literature procedure.[12]

To a round bottom flask was added tritylamine (10.0 g, 38.6 mmol, 1.0 equiv.) which was dissolved in diethyl ether (145 mL). Triethylamine (10.76 mL, 77.1 mmol, 2.0 equiv.) was added and the reaction was cooled to 0 °C, then thionyl chloride (2.80 mL, 38.6 mmol, 1.0 equiv.) was added dropwise. The reaction was stirred at 0 °C for 2 h. Filtration through Celite® and washing with diethyl ether (200 mL), afforded TrNSO (10.3 g, 87%) as a white solid. The spectroscopic data is in agreement with literature.[12]

MP (Et_2O) 93-94 °C

^{1}H NMR (400 MHz, $CDCl_3$) δ_H 7.46 – 7.29 (m, 15H, Ar*H*).

^{13}C NMR (101 MHz, $CDCl_3$) δ_C 144.4, 128.7, 128.3, 127.7, 81.2.

LRMS (ESI^+, m/z) $[M+H]^+$ 306.0 & $[M-NSO]^+$ 243.2.

1-(4-Bromophenyl)-5-(*p*-tolyl)-3-(trifluoromethyl)-1*H*-pyrazole (10)

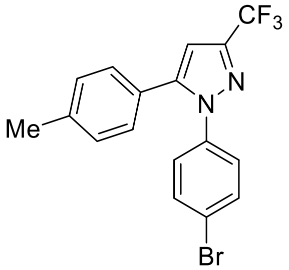

The title compound was prepared according to the literature procedure.[12]

The reaction was conducted under an atmosphere of air. 4,4,4-Trifluoro-1-(*p*-tolyl)butane-1,3-dione (1.30 g, 5.65 mmol, 1.0 equiv.) was added to a 3-necked round bottom flask and dissolved in EtOH (60 mL). 4-Bromophenylhydrazine hydrochloride (1.29 g, 5.76 mmol, 1.02 equiv.) was next added. The reaction mixture was refluxed at 90 °C for 15 h. The solvent was removed in vacuo then diluted with EtOAc (100 mL). The solution was washed with water (50 mL), then the layers were separated, and the aqueous phase was extracted with EtOAc × 3 (100 mL). The combined organic layers were dried over sodium sulfate, filtered and concentrated in vacuo. The crude product was dissolved in MeOH (10 mL) and heated to reflux using a heatgun, then allowed to cool to room temperature. Crystallisation was observed. The solution was cooled to 0 °C. Filtration and washing with cold MeOH (0 °C, 10 mL), afforded the title compound (1.50 g, 70%) as pale orange crystals. The spectroscopic data is in agreement with literature.[12]

^{1}H NMR (400 MHz, $CDCl_3$) δ_H 7.49 (d, J = 9.0 Hz, 2H, 2 × Ar*H*), 7.20 (d, J = 9.0 Hz, 2H, 2 × Ar*H*), 7.16 (d, J = 8.5 Hz, 2H, 2 × Tol*H*), 7.10 (q, J = 8.5 Hz, 2H, 2 × Tol*H*), 6.71 (s, 1H, HetAr*H*), 2.37 (s, 3H, TolCH_3).

^{13}C NMR (101 MHz, $CDCl_3$) δ_C 145.0, 143.6 (q, $^2J_{CF}$ = 38.0 Hz), 139.5, 138.5, 132.4, 129.7, 128.8, 127.0, 126.1, 122.3, 121.3 (q, $^1J_{CF}$ = 269.0 Hz), 105.8 (q, $^3J_{CF}$ = 1.5 Hz), 21.5.

^{19}F NMR (377 MHz, $CDCl_3$) δ_F -62.26.

LRMS (ESI$^+$, m/z) $[M+H]^+$ 381.0 & 383.0.

7.2. Synthesis of an Unsubstituted Sulfonimidamide

[1,1'-Biphenyl]-4-sulfonimidamide (4)

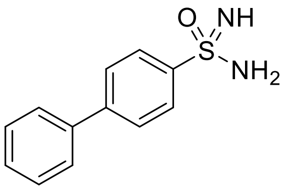

Prepared according to the modified procedure from Davies *et al.*[6] 4-Bromo-1,1'-biphenyl (4.66 g, 20.0 mmol, 1.00 equiv.) was placed in a 3-neck round bottom flask equipped with a

cold trap condenser, then dissolved in THF (100 mL). The solution was cooled to −78 °C with an acetone/dry ice bath and *n*-butyllithium (8.89 mL, 2.25 M in hexanes, 1.00 equiv.) was added slowly dropwise, the reaction mixture was left to stir at −78 °C for 1 h. TrNSO (6.11 g, 20.0 mmol, 1.00 equiv.) was prepared as a solution in THF (8 mL) and added dropwise to the reaction mixture at −78 °C, then left to stir for a further 20 min. The reaction was then warmed to 0 °C with an ice bath and stirred for 10 min before addition of *t*-butyl hypochlorite (2.38 mL, 21.0 mmol, 1.05 equiv.). After this period liquid ammonia was condensed directly into the reaction medium dropwise, by passing gaseous ammonia over the cold trap condenser filled with acetone/dry ice (note: the system was opened to prevent a build-up of pressure). After 15 min the system was left to stir under a nitrogen atmosphere for 16 h. Methanesulfonic acid (13.0 mL, 200.0 mmol, 10.0 equiv.) was added at room temperature and the reaction was stirred vigorously for 15 min before dilution with EtOAc (20 mL). The solution was then washed with saturated ammonium chloride (20 mL) and the aqueous phase extracted with EtOAc (20 mL × 3). The combined organic layers were dried with sodium sulfate, filtered then concentrated under reduced pressure. Purification by flash column chromatography (Petrol:EtOAc:MeOH, 4:1:0 to 0:9:1), afforded the title compound, as a brown solid. The compound was further purified by trituration with acetone, affording the title compound (1.44 g, 31%) as a colourless solid.

MP (Acetone) 119-121 °C.

^{1}H NMR (400 MHz, CD_3OD) δ_H 8.08 (d, *J* = 8.5 Hz, 2H, 2 × Ar*H*), 7.76 (d, *J* = 8.5 Hz, 2H, 2 × Ar*H*), 7.71 – 7.58 (m, 2H, 2 × Ph*H*), 7.53 – 7.40 (m, 2H, 2 × Ph*H*), 7.43 – 7.32 (m, 1H, Ph*H*), (note: NH_2 & N*H* not observed).

^{1}H NMR (400 MHz, $(CD_3)_2SO$) δ_H 7.98 (d, *J* = 8.0 Hz, 2H, 2 × Ar*H*), 7.80 (d, *J* = 8.0 Hz, 2H, 2 × Ar*H*), 7.72 (d, *J* = 7.5 Hz, 2H, 2 × Ph*H*), 7.54 – 7.46 (m, 2H, 2 × Ph*H*), 7.45 – 7.37 (m, 1H, Ph*H*), 5.96 (br. s, 3H, NH_2 & N*H*).

^{13}C NMR (101 MHz, CD_3OD) δ_C 146.0, 144.9, 140.7, 130.1, 129.3, 128.3, 128.2, 128.1.

^{13}C NMR (101 MHz, $(CD_3)_2SO$) δ_C 145.6, 142.6, 139.0, 129.2, 128.3, 127.1, 126.8, 126.7.

LRMS (ESI, *m/z*) $[M+H]^+$ 233.0.

The spectroscopic data is in agreement with literature.[6]

The following HCl salt of the title compound was obtained by dissolving sulfonimidamide **4** (*ca*. 10 mg) in water and adding a few drops of 1 M HCl, which subsequently crystallised allowing analysis by X-ray crystallography.

7.3. Sulfonimidamide Synthesis from a Sulfinamide

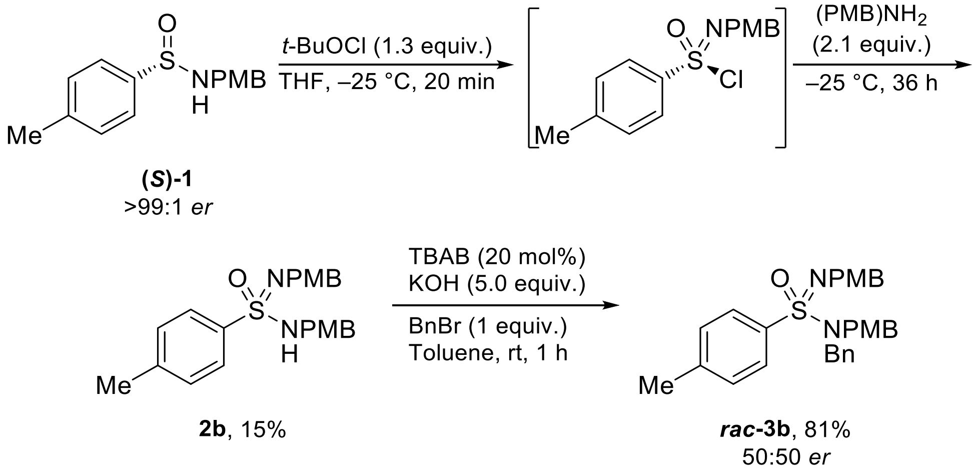

Scheme S3. Reaction sequence for the synthesis of **3b** from enantiopure starting material **(*S*)-1.**

(*S*)-sulfinamide **1** (193 mg, 0.70 mmol, 1.0 equiv.) was dissolved in THF (5.6 mL) and cooled to –25 °C using an immersion cooler. To this solution, was added *t*-butyl hypochlorite (103 µL, 0.91 mmol, 1.3 equiv.) by slow dropwise addition. The reaction was stirred at –25 °C for 20 min and then 4-methoxybenzylamine (0.19 mL, 1.54 mmol, 2.2 equiv.) dissolved in THF (1.4 mL) was added. The reaction mixture was left to stir at –25 °C for 36 h, using the immersion cooler apparatus. The reaction was quenched with water (10 mL) and extracted EtOAc × 3 (25 mL), the combined organic extracts were dried with sodium sulfate and concentrated in vacuo. Purification by flash column chromatography (Petrol:EtOAc, 9:1 to 1:1), afforded the title compound (41 mg, 15%) as a white solid. The spectroscopic data is in agreement with previously synthesised compound **2b**. Optical rotation measurements indicated a racemic mixture due to the small values obtained which are within error.

Under an atmosphere of air, sulfonimidamide **2b** (34.5 mg, 0.08 mmol, 1.0 equiv.), tetrabutylammonium bromide (6.8 mg, 0.02 mmol, 0.25 equiv.), finely ground potassium hydroxide (23.6 mg, 0.4 mmol, 5.0 equiv.) and benzyl bromide (10 µL, 0.08 mmol, 1.0 equiv.) were added to a reaction vial equipped with a stirrer bar. Toluene (0.8 mL) was added and the reaction was vigorously stirred for 1 h at room temperature, after which the reaction mixture was diluted with EtOAc (2 mL) and washed with water (1 mL). The aqueous layer was further extracted with EtOAc × 3 (2 mL), the organic layers were combined and then concentrated in

vacuo. Purification by flash column chromatography (Petrol:EtOAc, 1:0 to 4:1), affording the title compound **3b** (33.3 mg, 81%) as a white solid. The spectroscopic data of **3b** is presented on page S66. Analysis on chiral-phase HPLC indicated that **3b** was racemic.

7.4. Representative Examples of Catalyst Synthesis

N-(4-cyanobenzyl)cinchoninium bromide (CAT-1)

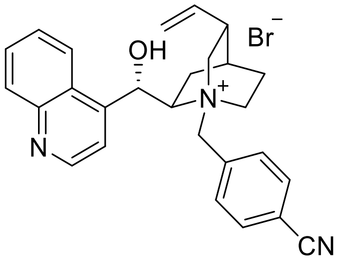

The title compound was prepared according to the modified literature procedure.[13]

The reaction was conducted under an atmosphere of air. To a flask equipped with a stirrer bar and a reflux condenser was added cinchonine (2.94 g, 10.0 mmol, 1.0 equiv.) THF (100 mL) and 4-(bromomethyl)benzonitrile (1.96 g, 10.0 mmol, 1.0 equiv.). The mixture was refluxed at 70 °C for 4 h then cooled to room temperature. Next Et_2O (50 mL) was added, whilst stirring to give a white precipitate. The resulting suspension was filtered and washed with Et_2O (200 mL). The resulting powder was dried under vacuum affording the title compound (4.90 g, 100%) as a white solid.

MP (Et_2O) 234-236 °C (decomp.)

^{1}H NMR (400 MHz, $(CD_3)_2SO$) δ_H 8.98 (d, *J* = 4.5 Hz, 1H, HetAr*H*), 8.43 – 8.35 (m, 1H, Ar*H*), 8.16 – 7.93 (m, 5H, Ar*H*, 4 × *p*-CNAr*H*), 7.88 – 7.79 (m, 2H, HetAr*H*, Ar*H*), 7.75 (ddd, *J* = 8.5, 7.0, 1.5 Hz, 1H, Ar*H*), 6.86 – 6.77 (m, 1H, O*H*), 6.54 – 6.48 (m, 1H, C*H*OH), 5.99 (ddd, *J* = 17.5, 10.5, 7.0 Hz, 1H, $CH_2CH{=}CH_AH_B$), 5.28 (d, *J* = 12.5 Hz, 1H, CH_AH_B*p*-CNAr), 5.25 – 5.18 (m, 2H, $CH_2CH{=}CH_AH_B$, $CH_2CH{=}CH_2$), 5.09 (d, *J* = 12.5 Hz, 1H, CH_AH_B*p*-CNAr), 4.25 (ddd, *J* = 12.0, 8.5, 2.5 Hz, 1H, NCH_AH_B), 4.11 – 4.00 (m, 1H, NCH_AH_B), 3.95 (app. t, *J* = 10.0 Hz, 1H, NC*H*), 3.46 (app. t, *J* = 11.5 Hz, 1H, NCH_AH_B), 2.98 (app. dt, *J* = 12.0, 9.5 Hz, 1H, NCH_AH_B), 2.63 (app. q, *J* = 8.5 Hz, 1H, $CHCH{=}CH_2$), 2.34 – 2.21 (m, 1H, CCH_AH_B), 1.90 – 1.81 (m, 1H, CC*H*), 1.80 – 1.70 (m, 2H, CCH_AH_B, CCH_AH_B), 1.12 – 1.00 (m, 1H, CCH_AH_B).

^{13}C NMR (101 MHz, $(CD_3)_2SO$) δ_C 150.1, 147.6, 144.9, 137.0, 134.8, 133.3, 132.6, 129.8, 129.4, 127.3, 124.3, 123.9, 120.1, 118.3, 117.0, 112.8, 67.4, 64.6, 61.3, 56.0, 54.0, 36.7, 26.2, 23.0, 20.7.

LRMS (ESI$^+$, m/z) [M-Br]$^+$ 410.2.

HRMS (ESI$^+$, m/z) calculated for $(C_{27}H_{28}ON_3)^+$ 410.2227 [M-Br]$^+$, found 410.2224.

IR (thin film, ν_{max}/cm^{-1}) 3186, 2229, 1639, 1592, 1510, 1461, 1422, 1329, 1215, 1164, 1137, 1003, 925, 869, 837, 802, 781, 765, 632.

[α]$_D^{25}$ = +116.8° (c = 1.0, MeOH).

***O*-allyl-*N*-(4-cyanobenzyl)cinchoninium bromide (CAT-13)**

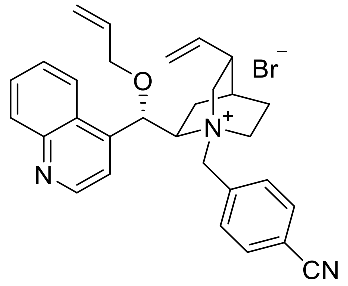

The title compound was prepared according to the modified literature procedure.[13]

The reaction was conducted under an atmosphere of air. To a flask equipped with a stirrer bar was added **CAT-1** (307 mg, 0.625 mmol, 1.0 equiv.) followed by CH_2Cl_2 (3 mL). To the stirred suspension was added allyl bromide (0.16 mL, 1.875 mmol, 3.0 equiv.) and aqueous potassium hydroxide (0.3 mL, 50% wt.) at room temperature. The reaction mixture was subsequently left to stir for 4 h. The reaction was diluted with water (1 mL) and extracted with CH_2Cl_2 × 3 (10 mL). The combined organic extracts were dried over anhydrous sodium sulfate, filtered and concentrated in vacuo. Recrystallisation of the residue from methanol and diethyl ether afforded the title compound (319 mg, 96%) as an off-white solid.

MP (MeOH/Et_2O) 199-201 °C (decomp.)

^{1}H NMR (400 MHz, MeOD) δ_H 9.03 (d, J = 4.5 Hz, 1H, HetAr*H*), 8.55 – 8.45 (m, 1H, Ar*H*), 8.21 – 8.14 (m, 1H, Ar*H*), 8.07 (d, J = 8.0 Hz, 2H, 2 × *p*-CNAr*H*), 7.97 (d, J = 8.0 Hz, 2H, 2 × *p*-CNAr*H*), 7.95 – 7.84 (m, 3H, HetAr*H*, 2 × Ar*H*), 6.52 – 6.46 (m, 1H, C*H*OAllyl), 6.30 (app. ddt, J = 16.5, 10.5, 6.0 Hz, 1H, $CH_2CH=CH_AH_B$), 6.09 (ddd, J = 17.5, 10.5, 7.0 Hz, 1H, $CHCH=CH_AH_B$), 5.54 (app. dq, J = 17.5, 1.5 Hz, 1H, $CH_2CH=CH_AH_B$), 5.47 – 5.29 (m, 4H, $CHCH=CH_AH_B$, $CH_2CH=CH_2$, $CHCH=CH_2$, CH_AH_B*p*-CNAr), 4.91 (d, J = 12.5 Hz, 1H, CH_AH_B*p*-CNAr), 4.42 – 4.29 (m, 3H, CH_AH_BOAllyl, 2 × NCH_AH_B), 4.30 – 4.10 (m, 2H, CH_AH_BOAllyl, NC*H*), 3.72 (ddd, J = 12.0, 10.5, 1.5 Hz, 1H, NCH_AH_B), 3.18 (app. dt, J = 11.5, 9.0 Hz, 1H, NCH_AH_B), 2.81 – 2.70 (m, 1H, $CHCH=CH_2$), 2.69 – 2.51 (m, 1H, CCH_AH_B), 2.11 – 1.99 (m, 1H, CC*H*), 1.99 – 1.82 (m, 2H, CCH_AH_B, CCH_AH_B), 1.33 – 1.19 (m, 1H, CCH_AH_B).

^{13}C NMR (101 MHz, MeOD) δ_C 150.9, 149.1, 142.7, 137.4, 136.1, 136.0, 134.6, 134.0 (2 × C), 131.4, 130.3, 129.5, 126.9, 124.8, 121.3, 119.7, 119.0, 118.1, 115.4, 71.4, 69.2, 64.1, 58.3, 56.5, 38.6, 28.3, 24.4, 22.9.

LRMS (ESI$^+$, m/z) [M-Br]$^+$ 450.2.

HRMS (ESI$^+$, m/z) calculated for $(C_{30}H_{32}ON_3)^+$ 450.2540 [M-Br]$^+$, found 450.2538.

IR (thin film, ν_{max}/cm^{-1}) 3463, 3005 2229, 2162, 1979, 1644, 1612, 1591, 1569, 1508, 1459, 1411, 1381, 1341, 1302, 1264, 1204, 1166, 1134, 1113, 1094, 1058, 1036, 998, 910, 866, 852, 836, 783, 759, 631.

[α]D^{25} = +116.4° (*c* = 1.0, MeOH).

***N*-(4-cyanobenzyl)-*N'*-(2,5-difluorobenzyl)cinchoninium dibromide (CAT-7)**

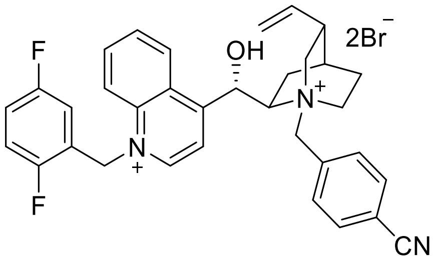

The title compound was prepared according to the modified literature procedure.[14]

A solution of **CAT-1** (2.45 g, 5.0 mmol, 1.0 equiv.) and 2,5-difluorobenzylbromide (1.29 mL, 10.0 mmol, 2.0 equiv.) in IPA (1.2 mL) and DMF (8.3 mL) was stirred at 70 °C for 6 h. The reaction mixture was cooled and EtOAc (20 mL) added dropwise. The resulting slurry was aged for 2 h at room temperature, filtered, rinsed with EtOAc × 2 (100 mL) and dried under vacuum affording the title compound (2.79 g, 80%) as a yellow solid.

MP (EtOAc) 149-151 °C.

^{1}H NMR (400 MHz, MeOD) δ_H 9.68 (dd, *J* = 6.5, 1.5 Hz, 1H, HetAr*H*), 8.94 (ddd, *J* = 7.5, 6.0, 1.5 Hz, 1H, Ar$_F$*H*), 8.61 (d, *J* = 6.5 Hz, 1H, HetAr*H*), 8.61 – 8.54 (m, 1H, Ar*H*), 8.30 (ddd, *J* = 9.0, 7.0, 1.5 Hz, 1H, Ar$_F$*H*), 8.23 (ddd, *J* = 8.0, 7.0, 1.0 Hz, 1H, Ar$_F$*H*), 8.07 (d, *J* = 8.5 Hz, 2H, 2 × *p*-CNAr*H*), 7.93 (d, *J* = 8.5 Hz, 2H, 2 × *p*-CNAr*H*), 7.40 – 7.16 (m, 3H, 3 × Ar*H*), 6.99 – 6.78 (m, 1H, C*H*OH), 6.48 (s, 2H, CH_2Ar$_F$), 6.08 (ddd, *J* = 14.5, 10.5, 7.0 Hz, 1H, CH=CH_AH$_B$), 5.51 (d, *J* = 12.0 Hz, 1H, CH_AH$_B$*p*-CNAr), 5.35 – 5.25 (m, 2H, CH=CH$_A$$H_B$, C*H*=CH$_2$), 5.14 (d, *J* = 12.0 Hz, 1H, CH$_A$$H_B$*p*-CNAr), 4.43 (ddd, *J* = 12.0, 8.5, 2.5 Hz, 1H, NCH_AH$_B$), 4.33 – 4.18 (m, 2H, NC*H*, NCH_AH$_B$), 3.65 (ddd, *J* = 12.0, 10.5, 1.5 Hz, 1H, NCH$_A$$H_B$), 3.13 (app. dt, *J* = 11.0, 9.0 Hz, 1H, NCH$_A$$H_B$), 2.76 – 2.63 (m, 1H, C*H*CH=CH$_2$), 2.48 (dd, *J* = 13.5, 10.5 Hz, 1H, CCH_AH$_B$), 2.08 – 1.97 (m, 1H, CC*H*), 1.97 – 1.84 (m, 2H, CCH_AH$_B$, CCH$_A$$H_B$), 1.37 – 1.21 (m, 1H, CCH$_A$$H_B$), (note: O*H* peak not observed).

^{13}C NMR (101 MHz, MeOD) δ_C 160.4, 160.3 (dd, J_{CF} = 243.5, 1.5 Hz), 158.3 (dd, J_{CF} = 243.5, 2.5 Hz), 150.8, 139.1, 137.4, 137.3, 136.1, 133.9 (2 × C), 132.6, 128.2, 128.1, 122.9 (dd, J_{CF} = 16.5, 8.0 Hz), 122.6, 120.5, 119.4 (dd, J_{CF} = 24.5, 9.0 Hz), 118.9 (dd, J_{CF} = 25.5, 9.0 Hz), 119.0, 118.2, 117.9 (dd, J_{CF} = 26.0, 3.5 Hz), 115.5, 68.9, 67.5, 63.5, 58.4, 56.8, 56.4, 38.8, 28.3, 24.4, 22.4.

^{19}F NMR (377 MHz, MeOD) δ_F -118.19 – -118.45 (m), -122.68 – -122.91 (m).

LRMS (ESI$^+$, m/z) [M-H-2Br]$^+$ 536.2.

HRMS (ESI$^+$, m/z) calculated for $(C_{34}H_{32}ON_3F_2)^+$ 536.2508 [M-H-2Br]$^+$, found 536.2507.

IR (thin film, ν_{max}/cm^{-1}) 3406, 2981, 2231, 1674, 1602, 1535, 1501, 1464, 1391, 1218, 1123, 1093, 1007 941, 864, 813, 757, 729, 617.

[α]D^{25} = +77.4° (*c* = 1.0, MeOH).

7.5. Synthesis of Mono-Substituted Sulfonimidamides

7.5.1. General Procedure A

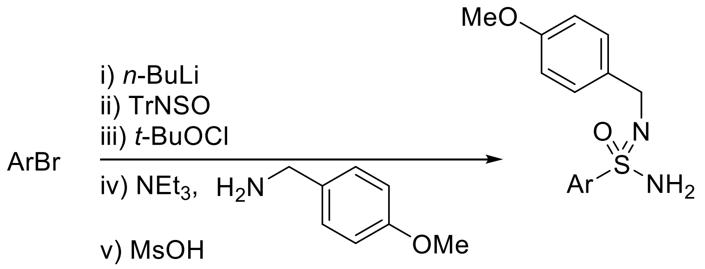

Scheme S4. General reaction sequence for the synthesis of mono-substituted sulfonimidamides from aryl bromides.

Procedure according to the literature procedure.[12]

Aryl bromide (1.00 equiv.) was placed in a reaction vessel, then dissolved in THF (0.20 M). The solution was cooled to −78 °C with an acetone/dry ice bath and *n*-butyllithium (2.25 M in hexanes, 1.00 equiv.) was added slowly dropwise, the reaction mixture was left to stir at −78 °C for 1 h. TrNSO (1.00 equiv.) was prepared as a solution in THF (2.50 M) and added dropwise to the reaction mixture at −78 °C, then left to stir for a further 20 min. The reaction was then warmed to 0 °C with an ice bath and stirred for 10 min before addition of *t*-butyl hypochlorite (1.05 equiv.). After this period triethylamine (1.00 equiv.) and the corresponding amine (1.20 equiv.) were added and the reaction was left to stir for 16 h. Methanesulfonic acid (5.00-15.00 equiv.) was added at room temperature and the reaction was stirred vigorously for 15 min before dilution with CH_2Cl_2. The solution was then washed with a saturated aqueous solution of potassium carbonate and the aqueous phase further extracted with CH_2Cl_2 × 3. The combined organic layers were dried with sodium sulfate, filtered then concentrated in vacuo. The crude material was purified by flash column chromatography.

7.5.2. General Procedure B

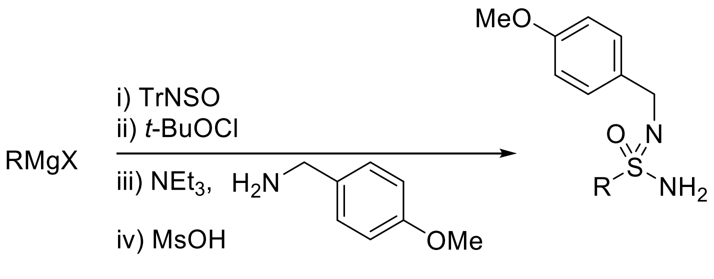

Scheme S5. General reaction sequence for the synthesis of mono-substituted sulfonimidamides from Grignard reagents.

Procedure according to the literature procedure.[12]

TrNSO (1.00 equiv.) was placed in a reaction vessel and dissolved in THF (0.15 M). The solution was cooled to 0 °C with an ice bath and the corresponding organometallic reagent (1.00 equiv.) was added dropwise, the reaction mixture was left to stir at 0 °C for 5 min. *t*-Butyl

hypochlorite (1.05 equiv.) was added and the mixture stirred for 15 min prior to addition of triethylamine (1.00 equiv.) and the corresponding amine (1.20 equiv.). The reaction mixture was stirred at room temperature for 16 h. Methanesulfonic acid (5.00-10.00 equiv.) was added and the reaction stirred vigorously for 15 min at room temperature before dilution with CH_2Cl_2. The solution was then washed with a saturated aqueous potassium carbonate solution, then the layers were separated and the aqueous phase was extracted with CH_2Cl_2 × 3. The combined organic layers were dried over sodium sulfate, filtered and concentrated in vacuo. The crude material was purified by flash column chromatography.

***N'*-(4-methoxybenzyl)-[1,1'-biphenyl]-4-sulfonimidamide (11a)**

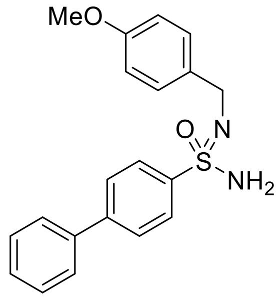

Prepared according to general procedure A, using 4-bromobiphenyl (4.662 g, 20.0 mmol, 1.00 equiv.), *n*-butyllithium (8.0 mL, 2.50 M in hexanes, 1.00 equiv.) TrNSO (6.108 g, 20.0 mmol, 1.00 equiv.), *t*-butyl hypochlorite (2.4 mL, 21.0 mmol, 1.05 equiv.), triethylamine (2.8 mL, 20.0 mmol, 1.00 equiv.), 4-methoxybenzylamine (3.1 mL, 24.0 mmol, 1.20 equiv.) and methanesulfonic acid (13.0 mL, 0.20 mol, 10.0 equiv.). Purification by flash column chromatography (Petrol:EtOAc, 4:1 to 1:4), afforded the title compound (4.029 g, 57%) as a white solid.

MP (Petrol/EtOAc) 102-104 °C.

^{1}H NMR (400 MHz, $CDCl_3$) δ_H 7.92 (d, *J* = 8.5 Hz, 2H, 2 × Ar*H*), 7.58 (d, *J* = 8.5 Hz, 2H, 2 × Ar*H*), 7.57 – 7.46 (m, 2H, 2 × Ph*H*), 7.45 – 7.28 (m, 3H, 3 × Ph*H*), 7.02 (d, *J* = 8.5 Hz, 2H, 2 × *p*-OMeAr*H*), 6.68 (d, *J* = 8.5 Hz, 2H, 2 × *p*-OMeAr*H*), 4.03 (d, *J* = 13.5 Hz, 1H, NCH_AH_B), 3.93 (d, *J* = 13.5 Hz, 1H, NCH_AH_B), 3.64 (s, 3H, OCH_3), (note: NH_2 peak not observed).

^{13}C NMR (101 MHz, $CDCl_3$) δ_C 159.2, 145.1, 139.8, 139.5, 129.3, 129.0, 128.9, 128.4, 127.9, 127.5, 127.3, 114.0, 55.3, 47.3.

LRMS (ESI$^+$, m/z) [M+H]$^+$ 353.1.

HRMS (ESI$^+$, m/z) calculated for $(C_{20}H_{21}O_2N_2S)^+$ 353.1318 [M+H]$^+$, found 353.1315.

IR ($CHCl_3$, v_{max}/cm^{-1}) 3271, 1612, 1513, 1248, 1177, 1134, 1006, 838, 764, 698, 664.

R$_f$ 0.31 (Petrol:EtOAc, 1:1).

***N*'-(4-methoxybenzyl)-4-methylbenzenesulfonimidamide (11b)**

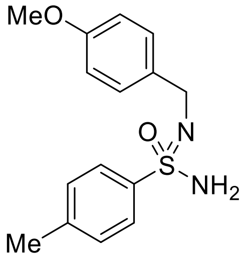

Prepared according to general procedure A using 4-bromotoluene (1.23 mL, 10.0 mmol, 1.00 equiv.), *n*-butyllithium (4.0 mL, 2.50 M in hexanes, 1.00 equiv.) TrNSO (3.054 g, 10.0 mmol, 1.00 equiv.), *t*-butyl hypochlorite (1.2 mL, 10.5 mmol, 1.05 equiv.), triethylamine (1.4 mL, 10.0 mmol, 1.00 equiv.), 4-methoxybenzylamine (1.6 mL, 12.0 mmol, 1.20 equiv.) and methanesulfonic acid (6.5 mL, 0.1 mol, 10.00 equiv.). Purification by flash column chromatography (Pentane:EtOAc, 4:1 to 1:4), afforded the title compound (2.766 g, 95%) as a white solid.

MP (Pentane/EtOAc) 96-98 °C.

^{1}H NMR (400 MHz, $CDCl_3$) δ_H 7.86 (d, *J* = 8.5 Hz, 2H, 2 × Ar*H*), 7.29 (d, *J* = 8.5 Hz, 2H, 2 × Ar*H*), 7.09 (d, *J* = 8.5 Hz, 2H, 2 × *p*-OMeAr*H*), 6.79 (d, *J* = 8.5 Hz, 2H, 2 × *p*-OMeAr*H*), 4.05 (d, *J* = 13.5 Hz, 1H, NCH_AH_B), 3.95 (d, *J* = 13.5 Hz, 1H, NCH_AH_B), 3.77 (s, 3H, OCH_3), 2.43 (s, 3H, $ArCH_3$), (note: NH_2 peak not observed).

^{13}C NMR (101 MHz, $CDCl_3$) δ_C 159.3, 143.1, 138.0, 129.7, 129.3, 129.0, 127.5, 114.2, 55.4, 47.6, 21.6.

LRMS (ESI^+, m/z) $[M+H]^+$ 291.1.

HRMS (ESI^-, m/z) calculated for $(C_{15}H_{17}O_2N_2S)^-$ 289.1016 $[M-H]^-$, found 289.1014.

IR ($CHCl_3$, v_{max}/cm^{-1}) 3009, 1613, 1514, 1456, 1251, 1177, 1099, 1041, 814, 753, 644.

R_f 0.52 (Petrol:EtOAc, 1:4).

***N*'-(4-methoxybenzyl)benzenesulfonimidamide (11c)**

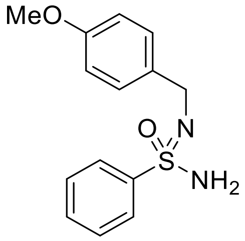

Prepared according to general procedure A, using bromobenzene (1.07 mL, 10.0 mmol, 1.00 equiv.), *n*-butyllithium (4.0 mL, 2.50 M in hexanes, 1.00 equiv.) TrNSO (3.054 g, 10.0 mmol, 1.00 equiv.), *t*-butyl hypochlorite (1.2 mL, 10.5 mmol, 1.05 equiv.), triethylamine (1.4 mL, 10.0 mmol, 1.00 equiv.), 4-methoxybenzylamine (1.6 mL, 12.0 mmol, 1.2 equiv.) and methanesulfonic acid (6.5 mL, 0.1 mol, 10.00 equiv.). Purification by flash column

chromatography (Pentane:EtOAc, 4:1 to 1:4), afforded the title compound (1.166 g, 42%) as a white solid.

MP (Pentane/EtOAc) 64-66 °C.

^{1}H NMR (400 MHz, CDCl$_3$) δ_H 8.00 – 7.95 (m, 2H, 2 × Ph*H*), 7.59 – 7.53 (m, 1H, Ph*H*), 7.52 – 7.46 (m, 2H, 2 × Ph*H*), 7.07 (d, *J* = 8.5 Hz, 2H, 2 × *p*-OMeAr*H*), 6.78 (d, *J* = 8.5 Hz, 2H, 2 × *p*-OMeAr*H*), 4.07 (d, *J* = 13.5 Hz, 1H, NCH_AH$_B$), 3.97 (d, *J* = 13.5 Hz, 1H, NCH$_A$$H_B$), 3.76 (s, 3H, OC$H_3$), (note: N$H_2$ peak not observed).

^{13}C NMR (101 MHz, CDCl$_3$) δ_C 159.3, 141.2, 132.4, 129.3, 129.1, 129.0, 127.4, 114.2, 55.4, 47.6.

LRMS (ESI$^+$, m/z) [M+H]$^+$ 277.1.

HRMS (ESI$^+$, m/z) calculated for $(C_{14}H_{17}O_2N_2S)^+$ 277.1005 [M+H]$^+$, found 277.1005.

IR (CHCl$_3$, ν_{max}/cm^{-1}) 3272, 1612, 1512, 1445, 1244, 1177, 1132, 1029, 822, 749, 689.

R$_f$ 0.46 (Petrol:EtOAc, 1:4).

***N'*-(4-methoxybenzyl)-3-methylbenzenesulfonimidamide (11d)**

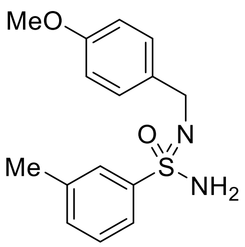

Prepared according to general procedure A, using 3-bromotoluene (1.21 mL, 10.0 mmol, 1.00 equiv.), *n*-butyllithium (4.0 mL, 2.50 M in hexanes, 1.00 equiv.) TrNSO (3.054 g, 10.0 mmol, 1.00 equiv.), *t*-butyl hypochlorite (1.2 mL, 10.5 mmol, 1.05 equiv.), triethylamine (1.4 mL, 10.0 mmol, 1.00 equiv.), 4-methoxybenzylamine (1.6 mL, 12.0 mmol, 1.20 equiv.) and methanesulfonic acid (6.5 mL, 0.1 mol, 10.0 equiv.). Purification by flash column chromatography (Pentane:EtOAc, 4:1 to 1:4), afforded the title compound (879 mg , 30%) as a colourless oil.

^{1}H NMR (400 MHz, CDCl$_3$) δ_H 7.79 – 7.74 (m, 2H, 2 × Ar*H*), 7.40 – 7.31 (m, 2H, 2 × Ar*H*), 7.08 (d, *J* = 8.5 Hz, 2H, 2 × *p*-OMeAr*H*), 6.77 (d, *J* = 8.5 Hz, 2H, 2 × *p*-OMeAr*H*), 4.06 (d, *J* = 13.5 Hz, 1H, NCH_AH$_B$), 3.96 (d, *J* = 13.5 Hz, 1H, NCH$_A$$H_B$), 3.76 (s, 3H, OC$H_3$), 2.40 (s, 3H, ArC$H_3$), (note: N$H_2$ peak not observed).

^{13}C NMR (101 MHz, CDCl$_3$) δ_C 159.2, 140.9, 139.2, 133.1, 129.3, 129.0, 128.9, 127.8, 124.5, 114.1, 55.4, 47.5, 21.5.

LRMS (ESI$^+$, m/z) [M+H]$^+$ 291.1.

HRMS (ESI$^+$, m/z) calculated for $(C_{15}H_{19}O_2N_2S)^+$ 291.1162 [M+H]$^+$, found 291.1162.

IR (CHCl$_3$, ν_{max}/cm^{-1}) 2981, 1612, 1512, 1463, 1246, 1177, 1031, 898, 818, 788, 747, 688.

R$_f$ 0.53 (Petrol:EtOAc, 1:4).

N'-(4-methoxybenzyl)-2-methylbenzenesulfonimidamide (11e)

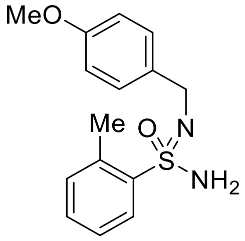

Prepared according to general procedure B, using 2-methylphenylmagnesium chloride (2.10 mL, 0.78 M in THF, 1.00 equiv), TrNSO (500 mg, 1.64 mmol, 1.00 equiv.), *t*-butyl hypochlorite (0.19 mL, 1.72 mmol, 1.05 equiv.), triethylamine (0.23 mL, 1.64 mmol, 1.00 equiv.), 4-methoxybenzylamine (0.26 mL, 1.96 mmol, 1.20 equiv.) and methanesulfonic acid (0.53 mL, 8.20 mmol, 5.00 equiv.). Purification by flash column chromatography (Petrol:EtOAc, 4:1 to 1:4), afforded the title compound (104 mg, 22%) as a colourless oil.

^{1}H NMR (400 MHz, $CDCl_3$) δ_H 8.06 (dd, *J* = 8.0, 1.5 Hz, 1H, Ar*H*), 7.43 (app. td, *J* = 7.5, 1.5 Hz, 1H, Ar*H*), 7.33 – 7.25 (m, 2H, 2 × Ar*H*), 7.08 (d, *J* = 9.0 Hz, 2H, 2 × *p*-OMeAr*H*), 6.78 (d, *J* = 9.0 Hz, 2H, 2 × *p*-OMeAr*H*), 4.10 (d, *J* = 14.0 Hz, 1H, NCH_AH_B), 3.93 (d, *J* = 14.0 Hz, 1H, NCH_AH_B), 3.77 (s, 3H, OCH_3), 2.67 (s, 3H, $ArCH_3$), (note: NH_2 peak not observed).

^{13}C NMR (101 MHz, $CDCl_3$) δ_C 159.2, 139.6, 137.0, 132.8, 132.2, 129.4 (2 × C), 129.1, 126.1, 114.0, 55.3, 47.0, 20.6.

LRMS (ESI^+, m/z) $[M+Na]^+$ 313.2.

HRMS (ESI^+, m/z) calculated for $(C_{15}H_{19}O_2N_2S)^+$ 291.1162 $[M+H]^+$, found 291.1162.

IR ($CHCl_3$, v_{max}/cm^{-1}) 3282, 2933, 2836, 1612, 1512, 1459, 1245, 1179, 1031, 821, 757, 709, 682.

R_f 0.50 (Petrol:EtOAc, 1:4).

4-fluoro-N'-(4-methoxybenzyl)benzenesulfonimidamide (11f)

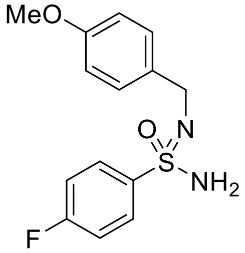

Prepared according to general procedure B, using 4-fluorophenylmagnesium bromide (1.84 mL, 0.89 M in THF, 1.00 equiv), TrNSO (500 mg, 1.64 mmol, 1.00 equiv.), *t*-butyl hypochlorite (0.19 mL, 1.72 mmol, 1.05 equiv.), triethylamine (0.23 mL, 1.64 mmol, 1.00 equiv.), 4-methoxybenzylamine (0.26 mL, 1.96 mmol, 1.20 equiv.) and methanesulfonic acid (0.53 mL, 8.20 mmol, 5.00 equiv.). Purification by flash column chromatography (Petrol:EtOAc, 4:1 to 1:4), afforded the title compound (219 mg, 45%) as a white solid.

MP (Petrol/EtOAc) 76-77 °C.

^{1}H NMR (400 MHz, $CDCl_3$) δ_H 7.87 (dd, *J*, J_{HF} = 9.0, 5.0 Hz, 2H, 2 × Ar*H*), 7.07 – 6.98 (m, 4H, 2 × Ar*H*, 2 × *p*-OMeAr*H*), 6.70 (d, *J* = 8.5 Hz, 2H, 2 × *p*-OMeAr*H*), 4.89 – 4.14 (br. s, 2H, NH_2), 3.98 (d, *J* = 14.0 Hz, 1H, NCH_AH$_B$), 3.88 (d, *J* = 14.0 Hz, 1H, NCH$_A$$H_B$), 3.69 (s, 3H, OC$H_3$).

^{13}C NMR (101 MHz, $CDCl_3$) δ_C 164.6 (d, $^1J_{CF}$ = 253.5 Hz), 158.9, 137.3 (d, $^4J_{CF}$ = 3.0 Hz), 129.9 (d, $^3J_{CF}$ = 9.0 Hz), 129.2, 128.8, 115.8 (d, $^2J_{CF}$ = 22.5 Hz), 113.8, 55.2, 46.9.

^{19}F NMR (377 MHz, $CDCl_3$) δ_F -106.4 – -107.0 (m).

LRMS (ESI$^+$, m/z) $[M+Na]^+$ 317.0.

HRMS (ESI$^+$, m/z) calculated for $(C_{14}H_{16}O_2N_2FS)^+$ 295.0911 $[M+H]^+$, found 295.0911.

IR ($CHCl_3$, v_{max}/cm^{-1}) 3274, 1612, 1589, 1513, 1493, 1248, 1177, 1134, 1111, 1031, 837.

R_f 0.61 (Petrol:EtOAc, 1:4).

***N'*-(4-methoxybenzyl)thiophene-2-sulfonimidamide (11g)**

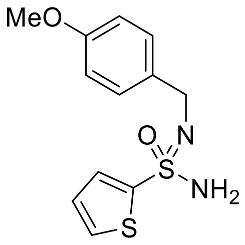

Prepared according to general procedure B, using 2-thienylmagnesium bromide solution (3.86 mL, 0.85 M in THF, 1.00 equiv.), TrNSO (1.000 g, 3.28 mmol, 1.00 equiv.), *t*-butyl hypochlorite (0.39 mL, 3.44 mmol, 1.05 equiv.), triethylamine (0.46 mL, 3.28 mmol, 1.00 equiv.), 4-methoxybenzylamine (0.51 mL, 3.94 mmol, 1.20 equiv.) and methanesulfonic acid (2.1 mL, 32.80 mmol, 10.00 equiv.). Purification by flash column chromatography (Petrol:EtOAc, 4:1 to 1:4), afforded the title compound (211 mg, 23%) as a black oil.

^{1}H NMR (400 MHz, $CDCl_3$) δ_H 7.57 (dd, *J* = 3.5, 1.5 Hz, 1H, HetAr*H*), 7.52 (dd, *J* = 5.0, 1.5 Hz, 1H, HetAr*H*), 7.10 (d, *J* = 9.0 Hz, 2H, 2 × *p*-OMeAr*H*), 7.03 (dd, *J* = 5.0, 3.5 Hz, 1H, HetAr*H*), 6.78 (d, *J* = 9.0 Hz, 2H, 2 × *p*-OMeAr*H*), 4.14 (d, *J* = 13.5 Hz, 1H, NCH_AH$_B$), 4.06 (d, *J* = 13.5 Hz, 1H, NCH$_A$$H_B$), 3.75 (s, 3H, OC$H_3$), (note: N$H_2$ peak not observed).

^{13}C NMR (101 MHz, $CDCl_3$) δ_C 159.2, 143.1, 132.0, 131.9, 129.3, 128.7, 127.5, 114.1, 55.4, 47.6.

LRMS (ESI$^+$, m/z) $[M+H]^+$ 283.2.

HRMS (ESI$^-$, m/z) calculated for $(C_{12}H_{13}N_2O_2S_2)^-$ 281.0424 $[M-H]^-$, found 281.0425.

IR ($CHCl_3$, v_{max}/cm^{-1}) 3277, 2837, 1611, 1512, 1405, 1247, 1177, 1029, 822, 722.

R_f 0.72 (Petrol:EtOAc, 1:4).

***N'*-(4-methoxybenzyl)-4-(5-(p-tolyl)-3-(trifluoromethyl)-1H-pyrazol-1-yl)benzenesulfonimidamide (11h)**

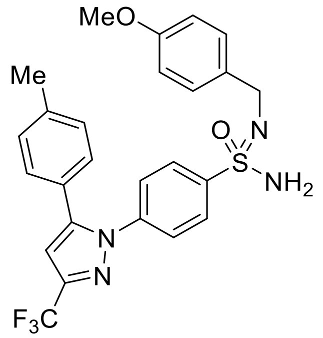

Prepared according to general procedure A, using bromide **10** (1.000 g, 2.62 mmol, 1.0 equiv), *n*-butyllithium (1.22 mL, 2.14 M in hexanes, 1.00 equiv.) TrNSO (800 mg, 2.62 mmol, 1.00 equiv.), *t*-butyl hypochlorite (0.31 mL, 2.75 mmol, 1.05 equiv.), triethylamine (0.37 mL, 2.62 mmol, 1.00 equiv.), 4-methoxybenzylamine (0.41 mL, 3.14 mmol, 1.20 equiv.) and methanesulfonic acid (2.6 mL, 39.3 mmol, 15.0 equiv.). Purification by flash column chromatography (Petrol:EtOAc, 4:1 to 1:4), afforded the title compound (320 mg, 24%) as a white solid.

MP (Petrol/EtOAc) 94-96 °C.

^{1}H NMR (400 MHz, $CDCl_3$) δ_H 7.95 (d, *J* = 8.5 Hz, 2H, 2 × Ar*H*), 7.43 (d, *J* = 8.5 Hz, 2H, 2 × Ar*H*), 7.16 (d, *J* = 8.5 Hz, 2H, 2 × Tol*H*), 7.10 (d, *J* = 8.5 Hz, 2H, 2 × Tol*H*), 7.06 (d, *J* = 8.5 Hz, 2H, 2 × *p*-OMeAr*H*), 6.79 (d, *J* = 8.5 Hz, 2H, 2 × *p*-OMeAr*H*), 6.74 (s, 1H, HetAr*H*), 4.06 (d, *J* = 13.5 Hz, 1H, NCH_AH_B), 3.97 (d, *J* = 13.5 Hz, 1H, NCH_AH_B), 3.76 (s, 3H, OCH_3), 2.37 (s, 3H, $TolCH_3$), (note: NH_2 peak not observed).

^{13}C NMR (101 MHz, $CDCl_3$) δ_C 159.5, 145.4, 144.2 (q, $^2J_{CF}$ = 39.0 Hz), 142.4, 140.6, 139.9, 129.9, 129.6, 129.3, 128.9, 128.5, 127.2 (q, $^1J_{CF}$ = 266.5 Hz), 125.6, 114.3 (2 × C), 106.3 (q, $^3J_{CF}$ = 1.5 Hz), 55.4, 47.7, 21.5.

^{19}F NMR (377 MHz, $CDCl_3$) δ_F -62.4 (s).

LRMS (ESI^+, m/z) $[M+Na]^+$ 523.2.

HRMS (ESI^+, m/z) calculated for $(C_{25}H_{24}O_2N_4F_3S)^+$ 501.1567 $[M+H]^+$, found 501.1554.

IR ($CHCl_3$, ν_{max}/cm^{-1}) 3265, 1613, 1514, 1471, 1374, 1237, 1161, 1134, 1033, 976, 826, 757.

R_f 0.79 (Petrol:EtOAc, 1:4).

***N'*-(4-methoxybenzyl)-2-methylprop-1-ene-1-sulfonimidamide (11i)**

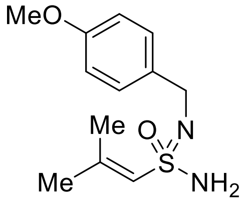

Prepared according to general procedure B, using 2-methyl-1-propenylmagnesium bromide solution (3.28 mL, 0.50 M in THF, 1.0 equiv.), TrNSO (500 mg, 1.64 mmol, 1.00 equiv.), *t*-butyl hypochlorite (0.19 mL, 1.72 mmol, 1.05 equiv.), triethylamine (0.23 mL, 1.64 mmol, 1.00 equiv.), 4-methoxybenzylamine (0.26 mL, 1.96 mmol, 1.20 equiv.) and methanesulfonic acid (0.53 mL, 8.20 mmol, 5.00 equiv.). Purification by flash column chromatography (Petrol:EtOAc, 4:1 to 1:4), afforded the title compound (75 mg, 18%) as a white solid.

MP (Petrol/EtOAc) 72-74 °C.

^{1}H NMR (400 MHz, $CDCl_3$) δ_H 7.21 (d, *J* = 8.5 Hz, 2H, 2 × *p*-OMeAr*H*), 6.84 (d, *J* = 8.5 Hz, 2H, 2 × *p*-OMeAr*H*), 6.09 (app. hept, *J* = 1.5 Hz, 1H, C=C*H*), 4.14 (d, *J* = 14.0 Hz, 1H, NC$H_A$$H_B$), 4.10 (d, *J* = 14.0 Hz, 1H, NC$H_A$$H_B$), 3.78 (s, 3H, OC$H_3$), 2.08 (d, *J* = 1.5 Hz, 3H, CCH_3), 1.84 (d, *J* = 1.5 Hz, 3H, CCH_3), (note: NH_2 peak not observed).

^{13}C NMR (101 MHz, $CDCl_3$) δ_C 159.3, 150.9, 129.6, 129.4, 125.5, 114.2, 55.4, 47.4, 26.8, 19.0.

LRMS (ESI$^+$, m/z) $[M+H]^+$ 255.1.

HRMS (ESI$^+$, m/z) calculated for $(C_{12}H_{19}O_2N_2S)^+$ 255.1161 $[M+H]^+$, found 255.1161.

IR ($CHCl_3$, v_{max}/cm^{-1}) 2912, 2361, 1612, 1513, 1441, 1246, 1176, 1030, 821.

R_f 0.15 (Petrol:EtOAc, 1:4).

***N'*-(4-methoxybenzyl)methanesulfonimidamide (11j)**

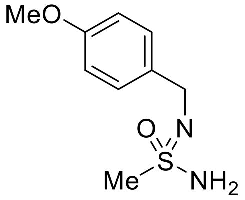

Prepared according to general procedure B, using methyl magnesium bromide solution (1.10 mL, 3.00 M in THF, 1.00 equiv.), TrNSO (1.000 g, 3.28 mmol, 1.00 equiv.), *t*-butyl hypochlorite (0.39 mL, 3.44 mmol, 1.05 equiv.), triethylamine (0.46 mL, 3.28 mmol, 1.00 equiv.), 4-methoxybenzylamine (0.53 mL, 3.94 mmol, 1.20 equiv.) and methanesulfonic acid (1.1 mL, 16.40 mmol, 5.00 equiv.). Purification by flash column chromatography (Petrol:EtOAc, 4:1 to 0:1), afforded the title compound (70 mg, 10%) as a yellow solid.

MP (Petrol/EtOAc) 65-67 °C.

^{1}**H NMR** (400 MHz, $CDCl_3$) δ_H 7.23 (d, J = 8.5 Hz, 2H, 2 × *p*-OMeAr*H*), 6.86 (d, J = 8.5 Hz, 2H, 2 × *p*-OMeAr*H*), 4.19 (app. s, 2H, NCH_AH_B, NCH_AH_B), 3.78 (s, 3H, OCH_3), 2.86 (s, 3H, SCH_3), (note: NH_2 peak not observed).

^{13}**C NMR** (101 MHz, $CDCl_3$) δ_C 159.3, 129.6, 129.3, 114.2, 55.4, 47.5, 42.2.

LRMS (ESI^+, m/z) $[M+H]^+$ 215.4 & $[2M+H]^+$ 429.7.

HRMS (ESI^+, m/z) calculated for $(C_9H_{15}O_2N_2S)^+$ 215.0849 $[M+H]^+$, found 215.0851.

IR ($CHCl_3$, v_{max}/cm^{-1}) 3263, 1612, 1513, 1463, 1246, 1178, 1030, 820.

$\mathbf{R_f}$ 0.12 (EtOAc).

7.6. Synthesis of Di-substituted *N,N'*-Sulfonimidamides

7.6.1. General Procedure C

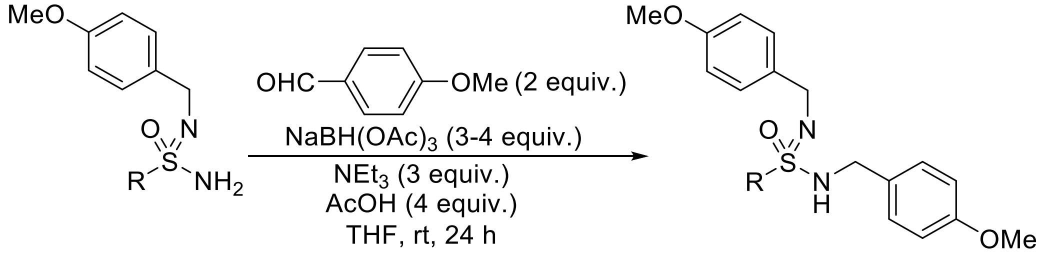

Scheme S6. General reaction sequence for the synthesis of di-substituted *N*,*N'*-sulfonimidamides using a one-pot reductive amination process.

Procedure according to the modified literature procedure.[15]

Sulfonimidamide (1.0 equiv.), 4-methoxybenzaldehyde (2.0 equiv.), and $NaBH(OAc)_3$ (3.0 equiv.) were added to a reaction vessel equipped with a stirrer bar and dissolved in THF (0.4 M) followed by the addition of triethylamine (3.0 equiv.). This solution was treated dropwise with acetic acid (4.0 equiv.) at room temperature and left to stir at this temperature for 24 h, then the reaction mixture was quenched by the dropwise addition of saturated aqueous $NaHCO_3$ solution and extracted with CH_2Cl_2 × 3, the combined organic layers were dried over sodium sulfate and the solvent was removed under reduced pressure. The crude material was purified by flash column chromatography.

Note: If after 24 h the reaction has not gone to full conversion as indicated by TLC, an additional equivalent of $NaBH(OAc)_3$ can be added and left to stir for an addition 6 h.

***N,N'*-bis(4-methoxybenzyl)-[1,1'-biphenyl]-4-sulfonimidamide (2a)**

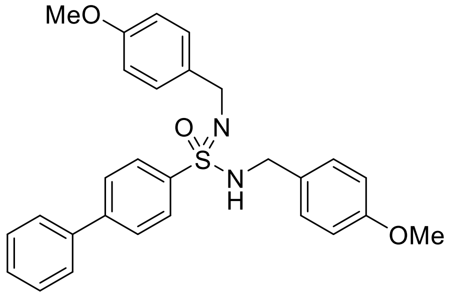

Prepared according to general procedure C, using sulfonimidamide **11a** (5.000 g, 14.2 mmol, 1.0 equiv.), 4-methoxybenzaldehyde (3.46 mL, 28.4 mmol, 2.0 equiv.), NaBH(OAc)$_3$ (9.029 g, 42.6 mmol, 3.0 equiv.), triethylamine (5.94 mL, 42.6 mmol, 3.0 equiv.) and acetic acid (3.25 mL, 56.8 mmol, 4.0 equiv.). Purification by flash column chromatography (Petrol:EtOAc, 4:1 to 1:1) followed by crystallisation (Petrol/Et$_2$O), afforded the title compound (5.342 g, 80%) as a white solid.

MP (Petrol/Et$_2$O) 68-70 °C.

^{1}H NMR (400 MHz, CDCl$_3$) δ_H 8.02 (d, *J* = 8.5 Hz, 2H, 2 × Ar*H*), 7.67 (d, *J* = 8.5 Hz, 2H, 2 × Ar*H*), 7.64 – 7.58 (m, 2H, 2 × Ph*H*), 7.53 – 7.45 (m, 2H, 2 × Ph*H*), 7.45 – 7.36 (m, 1H, Ph*H*), 7.21 (d, *J* = 8.5 Hz, 4H, 4 × *p*-OMeAr*H*), 6.81 (d, *J* = 8.5 Hz, 4H, 4 × *p*-OMeAr*H*), 4.20 (d, *J* = 14.0 Hz, 2H, 2 × NCH_AH$_B$), 4.16 (d, *J* = 14.0 Hz, 2H, 2 × NCH$_A$$H_B$), 3.77 (s, 6H, 2 × OC$H_3$), (note: N*H* peak not observed).

^{13}C NMR (101 MHz, CDCl$_3$) δ_C 158.9, 145.0, 139.7, 138.9, 131.6, 129.1, 129.0, 128.4, 128.2, 127.6, 127.3, 114.0, 55.4, 46.4.

LRMS (ESI$^+$, m/z) [M+H]$^+$ 473.2.

HRMS (ESI$^+$, m/z) calculated for $(C_{28}H_{29}O_3N_2S)^+$ 473.1893 [M+H]$^+$, found 473.1887.

IR (CHCl$_3$, ν_{max}/cm^{-1}) 3263, 3032, 2933, 2835, 1611, 1511, 1245, 1140, 1034, 820, 763, 675, 636.

R$_f$ 0.25 (Petrol:EtOAc, 4:1).

***N,N'*-bis(4-methoxybenzyl)-4-methylbenzenesulfonimidamide (2b)**

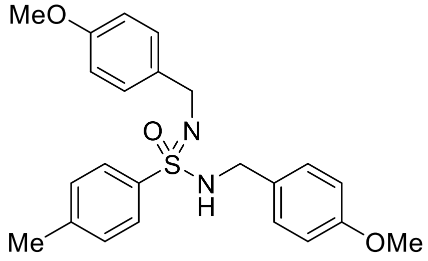

Prepared according to general procedure C, using sulfonimidamide **11b** (581 mg, 2.0 mmol, 1.0 equiv.), 4-methoxybenzaldehyde (0.49 mL, 4.0 mmol, 2.0 equiv.), NaBH(OAc)$_3$ (1.272 g, 6.0 mmol, 3.0 equiv.), triethylamine (0.84 mL, 6.0 mmol, 3.0 equiv.) and acetic acid (0.46 mL,

8.0 mmol, 4.0 equiv.). Purification by flash column chromatography (Pentane:EtOAc, 4:1 to 1:1), afforded the title compound (484 mg, 59%) as a white solid.

MP (Pentane/EtOAc) 74-76 °C.

^{1}H NMR (400 MHz, $CDCl_3$) δ_H 7.87 (d, *J* = 8.5 Hz, 2H, 2 × Ar*H*), 7.28 (d, *J* = 8.5 Hz, 2H, 2 × Ar*H*), 7.19 (d, *J* = 8.5 Hz, 4H, 4 × *p*-OMeAr*H*), 6.81 (d, *J* = 8.5 Hz, 4H, 4 × *p*-OMeAr*H*), 4.15 (d, *J* = 14.0 Hz, 2H, 2 × NCH_AH_B), 4.11 (d, *J* = 14.0 Hz, 2H, 2 × NCH_AH_B), 3.78 (s, 6H, 2 × OCH_3), 2.43 (s, 3H, $ArCH_3$), (note: N*H* peak not observed).

^{13}C NMR (101 MHz, $CDCl_3$) δ_C 158.7, 142.7, 137.1, 131.5, 129.5, 128.9, 127.6, 113.8, 55.3, 46.3, 21.5.

LRMS (ESI^+, m/z) $[M+H]^+$ 411.2 & $[M+Na]^+$ 433.0.

HRMS (ESI^-, m/z) calculated for $(C_{23}H_{25}O_3N_2S)^-$ 409.1580 $[M-H]^-$, found 409.1598.

IR ($CHCl_3$, v_{max}/cm^{-1}) 2835, 1612, 1511, 1463, 1244, 1175, 1140, 1034, 908, 814, 750, 670.

R_f 0.55 (Petrol:EtOAc, 1:1).

***N,N'*-bis(4-methoxybenzyl)benzenesulfonimidamide (2c)**

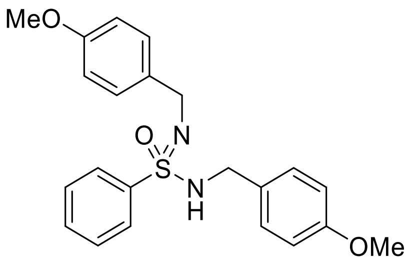

Prepared according to general procedure C, using sulfonimidamide **11c** (553 mg, 2.0 mmol, 1.0 equiv.), 4-methoxybenzaldehyde (0.49 mL, 4.0 mmol, 2.0 equiv.), $NaBH(OAc)_3$ (1.272 g, 6.0 mmol, 3.0 equiv.), triethylamine (0.84 mL, 6.0 mmol, 3.0 equiv.) and acetic acid (0.46 mL, 8.0 mmol, 4.0 equiv.). Purification by flash column chromatography (Pentane:EtOAc, 4:1 to 1:1), afforded the title compound (523 mg, 66%) as a colourless solid.

MP (Pentane/EtOAc) 47-49 °C.

^{1}H NMR (400 MHz, $CDCl_3$) δ_H 8.00 – 7.96 (m, 2H, 2 × Ph*H*), 7.57 – 7.51 (m, 1H, Ph*H*), 7.50 – 7.45 (m, 2H, 2 × Ph*H*), 7.18 (d, *J* = 8.5 Hz, 4H, 4 × *p*-OMeAr*H*), 6.80 (d, *J* = 8.5 Hz, 4H, 4 × *p*-OMeAr*H*), 4.16 (d, *J* = 14.0 Hz, 2H, 2 × NCH_AH_B), 4.12 (d, *J* = 14.0 Hz, 2H, 2 × NCH_AH_B), 3.77 (s, 6H, 2 × OCH_3), (note: N*H* peak not observed).

^{13}C NMR (101 MHz, $CDCl_3$) δ_C 158.8, 140.3, 132.1, 131.5, 129.0, 127.6, 113.9, 55.4, 46.4.

LRMS (ESI^+, m/z) $[M+Na]^+$ 419.2.

HRMS (ESI^+, m/z) calculated for $(C_{22}H_{25}O_3N_2S)^+$ 397.1580 $[M+H]^+$, found 398.1579.

IR ($CHCl_3$, v_{max}/cm^{-1}) 2836, 1612, 1512, 1445, 1246, 1175, 1142, 1034, 820, 750, 689.

R_f 0.48 (Petrol:EtOAc, 3:2).

***N,N'*-bis(4-methoxybenzyl)-3-methylbenzenesulfonimidamide (2d)**

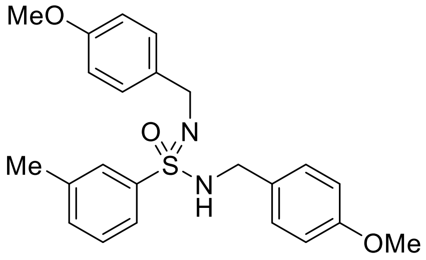

Prepared according to general procedure C, using sulfonimidamide **11d** (581 mg, 2.0 mmol, 1.0 equiv.), 4-methoxybenzaldehyde (0.49 mL, 4.0 mmol, 2.0 equiv.), $NaBH(OAc)_3$ (1.272 g, 6.0 mmol, 3.0 equiv.), triethylamine (0.84 mL, 6.0 mmol, 3.0 equiv.) and acetic acid (0.46 mL, 8.0 mmol, 4.0 equiv.). Purification by flash column chromatography (Pentane:EtOAc, 4:1 to 1:1), afforded the title compound (558 mg, 68%) as a colourless oil.

^{1}H NMR (400 MHz, $CDCl_3$) δ_H 7.82 – 7.76 (m, 2H, 2 × Ar*H*), 7.41 – 7.32 (m, 2H, 2 × Ar*H*), 7.18 (d, *J* = 8.5 Hz, 4H, 4 × *p*-OMeAr*H*), 6.81 (d, *J* = 8.5 Hz, 4H, 4 × *p*-OMeAr*H*), 4.16 (d, *J* = 14.0 Hz, 2H, 2 × NCH_AH_B), 4.12 (d, *J* = 14.0 Hz, 2H, 2 × NCH_AH_B), 3.77 (s, 6H, 2 × OCH_3), 2.40 (s, 3H, $ArCH_3$), (note: N*H* peak not observed).

^{13}C NMR (101 MHz, $CDCl_3$) δ_C 158.8, 140.0, 139.1, 132.9, 131.6, 129.0, 128.8, 128.0, 124.7, 113.9, 55.4, 46.4, 21.5.

LRMS (ESI^+, m/z) $[M+H]^+$ 411.2 & $[M+Na]^+$ 433.2.

HRMS (ESI^+, m/z) calculated for $(C_{23}H_{27}O_3N_2S)^+$ 411.1737 $[M+H]^+$, found 411.1735.

IR ($CHCl_3$, v_{max}/cm^{-1}) 2835, 1612, 1511, 1463, 1245, 1175, 1139, 1034, 819, 748, 688.

R_f 0.54 (Petrol:EtOAc, 1:1).

***N,N'*-bis(4-methoxybenzyl)-2-methylbenzenesulfonimidamide (2e)**

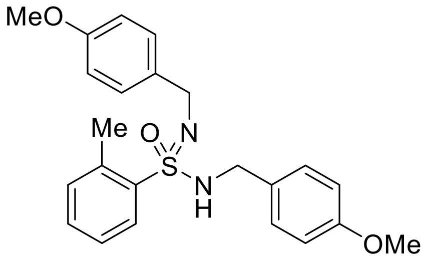

Prepared according to general procedure C, using sulfonimidamide **11e** (88 mg, 0.30 mmol, 1.0 equiv.), 4-methoxybenzaldehyde (73 µL, 0.60 mmol, 2.0 equiv.), $NaBH(OAc)_3$ (191 mg, 0.90 mmol, 3.0 equiv.), triethylamine (0.13 mL, 0.90 mmol, 3.0 equiv.) and acetic acid (0.07 mL, 1.20 mmol, 4.0 equiv.). Purification by flash column chromatography (Petrol:EtOAc, 4:1 to 1:1), afforded the title compound (70 mg, 56%) as a white solid.

MP (Petrol/EtOAc) 51-53 °C.

^{1}H NMR (400 MHz, $CDCl_3$) δ_H 8.09 (dd, *J* = 8.0, 1.5 Hz, 1H, Ar*H*), 7.43 (app. td, *J* = 7.5, 1.5 Hz, 1H, Ar*H*), 7.34 – 7.25 (m, 2H, 2 × Ar*H*), 7.18 (d, *J* = 8.5 Hz, 4H, 4 × *p*-OMeAr*H*), 6.81 (d,

J = 8.5 Hz, 4H, 4 × *p*-OMeAr*H*), 4.16 (d, J = 14.0 Hz, 2H, 2 × NCH_AH$_B$), 4.11 (d, J = 14.0 Hz, 2H, 2 × NCH$_A$$H_B$), 3.79 (s, 6H, 2 × OC$H_3$), 2.63 (s, 3H, ArC$H_3$), (note: N*H* peak not observed).
^{13}C NMR (101 MHz, $CDCl_3$) δ_C 158.7, 138.2, 137.3, 132.7, 132.1, 131.6, 129.9, 129.1, 126.1, 113.9, 55.4, 46.5, 20.6.
LRMS (ESI$^+$, m/z) [M+Na]$^+$ 433.2.
HRMS (ESI$^+$, m/z) calculated for $(C_{23}H_{27}O_3N_2S)^+$ 411.1748 [M+H]$^+$, found 411.1751.
IR ($CHCl_3$, v_{max}/cm^{-1}) 2931, 2835, 1612, 1586, 1512, 1462, 1245, 1175, 1146, 1063, 1034, 818, 755, 709.
R$_f$ 0.61 (Petrol:EtOAc, 1:1).

4-fluoro-*N,N'*-bis(4-methoxybenzyl)benzenesulfonimidamide (2f)

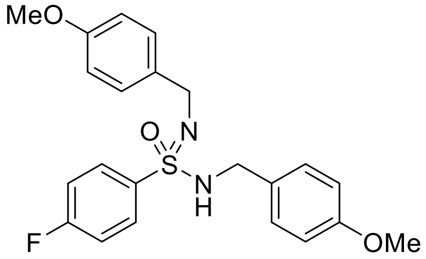

Prepared according to general procedure C, using sulfonimidamide **11f** (112 mg, 0.38 mmol, 1.0 equiv.), 4-methoxybenzaldehyde (93 µL, 0.76 mmol, 2.0 equiv.), NaBH(OAc)$_3$ (242 mg, 1.14 mmol, 3.0 equiv.), triethylamine (0.16 mL, 1.14 mmol, 3.0 equiv.) and acetic acid (0.09 mL, 1.52 mmol, 4.0 equiv.). Purification by flash column chromatography (Petrol:EtOAc, 4:1 to 1:1) followed by crystallisation (Petrol/Et_2O), afforded the title compound (87 mg, 55%) as a white solid.
MP (Petrol/Et_2O) 78-80 °C.
^{1}H NMR (400 MHz, $CDCl_3$) δ_H 7.96 (dd, J, J_{HF} = 8.5, 5.0 Hz, 2H, 2 × Ar*H*), 7.16 (d, J = 8.5 Hz, 4H, 4 × *p*-OMeAr*H*), 7.11 (app. t, J, J_{HF} = 8.5 Hz, 2H, 2 × Ar*H*), 6.81 (d, J = 8.5 Hz, 4H, 4 × *p*-OMeAr*H*), 4.14 (d, J = 14.0 Hz, 2H, 2 × NCH_AH$_B$), 4.10 (d, J = 14.0 Hz, 2H, 2 × NCH$_A$$H_B$), 3.77 (s, 6H, 2 × OC$H_3$), (note: N*H* peak not observed).
^{13}C NMR (101 MHz, $CDCl_3$) δ_C 164.9 (d, $^1J_{CF}$ = 253.5 Hz), 158.9, 136.4 (d, $^4J_{CF}$ = 3.0 Hz), 131.3, 130.3 (d, $^3J_{CF}$ = 9.0 Hz), 129.0, 116.0 (d, $^2J_{CF}$ = 22.4 Hz), 114.0, 55.4, 46.4.
^{19}F NMR (377 MHz, $CDCl_3$) δ_F -106.66 – -107.02 (m).
LRMS (ESI$^+$, m/z) [M+Na]$^+$ 437.2.
HRMS (ESI$^+$, m/z) calculated for $(C_{22}H_{24}O_3N_2FS)^+$ 415.1486 [M+H]$^+$, found 415.1480.
IR ($CHCl_3$, v_{max}/cm^{-1}) 2981, 1612, 1588, 1512, 1492, 1463, 1246, 1175, 1141, 1034, 836, 751.
R$_f$ 0.65 (Petrol:EtOAc, 1:1).

***N,N'*-bis(4-methoxybenzyl)thiophene-2-sulfonimidamide (2g)**

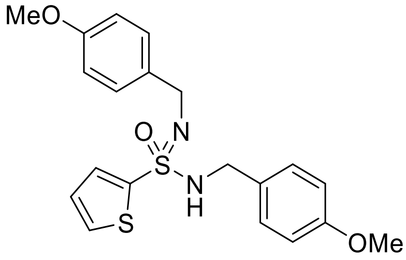

Prepared according to general procedure C, using sulfonimidamide **11g** (113 mg, 0.40 mmol, 1.0 equiv.), 4-methoxybenzaldehyde (98 µL, 0.80 mmol, 2.0 equiv.), $NaBH(OAc)_3$ (254 mg, 1.20 mmol, 3.0 equiv.), triethylamine (0.17 mL, 1.20 mmol, 3.0 equiv.) and acetic acid (0.09 mL, 1.60 mmol, 4.0 equiv.). Purification by flash column chromatography (Pentane:EtOAc, 4:1 to 1:1), afforded the title compound (56 mg, 35%) as a yellow oil.

^{1}H NMR (400 MHz, $CDCl_3$) δ_H 7.57 (dd, *J* = 3.5, 1.5 Hz, 1H, HetAr*H*), 7.54 (dd, *J* = 5.0, 1.5 Hz, 1H, HetAr*H*), 7.19 (d, *J* = 8.5 Hz, 4H, 4 × *p*-OMeAr*H*), 7.07 (dd, *J* = 5.0, 3.5 Hz, 1H, HetAr*H*), 6.82 (d, *J* = 8.5 Hz, 4H, 4 × *p*-OMeAr*H*), 4.19 (app. s, 4H, 2 × NCH_AH_B, 2 × NCH_AH_B), 3.78 (s, 6H, 2 × OCH_3), (note: N*H* peak not observed).

^{13}C NMR (101 MHz, $CDCl_3$) δ_C 158.9, 142.0, 132.0, 131.7, 131.2, 129.0, 127.6, 114.0, 55.4, 46.7.

LRMS (ESI$^+$, m/z) $[M+Na]^+$ 403.1.

HRMS (ESI$^+$, m/z) calculated for $(C_{20}H_{22}O_3N_2S_2Na)^+$ 403.1145 $[M+Na]^+$, found 403.1146.

IR ($CHCl_3$, ν_{max}/cm^{-1}) 3262, 2836, 1612, 1512, 1463, 1246, 1176, 1143, 1033, 819, 722.

R_f 0.46 (Petrol:EtOAc, 1:1).

***N,N'*-bis(4-methoxybenzyl)-4-(5-(p-tolyl)-3-(trifluoromethyl)-1H-pyrazol-1-yl)benzenesulfonimidamide (2h)**

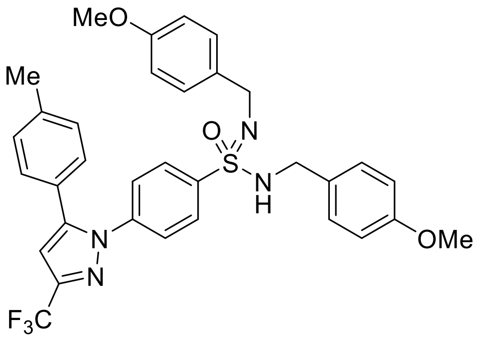

Prepared according to general procedure C, using sulfonimidamide **11h** (200 mg, 0.40 mmol, 1.0 equiv.), 4-methoxybenzaldehyde (98 µL, 0.80 mmol, 2.0 equiv.), $NaBH(OAc)_3$ (254 mg, 1.20 mmol, 3.0 equiv.), triethylamine (0.17 mL, 1.20 mmol, 3.0 equiv.) and acetic acid (0.09 mL, 1.60 mmol, 4.0 equiv.). Purification by flash column chromatography (Pentane:EtOAc, 4:1 to 1:1), afforded the title compound (127 mg, 51%) as a colourless oil.

^{1}H NMR (400 MHz, $CDCl_3$) δ_H 7.94 (d, J = 8.5 Hz, 2H, 2 × ArH), 7.41 (d, J = 8.5 Hz, 2H, 2 × ArH), 7.21 – 7.13 (m, 6H, 2 × Tol$H$, 4 × p-OMeArH), 7.10 (d, J = 8.0 Hz, 2H, 2 × Tol$H$), 6.81 (d, J = 8.5 Hz, 4H, 4 × p-OMeArH), 6.74 (s, 1H, HetArH), 4.16 (d, J = 14.0 Hz, 2H, 2 × NCH_AH$_B$), 4.10 (d, J = 14.0 Hz, 2H, 2 × NCH$_A$$H_B$), 3.77 (s, 6H, 2 × OC$H_3$), 2.37 (s, 3H, TolC$H_3$), (note: N$H$ peak not observed).
^{13}C NMR (101 MHz, $CDCl_3$) δ_C 159.0, 145.3, 144.1 (q, $^2J_{CF}$ = 38.5 Hz), 142.1, 139.9, 139.8, 131.1, 129.8, 129.0, 128.8, 128.6, 125.9, 125.5, 121.3 (q, $^1J_{CF}$ = 269.0 Hz), 114.0, 106.3 (q, $^3J_{CF}$ = 2.0 Hz), 55.4, 46.4, 21.4.
^{19}F NMR (377 MHz, $CDCl_3$) δ_F -62.36.
LRMS (ESI$^+$, m/z) [M+H]$^+$ 621.2.
HRMS (ESI$^+$, m/z) calculated for $(C_{33}H_{32}O_3N_4F_3S)^+$ 621.2142 [M+H]$^+$, found 621.2141.
IR ($CHCl_3$, v_{max}/cm^{-1}) 2837, 1612, 1512, 1471, 1374, 1238, 1135, 1035, 976, 812, 761, 640.
R$_f$ 0.74 (Petrol:EtOAc, 1:1).

***N,N'*-bis(4-methoxybenzyl)-2-methylprop-1-ene-1-sulfonimidamide (2i)**

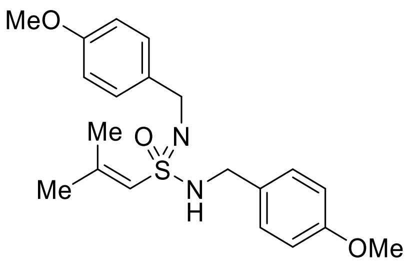

Prepared according to general procedure C, using sulfonimidamide **11i** (64 mg, 0.25 mmol, 1.0 equiv.), 4-methoxybenzaldehyde (61 µL, 0.50 mmol, 2.0 equiv.), $NaBH(OAc)_3$ (159 mg, 0.75 mmol, 3.0 equiv.), triethylamine (0.10 mL, 0.75 mmol, 3.0 equiv.) and acetic acid (0.06 mL, 1.00 mmol, 4.0 equiv.). Purification by flash column chromatography (Pentane:EtOAc, 1:0 to 1:1), afforded the title compound (48 mg, 51%) as a white solid.
MP (Pentane/EtOAc) 67-69 °C.
^{1}H NMR (400 MHz, $CDCl_3$) δ_H 7.24 (d, J = 8.5 Hz, 4H, 4 × p-OMeArH), 6.84 (d, J = 8.5 Hz, 4H, 4 × p-OMeArH), 6.11 (app. hept, J = 1.5 Hz, 1H, C=CH), 4.18 (d, J = 14.0 Hz, 2H, 2 × NCH_AH$_B$), 4.09 (d, J = 14.0 Hz, 2H, 2 × NCH$_A$$H_B$), 3.78 (s, 6H, 2 × OC$H_3$), 2.08 (d, J = 1.5 Hz, 3H, CCH_3), 1.84 (d, J = 1.5 Hz, 3H, CCH_3), (note: NH peak not observed).
^{13}C NMR (101 MHz, $CDCl_3$) δ_C 155.9, 150.6, 131.8, 129.2, 125.3, 114.0, 55.4, 46.2, 26.7, 19.1.
LRMS (ESI$^+$, m/z) [M+Na]$^+$ 397.2.
HRMS (ESI$^+$, m/z) calculated for $(C_{20}H_{27}O_3N_2S)^+$ 375.1737 [M+H]$^+$, found 375.1736.
IR ($CHCl_3$, v_{max}/cm^{-1}) 2836, 1612, 1511, 1441, 1245, 1174, 1128, 1034, 878, 819, 618.
R$_f$ 0.20 (Petrol:EtOAc, 4:1).

***N,N'*-bis(4-methoxybenzyl)methanesulfonimidamide (2j)**

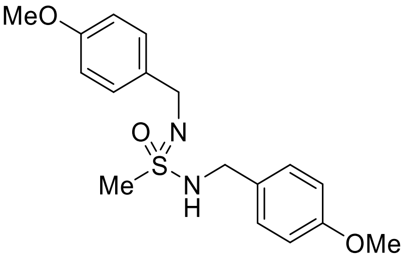

Prepared according to general procedure C, using sulfonimidamide **11j** (54 mg, 0.25 mmol, 1.0 equiv.), 4-methoxybenzaldehyde (61 µL, 0.50 mmol, 2.0 equiv.), $NaBH(OAc)_3$ (159 mg, 0.75 mmol, 3.0 equiv.), triethylamine (0.10 mL, 0.75 mmol, 3.0 equiv.) and acetic acid (0.06 mL, 1.00 mmol, 4.0 equiv.). Purification by flash column chromatography (Pentane:EtOAc, 1:0 to 1:4), afforded the title compound (58 mg, 69%) as a yellow solid.

MP (Pentane/EtOAc) 74-76 °C.

^{1}H NMR (400 MHz, $CDCl_3$) δ_H 7.17 (d, *J* = 8.5 Hz, 4H, 4 × *p*-OMeAr*H*), 6.78 (d, *J* = 8.5 Hz, 4H, 4 × *p*-OMeAr*H*), 4.13 (d, *J* = 14.0 Hz, 2H, 2 × NCH_AH_B), 4.04 (d, *J* = 14.0 Hz, 2H, 2 × NCH_AH_B), 3.72 (s, 6H, 2 × OCH_3), 2.84 (s, 3H, SCH_3), (note: N*H* peak not observed).

^{13}C NMR (101 MHz, $CDCl_3$) δ_C 159.6, 131.7, 129.1, 114.1, 55.4, 46.4, 42.6.

LRMS (ESI$^+$, m/z) $[M+H]^+$ 335.1.

HRMS (ESI$^+$, m/z) calculated for $(C_{17}H_{23}O_3N_2S)^+$ 335.1424 $[M+H]^+$, found 335.1422.

IR ($CHCl_3$, ν_{max}/cm^{-1}) 2836, 1612, 1512, 1463, 1246, 1176, 1135, 1033, 819.

R_f 0.21 (Petrol:EtOAc, 1:4).

7.7. Enantioselective Alkylation of Sulfonimidamides

7.7.1. General Procedure D

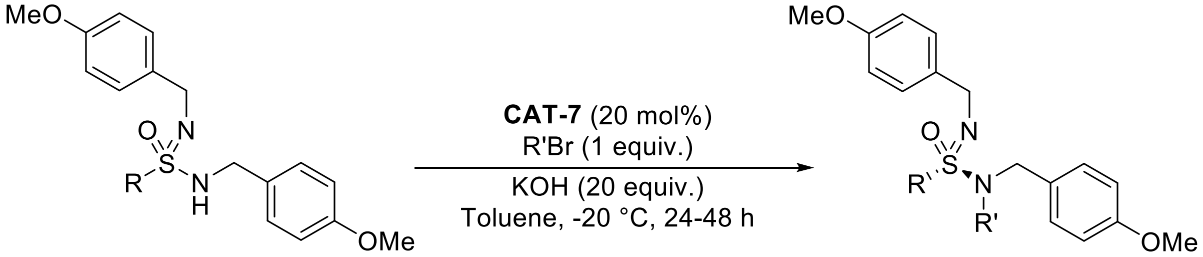

Scheme S7. General reaction for the asymmetric phase-transfer catalysed alkylation of sulfonimidamides.

Under an atmosphere of air, sulfonimidamide (0.05 mmol, 1.0 equiv.), phase-transfer catalyst **CAT-7** (6.2 mg, 0.01 mmol, 0.2 equiv.), finely ground potassium hydroxide (56 mg, 1.00 mmol, 20.0 equiv.) and electrophile (0.05 mmol, 1.0 equiv.) were added to a screw-cap vial equipped with a stirrer bar. Toluene (1.0 mL, 0.05 M) was added to the vial and was cooled to −20 °C in an Easymax 102 Advanced Thermostat system then stirred at 1000 rpm for 24-48 h, after

which the reaction mixture was diluted with EtOAc and washed with water. The aqueous layer was further extracted with EtOAc × 3, the organic layers were combined, then the solvent was removed under reduced pressure and the crude material was purified by flash column chromatography.

Note: To obtain samples of the racemic material for chiral-phase HPLC analysis the reaction was conducted with the alterations as below:

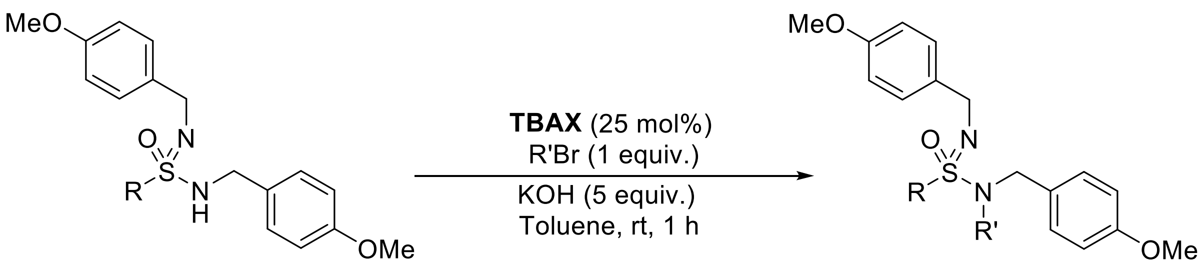

Scheme S8. General reaction for the phase-transfer catalysed alkylation of sulfonimidamides with an achiral catalyst.

Under an atmosphere of air, sulfonimidamide (1.0 equiv.), tetrabutylammonium bromide or tetrabutylammonium hydrogensulfate (0.25 equiv.), finely ground potassium hydroxide (5.0 equiv.) and electrophile (1.0 equiv.) were added to a reaction vial equipped with a stirrer bar. Toluene (0.1 M) was added and the reaction was vigorously stirred for 1 h at room temperature, after which the reaction mixture was diluted with EtOAc and washed with water. The aqueous layer was further extracted with EtOAc × 3, the organic layers were combined, then the solvent was removed under reduced pressure and the crude material was purified by flash column chromatography.

(*S*)-*N*-benzyl-*N*,*N'*-bis(4-methoxybenzyl)-[1,1'-biphenyl]-4-sulfonimidamide (3aa)

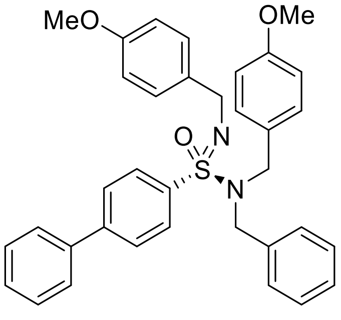

Prepared according to general procedure D, using sulfonimidamide **2a** (23.6 mg, 0.05 mmol, 1.0 equiv.) and benzyl bromide (6 µL, 0.05 mmol, 1.0 equiv.). Purification by flash column chromatography (Pentane:EtOAc, 1:0 to 4:1), afforded the title compound (22.5 mg, 80%) as a colourless oil.

^{1}H NMR (400 MHz, $CDCl_3$) δ_H 8.07 (d, J = 8.5 Hz, 2H, 2 × ArH), 7.69 (d, J = 8.5 Hz, 2H, 2 × ArH), 7.55 – 7.46 (m, 2H, 2 × PhH), 7.55 – 7.47 (m, 2H, 2 × PhH), 7.46 – 7.40 (m, 1H, PhH), 7.38 (d, J = 8.5 Hz, 2H, 2 × *p*-OMeArH), 7.26 – 7.17 (m, 3H, 3 × CH_2PhH), 7.09 – 6.98 (m, 2H, 2 × CH_2PhH), 6.93 (d, J = 8.5 Hz, 2H, 2 × *p*-OMeArH), 6.87 (d, J = 8.5 Hz, 2H, 2 × *p*-OMeArH), 6.73 (d, J = 8.5 Hz, 2H, 2 × *p*-OMeArH), 4.57 (d, J = 15.0 Hz, 1H, NC$H_A$$H_B$), 4.52 (d, J = 14.5 Hz, 1H, NC$H_A$$H_B$), 4.50 (d, J = 14.5 Hz, 1H, NC$H_A$$H_B$), 4.15 (d, J = 14.5 Hz, 1H, NC$H_A$$H_B$), 4.14 (d, J = 15.0 Hz, 1H, NC$H_A$$H_B$), 4.10 (d, J = 14.5 Hz, 1H, NC$H_A$$H_B$), 3.80 (s, 3H, OC$H_3$), 3.76 (s, 3H, OC$H_3$).

^{13}C NMR (101 MHz, $CDCl_3$) δ_C 159.1, 158.4, 144.8, 139.9, 139.8, 136.8, 133.7, 130.3, 128.1, 128.8, 128.7, 128.6, 128.4 (2 × C), 128.0, 127.6, 127.5, 127.4, 113.8, 113.8, 55.4 (2 × C), 51.0, 50.6, 45.3.

HRMS (ESI^+, m/z) calculated for $(C_{35}H_{35}O_3N_2S)^+$ 563.2363 $[M+H]^+$, found 563.2361.

IR ($CHCl_3$, ν_{max}/cm^{-1}) 3030, 2909, 2835, 2361, 1611, 1511, 1245, 1145, 1007, 894, 817, 763, 728, 697, 671.

er was determined by HPLC using a Chiralpak IA-3 column (*n*-hexane:*i*-PrOH, 70:30); τ_{major} = 19.52 min, τ_{minor} = 37.62 min (90.5:9.5 *er*).

$[\alpha]_D^{25}$ −1.1° (*c* = 0.75, $CHCl_3$).

R_f 0.38 (Petrol:EtOAc, 4:1).

(*S*)-*N*-(4-fluorobenzyl)-*N*,*N'*-bis(4-methoxybenzyl)-[1,1'-biphenyl]-4-sulfonimidamide (3ab)

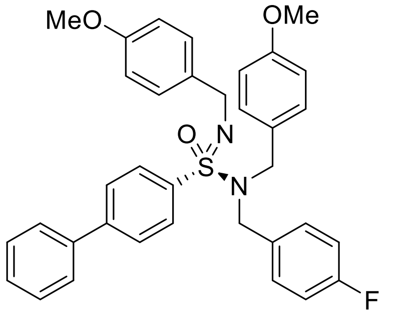

Prepared according to general procedure D, using sulfonimidamide **2a** (23.6 mg, 0.05 mmol, 1.0 equiv.) and 4-fluorobenzyl bromide (6 µL, 0.05 mmol, 1.0 equiv.). Purification by silica gel column chromatography (Pentane:EtOAc, 1:0 to 4:1), afforded the title compound (23.2 mg, 80%) as a colourless oil.

^{1}H NMR (400 MHz, $CDCl_3$) δ_H 8.05 (d, J = 8.5 Hz, 2H, ArH), 7.70 (d, J = 8.5 Hz, 2H, 2 × ArH), 7.66 – 7.60 (m, 2H, 2 × PhH), 7.55 – 7.46 (m, 2H, 2 × PhH), 7.50 – 7.39 (m, 1H, PhH), 7.37 (d, J = 8.5 Hz, 2H, 2 × *p*-OMeArH), 6.99 (dd, J, J_{HF} = 8.5, 5.5 Hz, 2H, 2 × *p*-FArH), 6.94 – 6.82 (m, 6H, 2 × *p*-FArH, 4 × *p*-OMeArH), 6.72 (d, J = 8.5 Hz, 2H, 2 × *p*-OMeArH), 4.51 (d, J = 15.0 Hz, 1H, NC$H_A$$H_B$), 4.48 (d, J = 14.5 Hz, 1H, NC$H_A$$H_B$), 4.46 (d, J = 14.5 Hz, 1H, NC$H_A$$H_B$), 4.15

(d, J = 14.5 Hz, 1H, NCH$_A$$H_B$), 4.10 (d, J = 14.5 Hz, 1H, NCH$_A$$H_B$), 4.08 (d, J = 15.0 Hz, 1H, NCH$_A$$H_B$), 3.79 (s, 3H, OC$H_3$), 3.76 (s, 3H, OC$H_3$).

^{13}C NMR (101 MHz, CDCl$_3$) δ_C 162.2 (d, $^1J_{CF}$ = 246.0 Hz), 159.2, 158.5, 144.9, 139.7, 139.6, 133.6, 132.7 (d, $^4J_{CF}$ = 3.5 Hz), 130.5 (d, $^3J_{CF}$ = 8.0 Hz), 130.2, 129.2, 128.7, 128.5, 128.4, 128.0, 127.6, 127.4, 115.3 (d, $^2J_{CF}$ = 21.5 Hz), 113.9, 113.8, 55.4 (2 × C), 50.9, 50.4, 45.3.

^{19}F NMR (377 MHz, CDCl$_3$) δ_F -114.92 – -115.34 (m).

LRMS (ESI$^+$, m/z) [M+H]$^+$ 581.2.

HRMS (ESI$^+$, m/z) calculated for $(C_{35}H_{34}O_3N_2FS)^+$ 581.2269 [M+H]$^+$, found 581.2264.

IR (CHCl$_3$, v_{max}/cm^{-1}) 1611, 1510, 1246, 1146, 1036, 905, 821, 760, 731, 697, 670.

er was determined by HPLC using a Chiralpak IA-3 column (*n*-hexane:*i*-PrOH, 80:20); τ_{major} = 25.28 min, τ_{minor} = 37.19 min (89:11 *er*).

[α]$_D^{25}$ −2.0° (*c* = 0.75, CHCl$_3$).

R$_f$ 0.45 (Petrol:EtOAc, 4:1).

(*S*)-*N*,*N'*-bis(4-methoxybenzyl)-*N*-(4-(methylthio)benzyl)-[1,1'-biphenyl]-4-sulfonimidamide (3ac)

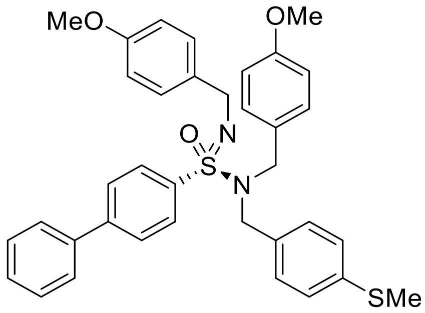

Prepared according to general procedure D, using sulfonimidamide **2a** (23.6 mg, 0.05 mmol, 1.0 equiv.) and 4-(methylthio)benzyl bromide (10.9 mg, 0.05 mmol, 1.0 equiv.). Purification by silica gel column chromatography (Pentane:EtOAc, 1:0 to 4:1), afforded the title compound (25.9 mg, 85%) as a colourless oil.

^{1}H NMR (400 MHz, CDCl$_3$) δ_H 8.05 (d, J = 8.5 Hz, 2H, 2 × ArH), 7.68 (d, J = 8.5 Hz, 2H, 2 × ArH), 7.65 – 7.61 (m, 2H, 2 × PhH), 7.53 – 7.47 (m, 2H, 2 × PhH), 7.46 – 7.39 (m, 1H, PhH), 7.36 (d, J = 8.5 Hz, 2H, 2 × *p*-OMeArH), 7.08 (d, J = 8.5 Hz, 2H, 2 × *p*-SMeArH), 6.93 (d, J = 8.5 Hz, 2H, 2 × *p*-OMeArH), 6.92 (d, J = 8.5 Hz, 2H, 2 × *p*-SMeArH), 6.86 (d, J = 8.5 Hz, 2H, 2 × *p*-OMeArH), 6.72 (d, J = 8.5 Hz, 2H, 2 × *p*-OMeArH), 4.50 (d, J = 15.0 Hz, 1H, NCH_AH$_B$), 4.49 (d, J = 14.5 Hz, 1H, NCH_AH$_B$), 4.48 (d, J = 14.5 Hz, 1H, NCH_AH$_B$), 4.14 (d, J = 14.5 Hz, 1H, NCH$_A$$H_B$), 4.08 (d, J = 14.5 Hz, 1H, NCH$_A$$H_B$), 4.07 (d, J = 15.0 Hz, 1H, NCH$_A$$H_B$), 3.79 (s, 3H, OC$H_3$), 3.76 (s, 3H, OC$H_3$), 2.44 (s, 3H, SC$H_3$).

^{13}C NMR (101 MHz, $CDCl_3$) δ_C 159.1, 158.5, 144.8, 139.8 (2 × C), 137.6, 133.7 (2 × C), 130.2, 129.4, 129.2, 128.7, 128.5, 128.4, 128.0, 127.6, 127.4, 126.6, 113.9, 113.8, 55.4 (2 × C), 50.7, 50.5, 45.3, 16.0.
LRMS (ESI$^+$, m/z) $[M+H]^+$ 609.2.
HRMS (ESI$^+$, m/z) calculated for $(C_{36}H_{37}O_3N_2S_2)^+$ 609.2240 $[M+H]^+$, found 609.2234.
IR ($CHCl_3$, ν_{max}/cm^{-1}) 1611, 1511, 1441, 1277, 1246, 1146, 1035, 903, 818, 763, 733, 698, 671.
er was determined by HPLC using a Chiralpak OD-H column (*n*-hexane:*i*-PrOH, 80:20); τ_{major} = 15.68 min, τ_{minor} = 19.53 min (84:16 *er*).
$[\alpha]_D^{25}$ +4.4° (*c* = 0.75, $CHCl_3$).
R_f 0.39 (Petrol:EtOAc, 4:1).

(*S*)-*N*-(4-cyanobenzyl)-*N,N'*-bis(4-methoxybenzyl)-[1,1'-biphenyl]-4-sulfonimidamide (3ad)

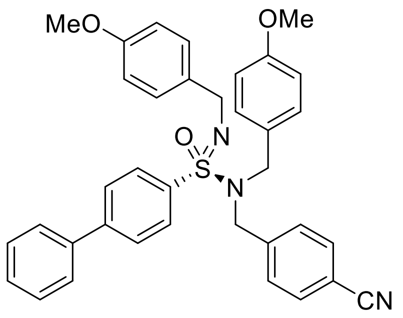

Prepared according to general procedure D, using sulfonimidamide **2a** (23.6 mg, 0.05 mmol, 1.0 equiv.) and 4-(bromomethyl)benzonitrile (9.8 mg, 0.05 mmol, 1.0 equiv.). Purification by silica gel column chromatography (Pentane:EtOAc, 1:0 to 4:1), afforded the title compound (25.3 mg, 86%) as a colourless oil.
^{1}H NMR (400 MHz, $CDCl_3$) δ_H 8.06 (d, *J* = 8.5 Hz, 2H, 2 × Ar*H*), 7.73 (d, *J* = 8.5 Hz, 2H, 2 × Ar*H*), 7.66 – 7.61 (m, 2H, 2 × Ph*H*), 7.56 – 7.46 (m, 2H, Ph*H*), 7.50 – 7.39 (m, 3H, Ph*H*, 2 × *p*-CNAr*H*), 7.36 (d, *J* = 8.5 Hz, 2H, 2 × *p*-OMeAr*H*), 7.13 (d, *J* = 8.5 Hz, 2H, 2 × *p*-CNAr*H*), 6.92 – 6.81 (m, 4H, 4 × *p*-OMeAr*H*), 6.69 (d, *J* = 8.5 Hz, 2H, 2 × *p*-OMeArH), 4.55 (d, *J* = 15.5 Hz, 1H, NCH_AH_B), 4.51 (d, *J* = 14.5 Hz, 1H, NCH_AH_B), 4.37 (d, *J* = 14.5 Hz, 1H, NCH_AH_B), 4.20 (d, *J* = 14.5 Hz, 1H, NCH_AH_B), 4.19 (d, *J* = 14.5 Hz, 1H, NCH_AH_B) 4.11 (d, *J* = 15.5 Hz, 1H, NCH_AH_B), 3.79 (s, 3H, OCH_3), 3.75 (s, 3H, OCH_3).
^{13}C NMR (101 MHz, $CDCl_3$) δ_C 159.4, 158.5, 145.2, 143.1, 139.5, 138.7, 133.3, 132.1, 130.3, 129.2, 129.0, 128.6 (2 × C), 128.0, 127.8, 127.7, 127.4, 118.8, 113.9, 113.8, 111.1, 55.4 (2 × C), 52.2, 51.2, 45.3.
LRMS (ESI$^+$, m/z) $[M+H]^+$ 588.2 & $[M+Na]^+$ 610.2.
HRMS (ESI$^+$, m/z) calculated for $(C_{36}H_{34}O_3N_3S)^+$ 588.2326 $[M+H]^+$, found 588.2311.

IR ($CHCl_3$, v_{max}/cm^{-1}) 2836, 2228, 1610, 1511, 1479, 1463, 1245, 1146, 1034, 902, 819, 762, 725, 698, 671.

er was determined by HPLC using a Chiralpak OD-H column (*n*-hexane:*i*-PrOH, 80:20); τ_{major} = 26.19 min, τ_{minor} = 32.39 min (82.5:17.5 *er*).

[*α*]$_D^{25}$ +8.4° (*c* = 0.75, $CHCl_3$).

R$_f$ 0.22 (Petrol:EtOAc, 4:1).

(*S*)-*N*-(3-formylbenzyl)-*N*,*N'*-bis(4-methoxybenzyl)-[1,1'-biphenyl]-4-sulfonimidamide (3ae)

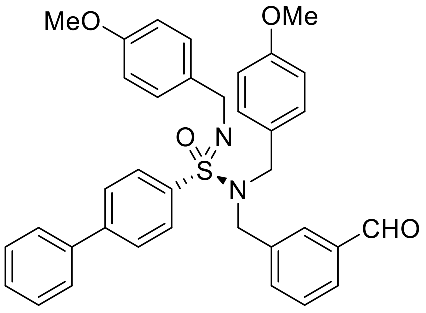

Prepared according to general procedure D, using sulfonimidamide **2a** (23.6 mg, 0.05 mmol, 1.0 equiv.) and 4-(bromomethyl)benzonitrile (10.0 mg, 0.05 mmol, 1.0 equiv.). Purification by silica gel column chromatography (Pentane:EtOAc, 1:0 to 4:1), afforded the title compound (12.7 mg, 43%) as a colourless oil.

^{1}H NMR (400 MHz, $CDCl_3$) δ_H 9.82 (s, 1H, C*H*O), 8.07 (d, *J* = 8.5 Hz, 2H, 2 × Ar*H*), 7.71 (d, *J* = 8.5 Hz, 2H, 2 × Ar*H*), 7.69 – 7.67 (m, 1H, CH_2Ar*H*), 7.66 – 7.58 (m, 2H, 2 × Ph*H*), 7.54 – 7.47 (m, 2H, 2 × Ph*H*), 7.46 – 7.40 (m, 1H, Ph*H*), 7.40 – 7.36 (m, 2H, 2 × CH_2Ar*H*), 7.36 – 7.30 (m, 3H, CH_2Ar*H*, 2 × *p*-OMeAr*H*), 6.92 (d, *J* = 8.5 Hz, 2H, 2 × *p*-OMeAr*H*), 6.86 (d, *J* = 8.5 Hz, 2H, 2 × *p*-OMeAr*H*), 6.70 (d, *J* = 8.5 Hz, 2H, 2 × *p*-OMeAr*H*), 4.58 (d, *J* = 15.0 Hz, 1H, NC$H_A$$H_B$), 4.51 (d, *J* = 14.5 Hz, 1H, NC$H_A$$H_B$), 4.44 (d, *J* = 15.0 Hz, 1H, NC$H_A$$H_B$), 4.19 (d, *J* = 14.5 Hz, 1H, NC$H_A$$H_B$), 4.16 (d, *J* = 15.0 Hz, 2H, 2 × NC$H_A$$H_B$), 3.79 (s, 3H, OC$H_3$), 3.74 (s, 3H, OC$H_3$).

^{13}C NMR (101 MHz, $CDCl_3$) δ_C 192.1, 159.3, 158.6, 145.1, 139.7, 139.1, 138.5, 136.5, 134.7, 133.5, 130.3, 129.9, 129.2, 129.1, 128.7, 128.6, 128.5, 128.2, 128.0, 127.7, 127.5, 114.0, 113.8, 55.5, 55.4, 51.8, 50.9, 45.3.

LRMS (ESI$^+$, m/z) $[M+H]^+$ 591.2.

HRMS (ESI$^+$, m/z) calculated for $(C_{36}H_{35}O_4N_2S)^+$ 591.2312 $[M+H]^+$, found 591.2307.

IR ($CHCl_3$, v_{max}/cm^{-1}) 2850, 1699, 1611, 1512, 1453, 1280, 1247, 1145, 1035, 908, 816, 759, 731, 670.

er was determined by HPLC using a Chiralpak IA-3 column (*n*-hexane:*i*-PrOH, 70:30); τ_{major} = 30.92 min, τ_{minor} = 47.55 min (85:15 *er*).

[α]$_D^{25}$ -1.5° (*c* = 0.75, $CHCl_3$).

R$_f$ 0.34 (Petrol:EtOAc 4:1).

(*S*)-*N,N'*-bis(4-methoxybenzyl)-*N*-(naphthalen-2-ylmethyl)-[1,1'-biphenyl]-4-sulfonimidamide (3af)

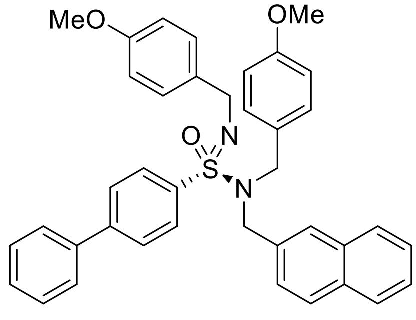

Prepared according to general procedure D, using sulfonimidamide **2a** (23.6 mg, 0.05 mmol, 1.0 equiv.) and 2-(bromomethyl)naphthalene (11.1 mg, 0.05 mmol, 1.0 equiv.). Purification by silica gel column chromatography (Pentane:EtOAc, 1:0 to 4:1), afforded the title compound (24.8 mg, 81%) as a colourless oil.

^{1}H NMR (400 MHz, $CDCl_3$) δ_H 8.09 (d, *J* = 8.5 Hz, 2H, 2 × Ar*H*), 7.82 – 7.75 (m, 1H, CH_2Ar*H*), 7.72 – 7.65 (m, 3H, CH_2Ar*H*, 3 × Ar*H*), 7.65 – 7.57 (m, 3H, CH_2Ar*H*, 2 × Ph*H*), 7.54 – 7.47 (m, 2H, 2 × Ph*H*), 7.47 – 7.41 (m, 2H, CH_2Ar*H*, Ph*H*), 7.37 (d, *J* = 8.5 Hz, 2H, 2 × *p*-OMeAr*H*), 7.31 (d, *J* = 2.0 Hz, 1H, CH_2Ar*H*), 7.16 (dd, *J* = 8.5, 2.0 Hz, 1H, CH_2Ar*H*), 6.94 (d, *J* = 8.5 Hz, 2H, 2 × *p*-OMeAr*H*), 6.85 (d, *J* = 8.5 Hz, 2H, 2 × *p*-OMeAr*H*), 6.71 (d, *J* = 8.5 Hz, 2H, 2 × *p*-OMeAr*H*), 4.72 (d, *J* = 15.0 Hz, 1H, NCH_AH_B), 4.56 (d, *J* = 14.5 Hz, 1H, NCH_AH_B), 4.53 (d, *J* = 14.5 Hz, 1H, NCH_AH_B), 4.28 (d, *J* = 15.0 Hz, 1H, NCH_AH_B), 4.19 (d, *J* = 14.5 Hz, 1H, NCH_AH_B), 4.12 (d, *J* = 14.5 Hz, 1H, NCH_AH_B), 3.78 (s, 3H, OCH_3), 3.73 (s, 3H, OCH_3).

^{13}C NMR (101 MHz, $CDCl_3$) δ_C 159.1, 158.5, 144.9, 140.0, 139.8, 134.2, 133.7, 133.3, 132.8, 130.3, 129.2, 128.7, 128.6, 128.4, 128.2, 128.0, 127.9, 127.7 (2 × C), 127.6, 127.5, 126.7, 126.2, 126.0, 113.9, 113.8, 55.5, 55.4, 51.1, 50.7, 45.3.

LRMS (ESI$^+$, m/z) $[M+H]^+$ 613.2.

HRMS (ESI$^+$, m/z) calculated for $(C_{39}H_{37}O_3N_2S)^+$ 613.2519 $[M+H]^+$, found 613.2514.

IR ($CHCl_3$, ν_{max}/cm^{-1}) 1611, 1511, 1463, 1246, 1145, 1035, 909, 818, 760, 730, 699, 671.

er was determined by HPLC using a Chiralpak IA-3 column (*n*-hexane/*i*-PrOH, 70:30); τ_{major} = 20.24 min, τ_{major} = 33.09 min (81:19 *er*).

[α]$_D^{25}$ +2.8° (*c* = 0.75, $CHCl_3$).

R$_f$ 0.45 (Petrol:EtOAc, 4:1).

(*S*)-*N*-allyl-*N*,*N'*-bis(4-methoxybenzyl)-[1,1'-biphenyl]-4-sulfonimidamidede (3ag)

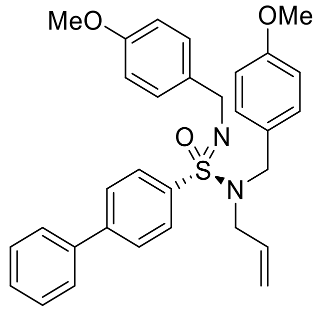

Prepared according to general procedure D, using sulfonimidamide **2a** (23.6 mg, 0.05 mmol, 1.0 equiv.) and allyl bromide (4 μL, 0.05 mmol, 1.0 equiv.). Purification by silica gel column chromatography (Pentane:EtOAc, 1:0 to 4:1), afforded the title compound (20.5 mg, 80%) as a colourless oil.

^{1}H NMR (400 MHz, $CDCl_3$) δ_H 8.07 (d, *J* = 8.5 Hz, 2H, 2 × Ar*H*), 7.71 (d, *J* = 8.5 Hz, 2H, 2 × Ar*H*), 7.67 – 7.59 (m, 2H, 2 × Ph*H*), 7.53 – 7.45 (m, 2H, 2 × Ph*H*), 7.45 – 7.42 (m, 1H), 7.40 (d, *J* = 8.5 Hz, 2H, 2 × *p*-OMeAr*H*), 7.18 (d, *J* = 8.5 Hz, 2H, 2 × *p*-OMeAr*H*), 6.88 (d, *J* = 8.5 Hz, 2H, 2 × *p*-OMeAr*H*), 6.84 (d, *J* = 8.5 Hz, 2H, 2 × *p*-OMeAr*H*), 5.40 (dddd, *J* = 17.0, 10.0, 7.0, 6.0 Hz, 1H, C*H*=CH_2), 5.03 (app. dq, *J* = 10.0, 1.5 Hz, 1H, CH=CH_AH_B), 4.99 (app. dq, *J* = 17.0, 1.5 Hz, 1H, CH=CH_AH_B), 4.51 (d, *J* = 14.5 Hz, 2H, 2 × NCH_AH_B), 4.23 (d, *J* = 14.5 Hz, 1H, NCH_AH_B), 4.20 (d, *J* = 14.5 Hz, 1H, NCH_AH_B), 3.86 – 3.77 (m, 7H, NCH_AH_B, 2 × OCH_3), 3.71 (dd, *J* = 15.5, 7.0 Hz, 1H, NCH_AH_B).

^{13}C NMR (101 MHz, $CDCl_3$) δ_C 159.1, 158.4, 144.7, 139.6, 139.4, 133.7, 133.0, 130.1, 129.1, 128.9, 128.7, 128.4, 128.0, 127.5, 127.4, 119.1, 113.9, 113.7, 55.4 (2 × C), 50.3, 50.0, 45.1.

LRMS (ESI^+, m/z) $[M+H]^+$ 513.2 & $[M+Na]^+$ 535.2.

HRMS (ESI^+, m/z) calculated for $(C_{31}H_{33}O_3N_2S)^+$ 513.2206 $[M+H]^+$, found 513.2197.

IR ($CHCl_3$, v_{max}/cm^{-1}) 2835, 1611, 1510, 1479, 1441, 1242.22, 1143, 1107, 1007, 816, 762, 733, 698, 673.

er was determined by HPLC using a Chiralpak IA-3 column (*n*-hexane:*i*-PrOH, 85:15); τ_{major} = 25.91 min, τ_{minor} = 29.00 min (89.5:10.5 *er*).

$[\alpha]_D^{25}$ −1.2° (*c* = 0.75, $CHCl_3$).

R_f 0.50 (Petrol:EtOAc, 4:1).

(*S*)-*N*,*N'*-bis(4-methoxybenzyl)-*N*-(2-methylallyl)-[1,1'-biphenyl]-4-sulfonimidamide (3ah)

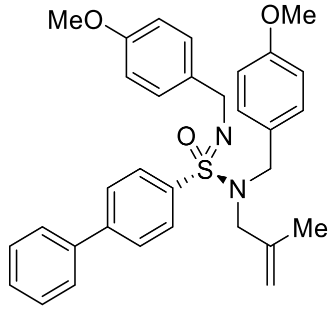

Prepared according to general procedure D, using sulfonimidamide **2a** (23.6 mg, 0.05 mmol, 1.0 equiv.) and 3-bromo-2-methylpropene (5 µL, 0.05 mmol, 1.0 equiv.). Purification by silica gel column chromatography (Pentane:EtOAc, 1:0 to 4:1), afforded the title compound (17.4 mg, 66%) as a colourless oil.

^{1}H NMR (400 MHz, $CDCl_3$) δ_H 8.01 (d, *J* = 8.5 Hz, 2H, 2 × Ar*H*), 7.66 (d, *J* = 8.5 Hz, 2H, 2 × Ar*H*), 7.63 – 7.57 (m, 2H, 2 × Ph*H*), 7.52 – 7.45 (m, 2H, 2 × Ph*H*), 7.44 – 7.39 (m, 1H, PhH), 7.37 (d, *J* = 8.5 Hz, 2H, 2 × *p*-OMeAr*H*), 7.09 (d, *J* = 8.5 Hz, 2H, 2 × *p*-OMeAr*H*), 6.87 (d, *J* = 8.5 Hz, 2H, 2 × *p*-OMeAr*H*), 6.75 (d, *J* = 8.5 Hz, 2H, 2 × *p*-OMeAr*H*), 4.82 (app. q, *J* = 1.0 Hz, 1H, CMe=CH_AH_B), 4.75 (app. q, *J* = 1.0 Hz, 1H, CMe=CH_AH_B), 4.51 (d, *J* = 14.5 Hz, 1H, NCH_AH_B), 4.46 (d, *J* = 14.5 Hz, 1H, NCH_AH_B), 4.16 (d, *J* = 14.5 Hz, 1H, NCH_AH_B), 4.12 (d, *J* = 14.5 Hz, 1H, NCH_AH_B), 3.82 – 3.74 (m, 7H, NCH_AH_B, 2 × OCH_3), 3.64 (d, *J* = 15.0 Hz, 1H, NCH_AH_B) 1.50 (app. t, *J* = 1.5 Hz, 3H, CCH_3).

^{13}C NMR (101 MHz, $CDCl_3$) δ_C 159.1, 158.4, 144.7, 141.1, 139.8, 139.6, 133.8, 130.6, 129.1, 129.0, 128.7, 128.4, 128.0, 127.4 (2 × C), 127.4, 114.4, 113.8, 113.7, 55.5, 55.4, 53.9, 50.9, 45.2, 20.4.

LRMS (ESI^+, m/z) $[M+H]^+$ 527.2.

HRMS (ESI^+, m/z) calculated for $(C_{32}H_{35}O_3N_2S)^+$ 527.2363 $[M+H]^+$, found 527.2360.

IR ($CHCl_3$, v_{max}/cm^{-1}) 1612, 1511, 1442, 1280, 1246, 1146, 1035, 905, 818, 761, 697.

er was determined by HPLC using a Chiralpak AD-H column (*n*-hexane:*i*-PrOH, 80:20); τ_{minor} = 31.24 min, τ_{major} = 36.46 min (11:89 *er*).

$[\alpha]_D^{25}$ −15.6° (*c* = 0.75, $CHCl_3$).

R_f 0.75 (Petrol:EtOAc, 4:1).

(*S*)-*N*-(cyclopropylmethyl)-*N*,*N'*-bis(4-methoxybenzyl)-[1,1'-biphenyl]-4-sulfonimidamide (3ai)

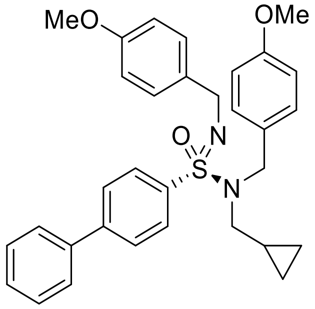

Prepared according to general procedure D, using sulfonimidamide **2a** (23.6 mg, 0.05 mmol, 1.0 equiv.) and (bromomethyl)cyclopropane (5 µL, 0.05 mmol, 1.0 equiv.). Purification by silica gel column chromatography (Pentane:EtOAc, 1:0 to 4:1), afforded the title compound (2.6 mg, 10%) as a colourless oil.

^{1}H NMR (400 MHz, $CDCl_3$) δ_H 8.06 (d, *J* = 8.5 Hz, 2H, 2 × Ar*H*), 7.69 (d, *J* = 8.5 Hz, 2H, 2 × Ar*H*), 7.65 – 7.58 (m, 2H, 2 × Ph*H*), 7.51 – 7.45 (m, 2H, 2 × Ph*H*), 7.44 – 7.36 (m, 3H, Ph*H,* 2 × *p*-OMeAr*H*), 7.18 (d, *J* = 8.5 Hz, 2H, 2 × *p*-OMeAr*H*), 6.87 (d, *J* = 8.5 Hz, 2H, 2 × *p*-OMeAr*H*), 6.82 (d, *J* = 8.5 Hz, 2H, 2 × *p*-OMeAr*H*), 4.56 (d, *J* = 15.0 Hz, 1H, NCH_AH_B), 4.52 (d, *J* = 14.5 Hz, 1H, NCH_AH_B), 4.39 (d, *J* = 15.0 Hz, 1H, NCH_AH_B), 4.24 (d, *J* = 14.5 Hz, 1H, NCH_AH_B), 3.79 (s, 3H, OCH_3), 3.78 (s, 3H, OCH_3), 3.09 (dd, *J* = 14.5, 6.5 Hz, 1H, NCH_AH_B), 2.97 (dd, *J* = 14.5, 7.0 Hz, 1H, NCH_AH_B), 0.68 – 0.54 (m, 1H, $(CH_2)_2CH$), 0.39 – 0.23 (m, 2H, 2 × $CHCH_AH_B$), -0.03 – -0.12 (m, 2H, 2 × $CHCH_AH_B$).

^{13}C NMR (101 MHz, $CDCl_3$) δ_C 159.0, 158.4, 144.6, 139.8, 139.5, 133.9, 129.6, 129.5, 129.1, 128.7, 128.4, 128.0, 127.5, 127.4, 113.9, 113.8, 55.5, 55.4, 52.3, 51.2, 45.2, 9.3, 4.6, 3.8.

LRMS (ESI^+, m/z) $[M+H]^+$ 527.2 & $[M+Na]^+$ 549.2.

HRMS (ESI^+, m/z) calculated for $(C_{32}H_{35}O_3N_2S)^+$ 527.2363 $[M+H]^+$, found 527.2360.

IR ($CHCl_3$, ν_{max}/cm^{-1}) 2925, 1741, 1612, 1512, 1462, 1380, 1245, 1149, 953, 907, 817, 762, 729, 670, 619.

er was determined by HPLC using a Chiralpak IA-3 column (*n*-hexane:*i*-PrOH, 80:20); τ_{major} = 21.97 min, τ_{minor} = 27.96 min (82:18 *er*).

$[\alpha]_D^{25}$ +4.4° (*c* = 0.75, $CHCl_3$).

R_f 0.69 (Petrol:EtOAc 4:1).

(*S*)-*N*-benzyl-*N*,*N'*-bis(4-methoxybenzyl)-4-methylbenzenesulfonimidamide (3b)

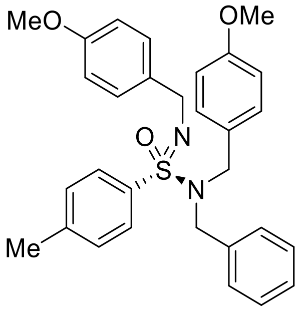

Prepared according to general procedure D, using sulfonimidamide **2b** (20.5 mg, 0.05 mmol, 1.0 equiv.) and benzyl bromide (6 μL, 0.05 mmol, 1.0 equiv.). Purification by flash column chromatography (Pentane:EtOAc, 1:0 to 4:1), afforded the title compound (22.8 mg, 91%) as a white solid.

MP (Petrol/EtOAc) 63-65 °C

^{1}H NMR (400 MHz, $CDCl_3$) δ_H 7.90 (d, *J* = 8.5 Hz, 2H, 2 × Ar*H*), 7.35 (d, *J* = 8.5 Hz, 2H, 2 × *p*-OMeAr*H*), 7.28 (d, *J* = 8.5 Hz, 2H, 2 × Ar*H*), 7.23 – 7.17 (m, 3H, 3 × CH_2Ph*H*), 7.05 – 6.96 (m, 2H, 2 × CH_2Ph*H*), 6.90 (d, *J* = 8.5 Hz, 2H, 2 × *p*-OMeAr*H*), 6.85 (d, *J* = 8.5 Hz, 2H, 2 × *p*-OMeAr*H*), 6.72 (d, *J* = 8.5 Hz, 2H, 2 × *p*-OMeAr*H*), 4.49 (d, *J* = 15.0 Hz, 1H, NCH_AH_B), 4.45 (d, *J* = 14.5 Hz, 1H, NCH_AH_B), 4.44 (d, *J* = 14.5 Hz, 1H, NCH_AH_B), 4.12 (d, *J* = 14.5 Hz, 1H, NCH_AH_B), 4.09 (d, *J* = 14.5 Hz, 1H, NCH_AH_B), 4.06 (d, *J* = 15.0 Hz, 1H, NCH_AH_B), 3.79 (s, 3H, OCH_3), 3.77 (s, 3H, OCH_3), 2.44 (s, 3H, $ArCH_3$).

^{13}C NMR (101 MHz, $CDCl_3$) δ_C 159.1, 158.4, 142.5, 138.1, 136.9, 130.3, 133.7, 129.6, 128.8, 128.7 (2 × C), 128.4, 127.6, 127.4, 113.8 (2 × C), 55.4 (2 × C), 51.0, 50.7, 45.3, 21.6.

LRMS (ESI^+, m/z) $[M+H]^+$ 501.2 & $[M+Na]^+$ 523.1.

HRMS (ESI^+, m/z) calculated for $(C_{30}H_{33}O_3N_2S)^+$ $[M+H]^+$ 501.2206, found 501.2202.

IR ($CHCl_3$, v_{max}/cm^{-1}) 1612, 1512, 1463, 1246, 1176, 1141, 1035, 815, 748.

er was determined by HPLC using a Chiralpak OD-H column (*n*-hexane:*i*-PrOH, 80:20); τ_{minor} = 7.37 min, τ_{major} = 10.05 min (13.5:86.5 *er*).

$[\alpha]_D^{25}$ −1.0° (*c* = 0.75, $CHCl_3$).

R_f 0.52 (Petrol:EtOAc, 3:2).

(*S*)-*N*-benzyl-*N*,*N'*-bis(4-methoxybenzyl)benzenesulfonimidamide (3c)

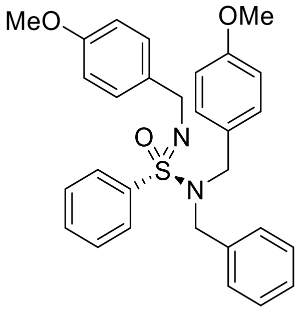

Prepared according to general procedure D, using sulfonimidamide **2c** (19.8 mg, 0.05 mmol, 1.0 equiv.) and benzyl bromide (6 μL, 0.05 mmol, 1.0 equiv.). Purification by flash column chromatography (Pentane:EtOAc, 1:0 to 4:1), afforded the title compound (20.2 mg, 83%) as a colourless oil.

^{1}H NMR (400 MHz, $CDCl_3$) δ_H 8.05 – 7.98 (m, 2H, 2 × Ph*H*), 7.58 – 7.52 (m, 1H, Ph*H*), 7.52 – 7.44 (m, 2H, 2 × Ph*H*), 7.34 (d, *J* = 9.0 Hz, 2H, 2 × *p*-OMeAr*H*), 7.23 – 7.16 (m, 3H, 3 × CH_2Ph*H*), 7.02 – 6.95 (m, 2H, 2 × CH_2Ph*H*), 6.87 (d, *J* = 8.5 Hz, 2H, 2 × *p*-OMeAr*H*), 6.85 (d, *J* = 8.5 Hz, 2H, 2 × *p*-OMeAr*H*), 6.71 (d, *J* = 8.5 Hz, 2H, 2 × *p*-OMeAr*H*), 4.52 (d, *J* = 15.0 Hz, 1H, NCH_AH_B), 4.48 (d, *J* = 14.5 Hz, 1H, NCH_AH_B), 4.46 (d, *J* = 14.5 Hz, 1H, NCH_AH_B), 4.11 (d, *J* = 14.5 Hz, 1H, NCH_AH_B), 4.07 (d, *J* = 15.0 Hz, 1H, NCH_AH_B), 4.04 (d, *J* = 14.5 Hz, 1H, NCH_AH_B), 3.79 (s, 3H, OCH_3), 3.77 (s, 3H, OCH_3).

^{13}C NMR (101 MHz, $CDCl_3$) δ_C 159.1, 158.4, 141.3, 136.8, 133.7, 131.9, 130.3, 129.0, 128.8, 128.7, 128.5, 128.5, 127.5 (2 × C), 113.8 (2 × C), 55.4 (2 × C), 50.8, 50.5, 45.2.

LRMS (ESI$^+$, m/z) $[M+Na]^+$ 509.2.

HRMS (ESI$^+$, m/z) calculated for $(C_{29}H_{31}O_3N_2S)^+$ 487.2050 $[M+H]^+$, found 487.2048.

IR ($CHCl_3$, ν_{max}/cm^{-1}) 2920, 1612, 1511, 1445, 1279, 1246, 1146, 1034, 894, 817, 736, 690.

er was determined by HPLC using a Chiralpak OD-H column (*n*-hexane:*i*-PrOH, 80:20); τ_{minor} = 7.72 min, τ_{minor} = 9.09 min (13:87 *er*).

$[\alpha]_D^{25}$ −1.5° (*c* = 0.75, $CHCl_3$).

R_f 0.57 (Petrol:EtOAc, 4:1).

(*S*)-*N*-benzyl-*N*,*N'*-bis(4-methoxybenzyl)-3-methylbenzenesulfonimidamide (3d)

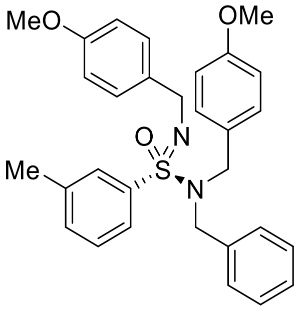

Prepared according to general procedure D, using sulfonimidamide **2d** (20.5 mg, 0.05 mmol, 1.0 equiv.) and benzyl bromide (6 μL, 0.05 mmol, 1.0 equiv.). Purification by flash column chromatography (Pentane:EtOAc, 1:0 to 4:1), afforded the title compound (16.0 mg, 64%) as a colourless oil.

^{1}H NMR (400 MHz, $CDCl_3$) δ_H 7.86 – 7.77 (m, 1H, Ar*H*), 7.80 – 7.74 (m, 1H, Ar*H*), 7.41 – 7.30 (m, 4H, 2 × Ar*H*, 2 × *p*-OMeAr*H*), 7.24 – 7.16 (m, 3H, 3 × CH_2Ph*H*), 7.05 – 6.96 (m, 2H, 2 × CH_2Ph*H*), 6.89 (d, *J* = 8.7 Hz, 2H, 2 × *p*-OMeAr*H*), 6.85 (d, *J* = 8.5 Hz, 2H, 2 × *p*-OMeAr*H*), 6.72 (d, *J* = 8.5 Hz, 2H, 2 × *p*-OMeAr*H*), 4.50 (d, *J* = 15.0 Hz, 1H, NCH_AH_B), 4.46 (d, *J* = 14.5 Hz, 2H, NCH_AH_B), 4.45 (d, *J* = 14.5 Hz, 1H, NCH_AH_B), 4.11 (d, *J* = 14.5 Hz, 1H, NCH_AH_B), 4.09 (d, *J* = 15.0 Hz, 1H, NCH_AH_B), 4.05 (d, *J* = 14.5 Hz, 1H, NCH_AH_B), 3.79 (s, 3H, OCH_3), 3.77 (s, 3H, OCH_3), 2.39 (s, 3H, $ArCH_3$).

^{13}C NMR (101 MHz, $CDCl_3$) δ_C 159.1, 158.4, 140.9, 139.0, 136.9, 133.8, 132.7, 130.3, 128.8, 128.7, 128.6, 128.4, 127.8, 127.5, 124.7, 113.8 (2 × C), 55.4 (2 × C), 50.9, 50.6, 45.3, 21.6.

LRMS (ESI$^+$, m/z) $[M+H]^+$ 501.2 & $[M+Na]^+$ 523.2.

HRMS (ESI$^+$, m/z) calculated for $(C_{30}H_{33}O_3N_2S)^+$ $[M+H]^+$ 501.2206, found 501.2204.

IR ($CHCl_3$, ν_{max}/cm^{-1}) 1745, 1612, 1512, 1457, 1370, 1245, 1041, 910, 732.

er was determined by HPLC using a Chiralpak IA-3 column (*n*-hexane:*i*-PrOH, 80:20); τ_{major} = 10.13 min, τ_{minor} = 13.22 min (85.5:14.5 er).

$[\alpha]_D^{25}$ −1.9° (*c* = 0.75, $CHCl_3$).

R_f 0.52 (Petrol:EtOAc, 4:1).

(*S*)-*N*-benzyl-*N*,*N'*-bis(4-methoxybenzyl)-2-methylbenzenesulfonimidamide (3e)

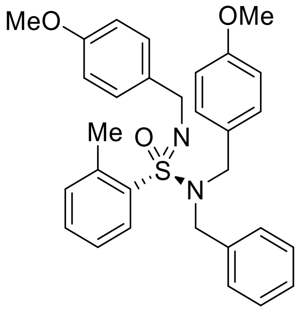

Prepared according to general procedure D, using sulfonimidamide **2e** (20.5 mg, 0.05 mmol, 1.0 equiv.) and benzyl bromide (6 μL, 0.05 mmol, 1.0 equiv.). Purification by flash column chromatography (Pentane:EtOAc, 1:0 to 4:1), afforded the title compound (12.5 mg, 50%) as a colourless oil.

^{1}H NMR (400 MHz, $CDCl_3$) δ_H 8.06 (dd, *J* = 8.0, 1.5 Hz, 1H, Ar*H*), 7.40 (app. td, *J* = 7.5, 1.5 Hz, 1H, Ar*H*), 7.33 – 7.18 (m, 7H, 2 × Ar*H*, 3 × CH_2Ph*H*, 2 × *p*-OMeAr*H*), 7.12 – 7.04 (m, 2H, 2 × CH_2Ph*H*), 6.96 (d, *J* = 8.5 Hz, 2H, 2 × *p*-OMeAr*H*), 6.82 (d, *J* = 8.5 Hz, 2H, 2 × *p*-OMeAr*H*), 6.78 (d, *J* = 8.5 Hz, 2H, 2 × *p*-OMeAr*H*), 4.50 (d, *J* = 15.0 Hz, 1H, NCH_AH$_B$), 4.46 (d, *J* = 14.5 Hz, 1H, NCH_AH$_B$), 4.32 (d, *J* = 14.5 Hz, 1H, NCH_AH$_B$), 4.23 (d, *J* = 15.0 Hz, 1H, NCH$_A$$H_B$), 4.17 (d, *J* = 14.5 Hz, 1H, NCH$_A$$H_B$), 4.02 (d, *J* = 14.5 Hz, 1H, NCH$_A$$H_B$), 3.79 (s, 3H, OC$H_3$), 3.78 (s, 3H, OC$H_3$), 2.65 (s, 3H, ArC$H_3$).

^{13}C NMR (101 MHz, $CDCl_3$) δ_C 159.2, 158.4, 139.9, 138.0, 136.8, 133.9, 133.0, 131.9, 130.6, 130.1, 129.1, 128.7, 128.6, 128.5, 127.7, 126.0, 113.9, 113.7, 55.4 (2 × C), 49.8, 49.4, 45.9, 21.1.

LRMS (ESI$^+$, m/z) $[M+H]^+$ 501.2.

HRMS (ESI$^+$, m/z) calculated for $(C_{30}H_{33}O_3N_2S)^+$ 501.2206 $[M+H]^+$, found 501.2209.

IR ($CHCl_3$, ν_{max}/cm^{-1}) 2911, 1611, 1511, 1456, 1246, 1175, 1150, 1129, 1036, 893, 817, 757, 731, 702.

er was determined by HPLC using a Chiralpak OD-H column (n-hexane/i-PrOH, 85:15); τ_{minor} = 6.75 min, τ_{minor} = 8.31 min (36.5:63.5 *er*).

$[\alpha]_D^{25}$ +4.0° (*c* = 0.75, $CHCl_3$).

R_f 0.39 (Petrol:EtOAc, 4:1).

(*S*)-*N*-benzyl-4-fluoro-*N*,*N'*-bis(4-methoxybenzyl)benzenesulfonimidamide (3f)

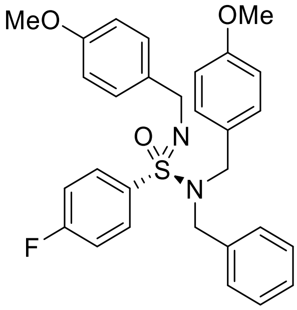

Prepared according to general procedure D, using sulfonimidamide **2f** (20.7 mg, 0.05 mmol, 1.0 equiv.) and benzyl bromide (6 μL, 0.05 mmol, 1.0 equiv.). Purification by flash column chromatography (Pentane:EtOAc, 1:0 to 4:1), afforded the title compound (22.7 mg, 90%) as a colourless oil.

^{1}H NMR (400 MHz, $CDCl_3$) δ_H 7.98 (dd, *J*, J_{HF} = 9.0, 5.0 Hz, 2 × Ar*H*), 7.32 (d, *J* = 8.5 Hz, 2H, 2 × *p*-OMeAr*H*), 7.25 – 7.19 (m, 3H, 3 × CH_2Ph*H*), 7.13 (app. t, *J*, J_{HF} = 8.5 Hz, 2H, 2 × Ar*H*), 7.05 – 6.96 (m, 2H, 2 × CH_2Ph*H*), 6.90 (d, *J* = 8.5 Hz, 2H, 2 × *p*-OMeAr*H*), 6.84 (d, *J* = 8.5 Hz, 2H, 2 × *p*-OMeAr*H*), 6.73 (d, *J* = 8.5 Hz, 2H, 2 × *p*-OMeAr*H*), 4.50 (d, *J* = 15.0 Hz, 1H, NCH_AH_B), 4.46 (d, *J* = 15.5 Hz, 1H, NCH_AH_B), 4.43 (d, *J* = 14.5 Hz, 1H, NCH_AH_B), 4.07 (app. t, *J* = 14.0 Hz, 3H, 3 × NCH_AH_B), 3.78 (s, 3H, OCH_3), 3.77 (s, 3H, OCH_3).

^{13}C NMR (101 MHz, $CDCl_3$) δ_C 164.8 (d, $^1J_{CF}$ = 253.5 Hz), 159.2, 158.5, 137.4 (d, $^4J_{CF}$ = 3.0 Hz), 136.6, 133.5, 130.2, 130.1 (d, $^3J_{CF}$ = 9.0 Hz), 128.8, 128.7, 128.5, 128.3, 127.6, 116.0 (d, $^2J_{CF}$ = 22.5 Hz), 113.9, 113.8, 55.4 (2 × C), 50.8, 50.5, 45.3.

^{19}F NMR (377 MHz, $CDCl_3$) δ_F -107.20 – -107.38 (m).

LRMS (ESI$^+$, m/z) $[M+Na]^+$ 527.2.

HRMS (ESI$^+$, m/z) calculated for $(C_{29}H_{30}O_3N_2FS)^+$ 505.1956 $[M+H]^+$, found 505.1955.

IR ($CHCl_3$, v_{max}/cm^{-1}) 1612, 1588, 1511, 1492, 1456, 1277, 1246, 1144, 1035, 894, 818, 751, 699.

er was determined by HPLC using a Chiralpak AD-H column (*n*-hexane:*i*-PrOH, 85:15); τ_{minor} = 21.36 min, τ_{major} = 34.89 min (82.5:17.5 *er*).

$[\alpha]_D^{25}$ −1.9° (*c* = 0.75, $CHCl_3$).

R_f 0.57 (Petrol:EtOAc, 4:1).

(*S*)-*N*-benzyl-*N*,*N*'-bis(4-methoxybenzyl)thiophene-2-sulfonimidamide (3g)

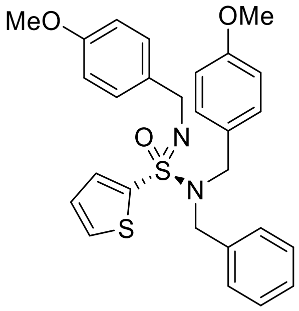

Prepared according to general procedure D, using sulfonimidamide **2g** (20.1 mg, 0.05 mmol, 1.0 equiv.) and benzyl bromide (6 µL, 0.05 mmol, 1.0 equiv.). Purification by flash column chromatography (Pentane:EtOAc, 1:0 to 4:1), afforded the title compound (19.7 mg, 80%) as a pale yellow oil.

^{1}H NMR (400 MHz, $CDCl_3$) δ_H 7.54 – 7.47 (m, 2H, 2 × HetAr*H*), 7.33 (d, *J* = 8.5 Hz, 2H, 2 × *p*-OMeAr*H*), 7.29 – 7.17 (m, 3H, 3 × CH_2Ph*H*), 7.11 – 7.00 (m, 3H, HetAr*H*, 2 × CH_2Ph*H*), 6.93 (d, *J* = 8.5 Hz, 2H, 2 × *p*-OMeAr*H*), 6.84 (d, *J* = 8.5 Hz, 2H, 2 × *p*-OMeAr*H*), 6.74 (d, *J* = 8.5 Hz, 2H, 2 × *p*-OMeAr*H*), 4.50 (d, *J* = 15.0 Hz, 1H), 4.46 (d, *J* = 15.0 Hz, 1H, NCH_AH_B), 4.41 (d, *J* = 14.5 Hz, 1H, NCH_AH_B), 4.18 (d, *J* = 15.0 Hz, 1H, NCH_AH_B), 4.15 (d, *J* = 15.0 Hz, 1H, NCH_AH_B), 4.12 (d, *J* = 14.5 Hz, 1H, NCH_AH_B), 3.78 (s, 3H, OCH_3), 3.77 (s, 3H, OCH_3).

^{13}C NMR (101 MHz, $CDCl_3$) δ_C 159.1, 158.4, 142.6, 136.6, 133.4, 131.4, 130.7, 130.3, 128.8, 128.5 (2 × C), 128.3, 127.6, 127.5, 113.9, 113.8, 55.4 (2 × C), 51.1, 50.8, 45.6.

LRMS (ESI$^+$, m/z) $[M+H]^+$ 493.2.

HRMS (ESI$^+$, m/z) calculated for $(C_{27}H_{29}O_3N_2S_2)^+$ 493.1590 $[M+H]^+$, found 493.1611.

IR ($CHCl_3$, ν_{max}/cm^{-1}) 1612, 1512, 1455, 1281, 1247, 1147, 1035, 908, 817, 731, 667, 620.

er was determined by HPLC using a Chiralpak OD-H column (*n*-hexane:*i*-PrOH, 80:20); τ_{minor} = 9.82 min, τ_{major} = 11.72 min (11:89 *er*).

$[\alpha]_D^{25}$ −10.8° (*c* = 0.5, $CHCl_3$).

R_f 0.40 (Petrol:EtOAc, 4:1).

(*S*)-*N*-benzyl-*N*,*N'*-bis(4-methoxybenzyl)-4-(5-(p-tolyl)-3-(trifluoromethyl)-1H-pyrazol-1-yl)benzenesulfonimidamide (3h)

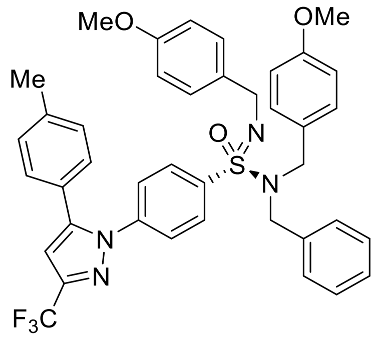

Prepared according to general procedure D, using sulfonimidamide **2h** (31.0 mg, 0.05 mmol, 1.0 equiv.) and benzyl bromide (6 µL, 0.05 mmol, 1.0 equiv.). Purification by flash column chromatography (Pentane:EtOAc, 1:0 to 4:1), afforded the title compound (24.5 mg, 69%) as a colourless oil.

^{1}H NMR (400 MHz, $CDCl_3$) δ_H 7.96 (d, *J* = 9.0 Hz, 2H, 2 × Ar*H*), 7.41 (d, *J* = 9.0 Hz, 2H, 2 × Ar*H*), 7.32 (d, *J* = 8.5 Hz, 2H, 2 × *p*-OMeAr*H*), 7.27 – 7.20 (m, 3H, 3 × CH_2Ph*H*), 7.15 (d, *J* = 8.5 Hz, 2H, 2 × Tol*H*), 7.10 (d, *J* = 8.5 Hz, 2H, 2 × Tol*H*), 7.07 – 6.99 (m, 2H, 2 × CH_2Ph*H*), 6.90 (d, *J* = 8.5 Hz, 2H, 2 × *p*-OMeAr*H*), 6.85 (d, *J* = 8.5 Hz, 2H, 2 × *p*-OMeAr*H*), 6.74 (s, 1H, HetAr*H*), 6.73 (d, *J* = 8.5 Hz, 2H, 2 × *p*-OMeAr*H*), 4.48 (d, *J* = 15.0 Hz, 1H, NCH_AH_B), 4.45 (d, *J* = 14.5 Hz, 1H, NCH_AH_B), 4.41 (d, *J* = 15.0 Hz, 1H, NCH_AH_B), 4.07 (app. t, *J* = 15.0 Hz, 3H, 3 × NCH_AH_B), 3.79 (s, 3H, OCH_3), 3.76 (s, 3H, OCH_3), 2.37 (s, 3H, TolCH_3).

^{13}C NMR (101 MHz, $CDCl_3$) δ_C 159.2, 158.5, 145.3, 144.1 (q, $^2J_{CF}$ = 38.5 Hz), 141.9, 140.5, 139.8, 136.5, 133.3, 130.3, 129.9, 128.8 (2 × C), 128.7, 128.6, 128.5, 128.1, 127.7, 126.0, 125.4, 121.3 (q, $^1J_{CF}$ = 269.0 Hz), 113.9, 113.8, 106.3 (q, $^3J_{CF}$ = 2.5 Hz), 55.4 (2 × C), 51.1, 50.7, 45.2, 21.5.

^{19}F NMR (377 MHz, $CDCl_3$) δ_F -62.36.

HRMS (ESI^+, m/z) calculated for $(C_{40}H_{38}O_3N_4F_3S)^+$ 711.2611 $[M+H]^+$, found 711.2606.

IR ($CHCl_3$, ν_{max}/cm^{-1}) 1612, 1511, 1471, 1373, 1237, 1136, 1036, 975, 894, 812, 771, 630.

er was determined by HPLC using a Chiralpak IA-3 column (n-hexane/i-PrOH, 80:20); τ_{major} = 15.14 min, τ_{minor} = 22.93 min (73.5:26.5 *er*).

$[\alpha]_D^{25}$ −12.7° (*c* = 0.75, $CHCl_3$).

R_f 0.52 (Petrol:EtOAc, 4:1).

(*S*)-*N*-benzyl-*N*,*N'*-bis(4-methoxybenzyl)-2-methylprop-1-ene-1-sulfonimidamide (3i)

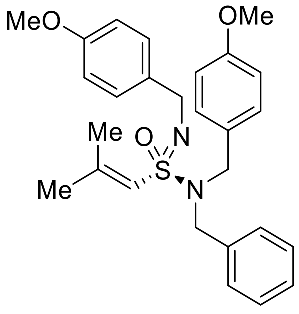

Prepared according to general procedure D, using sulfonimidamide **2i** (18.7 mg, 0.05 mmol, 1.0 equiv.) and benzyl bromide (6 μL, 0.05 mmol, 1.0 equiv.). Purification by flash column chromatography (Pentane:EtOAc, 1:0 to 1:1), afforded the title compound (14.4 mg, 62%) as a colourless oil.

^{1}H NMR (400 MHz, $CDCl_3$) δ_H 7.36 – 7.26 (m, 7H, 2 × *p*-OMeAr*H*, 5 × CH_2Ph*H*), 7.18 (d, *J* = 8.5 Hz, 2H, 2 × *p*-OMeAr*H*), 6.84 (d, *J* = 8.5 Hz, 2H, 2 × *p*-OMeAr*H*), 6.82 (d, *J* = 8.5 Hz, 2H, 2 × *p*-OMeAr*H*), 5.99 (app. p, *J* = 1.5 Hz, 1H, C=C*H*), 4.42 (d, *J* = 14.5 Hz, 1H, NCH_AH_B), 4.38 (d, *J* = 14.0 Hz, 1H, NCH_AH_B), 4.35 (d, *J* = 14.0 Hz, 1H, NCH_AH_B), 4.20 (d, *J* = 14.5 Hz, 1H, NCH_AH_B), 4.17 (d, *J* = 14.0 Hz, 1H, NCH_AH_B), 3.95 (d, *J* = 14.5 Hz, 1H, NCH_AH_B), 3.81 (s, 3H, OCH_3), 3.77 (s, 3H, OCH_3), 2.14 (d, *J* = 1.5 Hz, 3H, CCH_3), 1.80 (d, *J* = 1.5 Hz, 3H, CCH_3).

^{13}C NMR (101 MHz, $CDCl_3$) δ_C 159.2, 158.4, 149.6, 136.9, 133.8, 130.6, 129.0 (2 × C), 128.7, 128.6, 127.7, 125.4, 113.9, 113.8, 55.4 (2 × C), 50.2, 49.9, 45.3, 26.8, 19.6.

LRMS (ESI$^+$, m/z) $[M+H]^+$ 465.2.

HRMS (ESI$^+$, m/z) calculated for $(C_{27}H_{33}O_3N_2S)^+$ 465.2182 $[M+H]^+$, found 465.2192.

IR ($CHCl_3$, v_{max}/cm^{-1}) 1611, 1511, 1441, 1244, 1174, 1131, 1035, 894, 817, 751, 703, 614.

er was determined by HPLC using a Chiralpak IA-3 column (*n*-hexane:*i*-PrOH, 80:20); τ_{major} = 9.31 min, τ_{minor} = 12.31 min (73.5:26.5 *er*).

$[\alpha]_D^{25}$ +8.5° (*c* = 0.75, $CHCl_3$).

R_f 0.19 (Petrol:EtOAc, 4:1).

(*S*)-*N*-benzyl-*N*,*N'*-bis(4-methoxybenzyl)methanesulfonimidamide (3j)

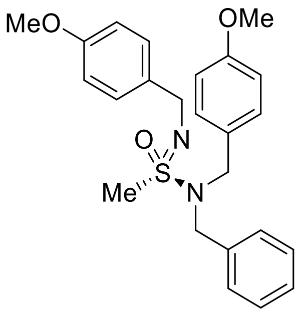

Prepared according to general procedure D, using sulfonimidamide **2j** (16.7 mg, 0.05 mmol, 1.0 equiv.) and benzyl bromide (6 μL, 0.05 mmol, 1.0 equiv.). Purification by flash column

chromatography (Pentane:EtOAc, 1:0 to 1:1), afforded the title compound (10.0 mg, 47%) as a pale yellow oil.

^{1}H NMR (400 MHz, $CDCl_3$) δ_H 7.40 – 7.31 (m, 5H, 5 × CH_2Ph*H*), 7.29 (d, *J* = 8.5 Hz, 2H, 2 × *p*-OMeAr*H*), 7.25 (d, *J* = 8.5 Hz, 2H, 2 × *p*-OMeAr*H*), 6.88 (d, *J* = 8.5 Hz, 2H, 2 × *p*-OMeAr*H*), 6.83 (d, *J* = 8.5 Hz, 2H, 2 × *p*-OMeAr*H*), 4.40 – 4.28 (m, 5H, 3 × NCH_AH_B, 2 × NCH_AH_B), 4.00 (d, *J* = 14.0 Hz, 1H, NCH_AH_B), 3.82 (s, 3H, OCH_3), 3.78 (s, 3H, OCH_3), 2.77 (s, 3H, SCH_3).

^{13}C NMR (101 MHz, $CDCl_3$) δ_C 159.4, 158.5, 136.4, 133.6, 130.5, 129.0 (2 × C), 128.9, 128.1, 127.9, 114.2, 113.9, 55.4 (2 × C), 50.2, 49.8, 45.3, 41.1.

LRMS (ESI^+, m/z) $[M+Na]^+$ 447.2.

HRMS (ESI^+, m/z) calculated for $(C_{24}H_{29}O_3N_2S)^+$ 425.1893 $[M+H]^+$, found 425.1898.

IR ($CHCl_3$, v_{max}/cm^{-1}) 1727, 1611, 1512, 1457, 1247, 1178, 1136, 1080, 1035, 972, 893, 817, 750, 703.

er was determined by HPLC using a Chiralpak IA-3 column (*n*-hexane:*i*-PrOH, 80:20); τ_{minor} = 10.69 min, τ_{major} = 15.14 min (32:68 *er)*.

$[\alpha]_D^{25}$ −8.7° (*c* = 0.5, $CHCl_3$).

R_f 0.27 (Petrol:EtOAc, 4:1).

7.8. Authentic Sample Synthesis

(*R*)-*N*-benzyl-*N,N'*-bis(4-methoxybenzyl)-4-methylbenzenesulfonimidamide ((*R*)-3b)

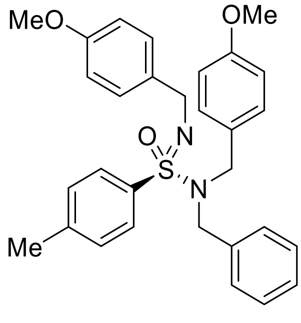

The title compound was prepared according to the modified literature procedure.[7b]

(*S*)-sulfinamide **1** (606 mg, 2.20 mmol, 1.0 equiv.) was dissolved in THF (18 mL) and cooled to –25 °C using an immersion cooler. To this solution, was added *t*-butyl hypochlorite (0.32 mL, 2.86 mmol, 1.3 equiv.) by slow dropwise addition. The reaction was stirred at –25 °C for 20 min and then *N*-benzyl-1-(4-methoxyphenyl)methanamine **8** (1.05 g, 4.84 mmol, 2.2 equiv.) dissolved in THF (4.4 mL) was added. The reaction mixture was left to stir at –25 °C for 36 h, using the immersion cooler apparatus. The reaction was quenched with water (10 mL) and extracted EtOAc × 3 (50 mL), the combined organic extracts were dried with sodium sulfate and concentrated in vacuo. Purification by flash column chromatography (Petrol:EtOAc, 9:1 to

4:1), afforded the title compound (597 mg, 54%) as a white solid. The spectroscopic data is in agreement with previously synthesised compound **3b**.

er was determined by HPLC using a Chiralpak OD-H column (*n*-hexane:*i*-PrOH, 80:20); τ_{major} = 7.37 min, τ_{minor} = 10.05 min (92:8 *er*).

$[\alpha]_D^{25}$ +2.9° (*c* = 0.75, $CHCl_3$).

7.9. Deprotections

(*R*)-*N*-benzyl-*N*-(4-methoxybenzyl)-4-methylbenzenesulfonimidamide (5)

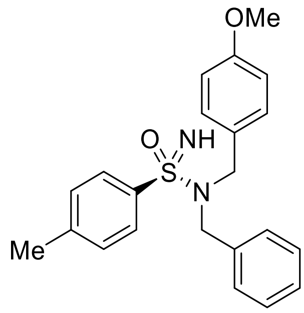

Sulfonimidamide **(*R*)-3b** (50.1 mg, 0.10 mmol, 1.0 equiv.), was added to a reaction vial and dissolved in toluene (1 mL). To this solution, was added DDQ (34.1 mg, 0.15 mmol, 1.5 equiv.) and the reaction was left to stir at room temperature for 15 min. After this time period, the crude mixture was directly purified by flash column chromatography (Petrol:EtOAc, 9:1 to 1:1), affording the title compound (31.5 mg, 83%) as a yellow oil.

^{1}H NMR (400 MHz, $CDCl_3$) δ_H 7.88 (d, *J* = 8.0 Hz, 2H, 2 × Ar*H*), 7.30 (d, *J* = 8.0 Hz, 2H, 2 × Ar*H*), 7.24 – 7.18 (m, 3H, 3 × CH_2Ph*H*), 7.10 – 7.01 (m, 2H, 2 × CH_2Ph*H*), 6.95 (d, *J* = 8.5 Hz, 2H, 2 × *p*-OMeAr*H*), 6.73 (d, *J* = 8.5 Hz, 2H, 2 × *p*-OMeAr*H*), 4.30 (app. d, *J* = 14.0 Hz, 4H, 2 × NCH_AH_B), 3.76 (s, 3H, OCH_3), 2.44 (s, 3H, ArCH_3).

^{13}C NMR (101 MHz, $CDCl_3$) δ_C 159.2, 143.2, 137.6, 136.8, 130.0, 129.7 (2 × C), 128.5 (2 × C), 127.7, 127.6, 113.9, 55.4, 51.7, 51.4, 21.6.

LRMS (ESI$^+$, m/z) $[M+Na]^+$ 403.2.

HRMS (ESI$^+$, m/z) calculated for $(C_{22}H_{25}O_2N_2S)^+$ 381.1631 $[M+H]^+$, found 381.1632.

IR ($CHCl_3$, v_{max}/cm^{-1}) 3287, 2918, 1611, 1586, 1512, 1494, 1455, 1367, 1303, 1248, 1176, 1133, 1110, 1074, 1031, 982, 938, 914, 893, 816, 765, 732, 698, 643.

er was determined by HPLC using a Chiralpak IA-3 column (*n*-hexane:*i*-PrOH, 85:15); τ_{minor} = 31.11 min, τ_{major} = 32.91 min (8:92 *er*).

$[\alpha]_D^{25}$ −33.8° (*c* = 0.75, $CHCl_3$).

R_f 0.50 (Petrol:EtOAc, 1:1).

Note: To obtain a sample of the racemic material for chiral-phase HPLC analysis the reaction was conducted with the racemic starting material under identical conditions.

(*R*)-*N'*-benzyl-4-methylbenzenesulfonimidamide (6)

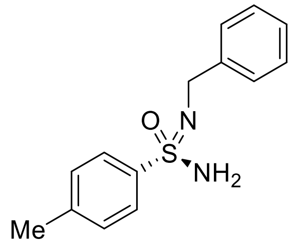

Sulfonimidamide **(*R*)-3b** (50.1 mg, 0.10 mmol, 1.0 equiv.), was added to a microwave vial and sealed with a microwave vial cap. The sulfonimidamide **(*R*)-3b** was then dissolved in TFA (1 mL) and heated to 80 °C. The reaction was left to stir for 3.5 h, after which it was quenched by the dropwise addition of saturated aqueous sodium carbonate solution until effervescence stopped. The reaction was extracted with CH_2Cl_2 × 3 (10 mL) and the combined organic extracts were dried with sodium sulfate then concentrated in vacuo. Purification by flash column chromatography (Petrol:EtOAc, 4:1 to 1:4), afforded the title compound (21.2 mg, 81%) as a yellow oil.

Note: To obtain a sample of the racemic material for chiral-phase HPLC analysis the reaction was conducted with the racemic starting material under identical conditions.

^{1}H NMR (400 MHz, $CDCl_3$) δ_H 7.79 (d, J = 8.5 Hz, 2H, 2 × Ar*H*), 7.35 – 7.26 (m, 3H, 3 × CH_2Ph*H*), 7.25 – 7.13 (m, 4H, 2 × Ar*H*, 2 × CH_2Ph*H*), 5.97 (br. s, 2H, NH_2), 4.19 (d, J = 14.5 Hz, 1H, NC$H_A$$H_B$), 4.10 (d, J = 14.5 Hz, 1H, NC$H_A$$H_B$), 2.43 (s, 3H, ArC$H_3$).

^{13}C NMR (101 MHz, $CDCl_3$) δ_C 145.5, 135.8, 130.2, 128.8, 128.1 (3 × C), 128.0, 46.6, 21.7.

LRMS (ESI$^+$, m/z) [M+H]$^+$ 261.0 & [M+Na]$^+$ 283.0.

HRMS (ESI$^+$, m/z) calculated for $(C_{14}H_{17}ON_2S)^+$ 261.1056 [M+H]$^+$, found 261.1056.

IR ($CHCl_3$, v_{max}/cm^{-1}), 3257, 2922, 1598, 1495, 1454, 1361, 1247, 1183, 1131, 1103, 1064, 1026, 1012, 814, 735, 698, 646.

er was determined by HPLC using a Chiralpak IA-3 column (*n*-hexane:*i*-PrOH, 85:15); τ_{major} = 14.91 min, τ_{minor} = 17.74 min (92:8 *er*).

$[\alpha]_D^{25}$ −21.2° (*c* = 0.75, $CHCl_3$).

R_f 0.21 (Petrol:EtOAc, 1:1).

8. Crystallography

8.1. General Comments

Crystallographic data was collected on an Agilent SuperNova diffractometer fitted with an Oxford CryoSystems CryoStream unit. Raw frame data were reduced using CrysAlisPro, including unit cell determination and refinement, integration of intensities and associated correction. The structures were solved using SHELXT[16] and refined using full-matrix least squares refinement on all F^2 data with SHELXL[17] using the interface OLEX2.[18]

9. References

[1] Y. Liu; Z. Wang; B. Guo; Q. Cai, *Tetrahedron Lett.* **2016**, *57*, 2379.

[2] a) Y. Chen; J. Gibson, *RSC Advances* **2015**, *5*, 4171; b) P. K. Chinthakindi; A. Benediktsdottir; P. I. Arvidsson; Y. Chen; A. Sandström, *Eur. J. Org. Chem.* **2020**, 3796.

[3] S. Higashibayashi; H. Tohmiya; T. Mori; K. Hashimoto; M. Nakata, *Synlett* **2004**, *2004*, 457.

[4] A. F. Garrido-Castro; N. Salaverri; M. C. Maestro; J. Alemán, *Org. Lett.* **2019**, *21*, 5295.

[5] M. Revés; T. Achard; J. Solà; A. Riera; X. Verdaguer, *J. Org. Chem.* **2008**, *73*, 7080.

[6] E. U. Jonsson; C. R. Johnson, *J. Am. Chem. Soc.* **1971**, *93*, 5308.

[7] a) M. Steurer; C. Bolm, *J. Org. Chem.* **2010**, *75*, 3301; b) C. Worch; I. Atodiresei; G. Raabe; C. Bolm, *Chem. Eur. J.* **2010**, *16*, 677.

[8] F. A. Davis; Y. Zhang; Y. Andemichael; T. Fang; D. L. Fanelli; H. Zhang, *J. Org. Chem.* **1999**, *64*, 1403.

[9] G.-J. Li; Y.-L. Pan; Y.-L. Liu; H.-F. Xu; J.-Z. Chen, *Tetrahedron Lett.* **2019**, *60*, 151260.

[10] D. S. Karanewsky; J. R. Fotsing; C. Tachdjian; M. Arellano, US9247759, B2, **2016**.

[11] P. R. Likhar; R. Arundhathi; M. L. Kantam; P. S. Prathima, *Eur. J. Org. Chem.* **2009**, *2009*, 5383.

[12] T. Q. Davies; A. Hall; M. C. Willis, *Angew. Chem. Int. Ed.* **2017**, *56*, 14937.

[13] M. Lian; Z. Li; J. Du; Q. Meng; Z. Gao, **2010**, *2010*, 6525.

[14] B. Xiang; K. M. Belyk; R. A. Reamer; N. Yasuda, *Angew. Chem. Int. Ed.* **2014**, *53*, 8375.

[15] A. F. Abdel-Magid; K. G. Carson; B. D. Harris; C. A. Maryanoff; R. D. Shah, *J. Org. Chem.* **1996**, *61*, 3849.

[16] Sheldrick, G. M. *Acta Cryst.* **2015**, *71A*, 3.

[17] Sheldrick, G. M. *Acta Cryst.* **2015**, *71C*, 3.

[18] Dolomanov, O. V.; Bourhis, L. J.; Gildea, R. J.; Howard, J. A. K.; Puschmann, H. *J. Appl. Cryst.* **2009**, *42*, 339.

[19] Han, J.; Wzorek, A.; Klika, K. D.; Soloshonok, V. A. *Molecules* **2021**, *26*, 2757.

10. HPLC-Data

4-methyl-*N*-(4-methylbenzyl)benzenesulfinamide (1)

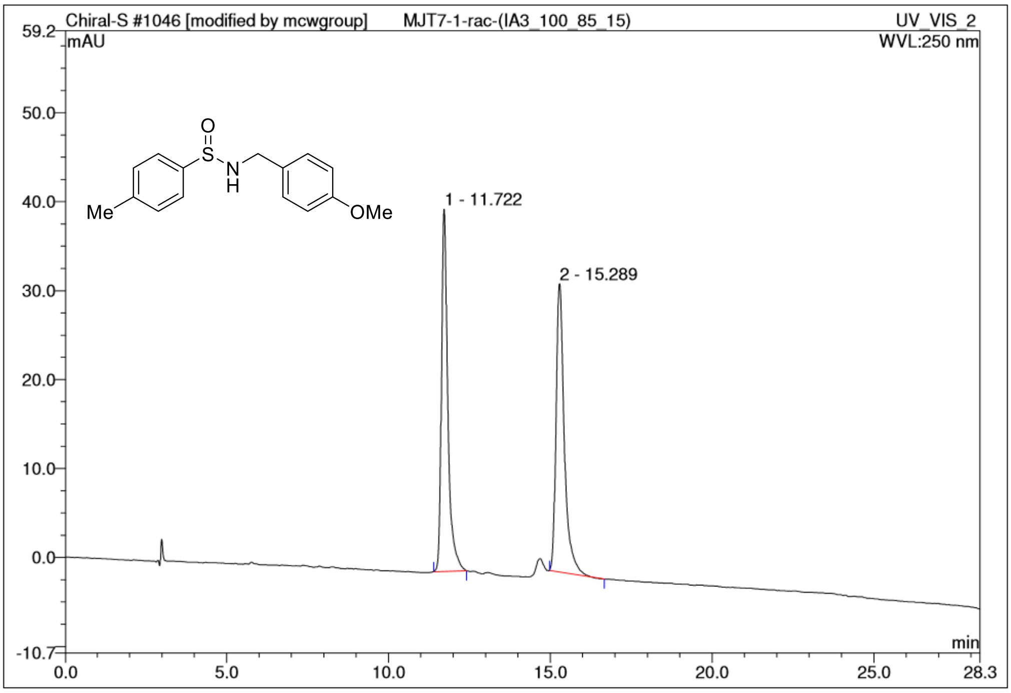

No.	Ret.Time min	Peak Name	Height mAU	Area mAU*min	Rel.Area %	Amount	Type
1	11.72	n.a.	40.752	9.300	49.36	n.a.	BMB*
2	15.29	n.a.	32.415	9.543	50.64	n.a.	BMB*
Total:			73.167	18.843	100.00	0.000	

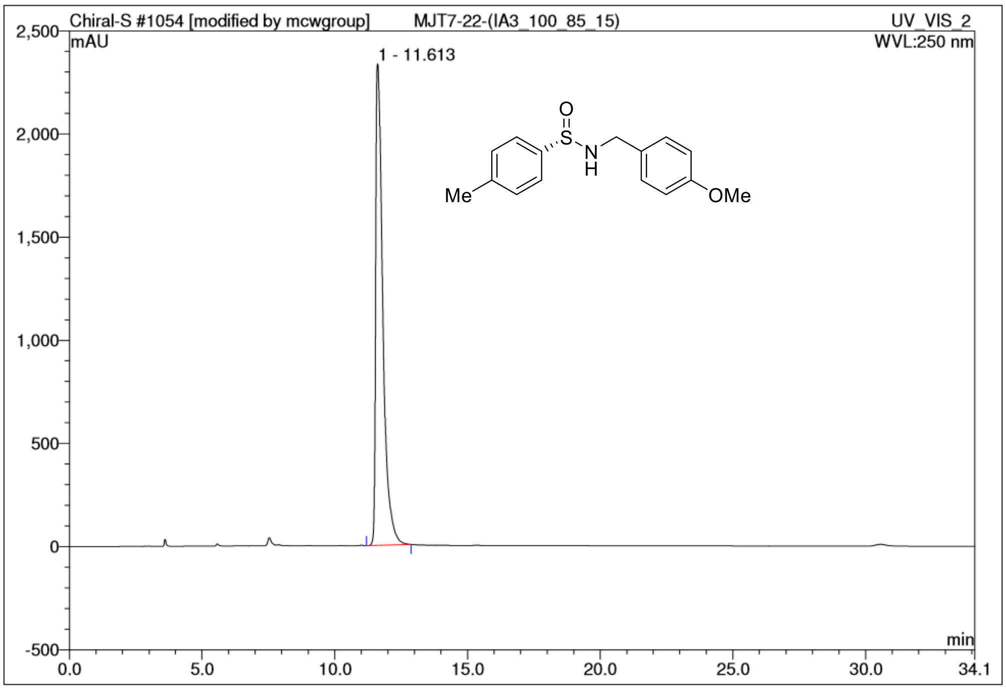

No.	Ret.Time min	Peak Name	Height mAU	Area mAU*min	Rel.Area %	Amount	Type
1	11.61	n.a.	2333.485	733.057	100.00	n.a.	BMB*
Total:			2333.485	733.057	100.00	0.000	

N-benzyl-N,N'-bis(4-methoxybenzyl)-[1,1'-biphenyl]-4-sulfonimidamide (3aa)

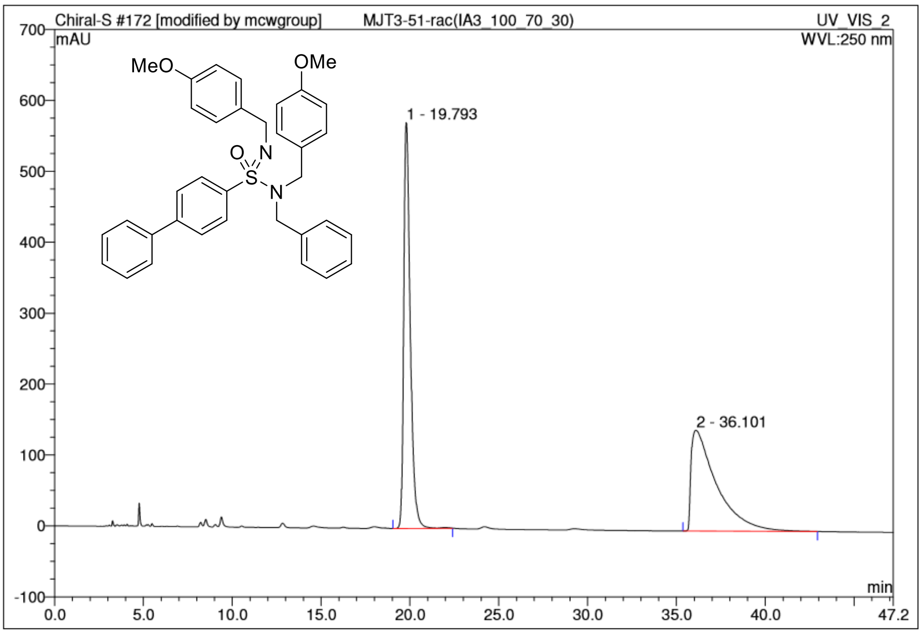

No.	Ret.Time min	Peak Name	Height mAU	Area mAU*min	Rel.Area %	Amount	Type
1	19.79	n.a.	572.290	243.177	50.76	n.a.	BMB*
2	36.10	n.a.	141.996	235.876	49.24	n.a.	BMB*
Total:			714.286	479.053	100.00	0.000	

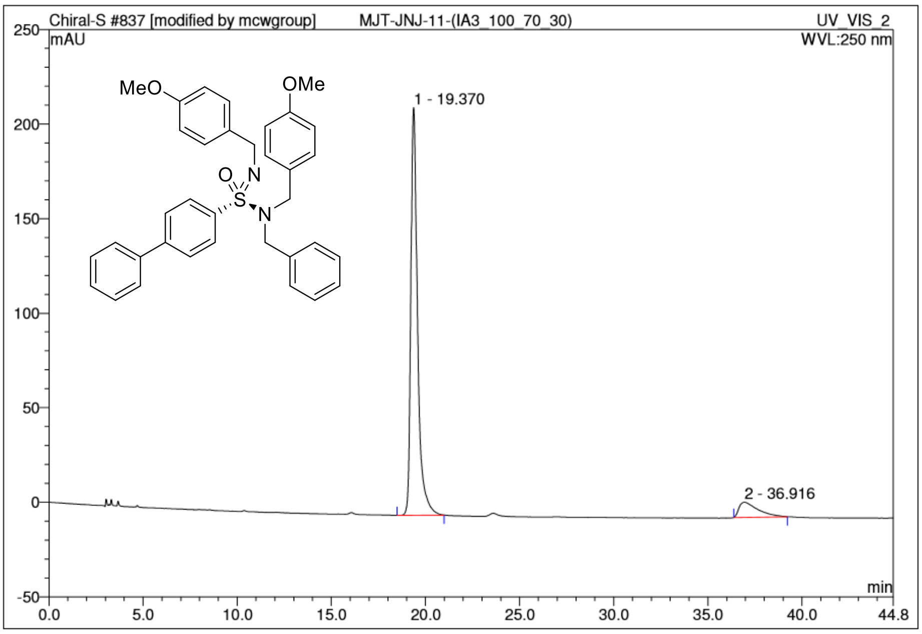

No.	Ret.Time min	Peak Name	Height mAU	Area mAU*min	Rel.Area %	Amount	Type
1	19.37	n.a.	215.670	89.525	90.54	n.a.	BMB*
2	36.92	n.a.	7.975	9.356	9.46	n.a.	BMB*
Total:			223.645	98.881	100.00	0.000	

***N*-(4-fluorobenzyl)-*N*,*N'*-bis(4-methoxybenzyl)-[1,1'-biphenyl]-4-sulfonimidamide (3ab)**

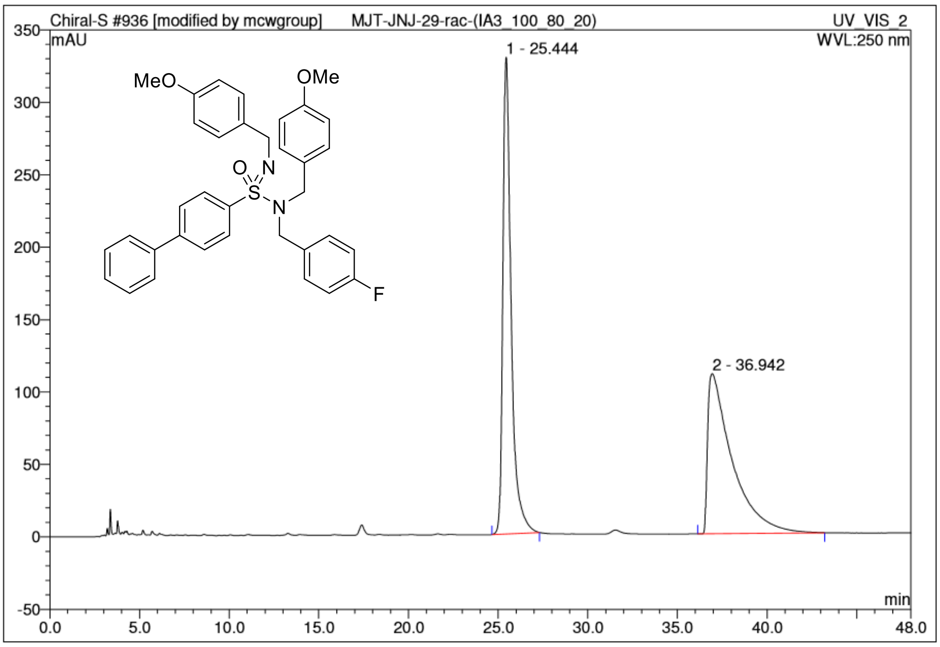

No.	Ret.Time min	Peak Name	Height mAU	Area mAU*min	Rel.Area %	Amount	Type
1	25.44	n.a.	329.122	176.327	50.29	n.a.	BMB*
2	36.94	n.a.	110.482	174.317	49.71	n.a.	BMB*
Total:			439.604	350.644	100.00	0.000	

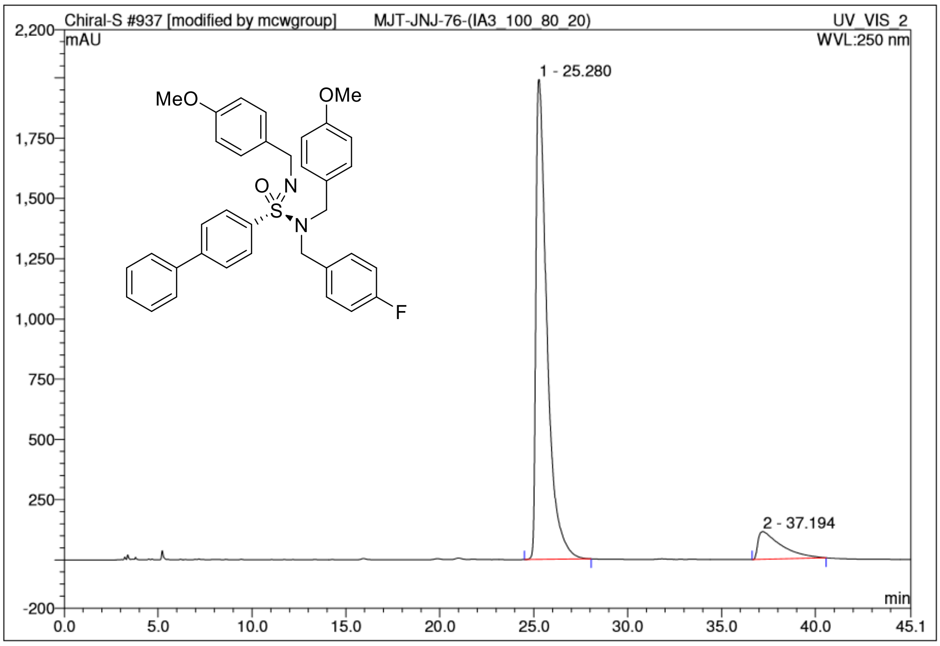

No.	Ret.Time min	Peak Name	Height mAU	Area mAU*min	Rel.Area %	Amount	Type
1	25.28	n.a.	1991.140	1357.768	89.15	n.a.	BMB*
2	37.19	n.a.	114.795	165.245	10.85	n.a.	BMB*
Total:			2105.934	1523.014	100.00	0.000	

***N*,*N'*-bis(4-methoxybenzyl)-N-(4-(methylthio)benzyl)-[1,1'-biphenyl]-4-sulfonimidamide (3ac)**

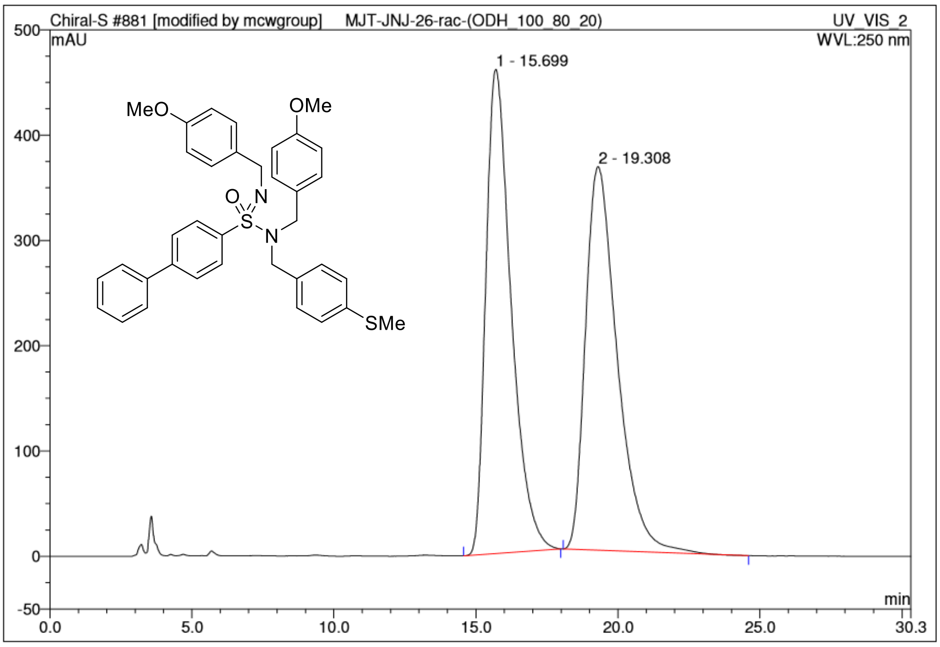

No.	Ret.Time min	Peak Name	Height mAU	Area mAU*min	Rel.Area %	Amount	Type
1	15.70	n.a.	459.736	474.044	50.08	n.a.	BMB*
2	19.31	n.a.	364.278	472.583	49.92	n.a.	BMB*
Total:			824.014	946.627	100.00	0.000	

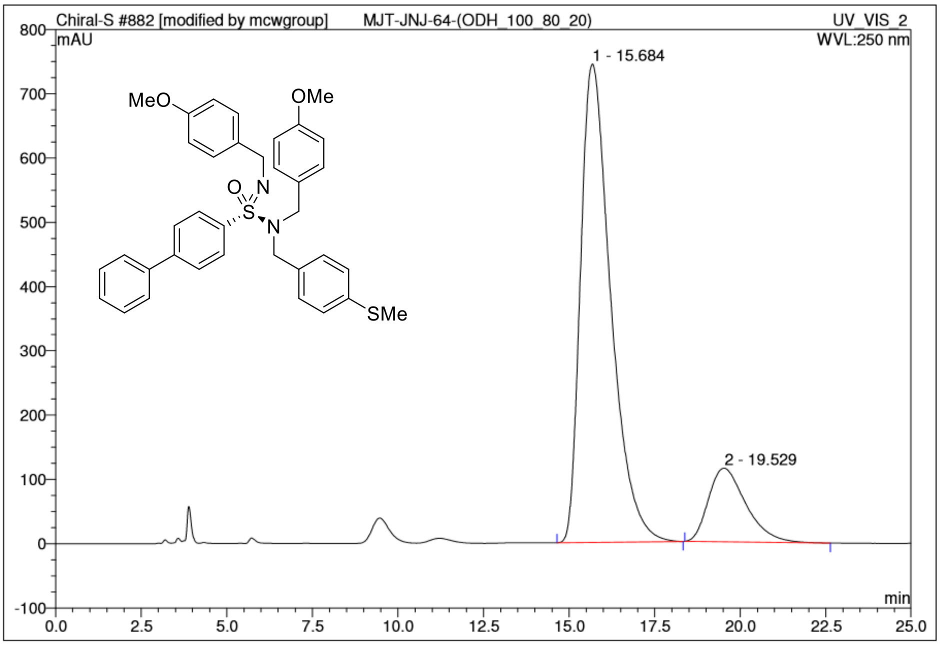

No.	Ret.Time min	Peak Name	Height mAU	Area mAU*min	Rel.Area %	Amount	Type
1	15.68	n.a.	744.251	759.847	83.96	n.a.	BMB*
2	19.53	n.a.	114.489	145.178	16.04	n.a.	BMB*
Total:			858.740	905.025	100.00	0.000	

N-(4-cyanobenzyl)-*N*,*N'*-bis(4-methoxybenzyl)-[1,1'-biphenyl]-4-sulfonimidamide (3ad)

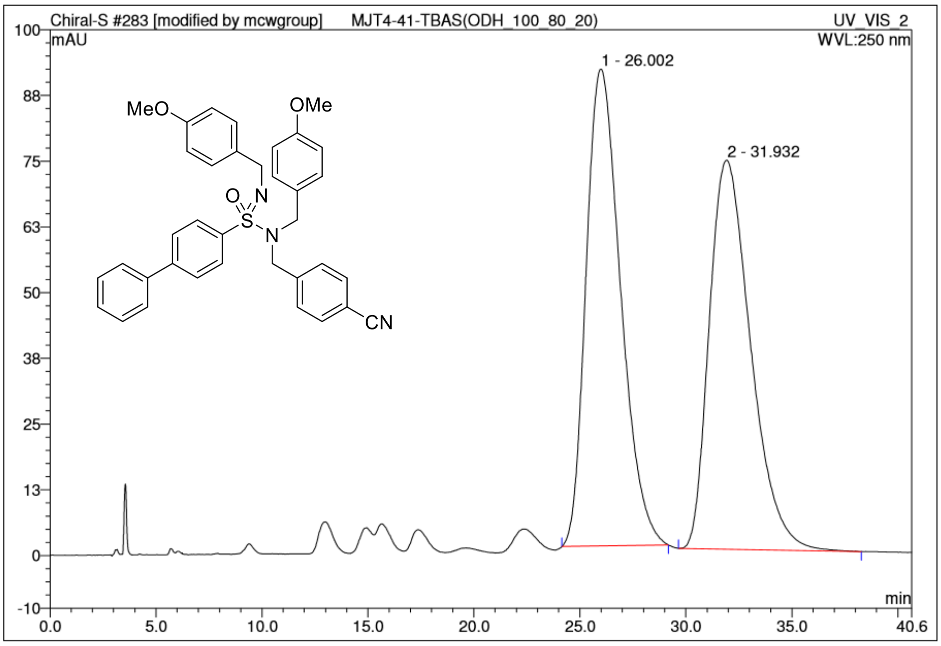

No.	Ret.Time min	Peak Name	Height mAU	Area mAU*min	Rel.Area %	Amount	Type
1	26.00	n.a.	90.675	170.835	50.54	n.a.	BMB*
2	31.93	n.a.	74.005	167.171	49.46	n.a.	BMB*
Total:			164.681	338.006	100.00	0.000	

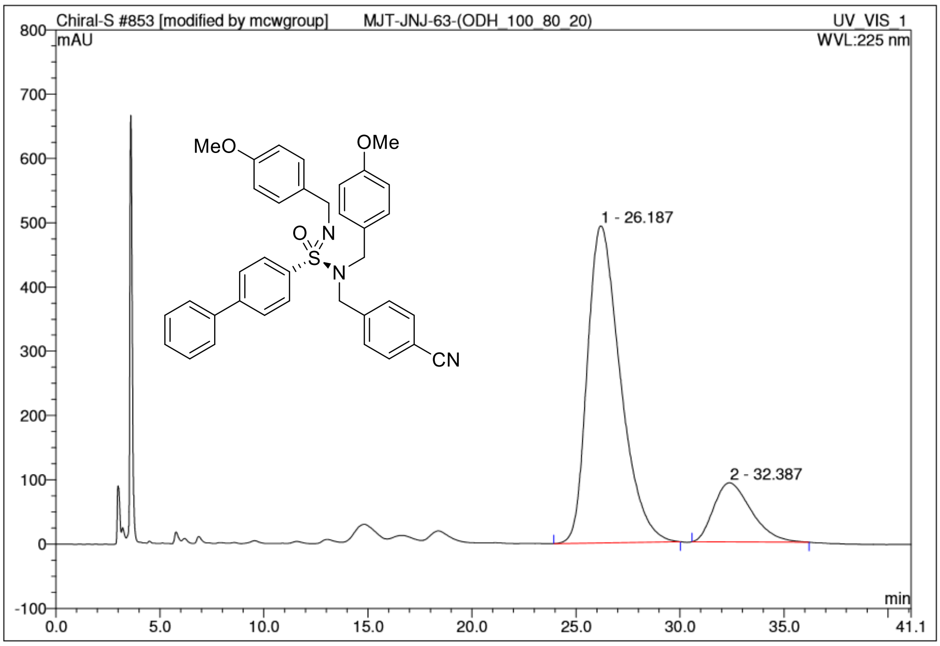

No.	Ret.Time min	Peak Name	Height mAU	Area mAU*min	Rel.Area %	Amount	Type
1	26.19	n.a.	493.187	908.533	82.53	n.a.	BMB*
2	32.39	n.a.	91.877	192.265	17.47	n.a.	BMB*
Total:			585.065	1100.798	100.00	0.000	

N-(3-formylbenzyl)-N,N'-bis(4-methoxybenzyl)-[1,1'-biphenyl]-4-sulfonimidamide (3ae)

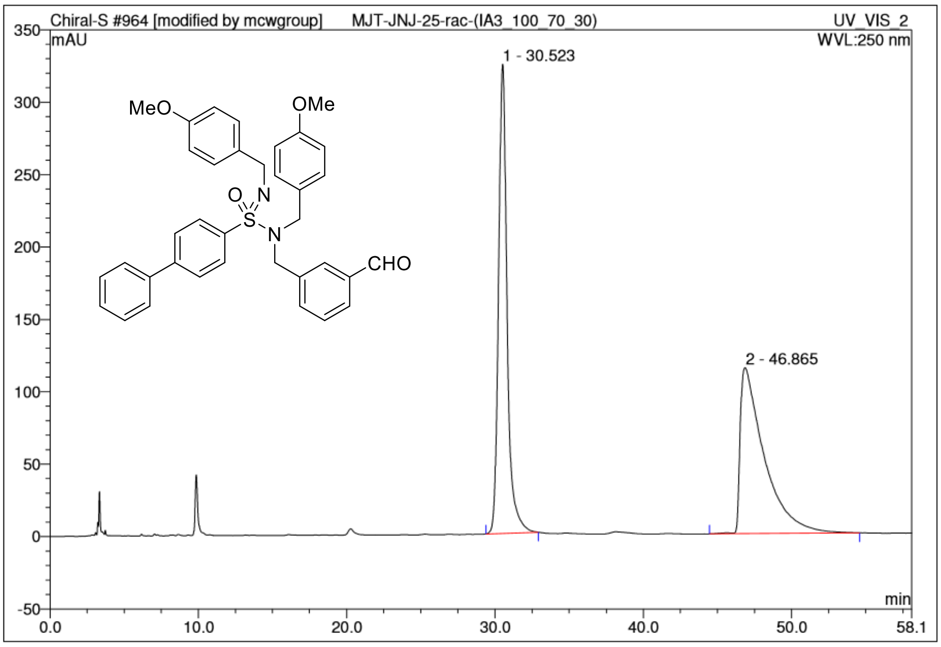

No.	Ret.Time min	Peak Name	Height mAU	Area mAU*min	Rel.Area %	Amount	Type
1	30.52	n.a.	324.102	216.727	50.19	n.a.	BMB*
2	46.87	n.a.	114.588	215.109	49.81	n.a.	BMB*
Total:			438.690	431.836	100.00	0.000	

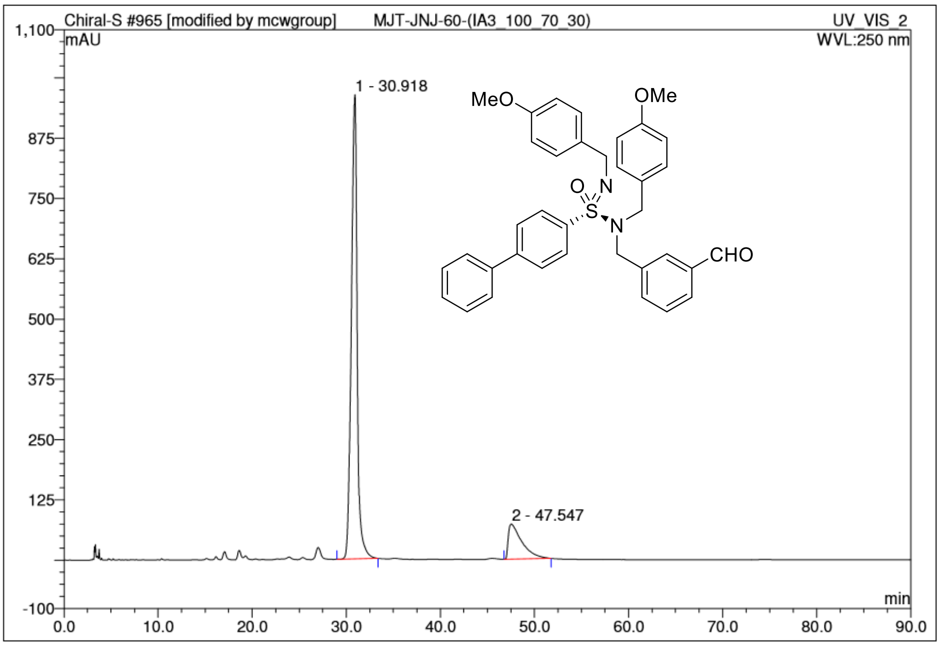

No.	Ret.Time min	Peak Name	Height mAU	Area mAU*min	Rel.Area %	Amount	Type
1	30.92	n.a.	962.843	689.726	84.98	n.a.	BMB*
2	47.55	n.a.	73.020	121.881	15.02	n.a.	BMB*
Total:			1035.863	811.607	100.00	0.000	

N,N'-bis(4-methoxybenzyl)-N-(naphthalen-2-ylmethyl)-[1,1'-biphenyl]-4-sulfonimidamide (3af)

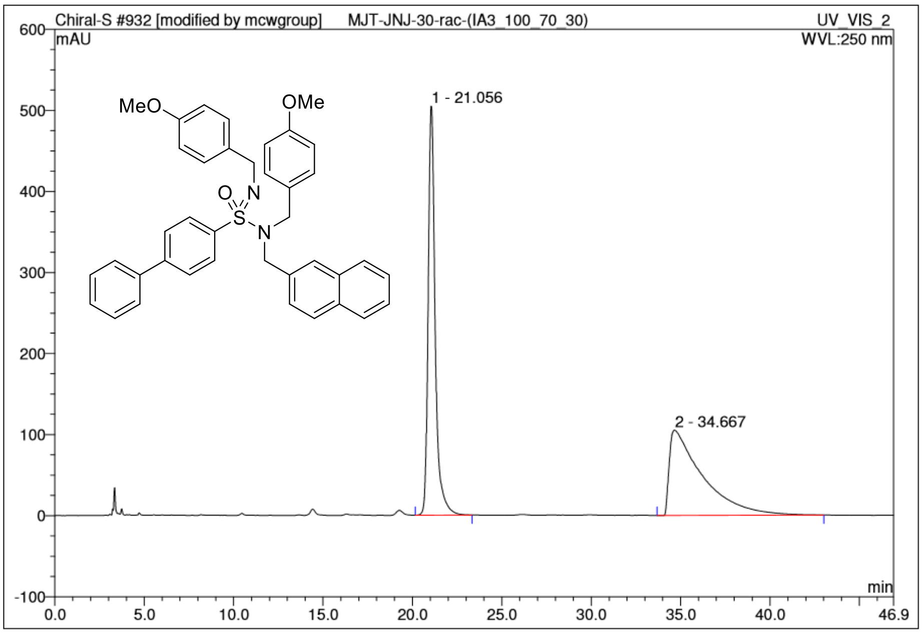

No.	Ret.Time min	Peak Name	Height mAU	Area mAU*min	Rel.Area %	Amount	Type
1	21.06	n.a.	504.827	229.397	50.39	n.a.	BMB*
2	34.67	n.a.	105.147	225.875	49.61	n.a.	BMB*
Total:			609.974	455.273	100.00	0.000	

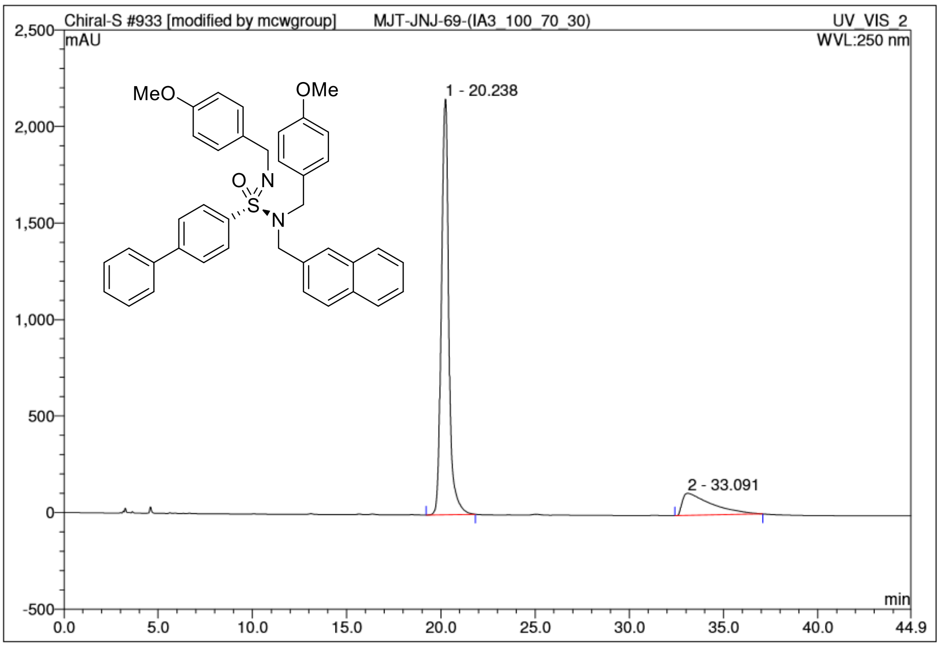

No.	Ret.Time min	Peak Name	Height mAU	Area mAU*min	Rel.Area %	Amount	Type
1	20.24	n.a.	2153.249	979.978	82.65	n.a.	BMB*
2	33.09	n.a.	114.338	205.728	17.35	n.a.	BMB*
Total:			2267.587	1185.707	100.00	0.000	

_N_-allyl-_N_,_N'_-bis(4-methoxybenzyl)-[1,1'-biphenyl]-4-sulfonimidamidede (3ag)

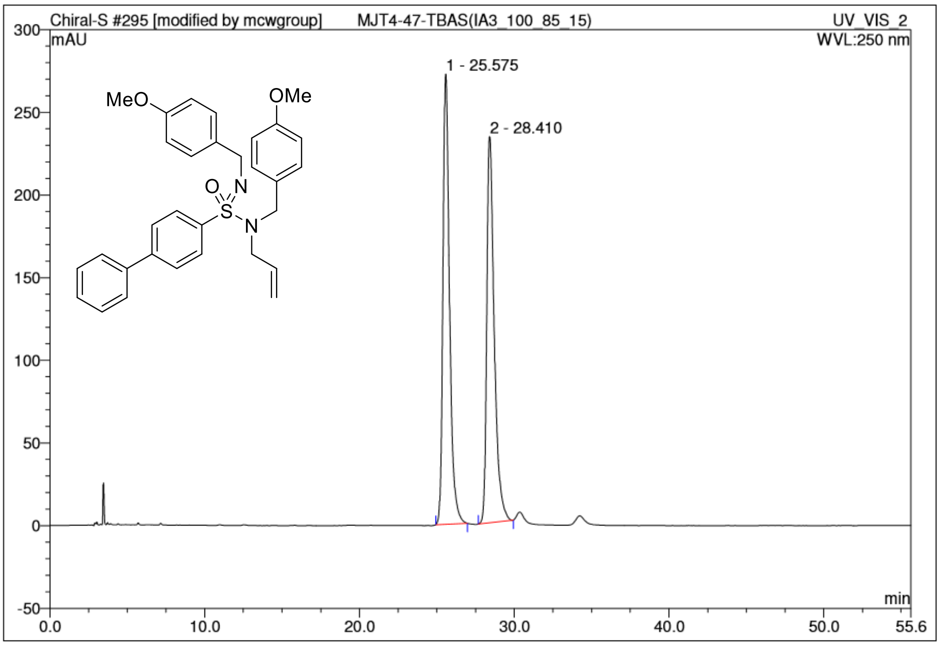

No.	Ret.Time min	Peak Name	Height mAU	Area mAU*min	Rel.Area %	Amount	Type
1	25.58	n.a.	272.347	134.489	50.73	n.a.	BMB*
2	28.41	n.a.	233.636	130.644	49.27	n.a.	BMB*
Total:			505.982	265.134	100.00	0.000	

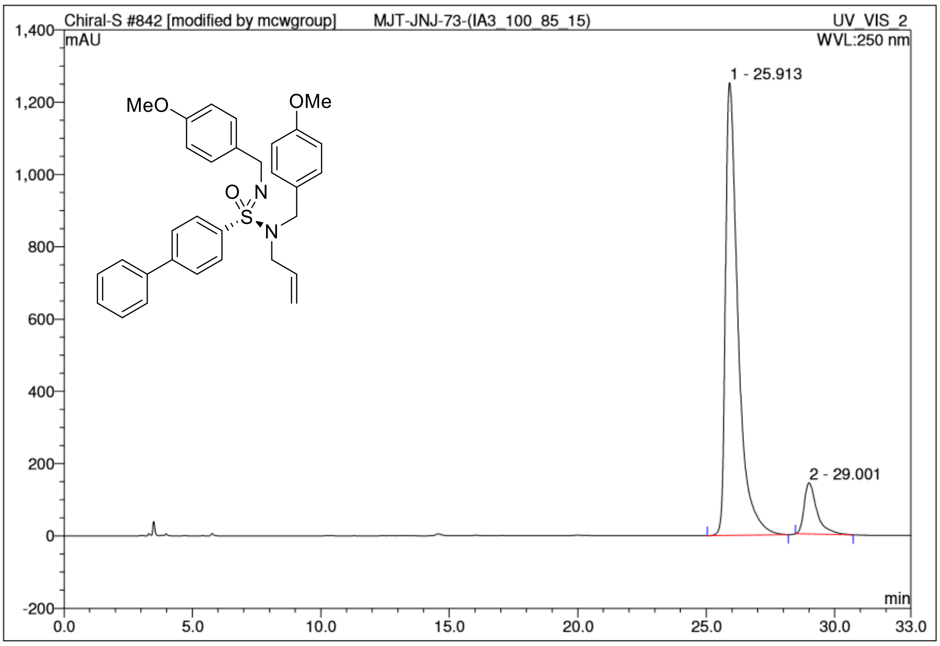

No.	Ret.Time min	Peak Name	Height mAU	Area mAU*min	Rel.Area %	Amount	Type
1	25.91	n.a.	1252.286	707.992	89.65	n.a.	BMB*
2	29.00	n.a.	141.525	81.756	10.35	n.a.	BMB*
Total:			1393.810	789.748	100.00	0.000	

N,*N'*-bis(4-methoxybenzyl)-*N*-(2-methylallyl)-[1,1'-biphenyl]-4-sulfonimidamide (3ah)

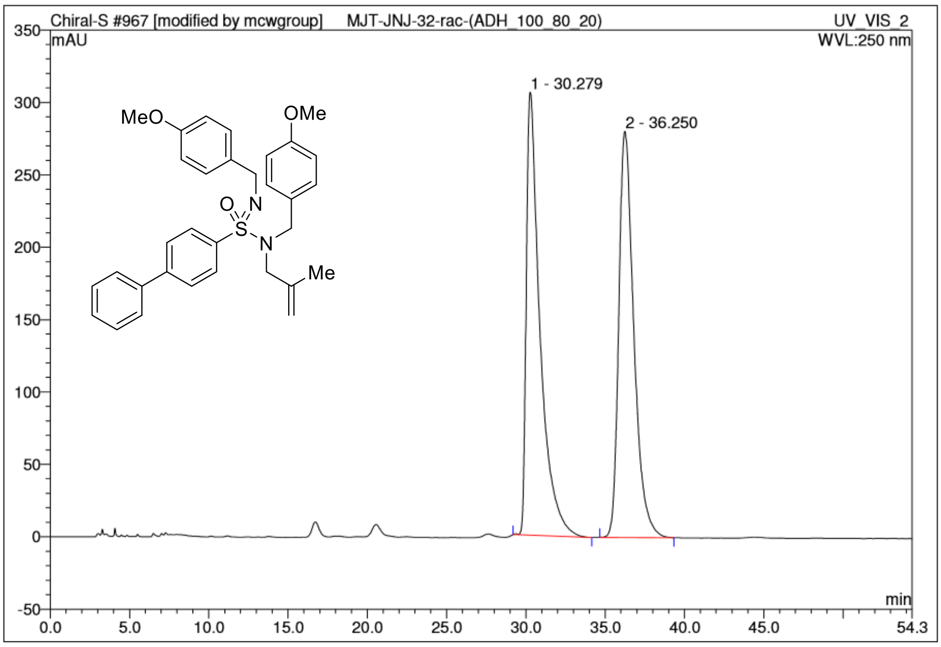

No.	Ret.Time min	Peak Name	Height mAU	Area mAU*min	Rel.Area %	Amount	Type
1	30.28	n.a.	305.982	305.429	49.79	n.a.	BMB*
2	36.25	n.a.	280.572	308.014	50.21	n.a.	BMB*
Total:			586.554	613.443	100.00	0.000	

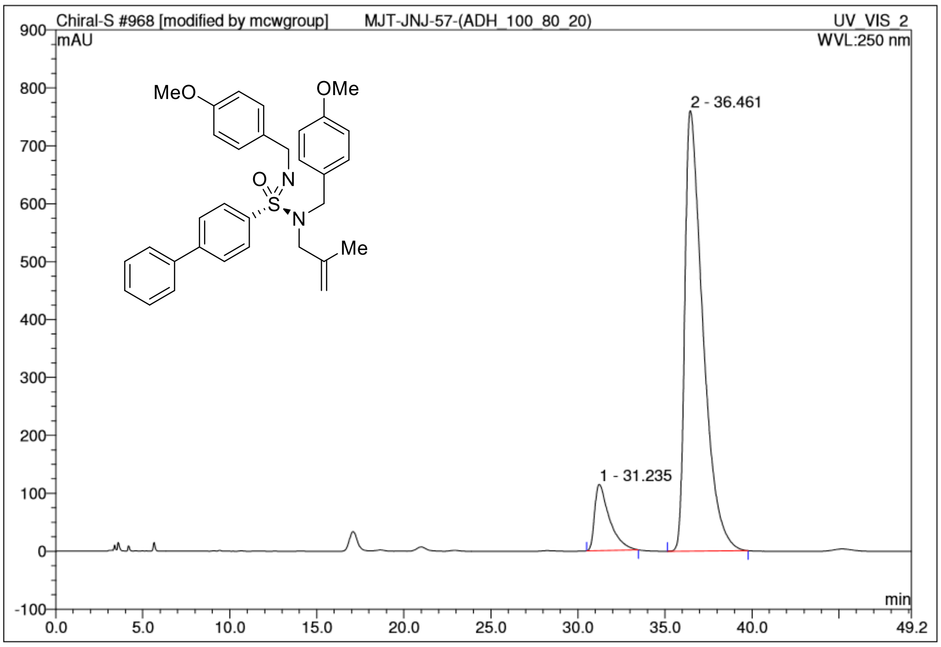

No.	Ret.Time min	Peak Name	Height mAU	Area mAU*min	Rel.Area %	Amount	Type
1	31.24	n.a.	114.388	112.602	10.96	n.a.	BMB*
2	36.46	n.a.	760.676	914.906	89.04	n.a.	BMB*
Total:			875.063	1027.508	100.00	0.000	

N-(cyclopropylmethyl)-N,N'-bis(4-methoxybenzyl)-[1,1'-biphenyl]-4-sulfonimidamide (3ai)

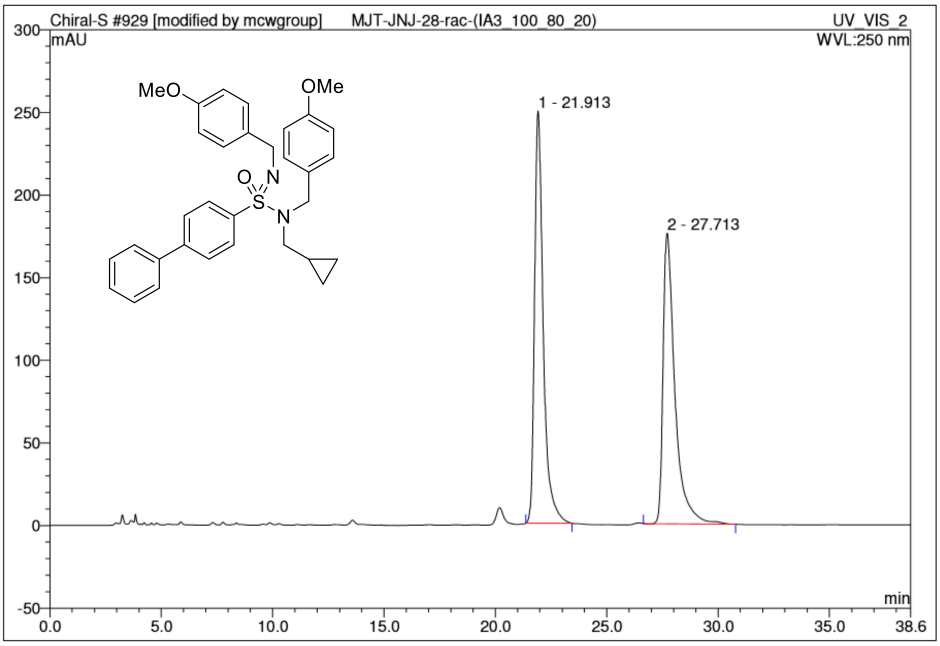

No.	Ret.Time min	Peak Name	Height mAU	Area mAU*min	Rel.Area %	Amount	Type
1	21.91	n.a.	249.459	112.649	50.98	n.a.	BMB*
2	27.71	n.a.	175.858	108.301	49.02	n.a.	BMB*
Total:			425.317	220.951	100.00	0.000	

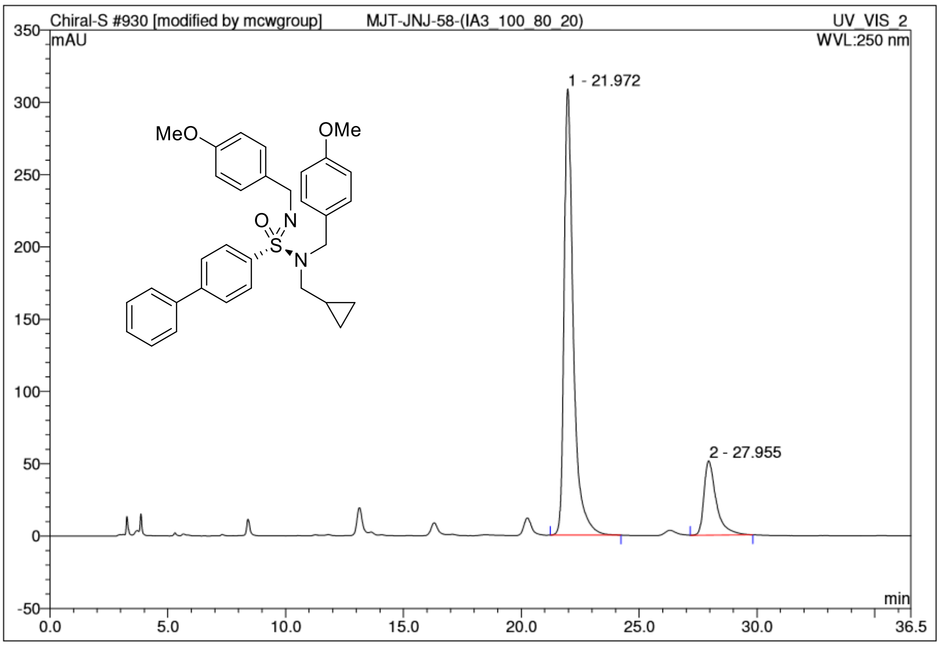

No.	Ret.Time min	Peak Name	Height mAU	Area mAU*min	Rel.Area %	Amount	Type
1	21.97	n.a.	308.450	140.247	81.90	n.a.	BMB*
2	27.95	n.a.	51.322	30.994	18.10	n.a.	BMB*
Total:			359.773	171.241	100.00	0.000	

N-benzyl-*N*,*N'*-bis(4-methoxybenzyl)-4-methylbenzenesulfonimidamide (3b)

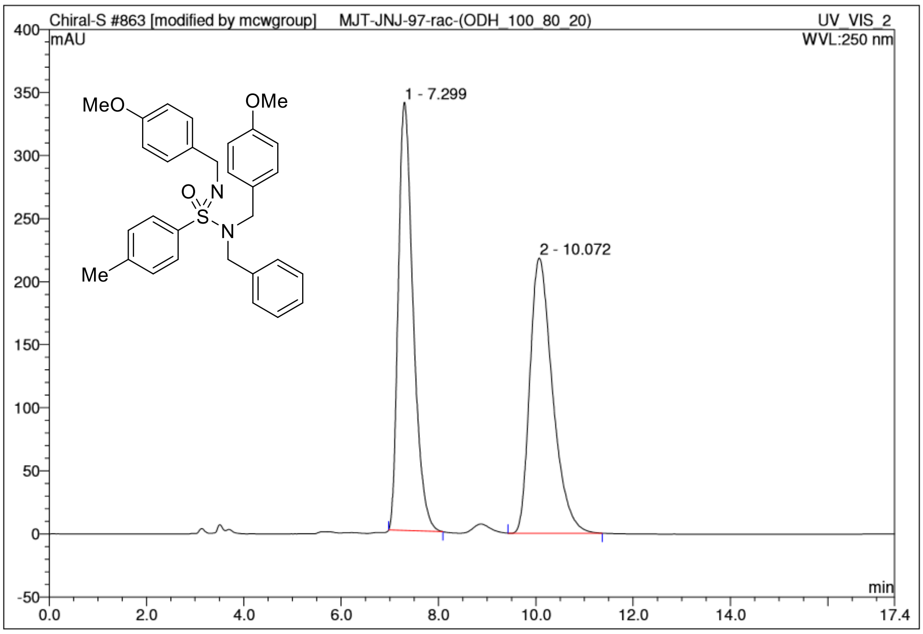

No.	Ret.Time min	Peak Name	Height mAU	Area mAU*min	Rel.Area %	Amount	Type
1	7.30	n.a.	339.601	119.879	50.40	n.a.	BMB*
2	10.07	n.a.	218.286	117.970	49.60	n.a.	BMB*
Total:			557.887	237.849	100.00	0.000	

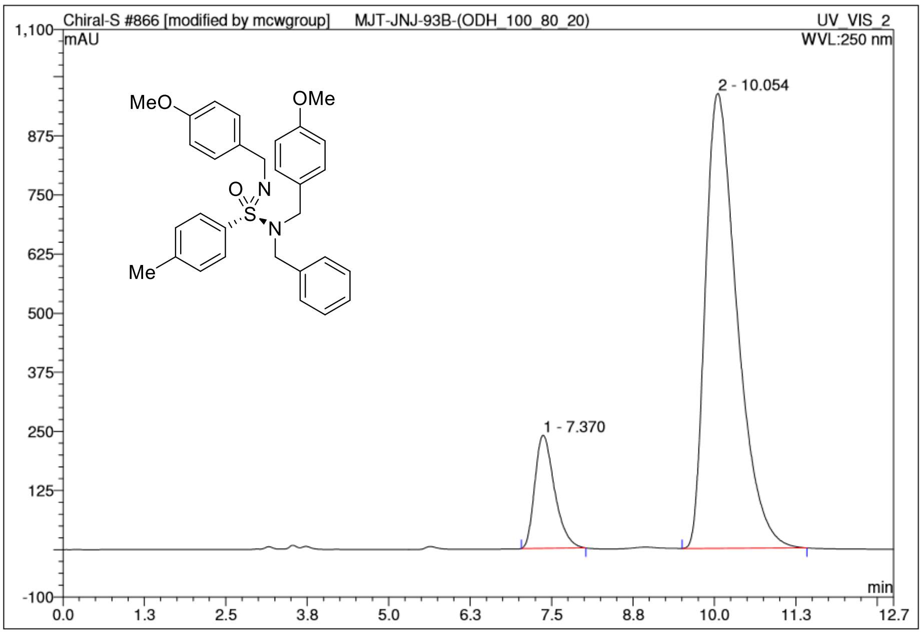

No.	Ret.Time min	Peak Name	Height mAU	Area mAU*min	Rel.Area %	Amount	Type
1	7.37	n.a.	238.990	84.136	13.44	n.a.	BMB*
2	10.05	n.a.	961.662	541.867	86.56	n.a.	BMB*
Total:			1200.652	626.003	100.00	0.000	

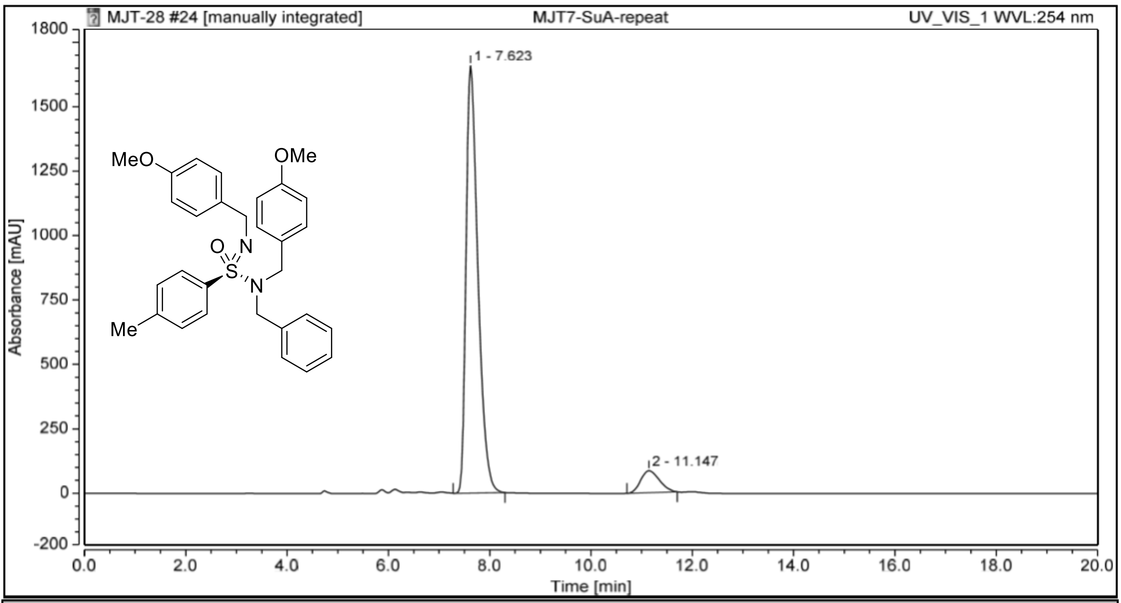

Integration Results

No.	Peak Name	Retention Time<br>min	Area<br>mAU*min	Height<br>mAU	Relative Area<br>%
1		7.623	450.911	1658.573	92.53
2		11.147	36.397	85.003	7.47
**Total:**			**487.309**	**1743.576**	**100.00**

N-benzyl-*N*,*N'*-bis(4-methoxybenzyl)benzenesulfonimidamide (3c)

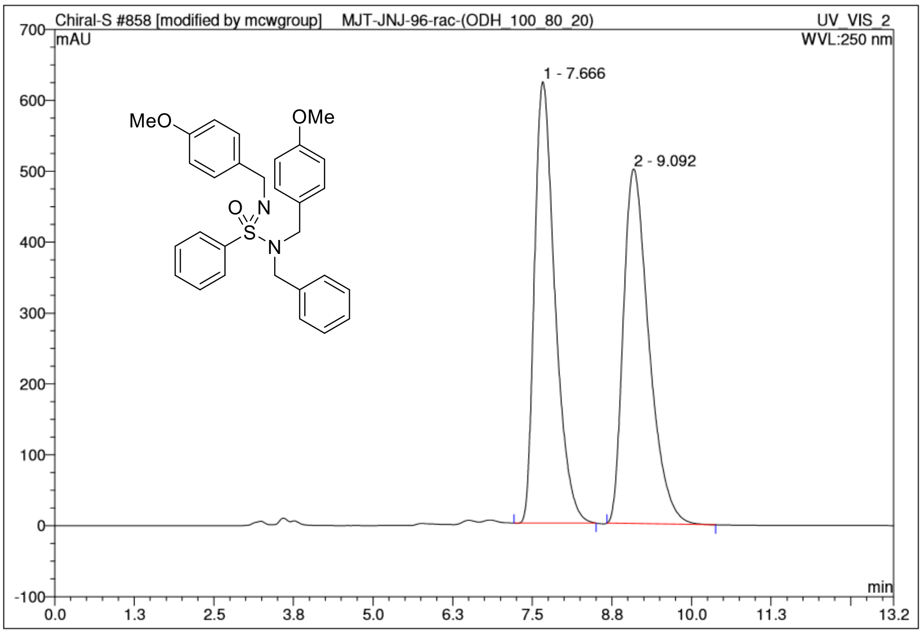

No.	Ret.Time min	Peak Name	Height mAU	Area mAU*min	Rel.Area %	Amount	Type
1	7.67	n.a.	622.473	231.759	49.92	n.a.	BMB*
2	9.09	n.a.	499.903	232.537	50.08	n.a.	BMB*
Total:			1122.375	464.296	100.00	0.000	

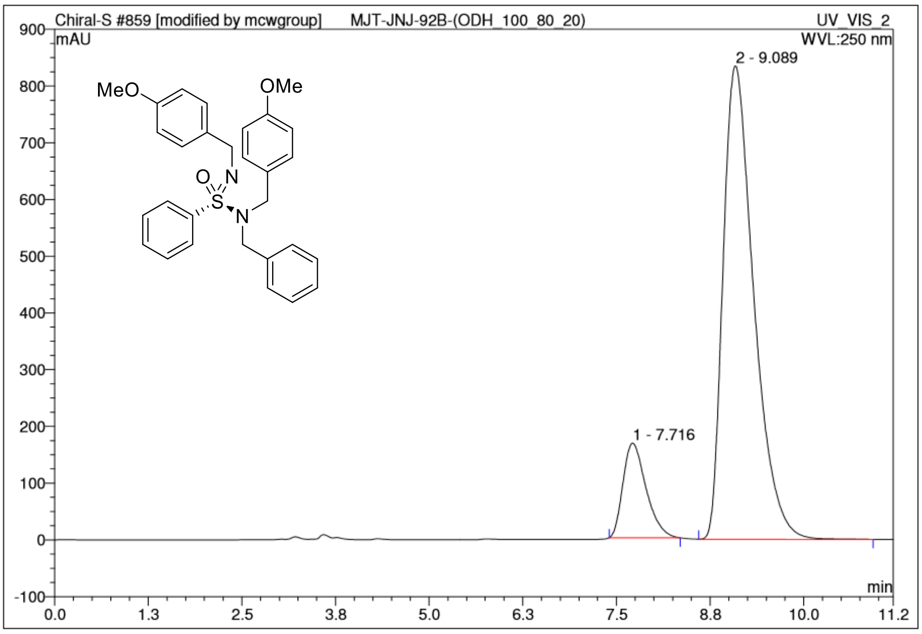

No.	Ret.Time min	Peak Name	Height mAU	Area mAU*min	Rel.Area %	Amount	Type
1	7.72	n.a.	166.751	59.683	13.10	n.a.	BMB*
2	9.09	n.a.	834.213	395.942	86.90	n.a.	BMB*
Total:			1000.964	455.625	100.00	0.000	

***N*-benzyl-*N*,*N'*-bis(4-methoxybenzyl)-3-methylbenzenesulfonimidamide (3d)**

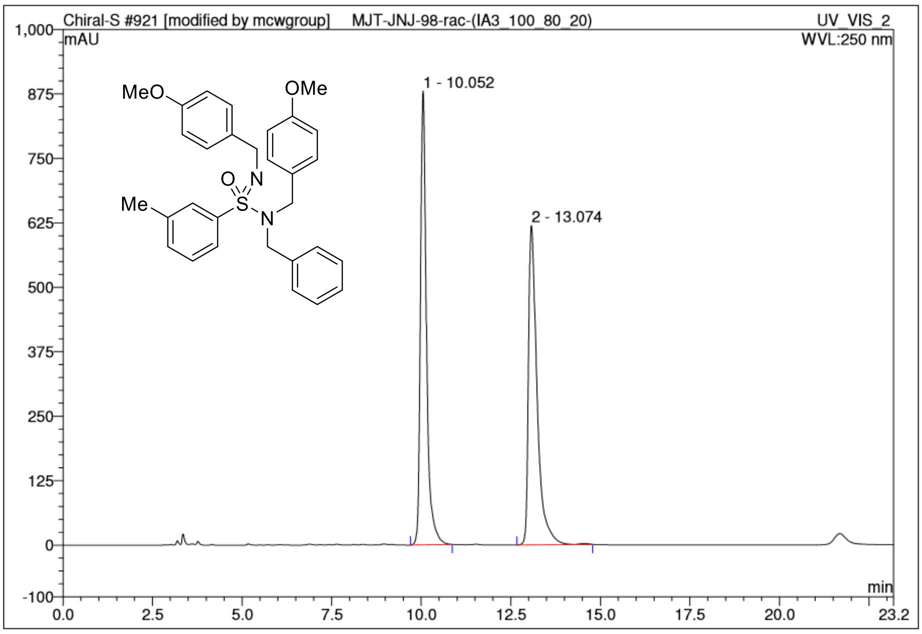

No.	Ret.Time min	Peak Name	Height mAU	Area mAU*min	Rel.Area %	Amount	Type
1	10.05	n.a.	879.899	172.167	49.75	n.a.	BMB*
2	13.07	n.a.	619.476	173.908	50.25	n.a.	BMB*
Total:			1499.375	346.074	100.00	0.000	

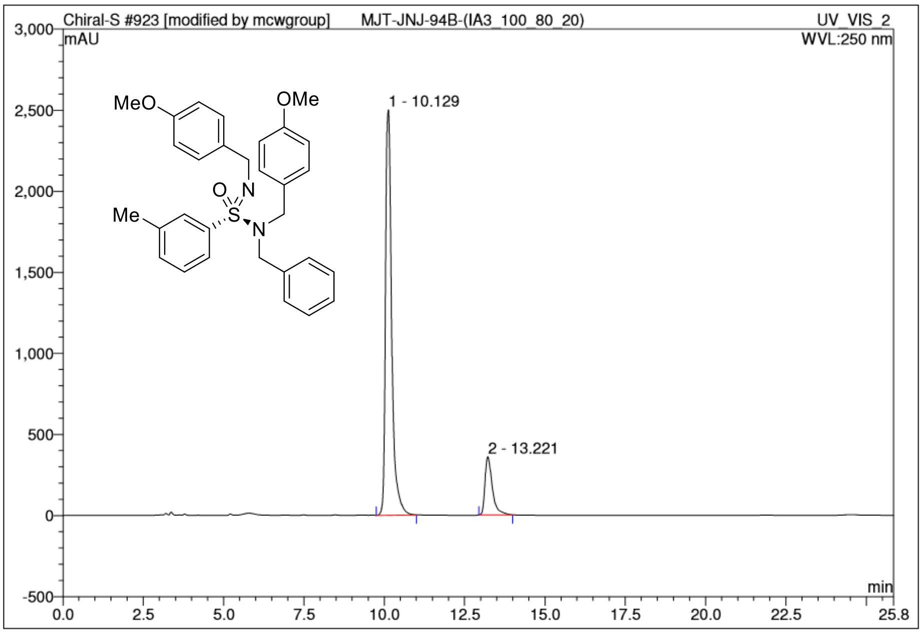

No.	Ret.Time min	Peak Name	Height mAU	Area mAU*min	Rel.Area %	Amount	Type
1	10.13	n.a.	2500.101	558.419	85.49	n.a.	BMB*
2	13.22	n.a.	359.050	94.812	14.51	n.a.	BMB*
Total:			2859.152	653.231	100.00	0.000	

_N_-benzyl-_N_,_N'_-bis(4-methoxybenzyl)-2-methylbenzenesulfonimidamide (3e)

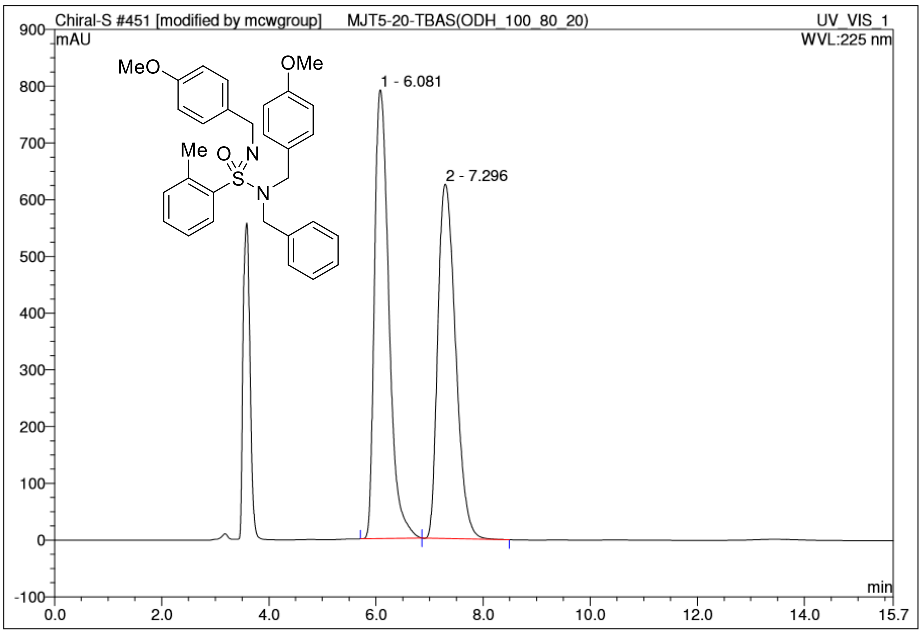

No.	Ret.Time min	Peak Name	Height mAU	Area mAU*min	Rel.Area %	Amount	Type
1	6.08	n.a.	791.043	246.467	50.71	n.a.	BMb*
2	7.30	n.a.	624.559	239.536	49.29	n.a.	bMB*
Total:			1415.602	486.004	100.00	0.000	

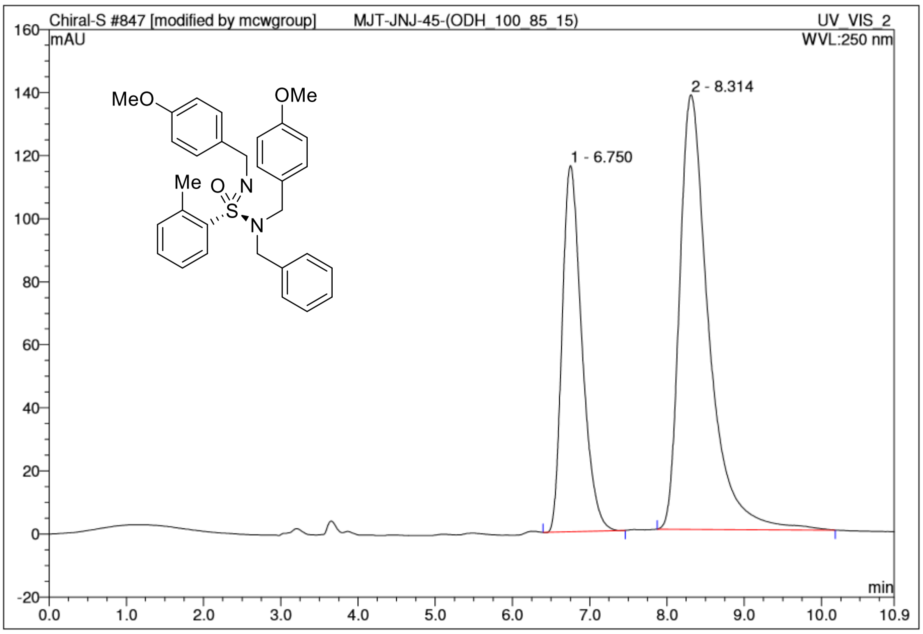

No.	Ret.Time min	Peak Name	Height mAU	Area mAU*min	Rel.Area %	Amount	Type
1	6.75	n.a.	116.161	34.326	36.33	n.a.	BMB*
2	8.31	n.a.	137.800	60.169	63.67	n.a.	BMB*
Total:			253.961	94.495	100.00	0.000	

N-benzyl-4-fluoro-*N*,*N'*-bis(4-methoxybenzyl)benzenesulfonimidamide (3f)

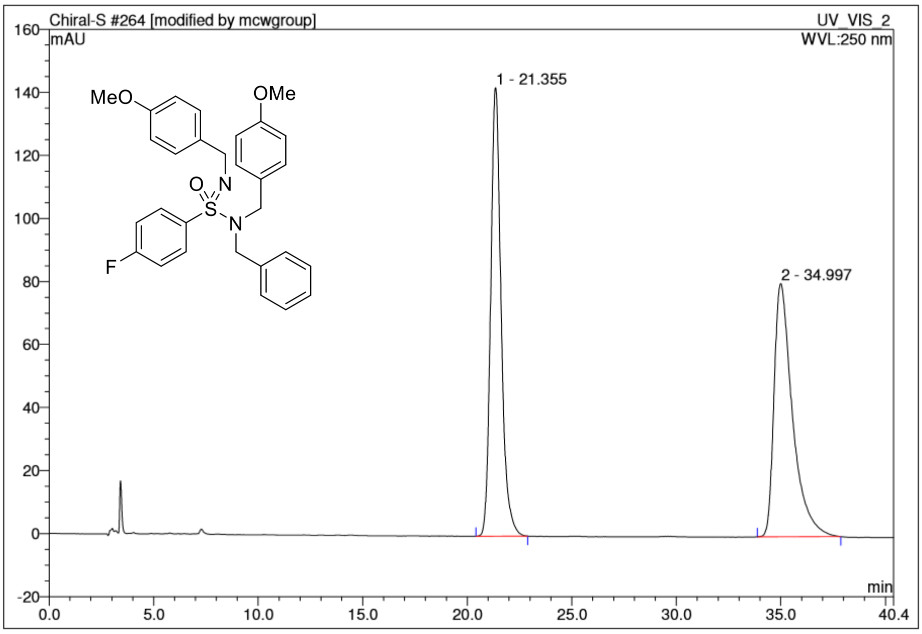

No.	Ret.Time min	Peak Name	Height mAU	Area mAU*min	Rel.Area %	Amount	Type
1	21.35	n.a.	142.353	82.458	50.16	n.a.	BMB*
2	35.00	n.a.	80.373	81.936	49.84	n.a.	BMB*
Total:			222.726	164.394	100.00	0.000	

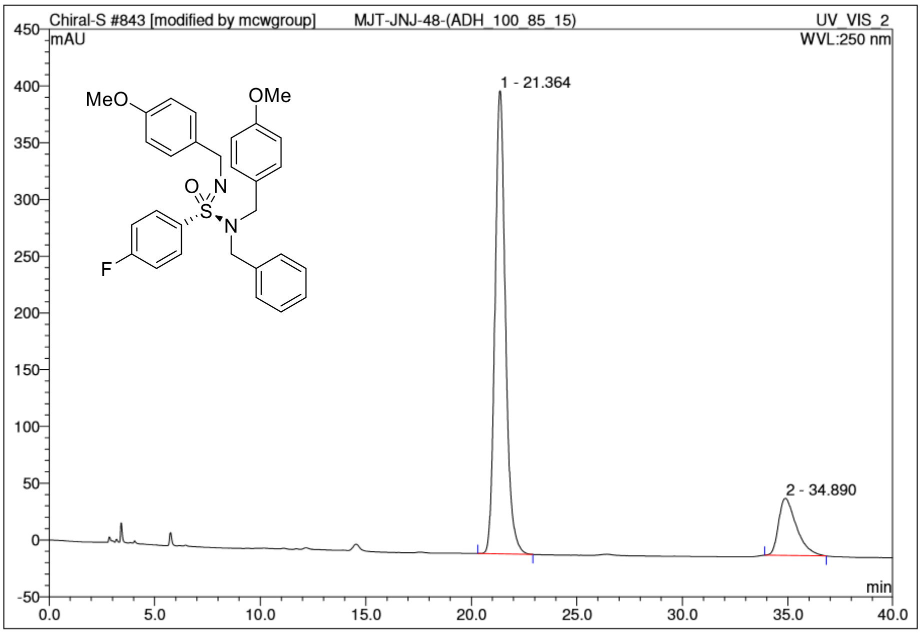

No.	Ret.Time min	Peak Name	Height mAU	Area mAU*min	Rel.Area %	Amount	Type
1	21.36	n.a.	408.063	234.807	82.65	n.a.	BMB*
2	34.89	n.a.	50.189	49.291	17.35	n.a.	BMB*
Total:			458.253	284.098	100.00	0.000	

N-benzyl-*N*,*N'*-bis(4-methoxybenzyl)thiophene-2-sulfonimidamide (3g)

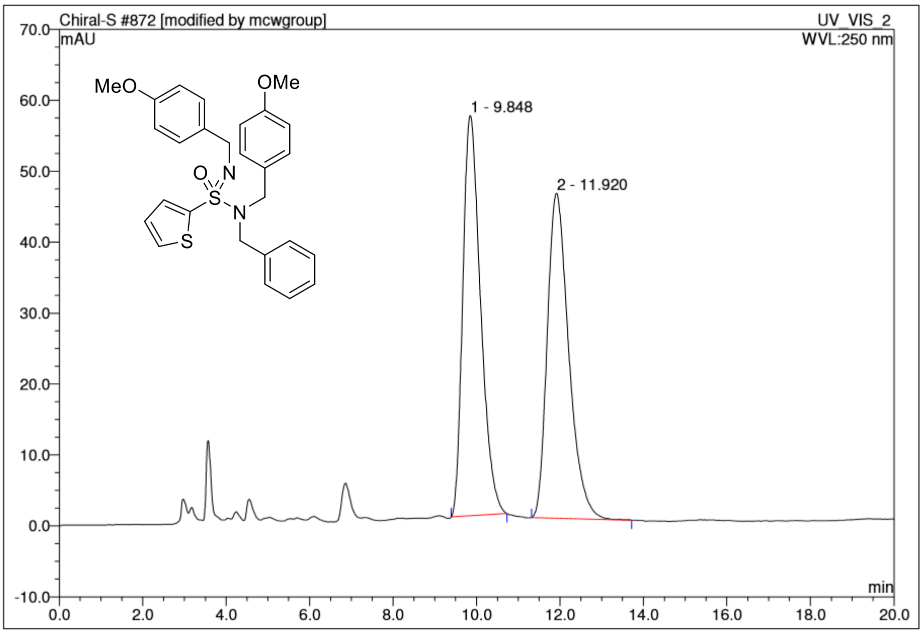

No.	Ret.Time min	Peak Name	Height mAU	Area mAU*min	Rel.Area %	Amount	Type
1	9.85	n.a.	56.407	27.792	50.45	n.a.	BMB*
2	11.92	n.a.	45.814	27.298	49.55	n.a.	BMB*
Total:			102.221	55.090	100.00	0.000	

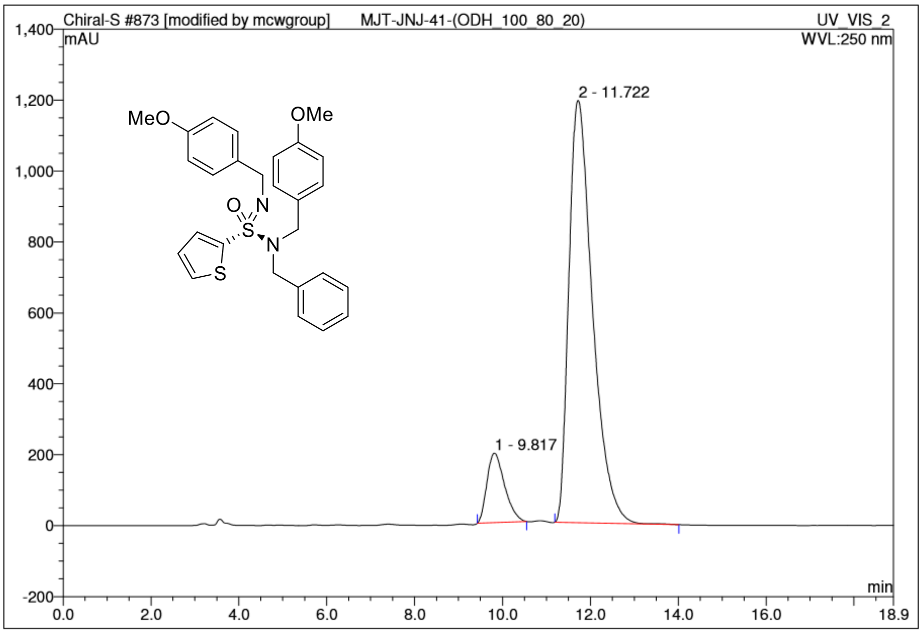

No.	Ret.Time min	Peak Name	Height mAU	Area mAU*min	Rel.Area %	Amount	Type
1	9.82	n.a.	195.506	90.943	10.92	n.a.	BMB*
2	11.72	n.a.	1190.596	741.725	89.08	n.a.	BMB*
Total:			1386.101	832.668	100.00	0.000	

***N*-benzyl-*N*,*N'*-bis(4-methoxybenzyl)-4-(5-(p-tolyl)-3-(trifluoromethyl)-1H-pyrazol-1-yl)benzenesulfonimidamide (3h)**

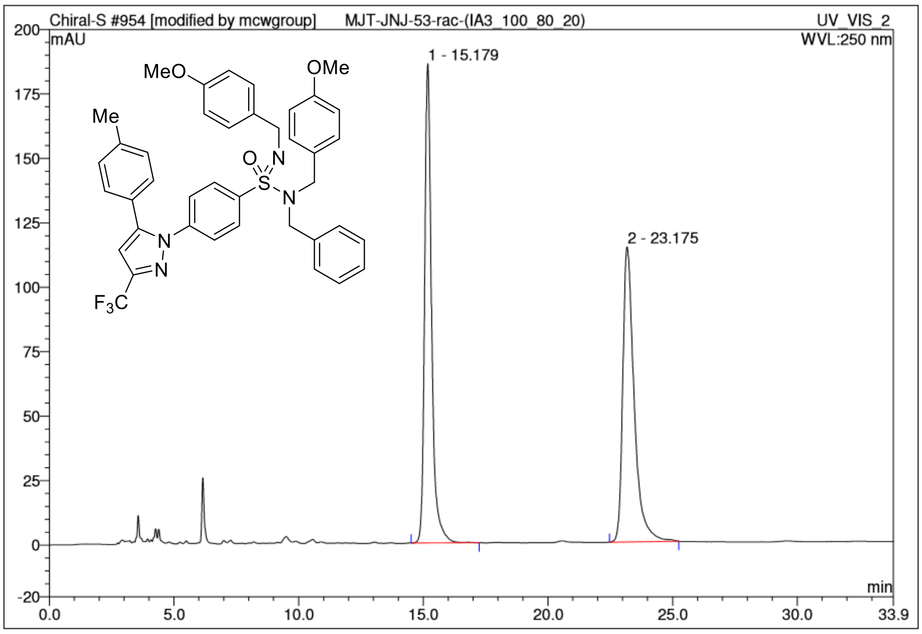

No.	Ret.Time min	Peak Name	Height mAU	Area mAU*min	Rel.Area %	Amount	Type
1	15.18	n.a.	185.951	60.557	49.90	n.a.	BMB*
2	23.18	n.a.	114.516	60.795	50.10	n.a.	BMB*
Total:			300.467	121.352	100.00	0.000	

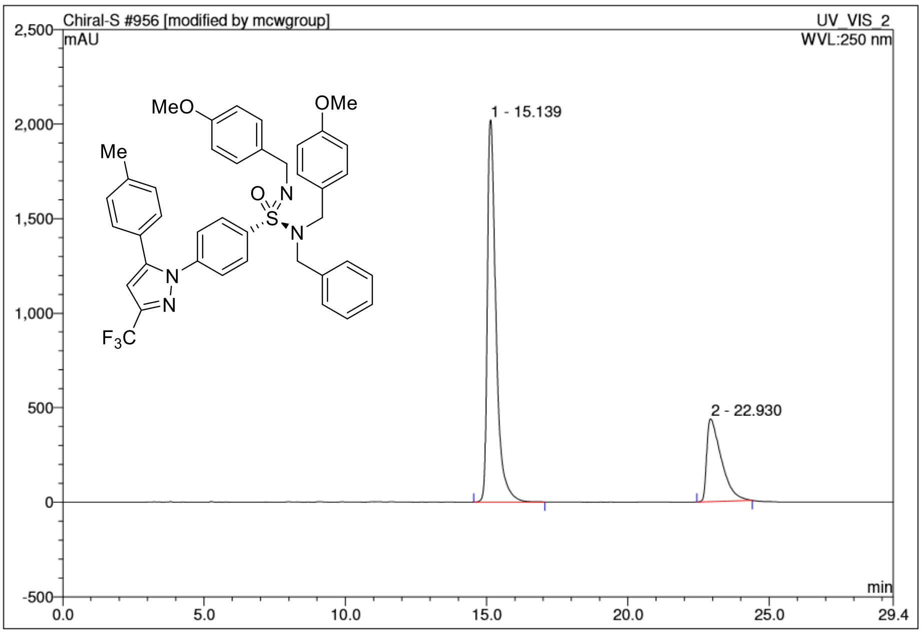

No.	Ret.Time min	Peak Name	Height mAU	Area mAU*min	Rel.Area %	Amount	Type
1	15.14	n.a.	2021.099	716.712	73.20	n.a.	BMB*
2	22.93	n.a.	437.543	262.455	26.80	n.a.	BMB*
Total:			2458.642	979.167	100.00	0.000	

***N*-benzyl-*N*,*N'*-bis(4-methoxybenzyl)-2-methylprop-1-ene-1-sulfonimidamide (3i)**

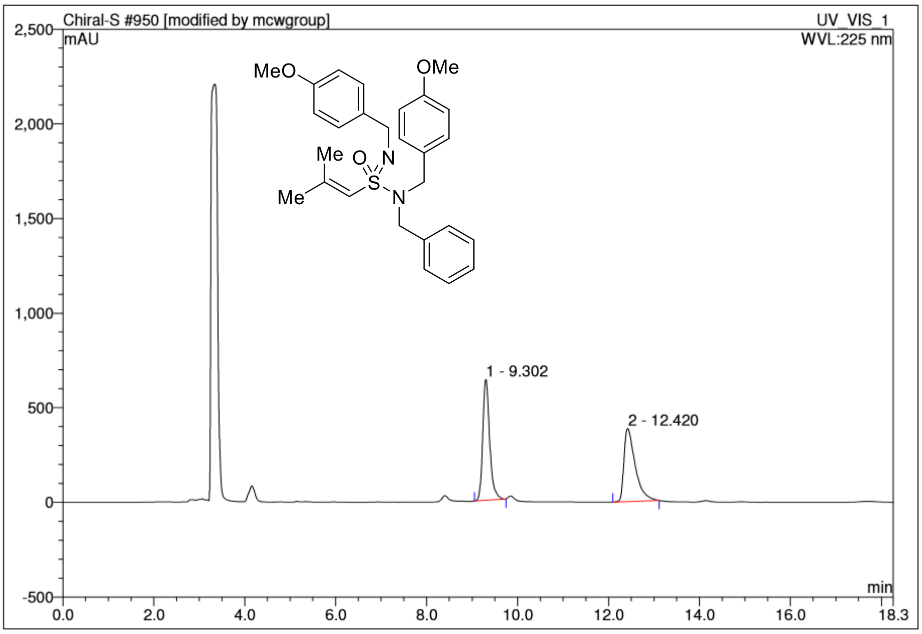

No.	Ret.Time min	Peak Name	Height mAU	Area mAU*min	Rel.Area %	Amount	Type
1	9.30	n.a.	637.643	112.341	50.39	n.a.	BMB*
2	12.42	n.a.	385.143	110.621	49.61	n.a.	BMB*
Total:			1022.786	222.962	100.00	0.000	

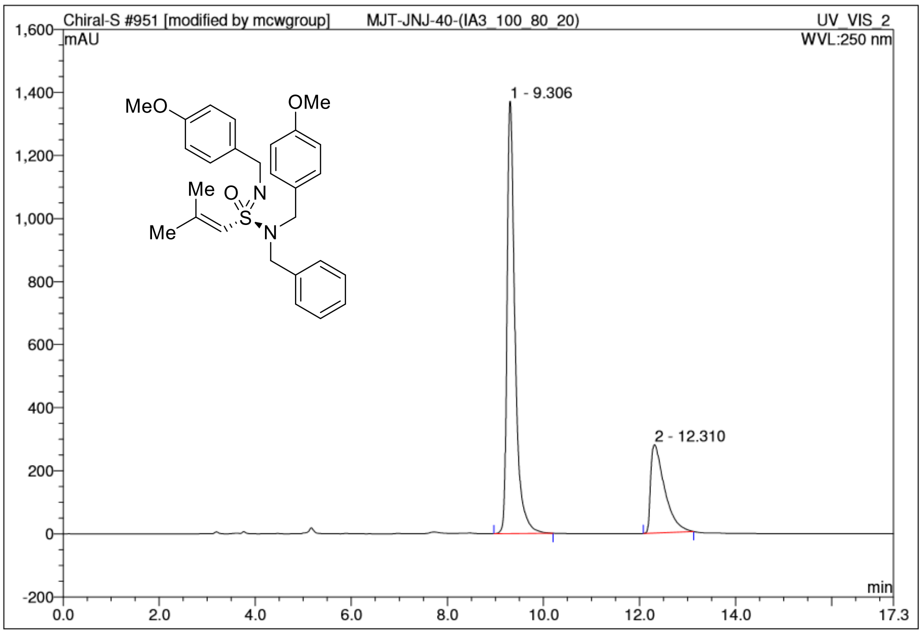

No.	Ret.Time min	Peak Name	Height mAU	Area mAU*min	Rel.Area %	Amount	Type
1	9.31	n.a.	1371.234	262.603	73.56	n.a.	BMB*
2	12.31	n.a.	280.444	94.409	26.44	n.a.	BMB*
Total:			1651.678	357.012	100.00	0.000	

N-benzyl-N,N'-bis(4-methoxybenzyl)methanesulfonimidamide (3j)

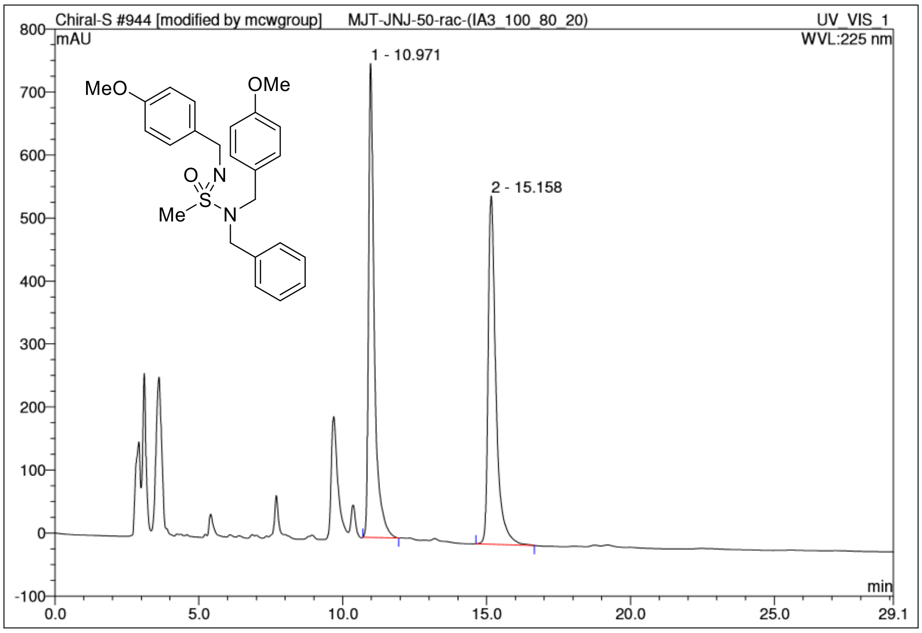

No.	Ret.Time min	Peak Name	Height mAU	Area mAU*min	Rel.Area %	Amount	Type
1	10.97	n.a.	752.505	175.470	50.35	n.a.	BMB*
2	15.16	n.a.	552.989	173.030	49.65	n.a.	BMB*
Total:			1305.494	348.500	100.00	0.000	

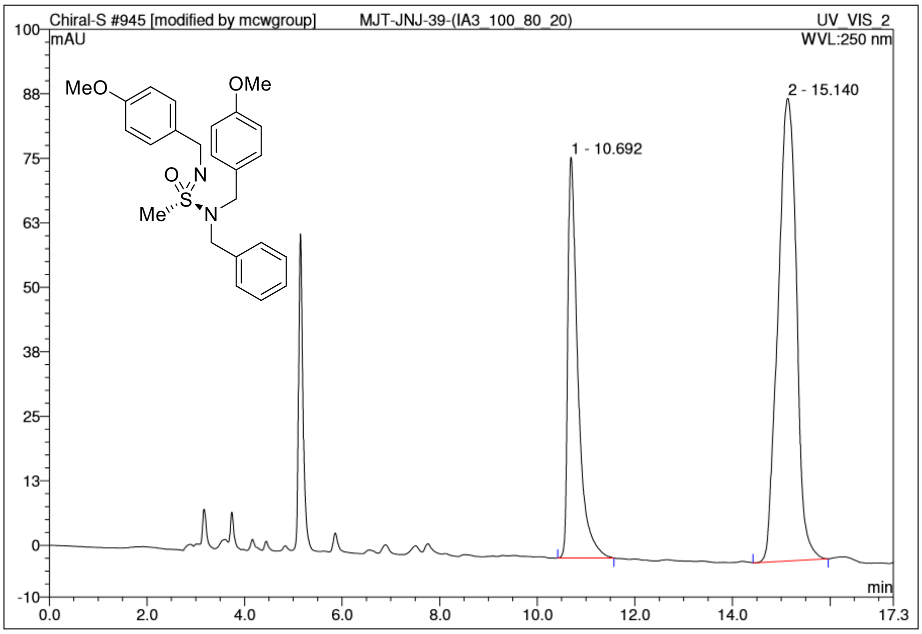

No.	Ret.Time min	Peak Name	Height mAU	Area mAU*min	Rel.Area %	Amount	Type
1	10.69	n.a.	77.707	18.695	32.17	n.a.	BMB*
2	15.14	n.a.	89.646	39.421	67.83	n.a.	BMB*
Total:			167.353	58.115	100.00	0.000	

N-benzyl-*N*-(4-methoxybenzyl)-4-methylbenzenesulfonimidamide (5)

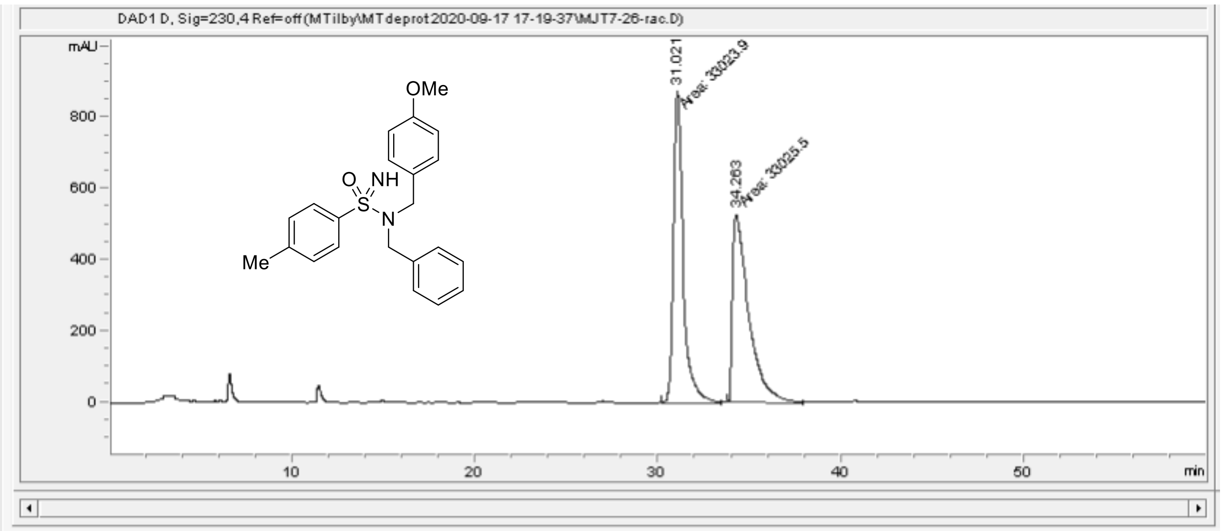

#	Time	Type	Area	Height	Width	Area%	Symmetry
1	31.021	MM	33023.9	879	0.6262	49.999	0.565
2	34.263	MM	33025.5	528	1.0424	50.001	0.254

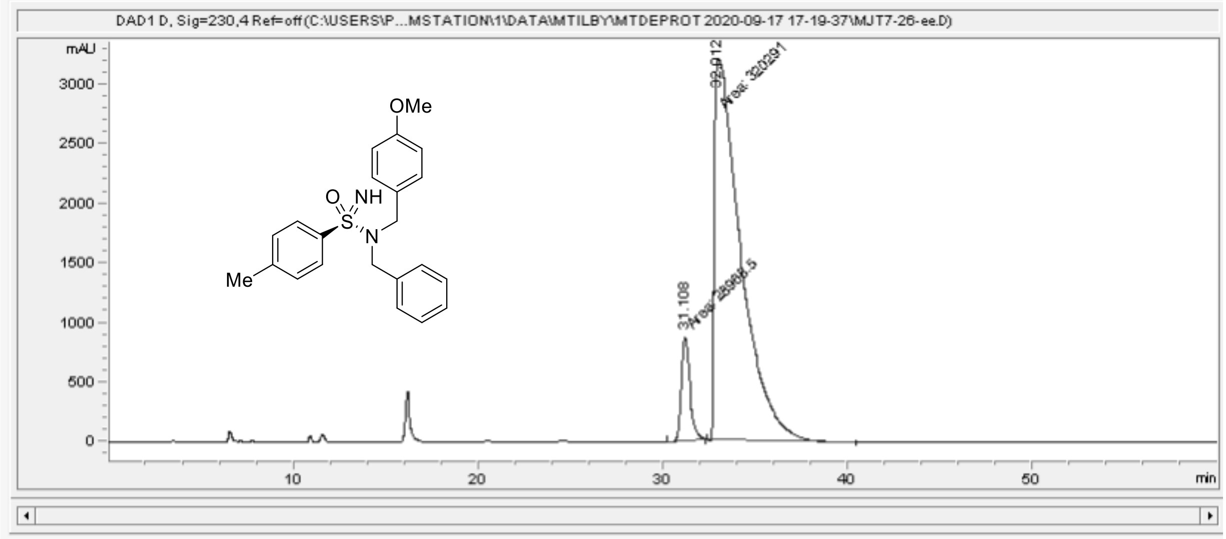

#	Time	Type	Area	Height	Width	Area%	Symmetry
1	31.108	MM	28968.5	877.6	0.5502	8.294	0.643
2	32.912	MM	320291.5	3191.6	1.6726	91.706	0.155

N'-benzyl-4-methylbenzenesulfonimidamide (6)

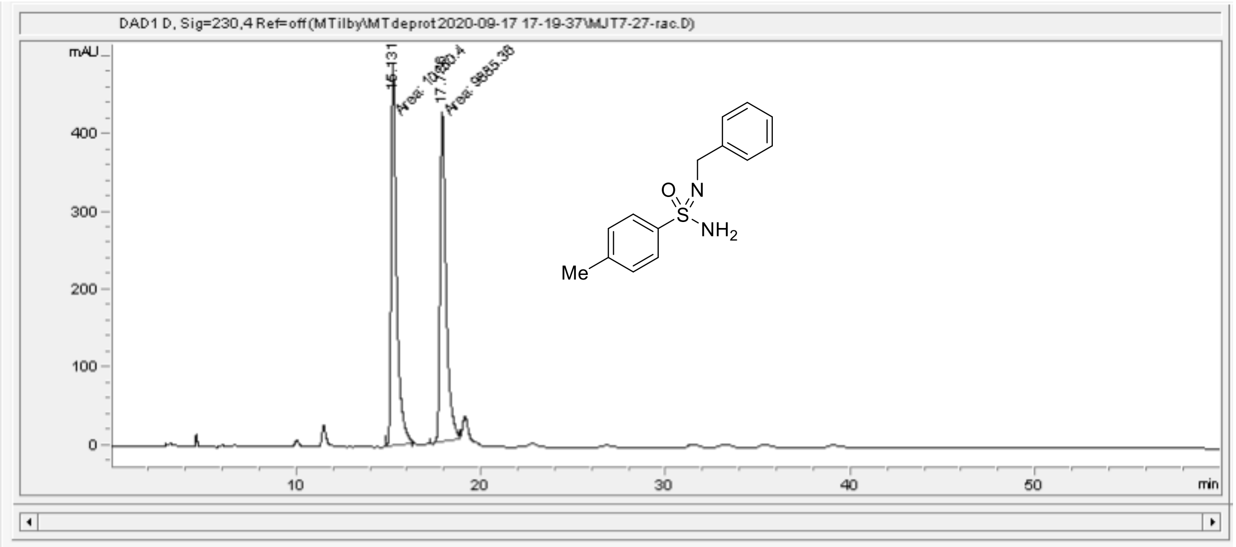

#	Time	Type	Area	Height	Width	Area%	Symmetry
1	15.131	MM	10150.4	491.6	0.3441	50.661	0.489
2	17.786	MM	9885.4	426.4	0.3863	49.339	0.549

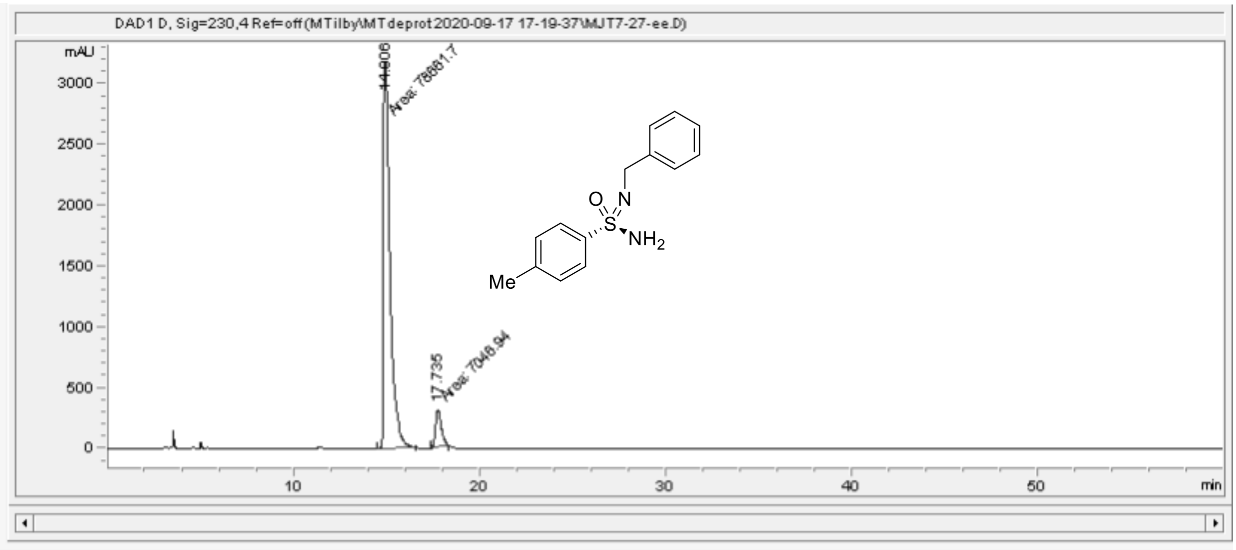

#	Time	Type	Area	Height	Width	Area%	Symmetry
1	14.906	MM	78661.7	3177.6	0.4126	91.778	0.37
2	17.735	MM	7046.9	320.3	0.3667	8.222	0.601

11. NMR-Spectra

(*S*)-4-methyl-*N*-(4-methylbenzylidene)benzenesulfinamide (7)

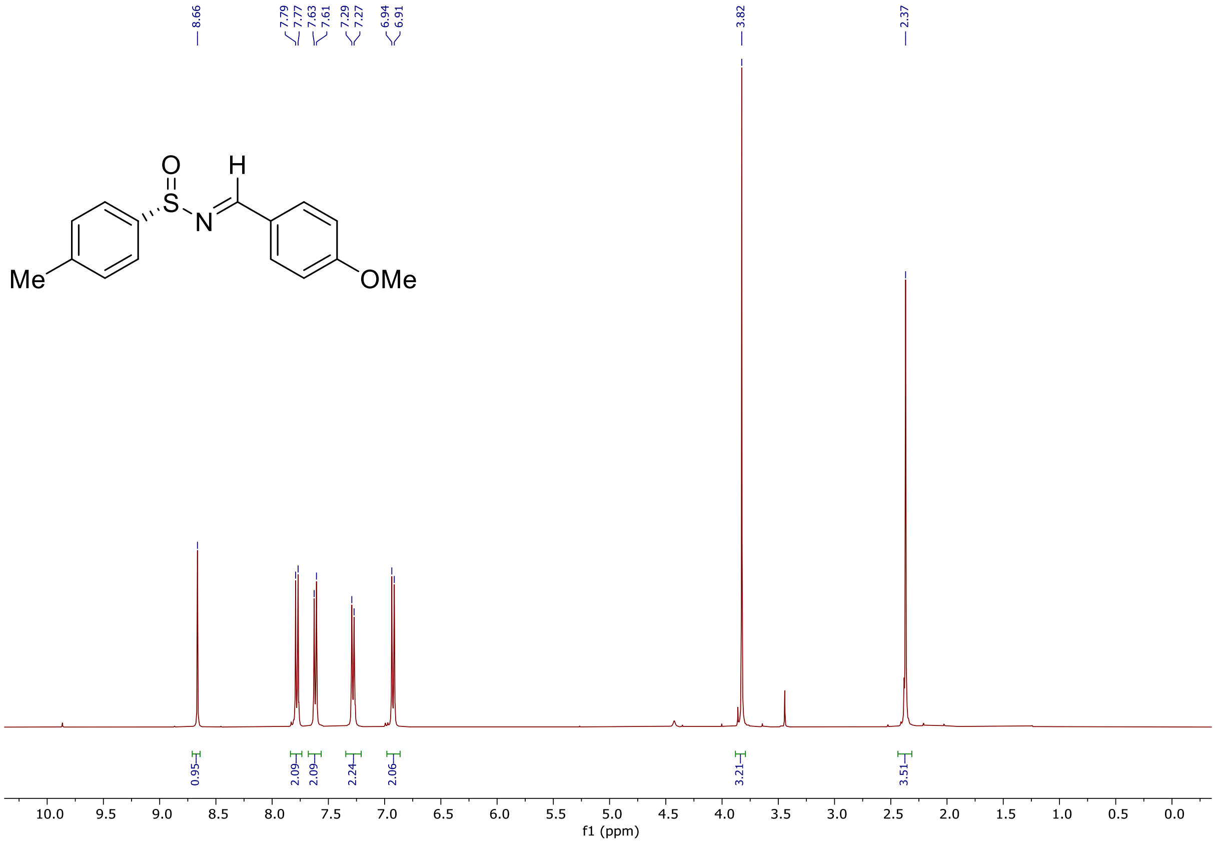

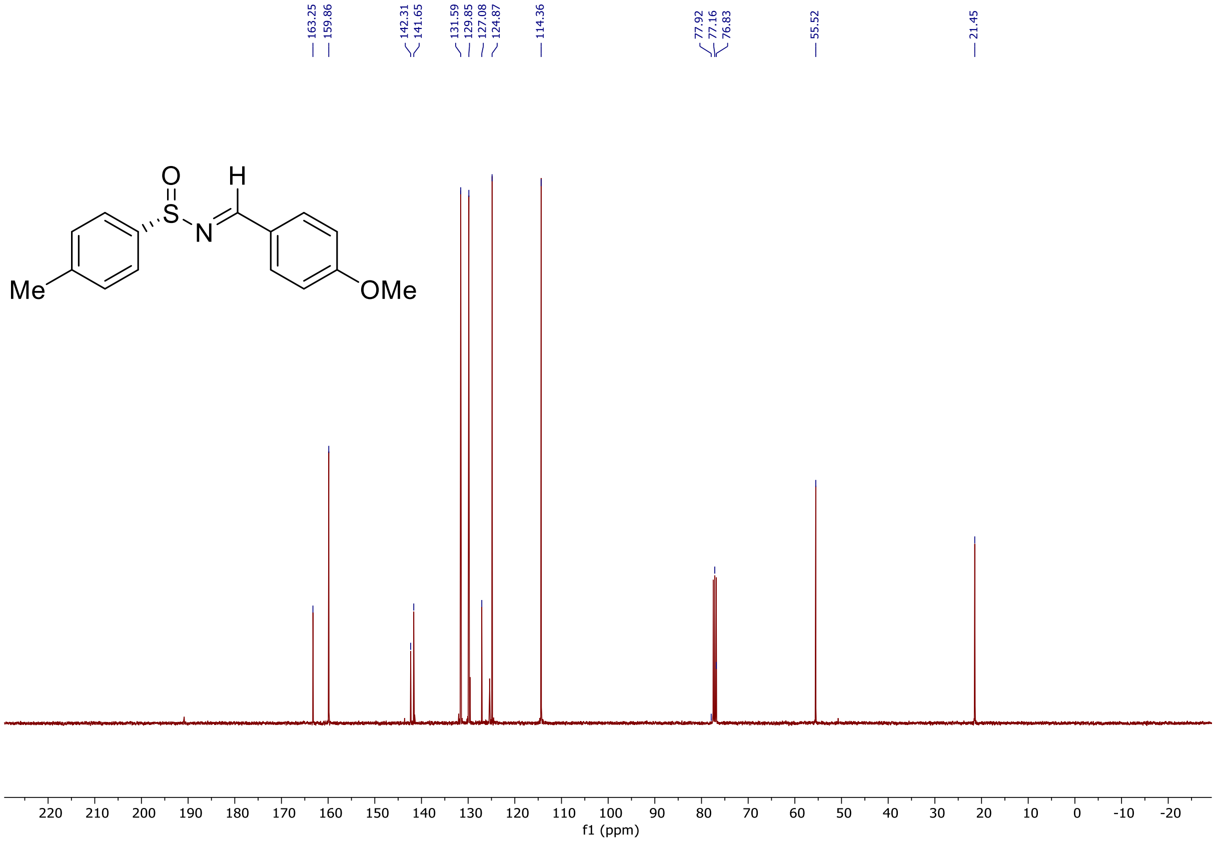

(*S*)-4-methyl-*N*-(4-methylbenzyl)benzenesulfinamide (1)

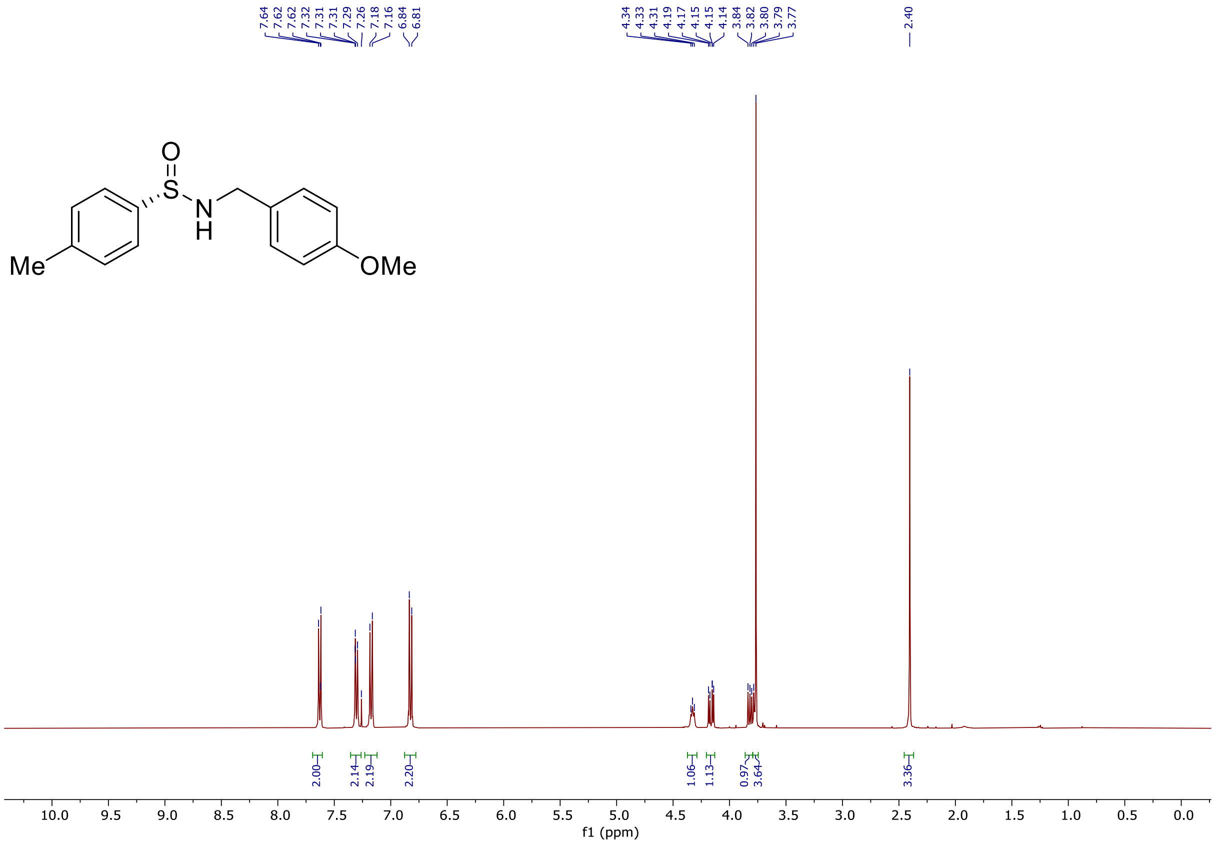

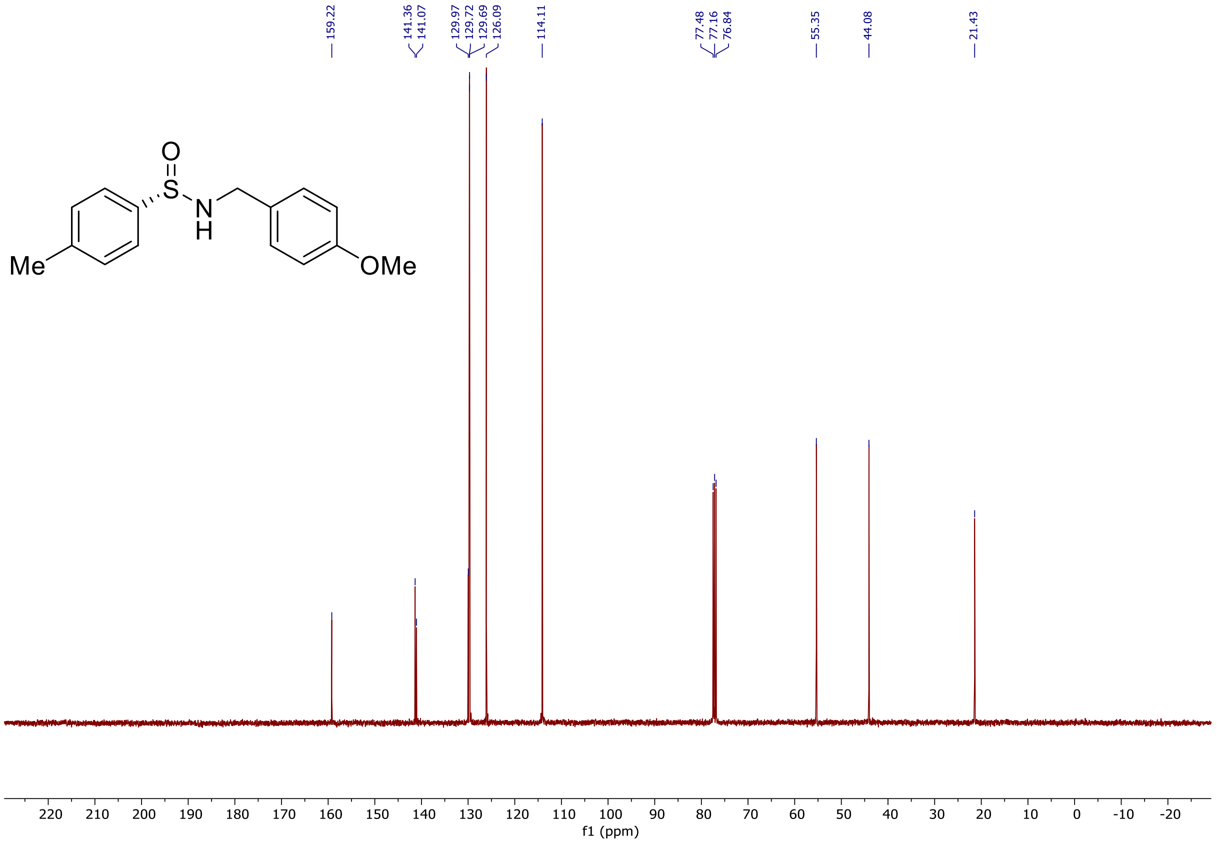

N-benzyl-1-(4-methoxyphenyl)methanamine (8)

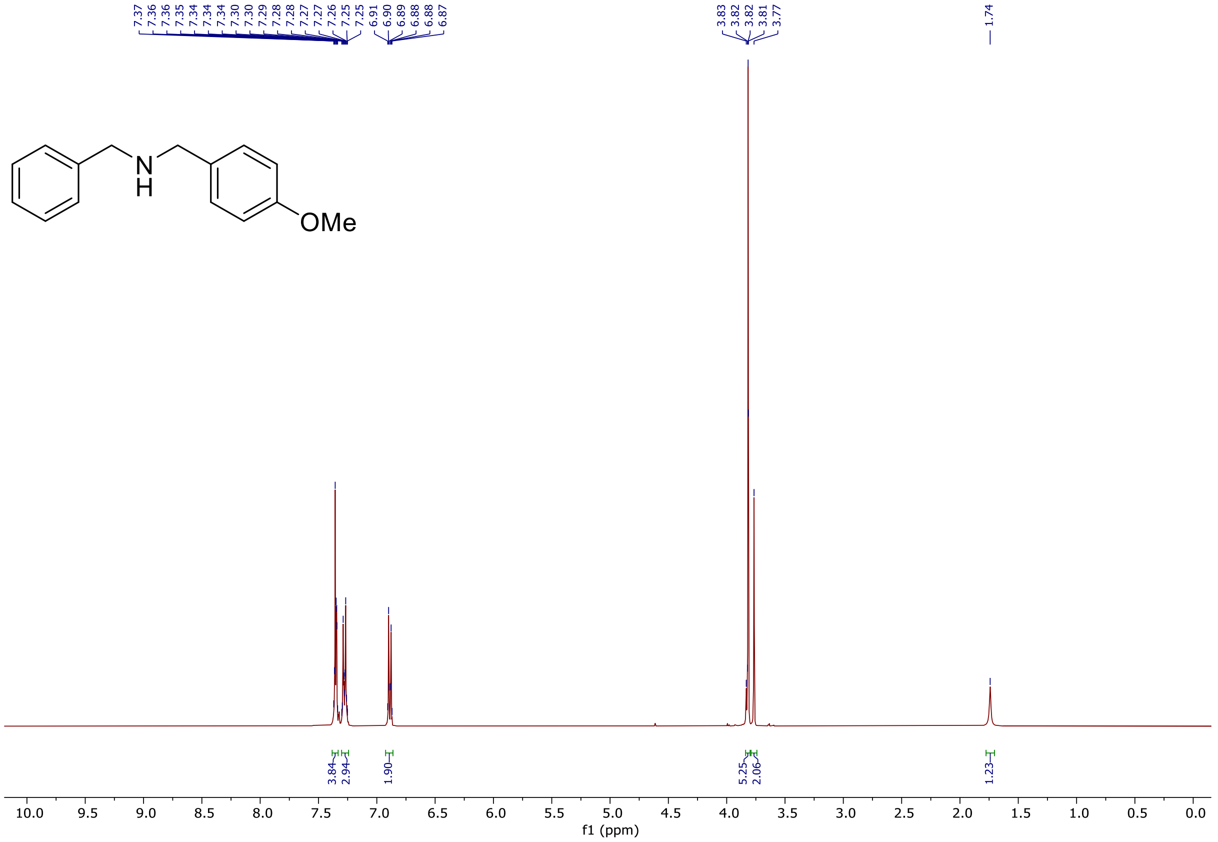

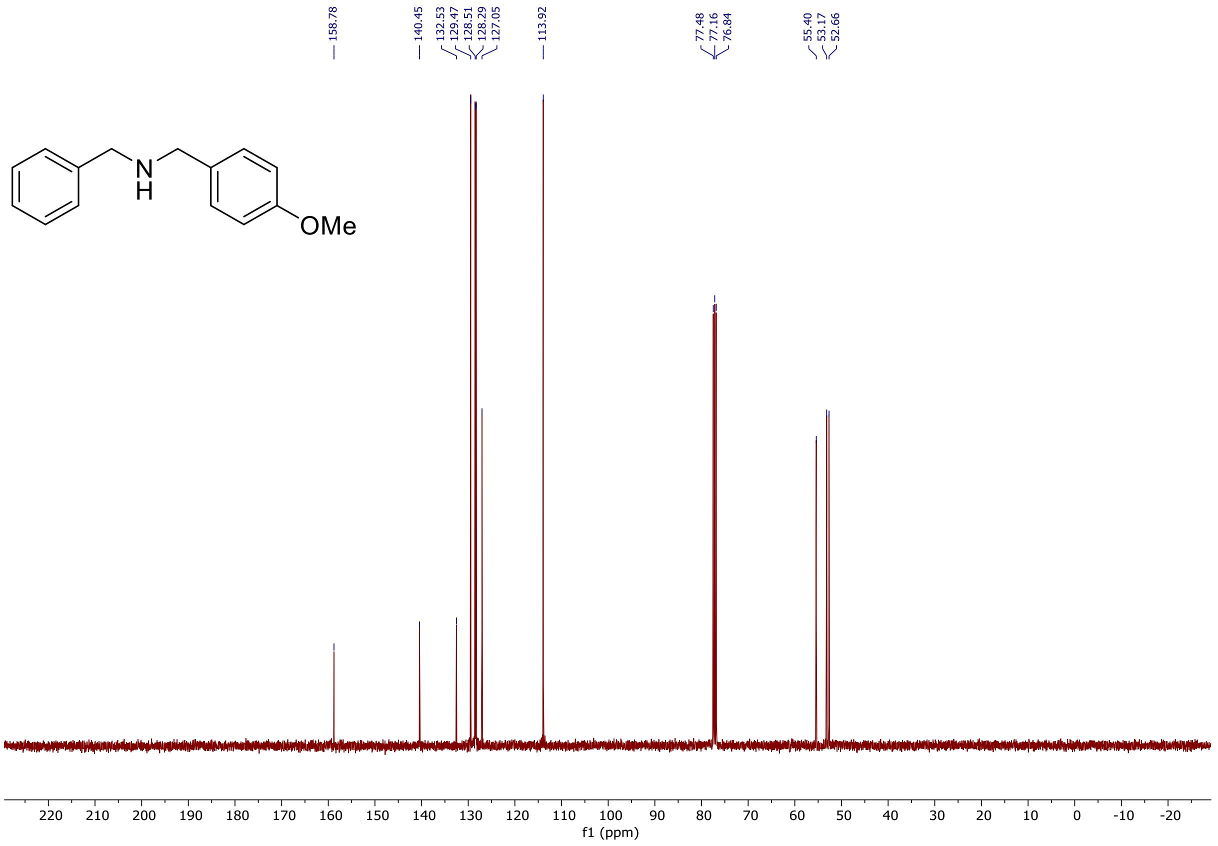

TrNSO (9)

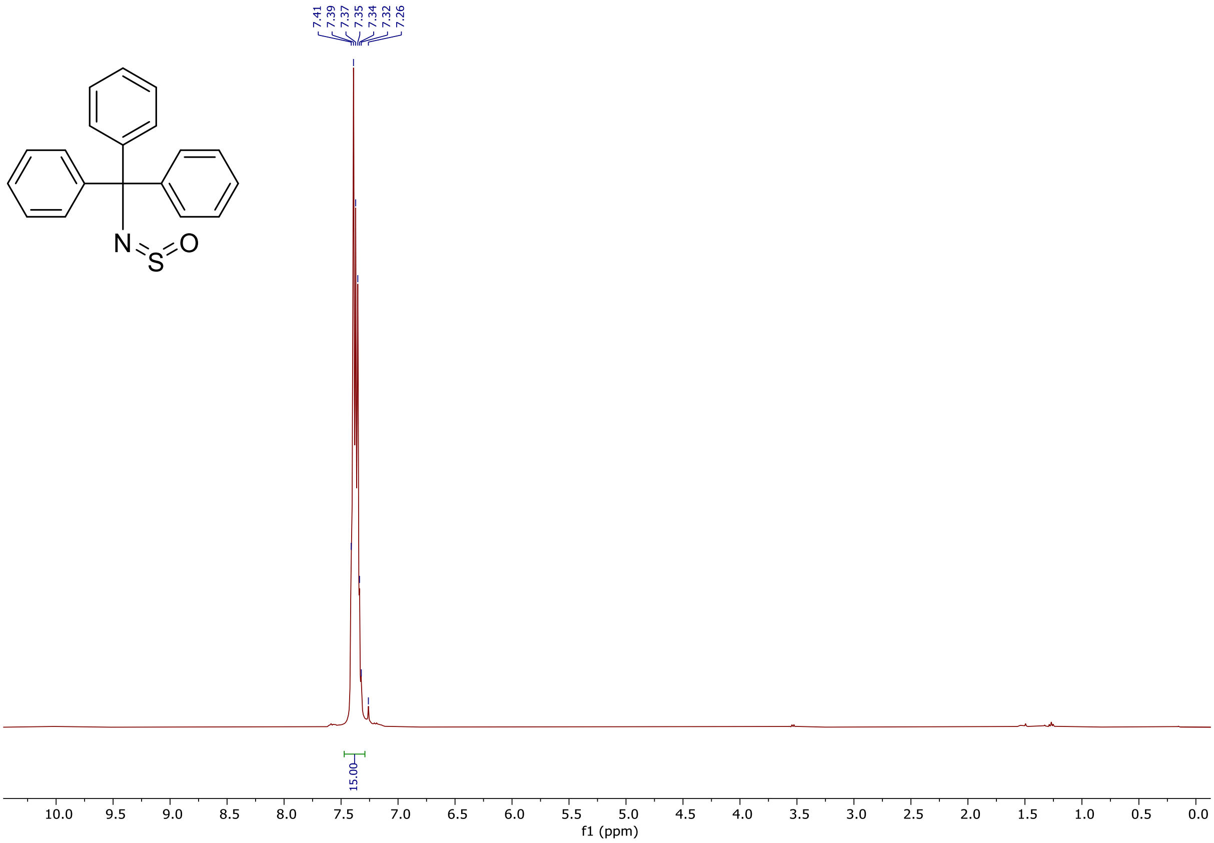

7.41
7.39
7.37
7.35
7.34
7.32
7.26
15.00
10.0 9.5 9.0 8.5 8.0 7.5 7.0 6.5 6.0 5.5 5.0 4.5 4.0 3.5 3.0 2.5 2.0 1.5 1.0 0.5 0.0
f1 (ppm)

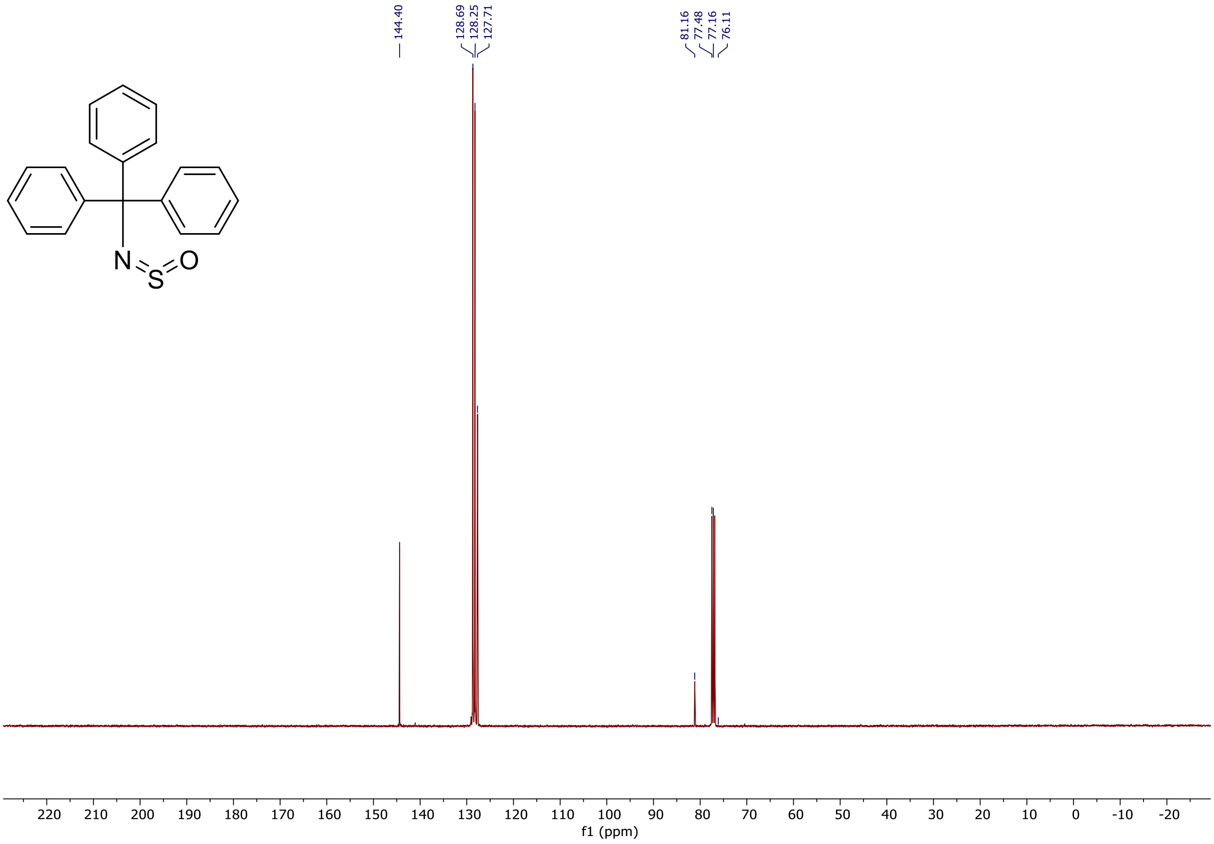

144.40
128.69
128.25
127.71
81.16
77.48
77.16
76.11
220 210 200 190 180 170 160 150 140 130 120 110 100 90 80 70 60 50 40 30 20 10 0 -10 -20
f1 (ppm)

1-(4-Bromophenyl)-5-(*p*-tolyl)-3-(trifluoromethyl)-1*H*-pyrazole (10)

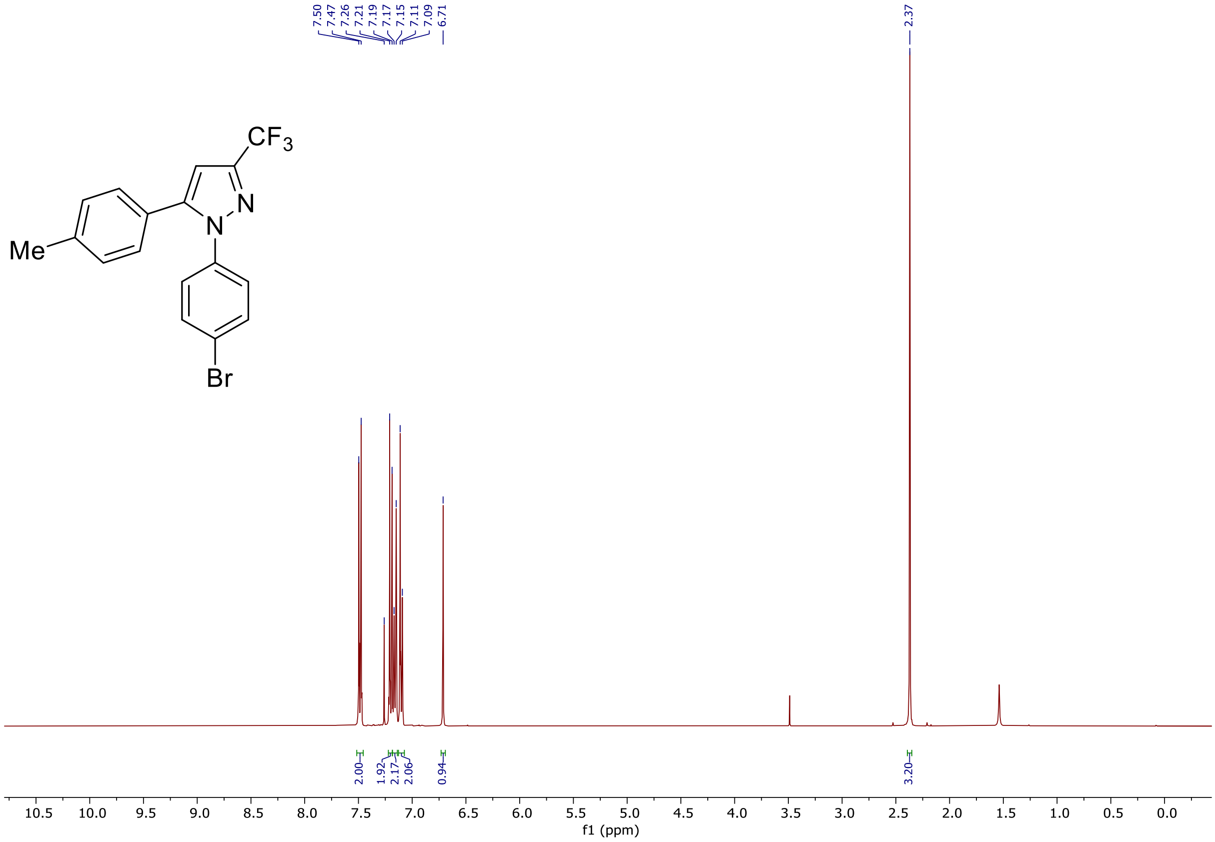

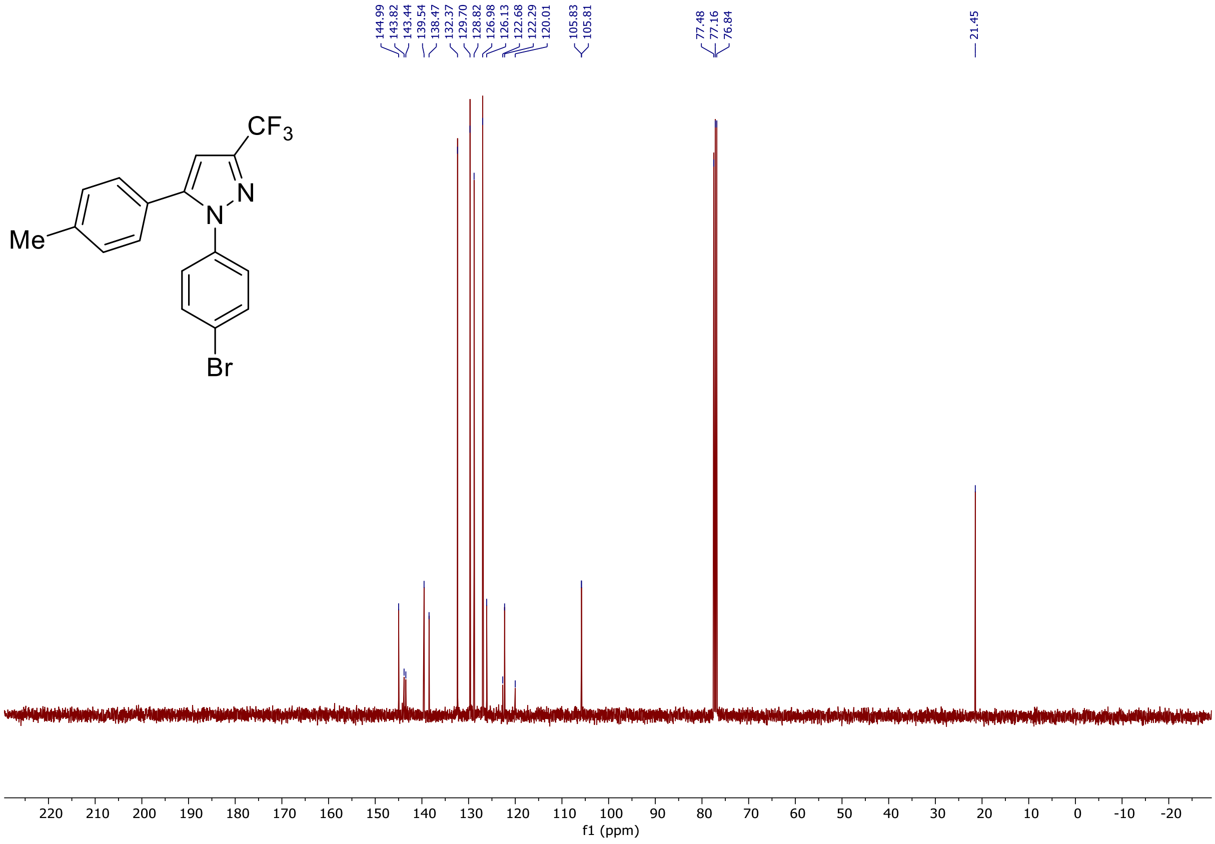

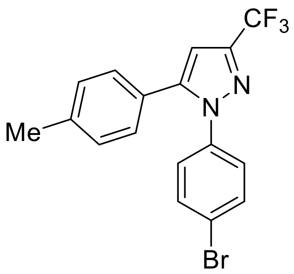
CF3
N
N
Me
Br

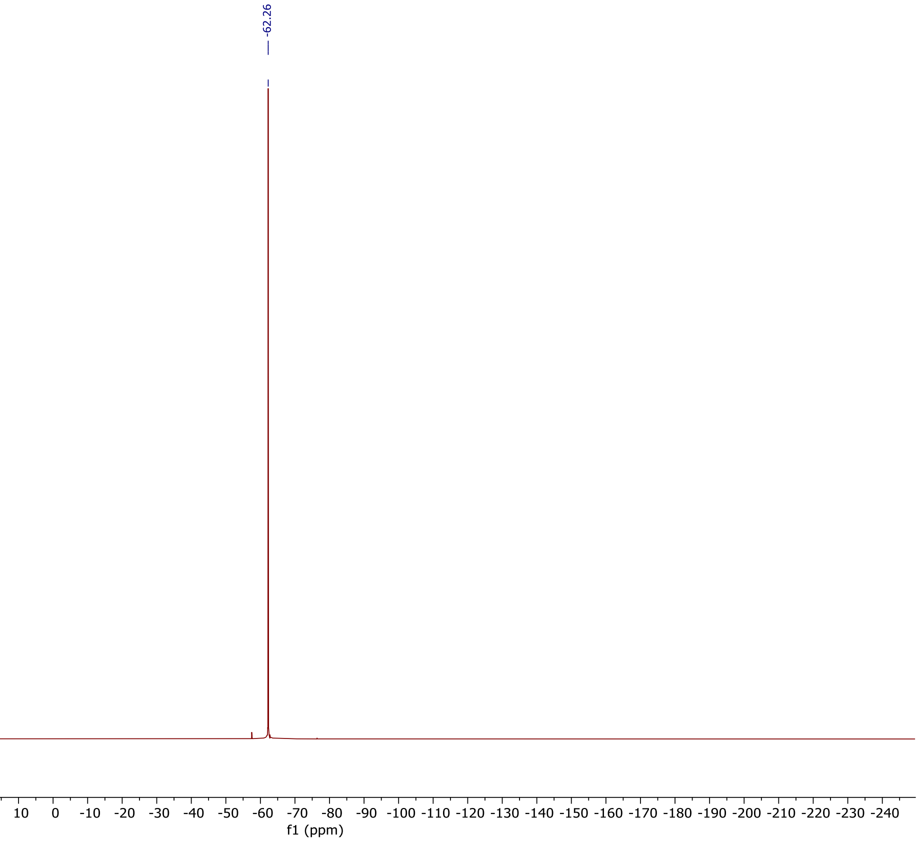
-62.26
90 80 70 60 50 40 30 20 10 0 -10 -20 -30 -40 -50 -60 -70 -80 -90 -100 -110 -120 -130 -140 -150 -160 -170 -180 -190 -200 -210 -220 -230 -240
f1 (ppm)

[1,1'-biphenyl]-4-sulfonimidamide (4)

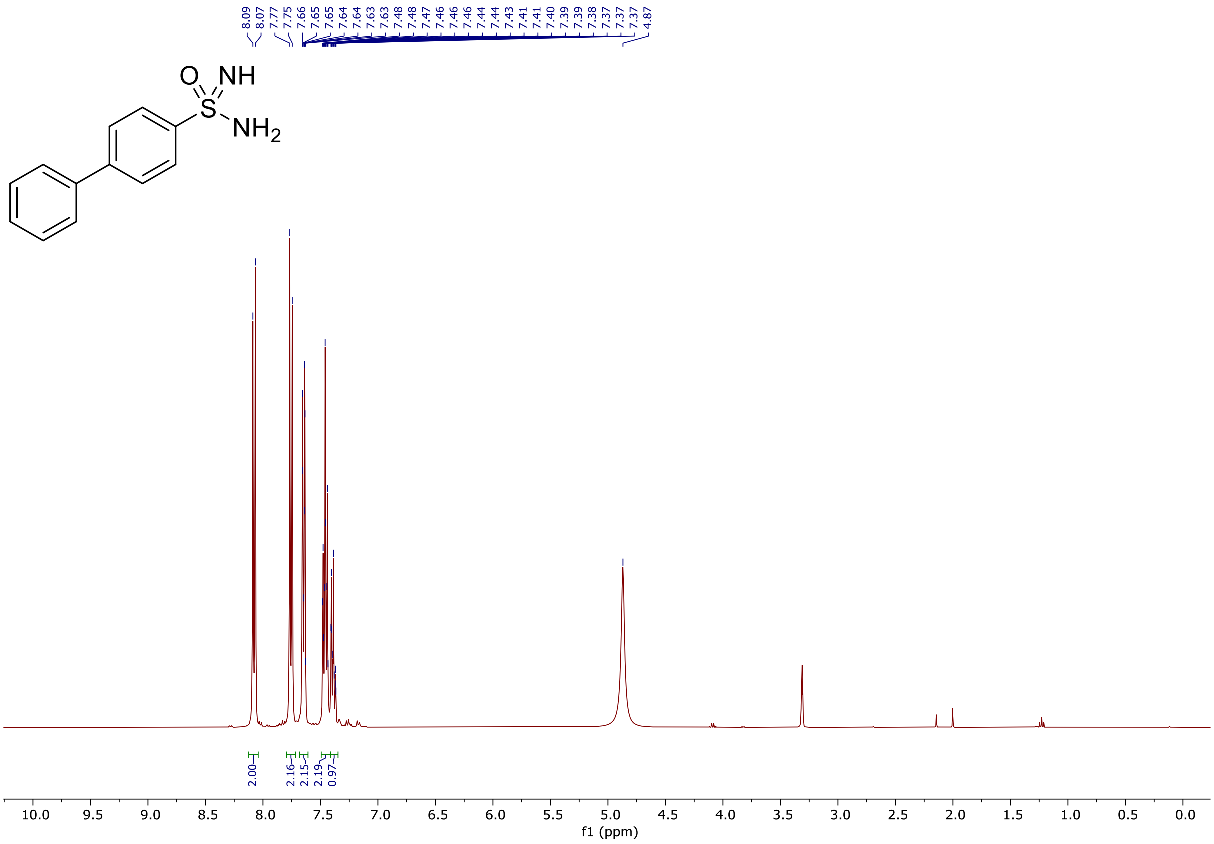

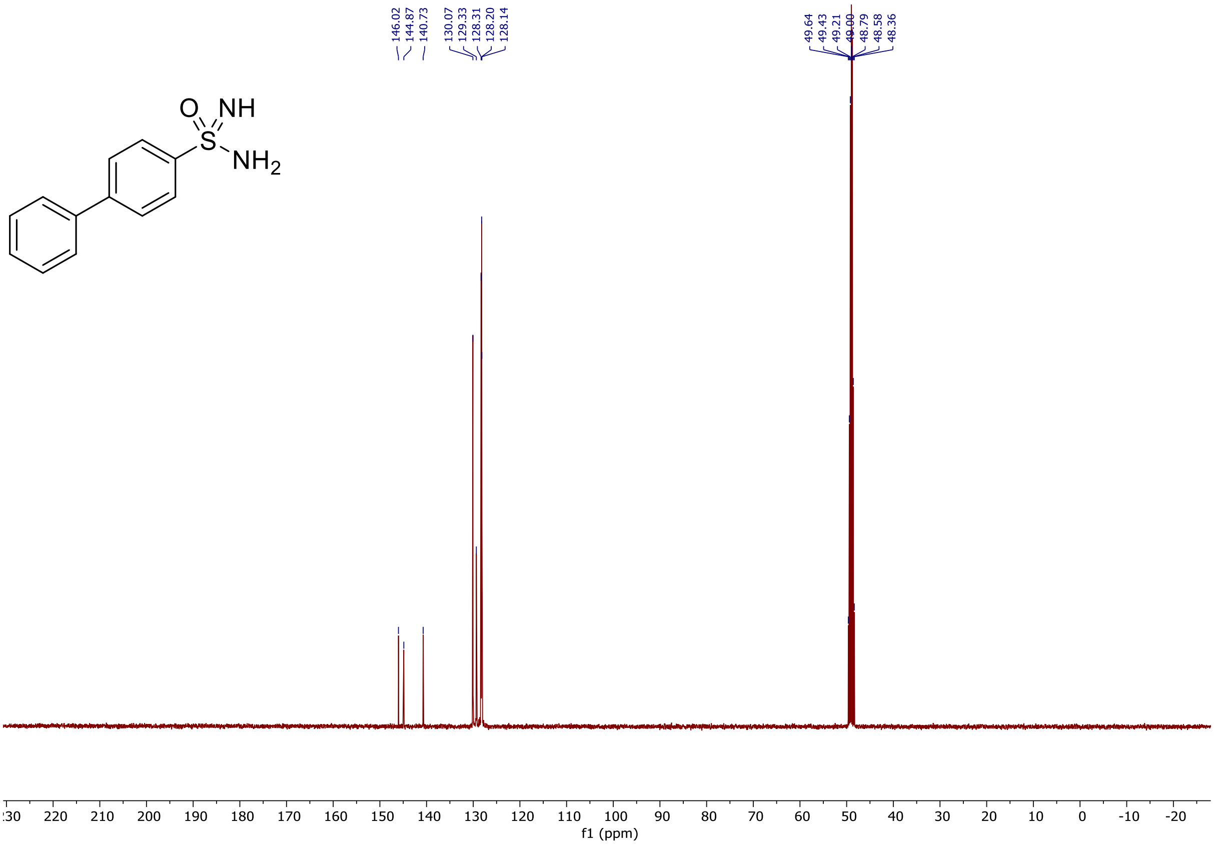

[1,1'-biphenyl]-4-sulfonimidamide (4)

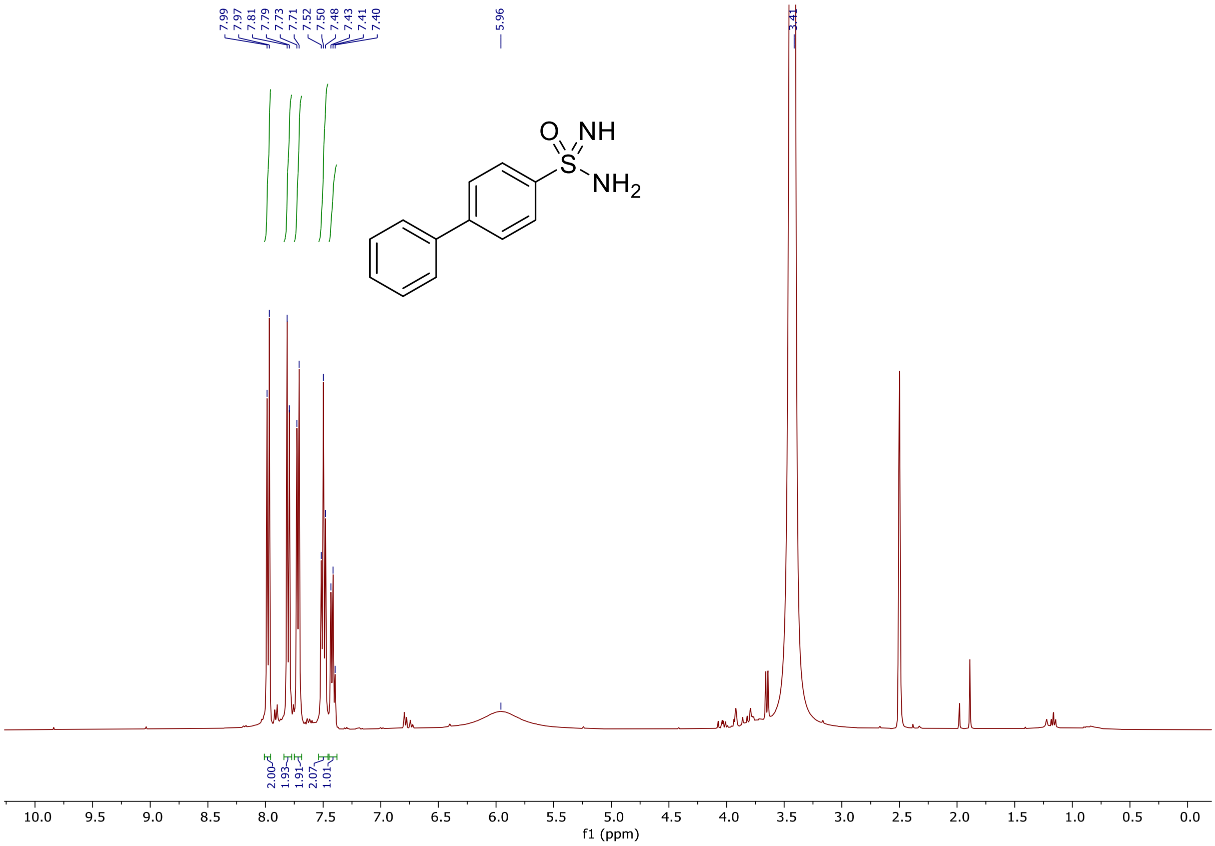

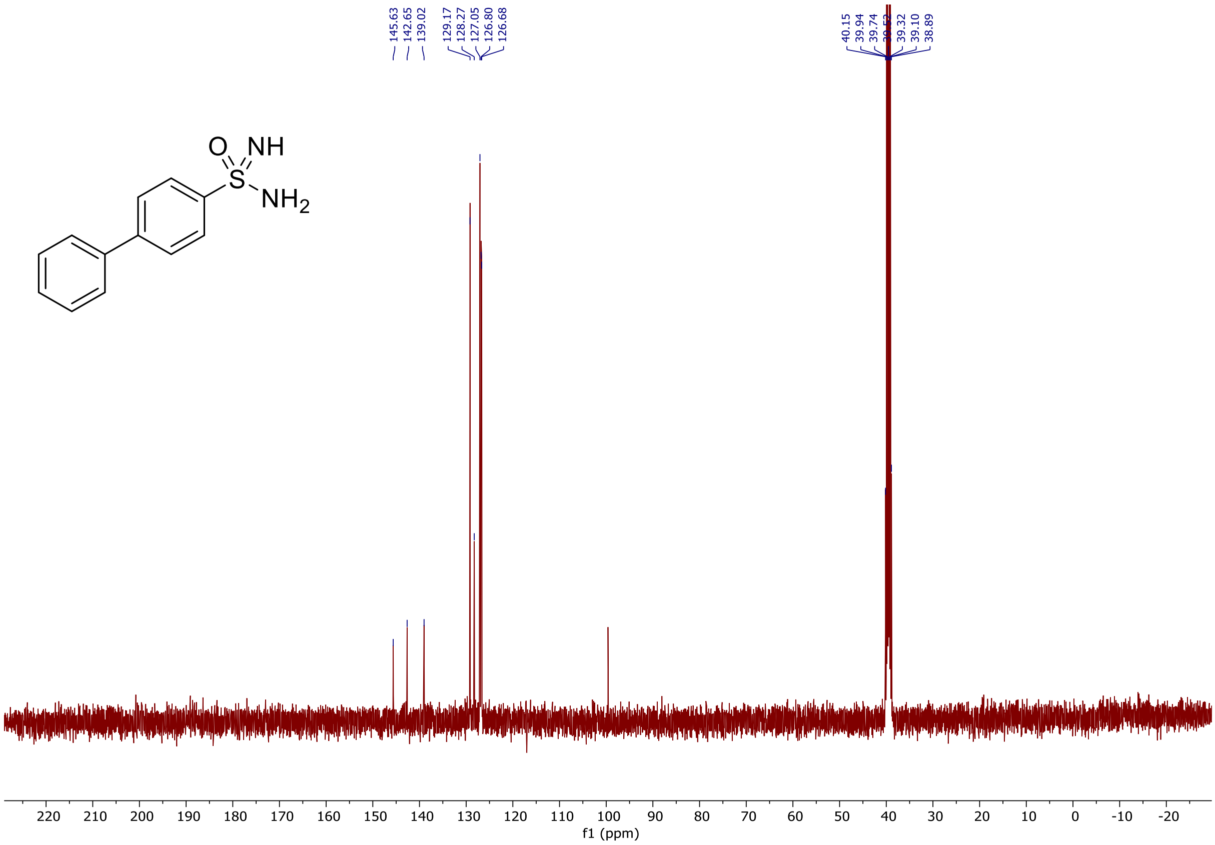

N-(4-cyanobenzyl)cinchoninium bromide (CAT-1)

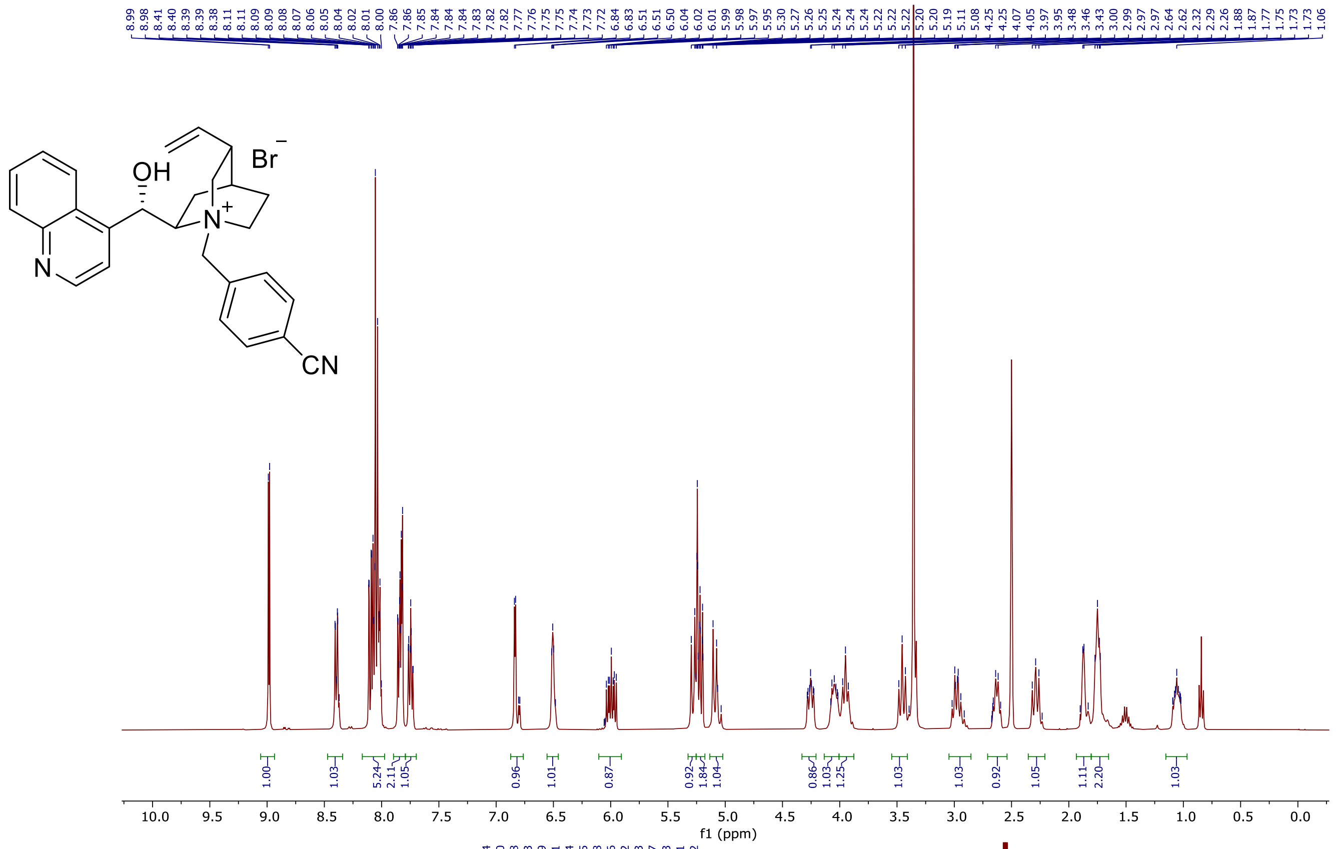

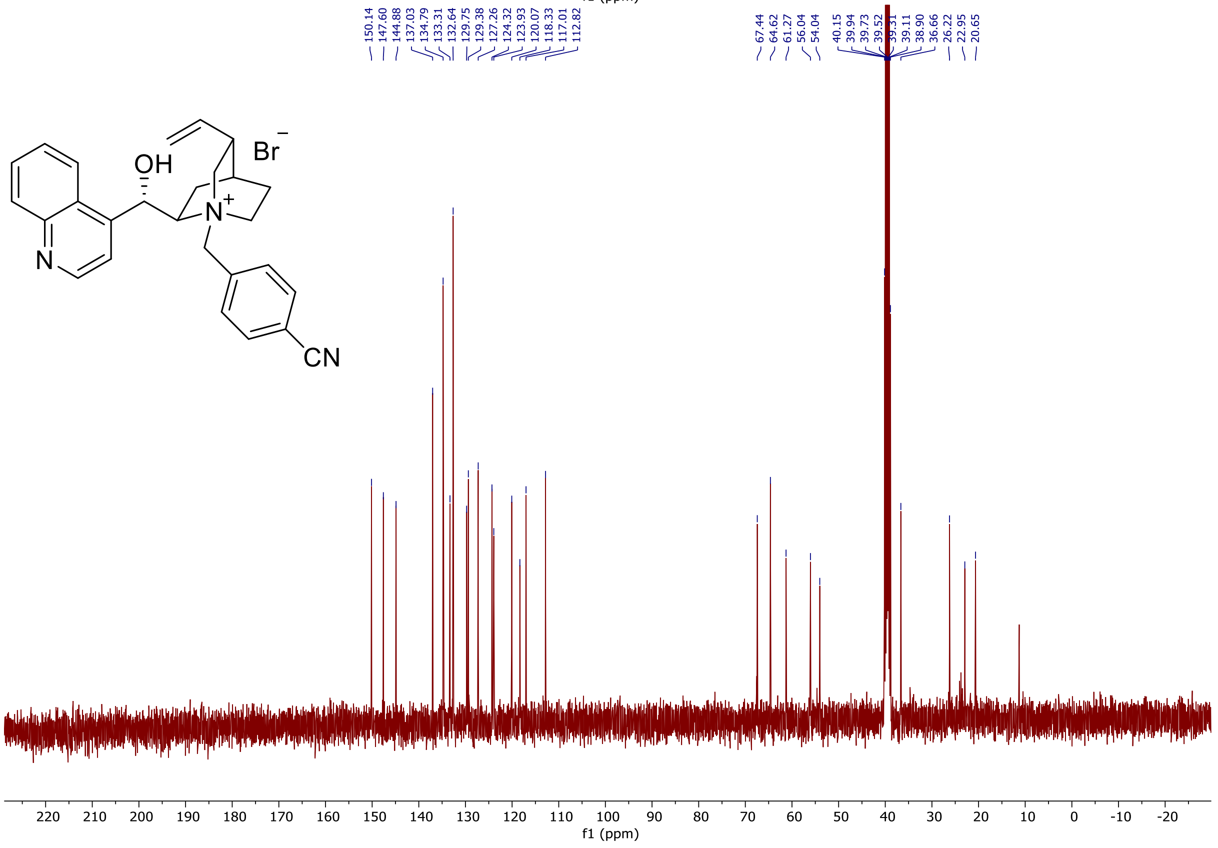

***O*-allyl-*N*-(4-cyanobenzyl)cinchoninium bromide (CAT-13)**

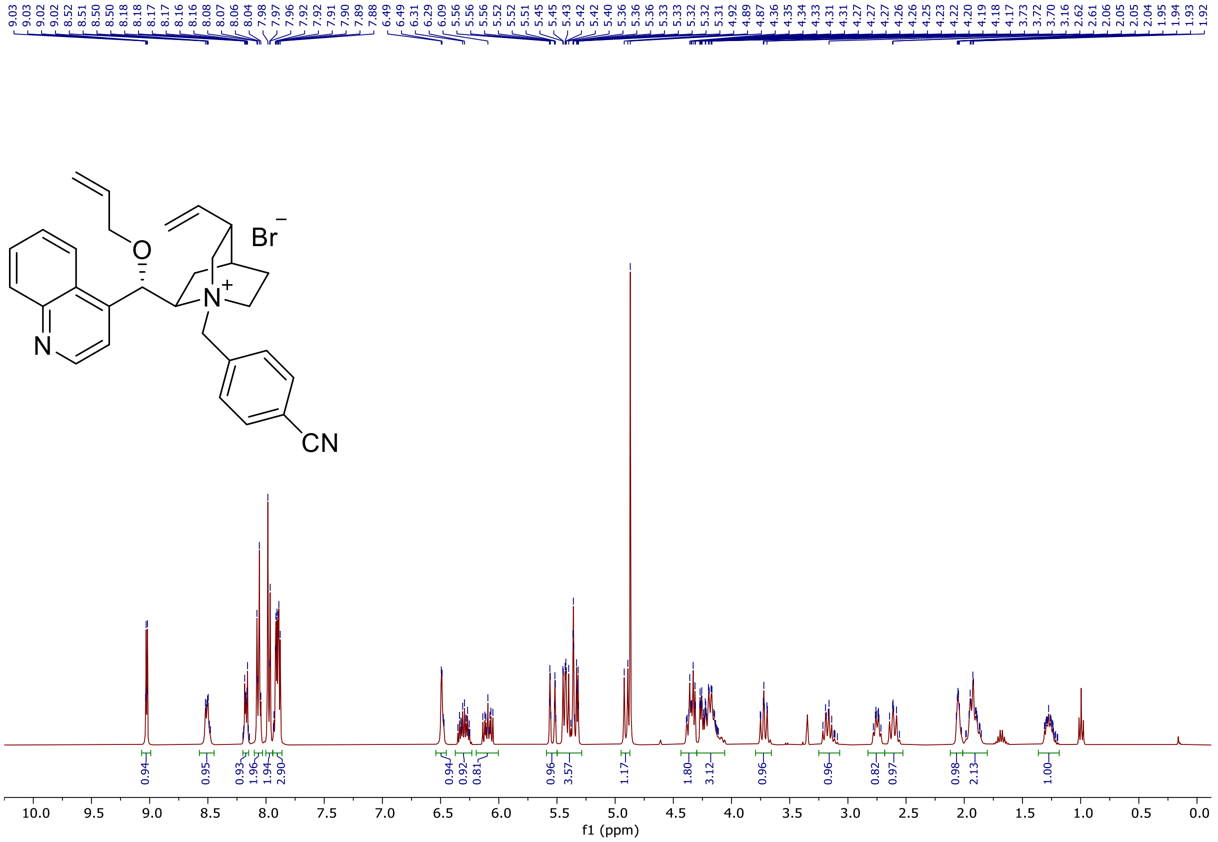

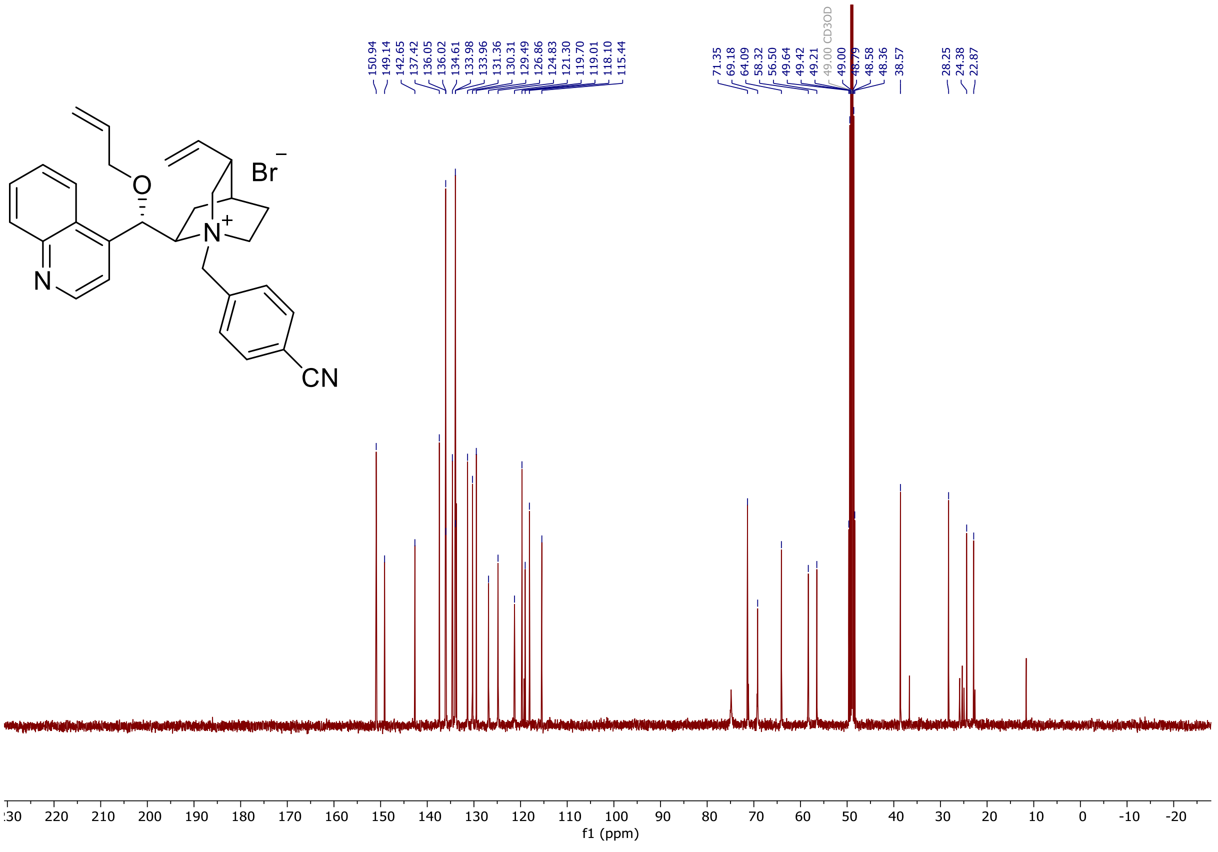

N-(4-cyanobenzyl)-*N'*-(2,5-difluorobenzyl)cinchoninium dibromide (CAT-7)

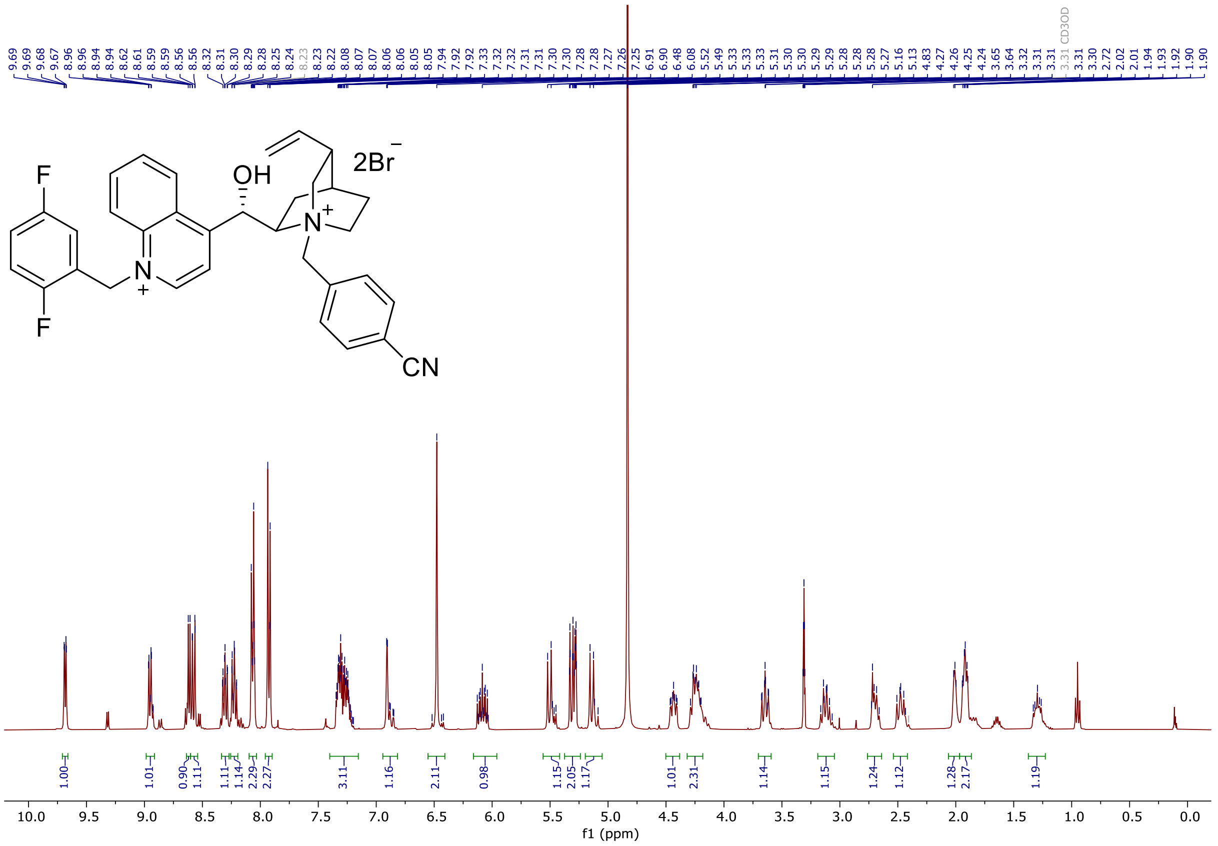

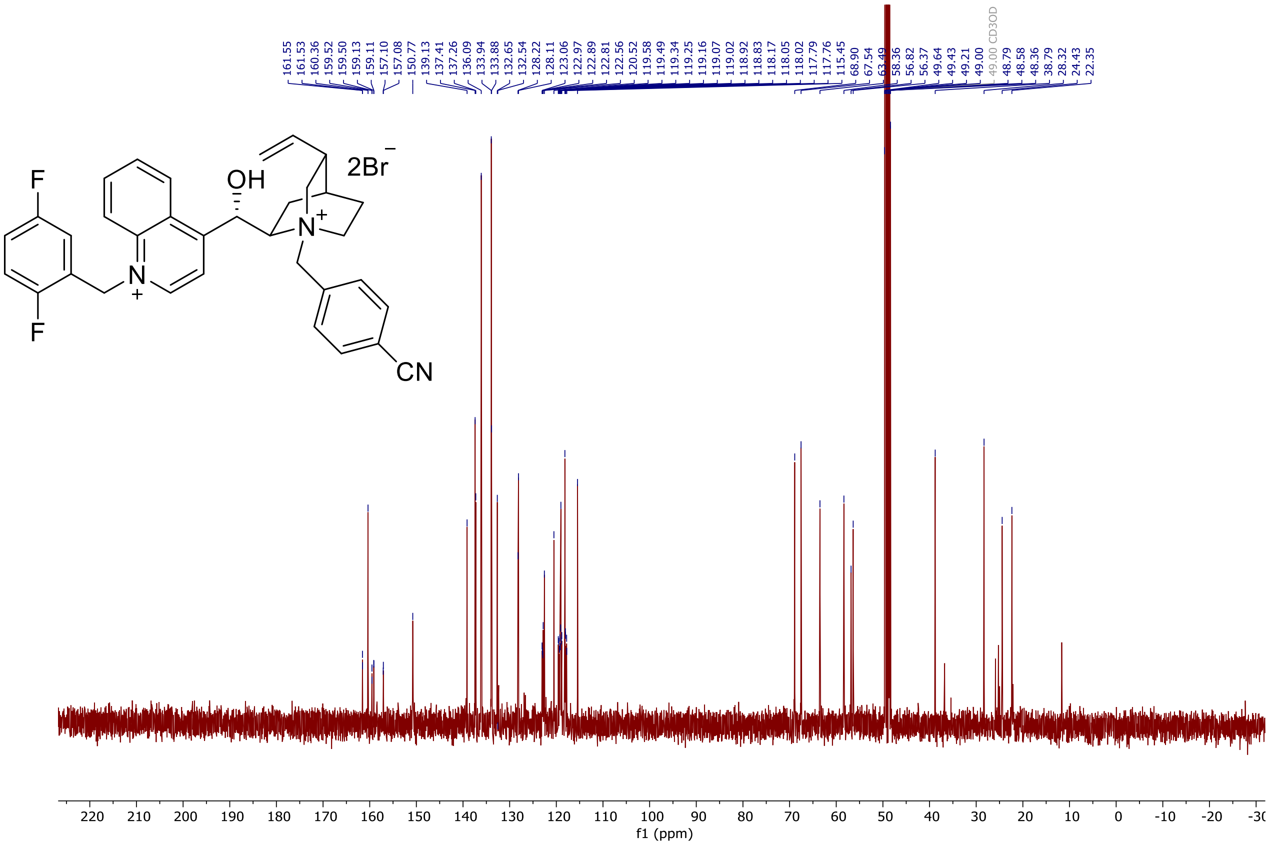

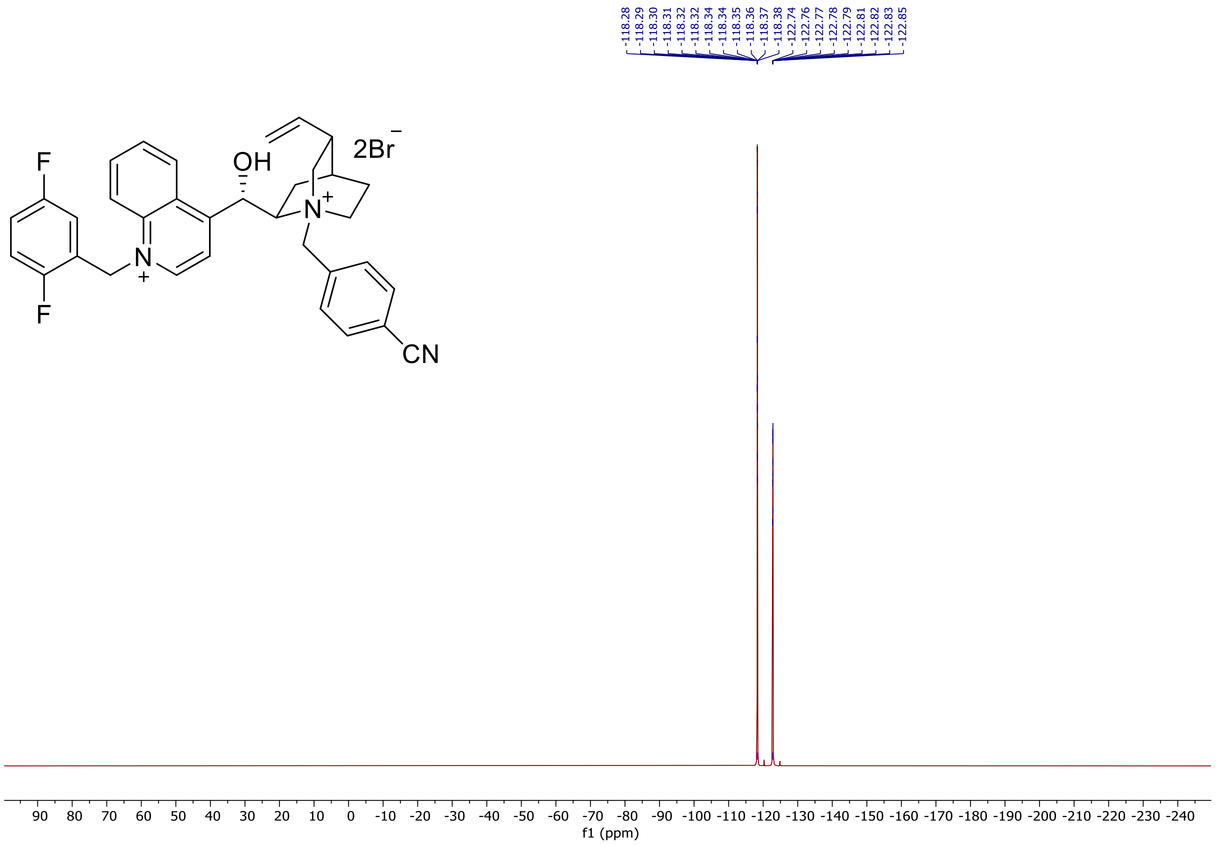

-118.28
-118.29
-118.30
-118.31
-118.32
-118.32
-118.34
-118.34
-118.35
-118.36
-118.37
-118.38
-122.74
-122.76
-122.77
-122.78
-122.79
-122.81
-122.82
-122.83
-122.85
F
F
N+
OH
N+
2Br−
CN
90 80 70 60 50 40 30 20 10 0 -10 -20 -30 -40 -50 -60 -70 -80 -90 -100 -110 -120 -130 -140 -150 -160 -170 -180 -190 -200 -210 -220 -230 -240
f1 (ppm)

N'-(4-methoxybenzyl)-[1,1'-biphenyl]-4-sulfonimidamide (11a)

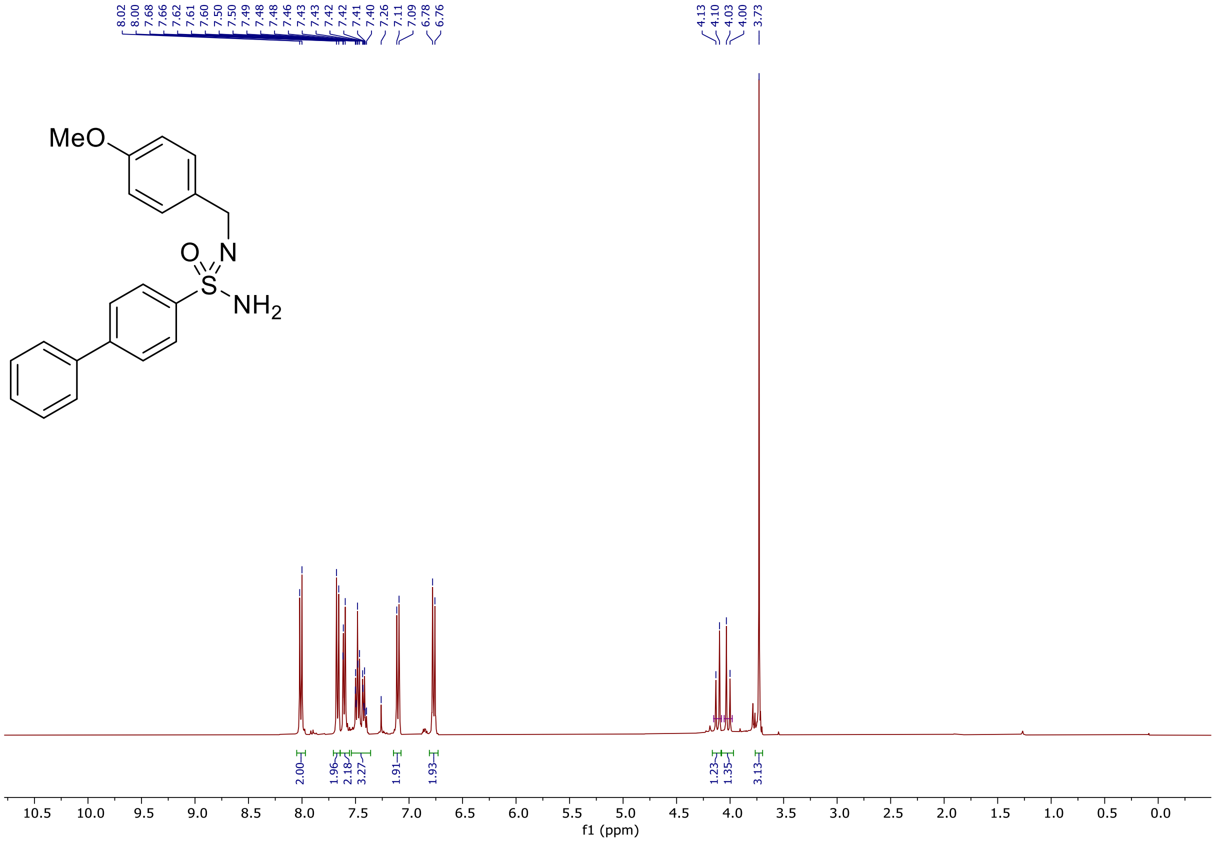

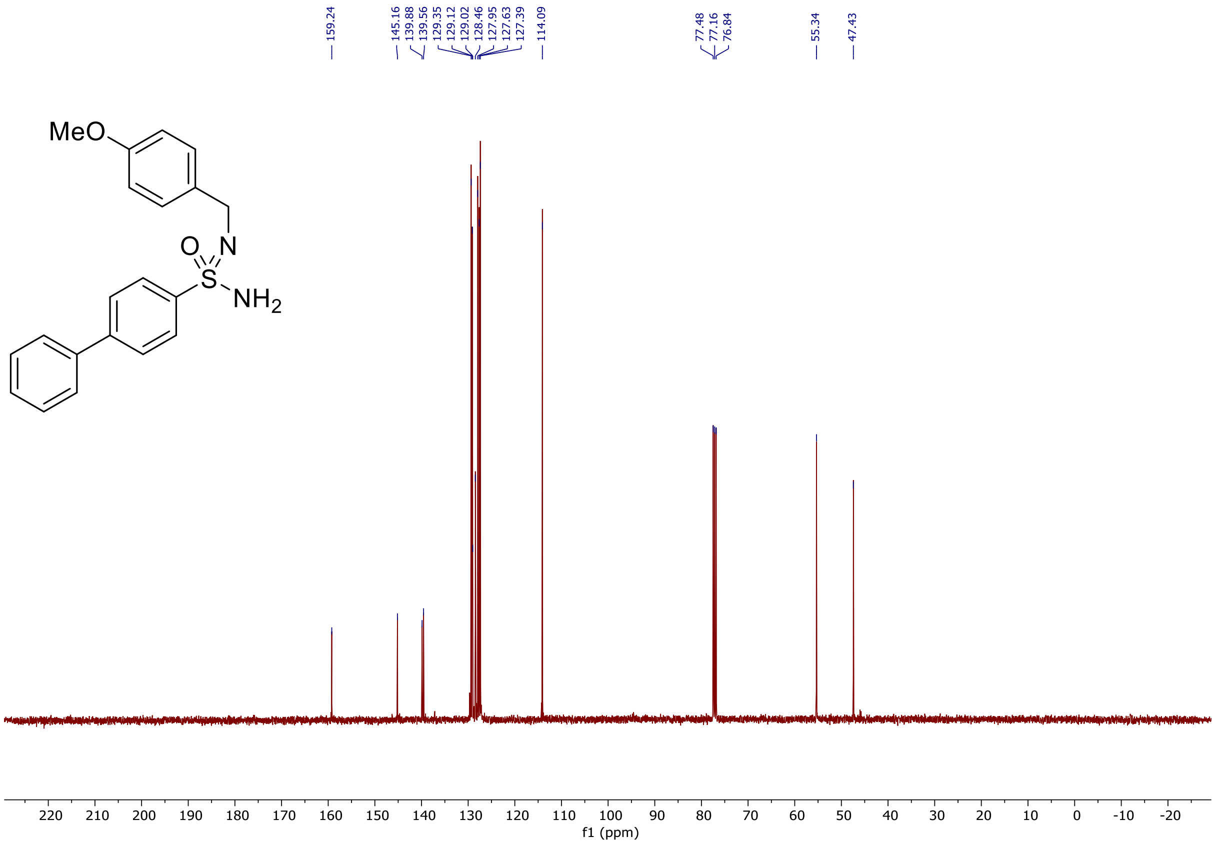

N'-(4-methoxybenzyl)-4-methylbenzenesulfonimidamide (11b)

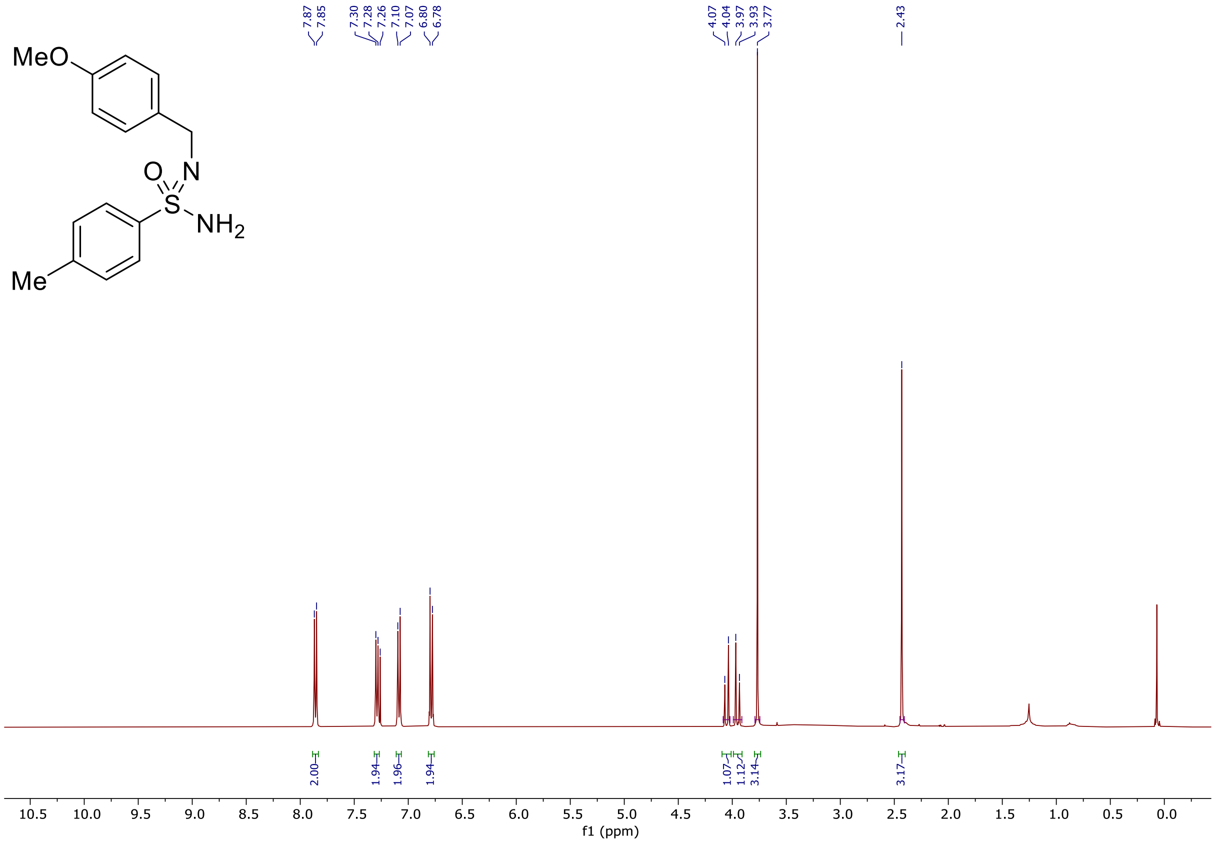

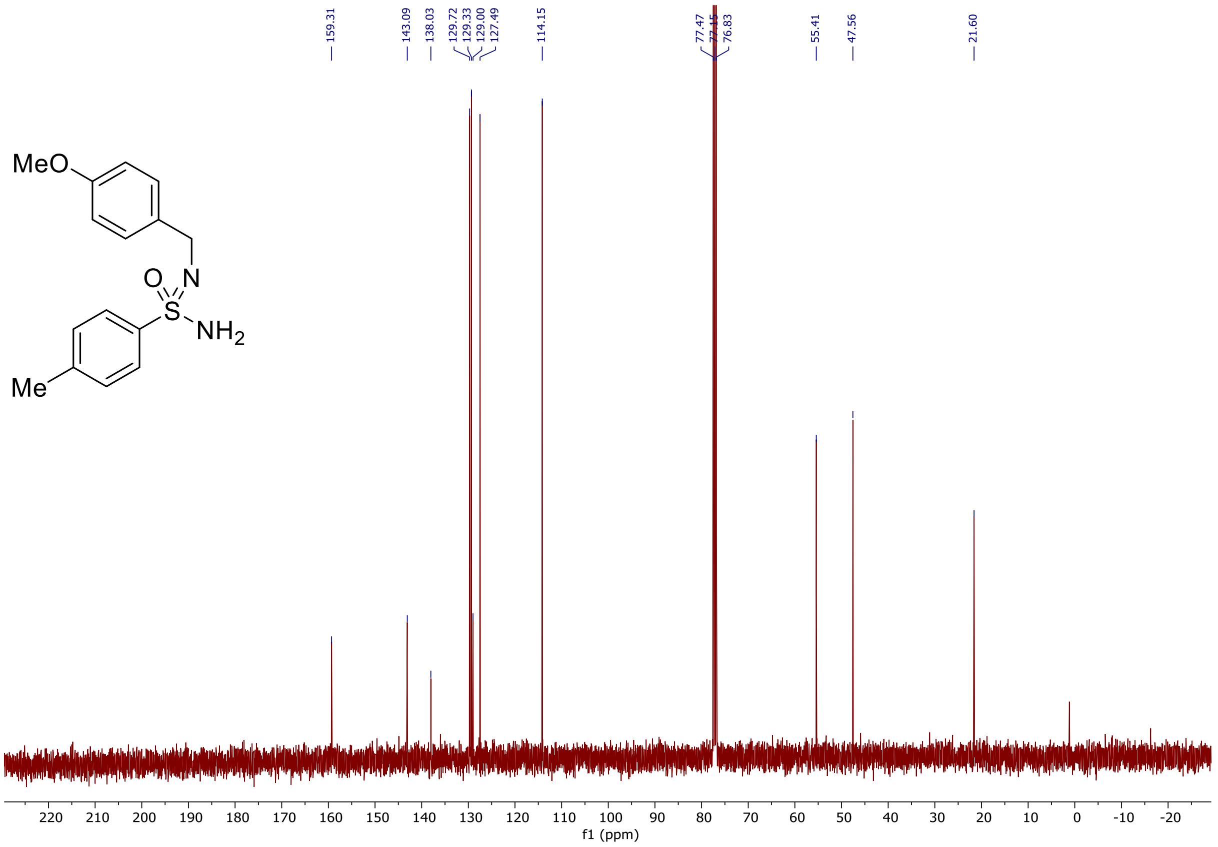

N'-(4-methoxybenzyl)benzenesulfonimidamide (11c)

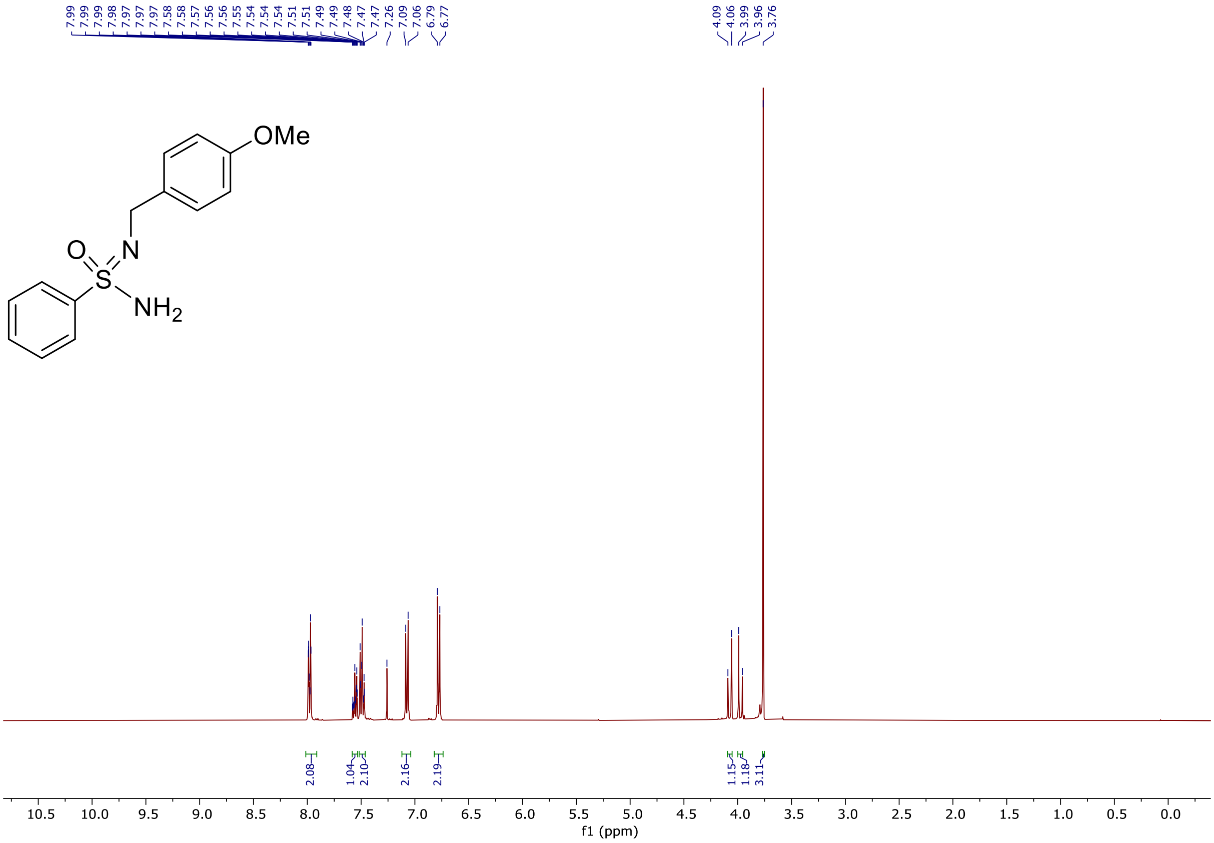

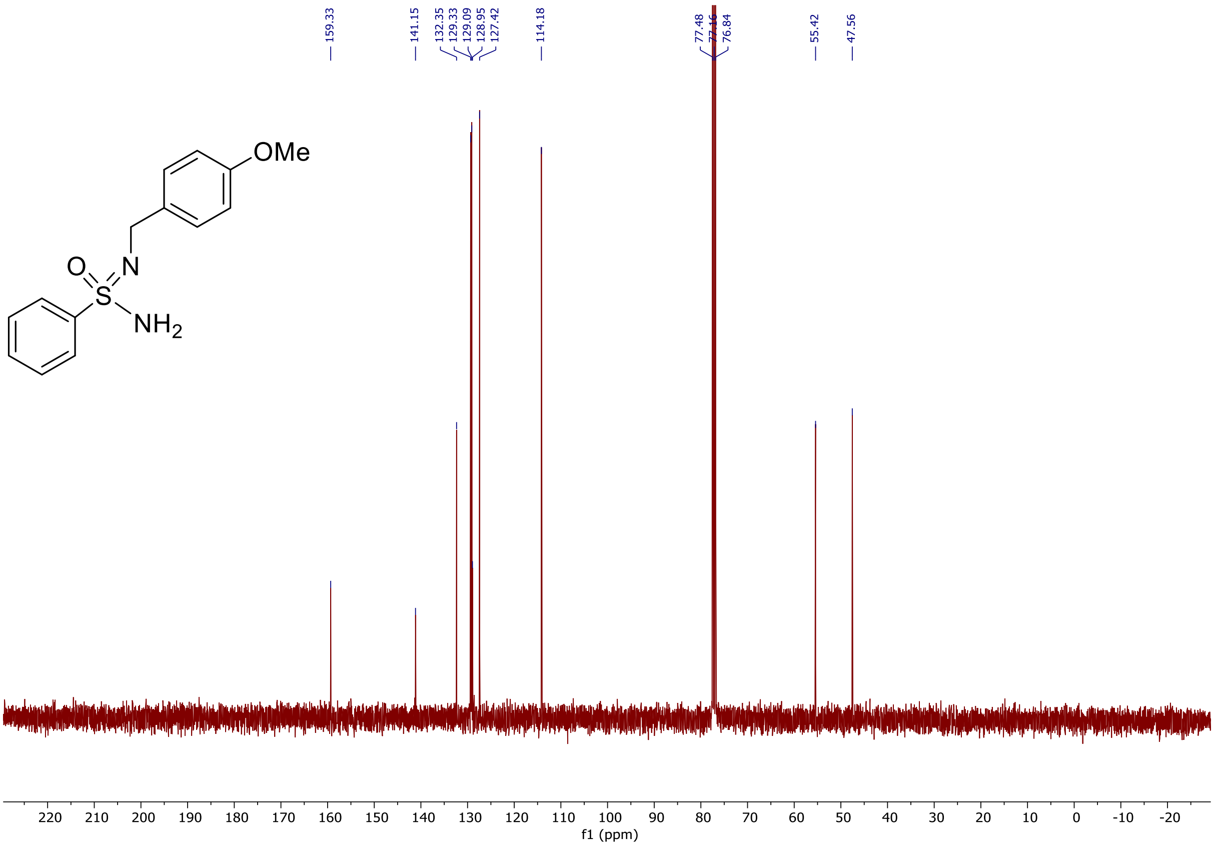

***N'*-(4-methoxybenzyl)-3-methylbenzenesulfonimidamide (11d)**

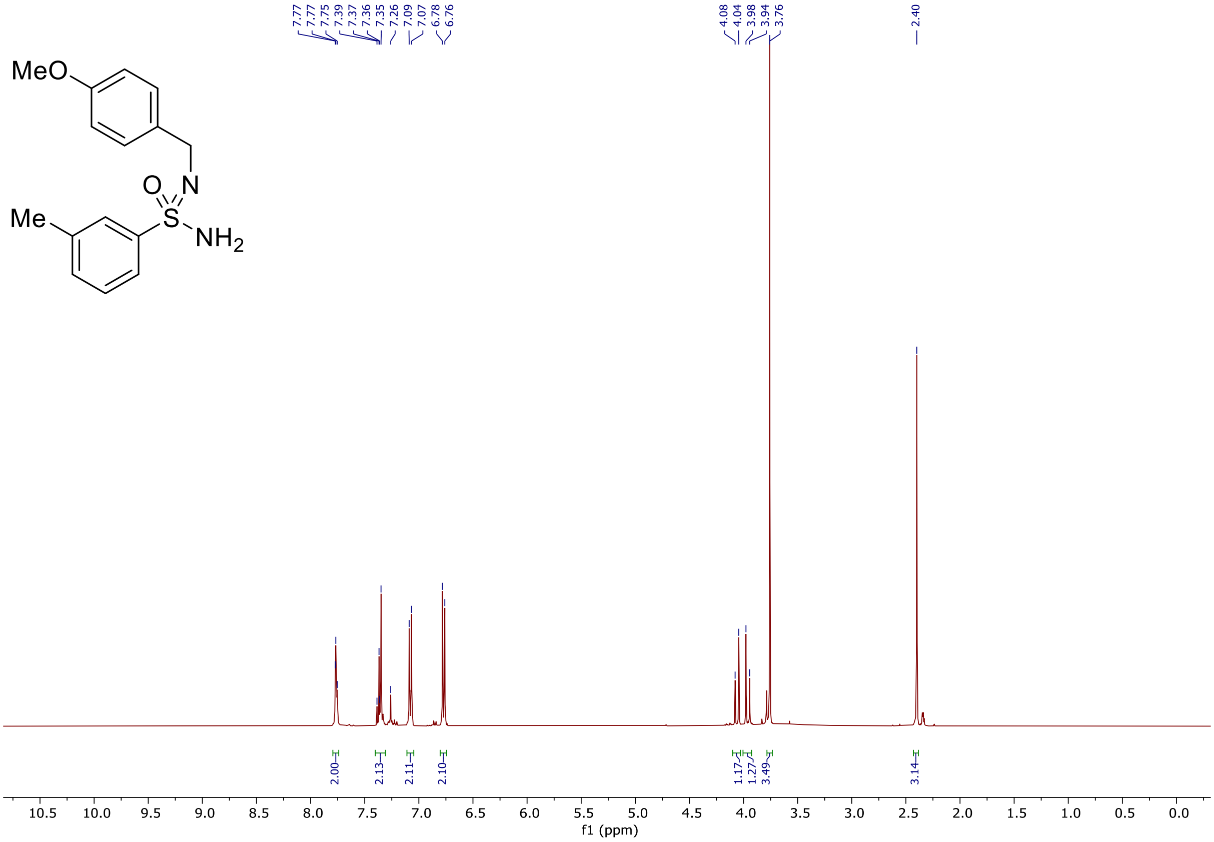

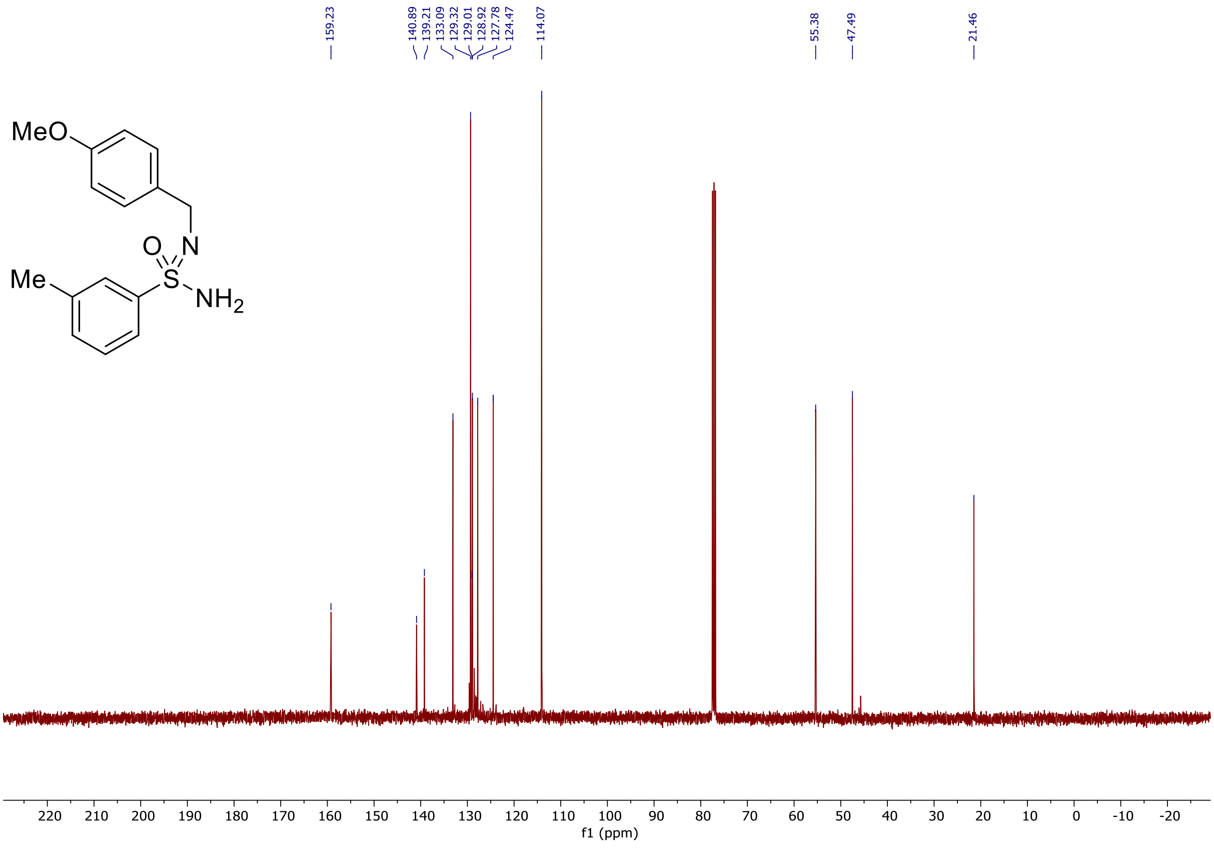

N'-(4-methoxybenzyl)-2-methylbenzenesulfonimidamide (11e)

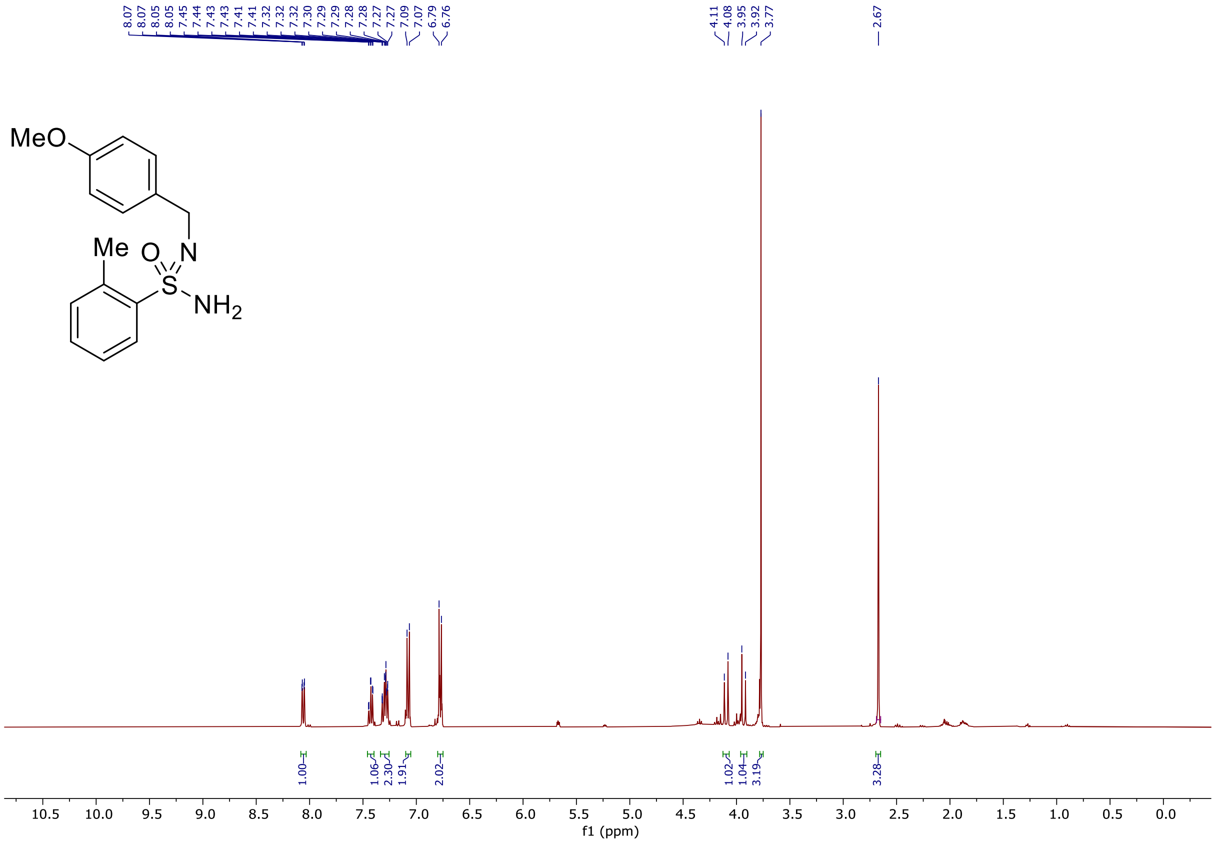

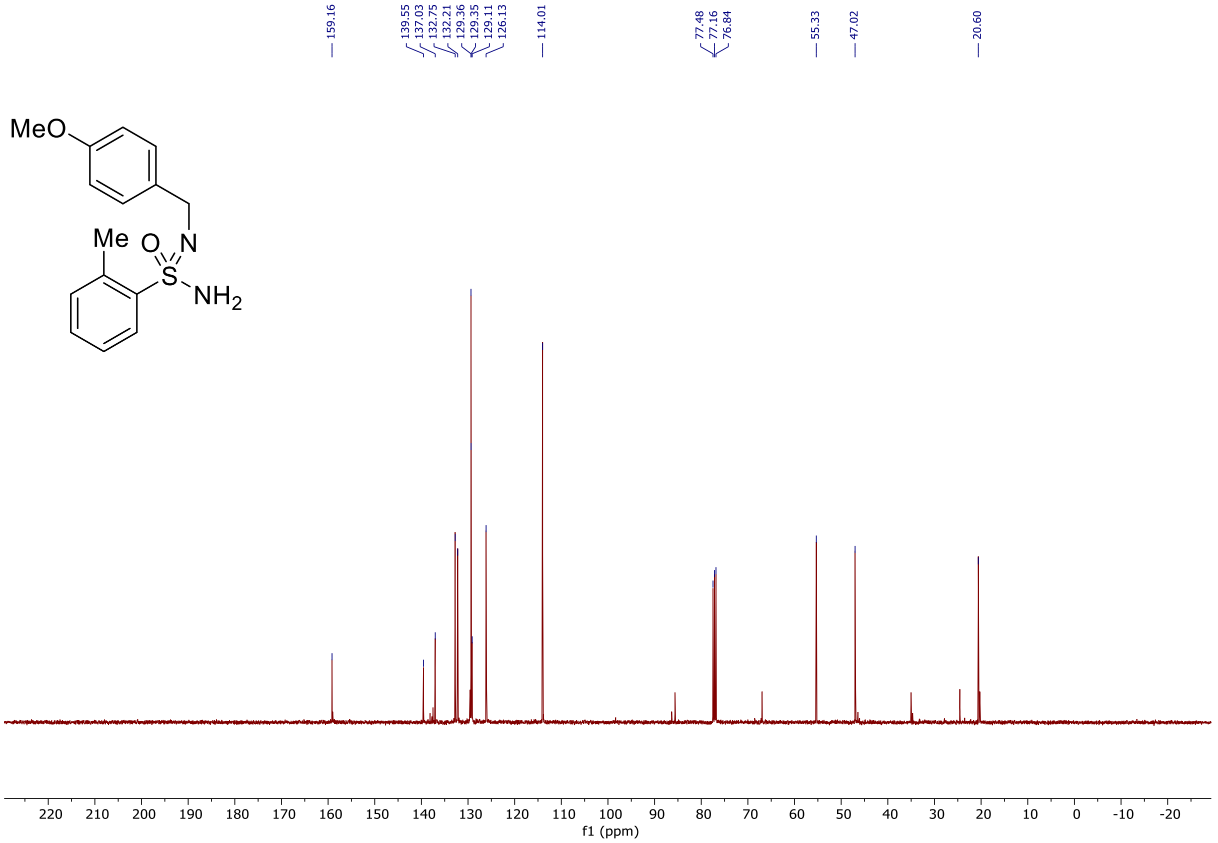

4-fluoro-*N*'-(4-methoxybenzyl)benzenesulfonimidamide (11f)

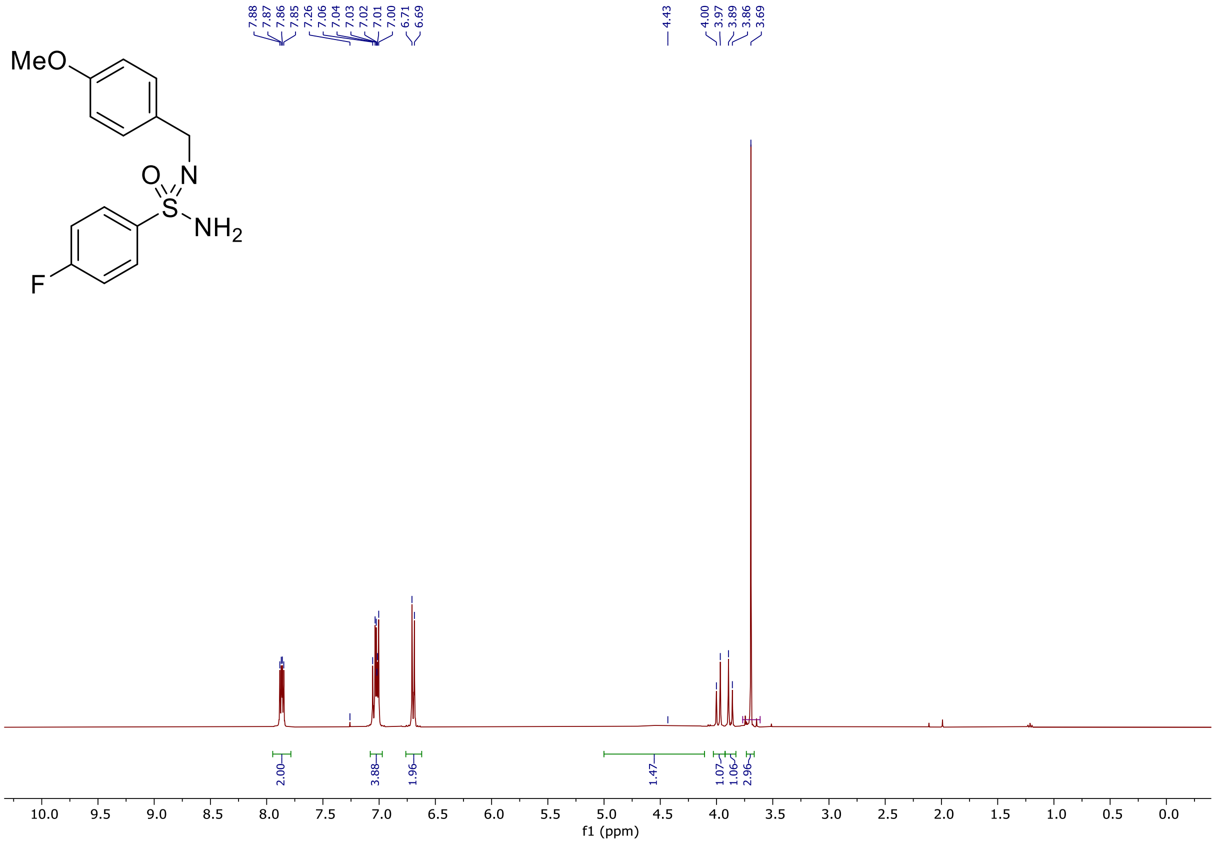

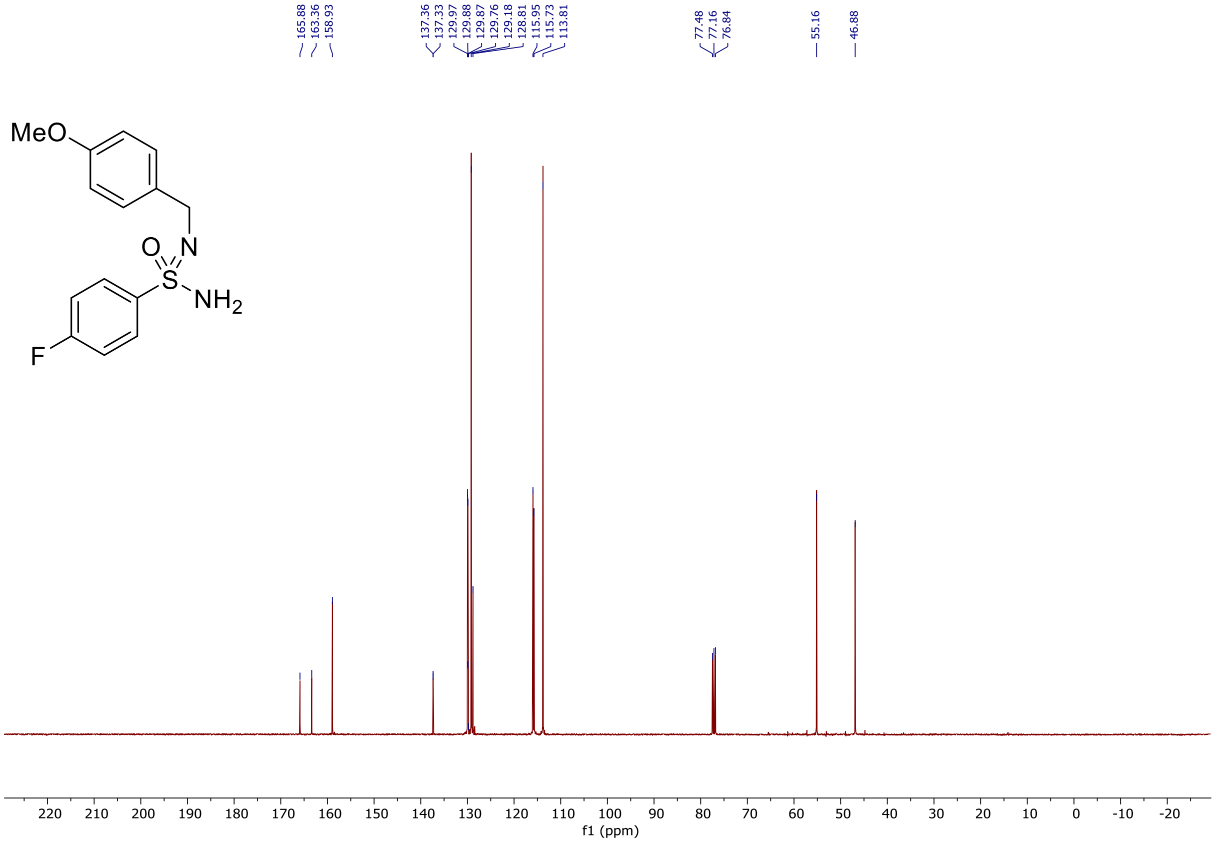

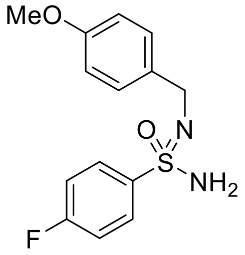

MeO
O
N
S
NH2
F

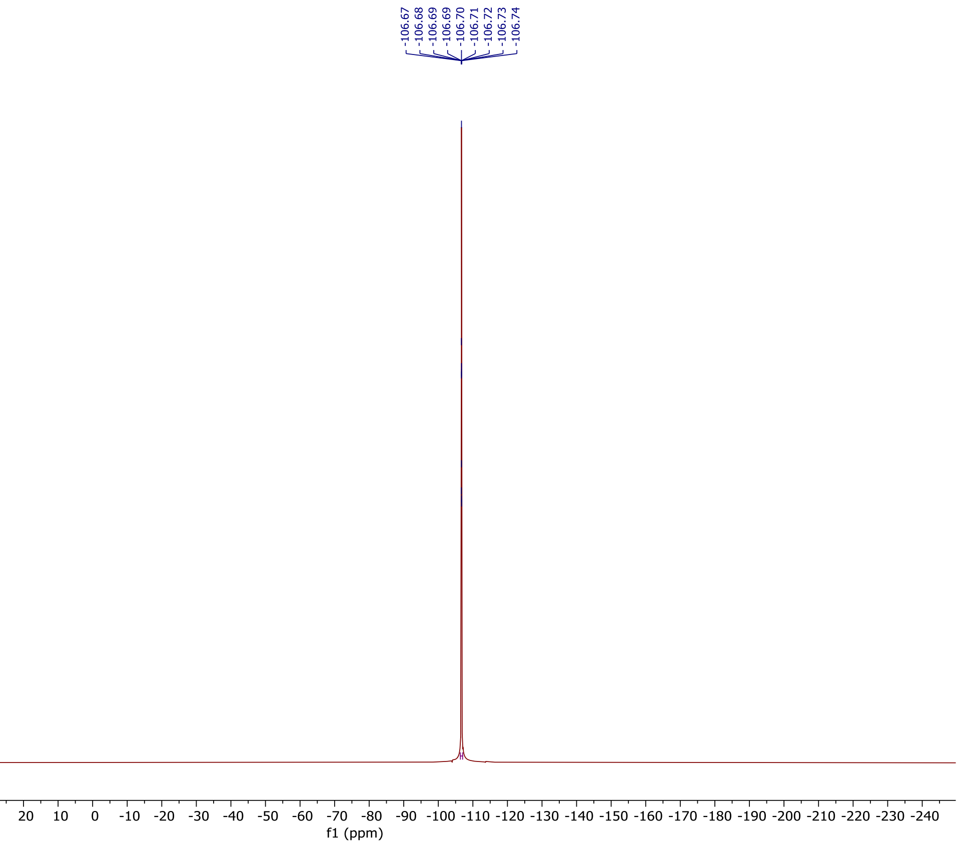

-106.67
-106.68
-106.69
-106.69
-106.70
-106.71
-106.72
-106.73
-106.74
90 80 70 60 50 40 30 20 10 0 -10 -20 -30 -40 -50 -60 -70 -80 -90 -100 -110 -120 -130 -140 -150 -160 -170 -180 -190 -200 -210 -220 -230 -240
f1 (ppm)

N'-(4-methoxybenzyl)thiophene-2-sulfonimidamide (11g)

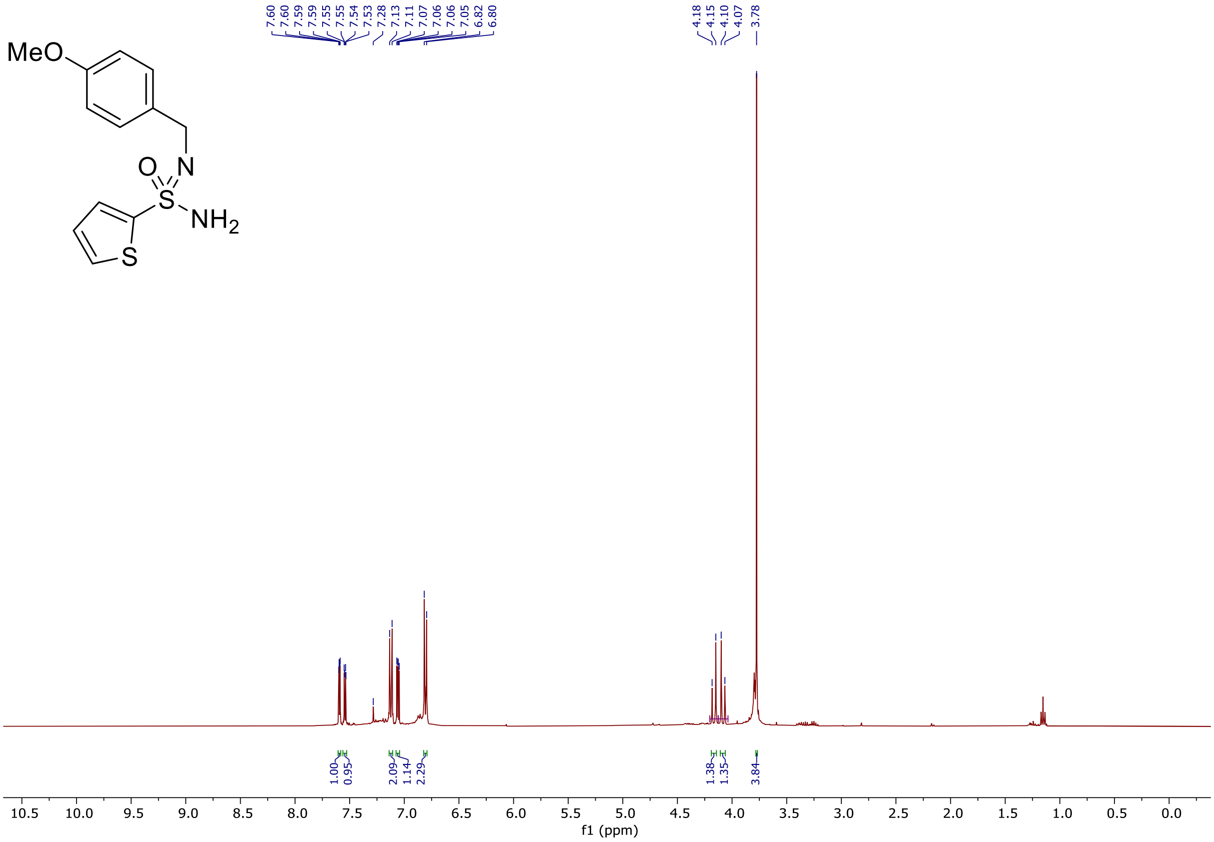

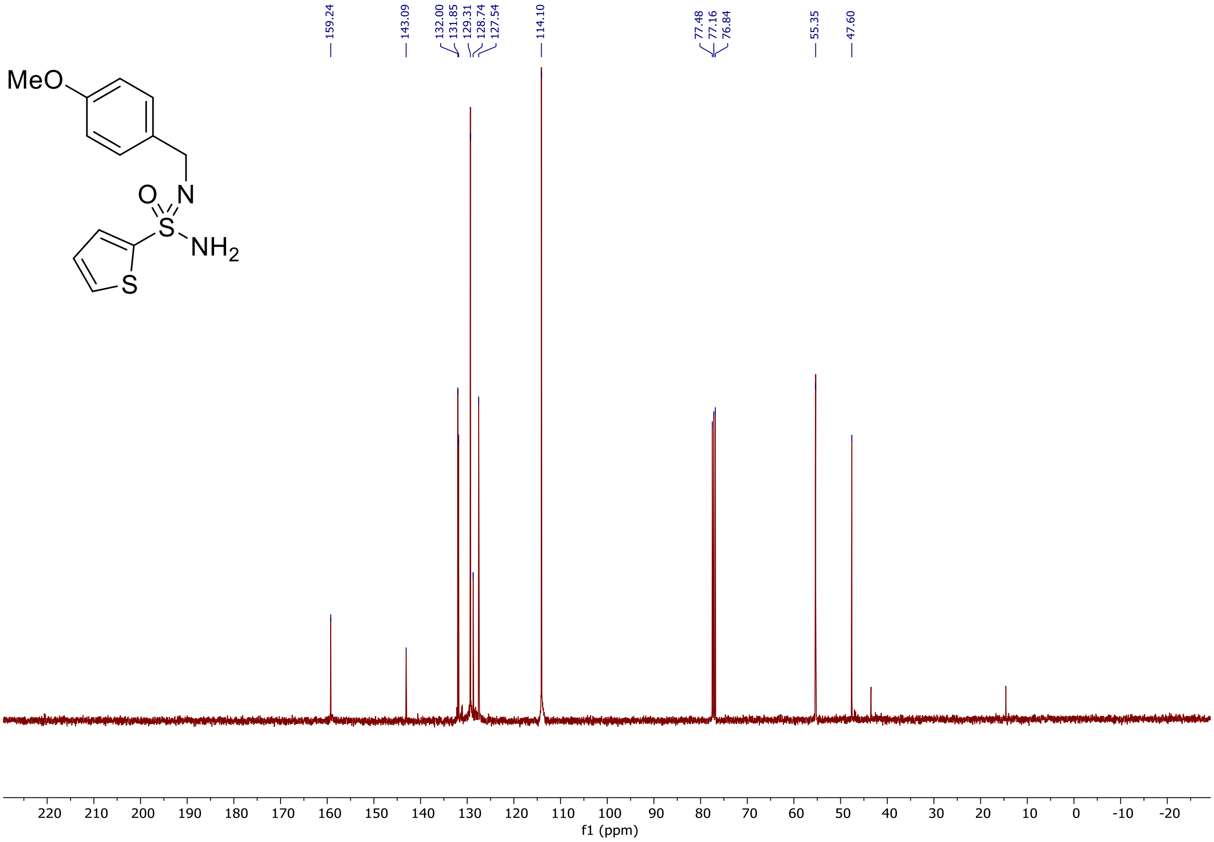

***N'*-(4-methoxybenzyl)-4-(5-(p-tolyl)-3-(trifluoromethyl)-1H-pyrazol-1-yl)benzenesulfonimidamide (11h)**

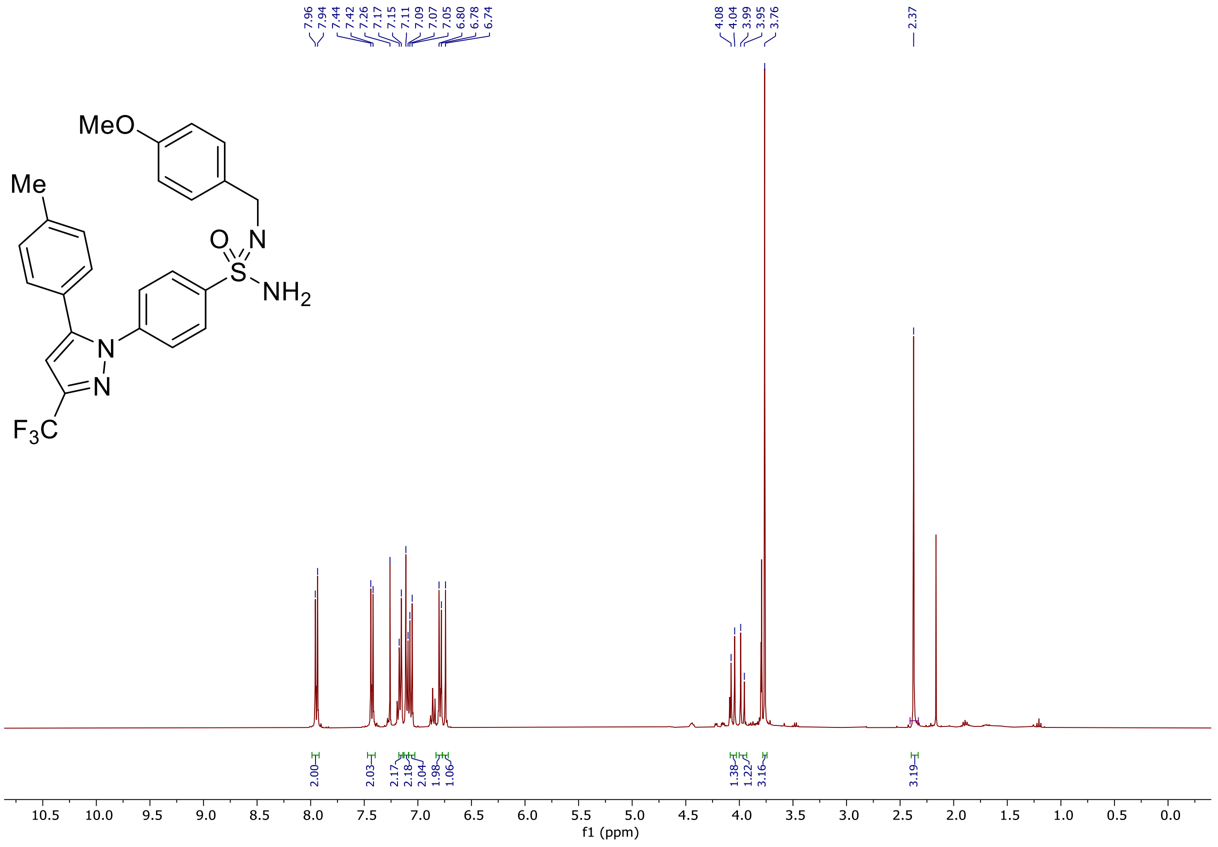

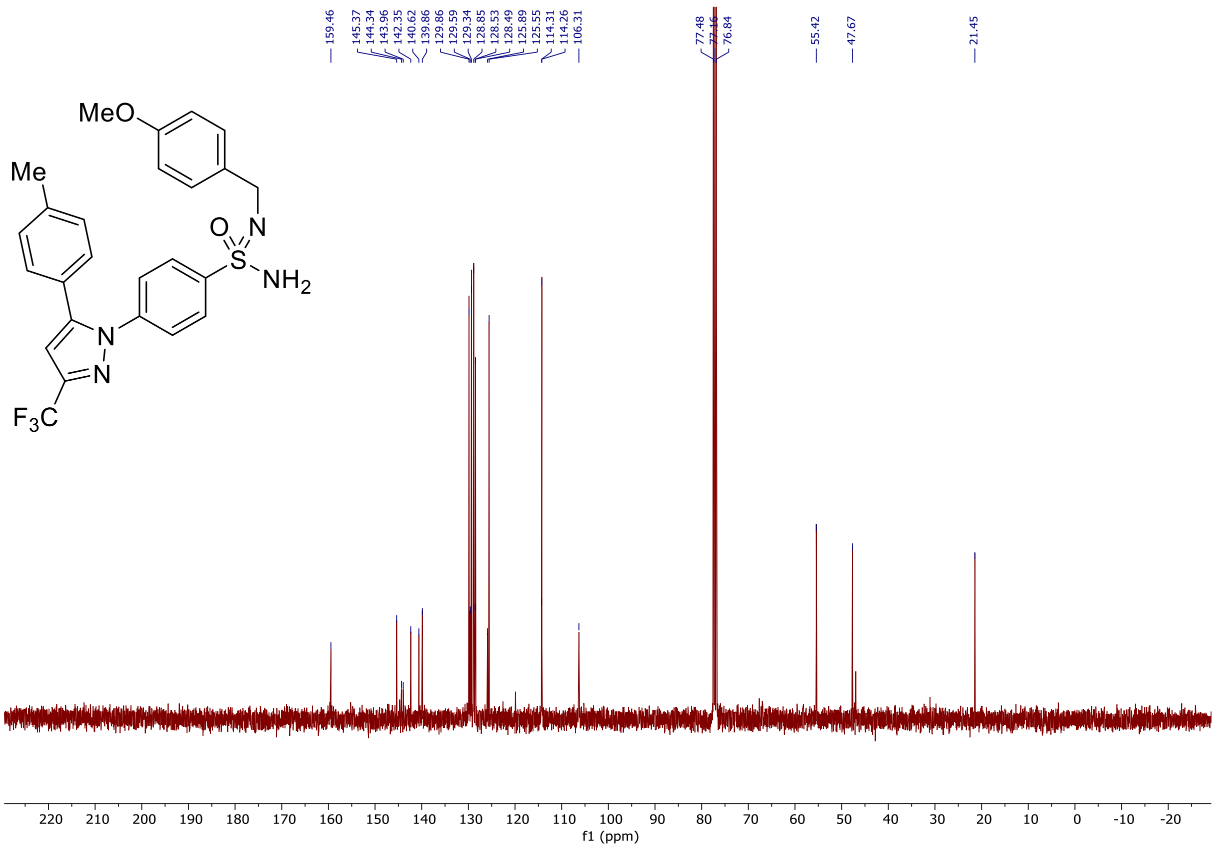

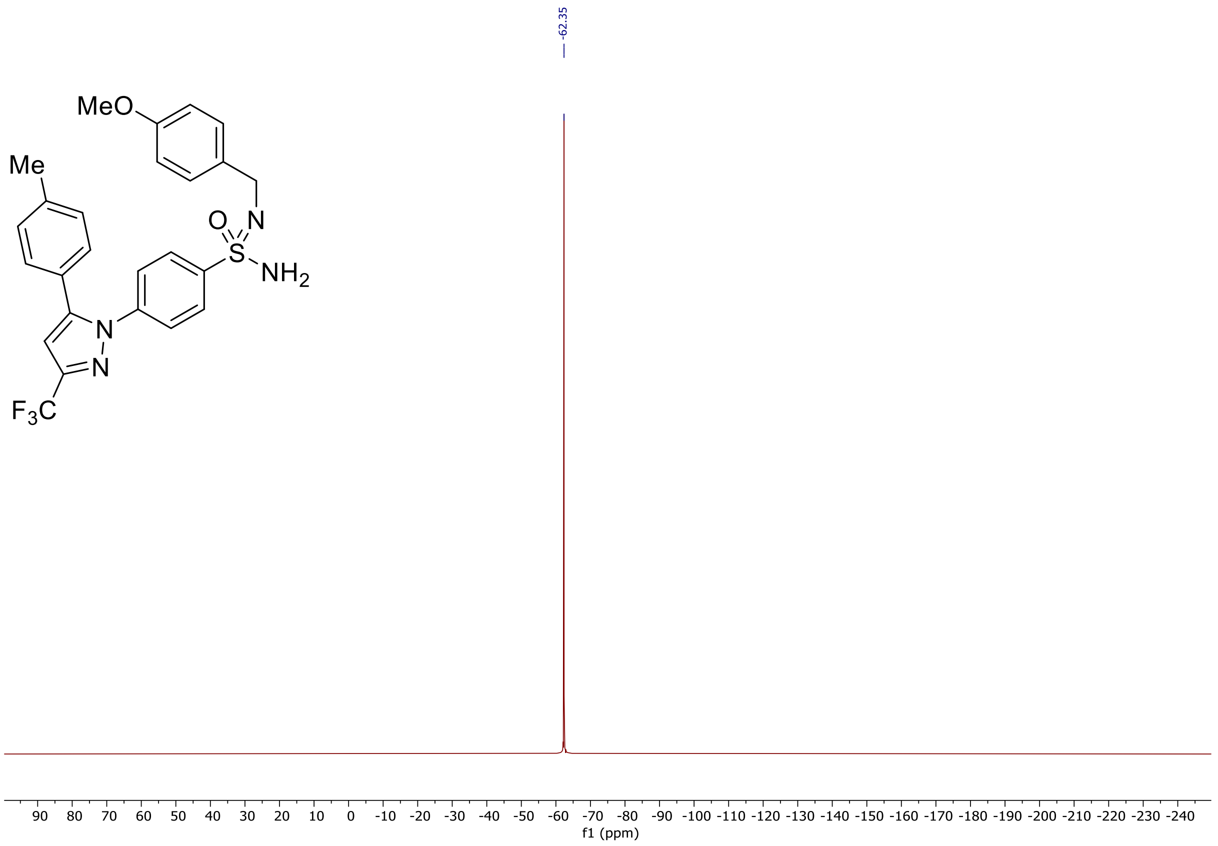
-62.35
MeO
Me
O
N
S
NH2
N
N
F3C
90 80 70 60 50 40 30 20 10 0 -10 -20 -30 -40 -50 -60 -70 -80 -90 -100 -110 -120 -130 -140 -150 -160 -170 -180 -190 -200 -210 -220 -230 -240
f1 (ppm)

N'-(4-methoxybenzyl)-2-methylprop-1-ene-1-sulfonimidamide (11i)

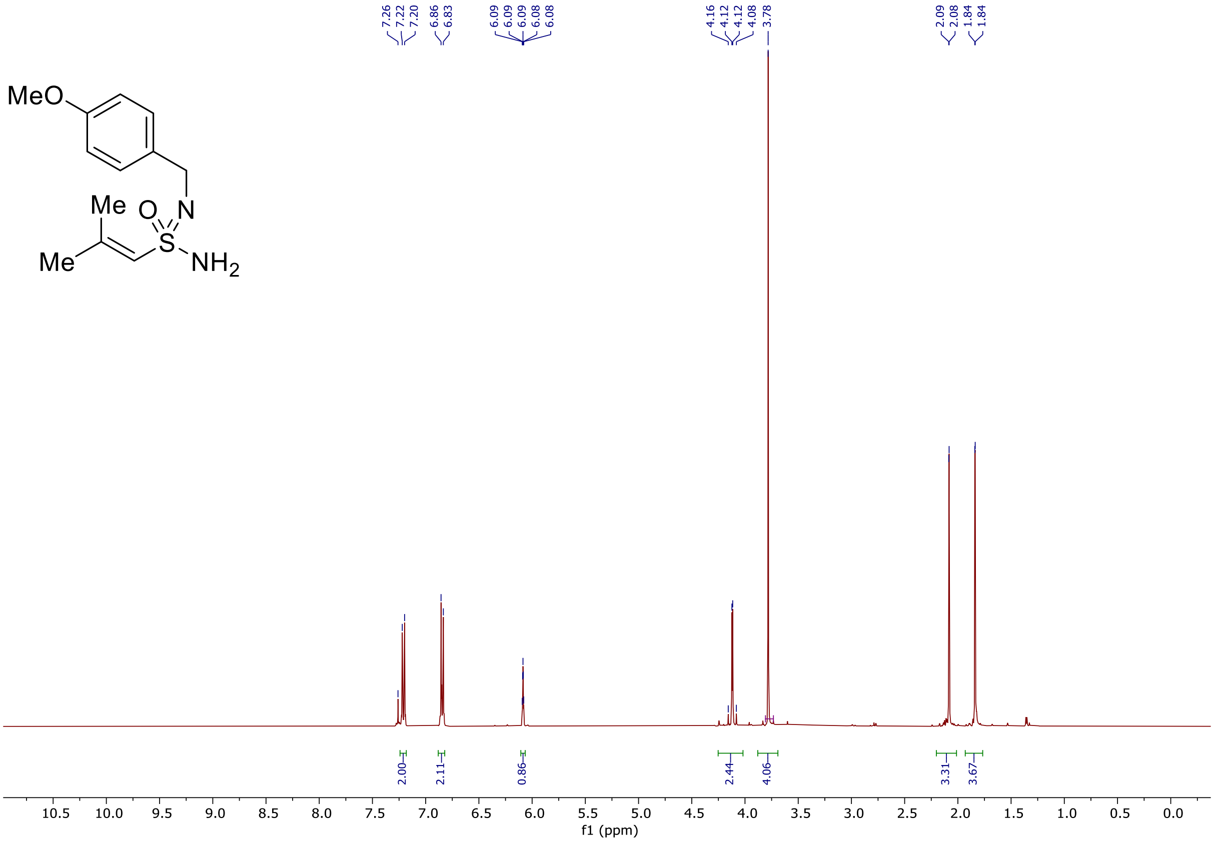

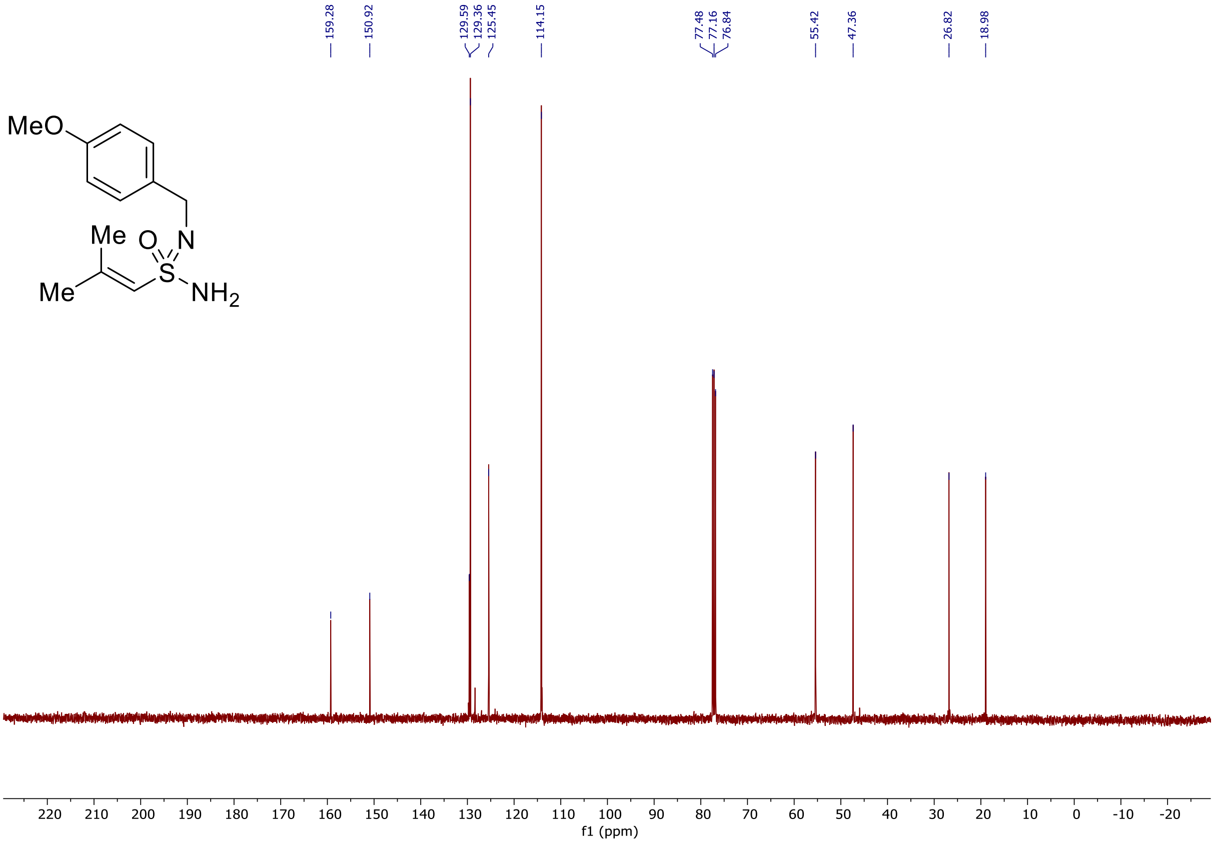

N'-(4-methoxybenzyl)methanesulfonimidamide (11j)

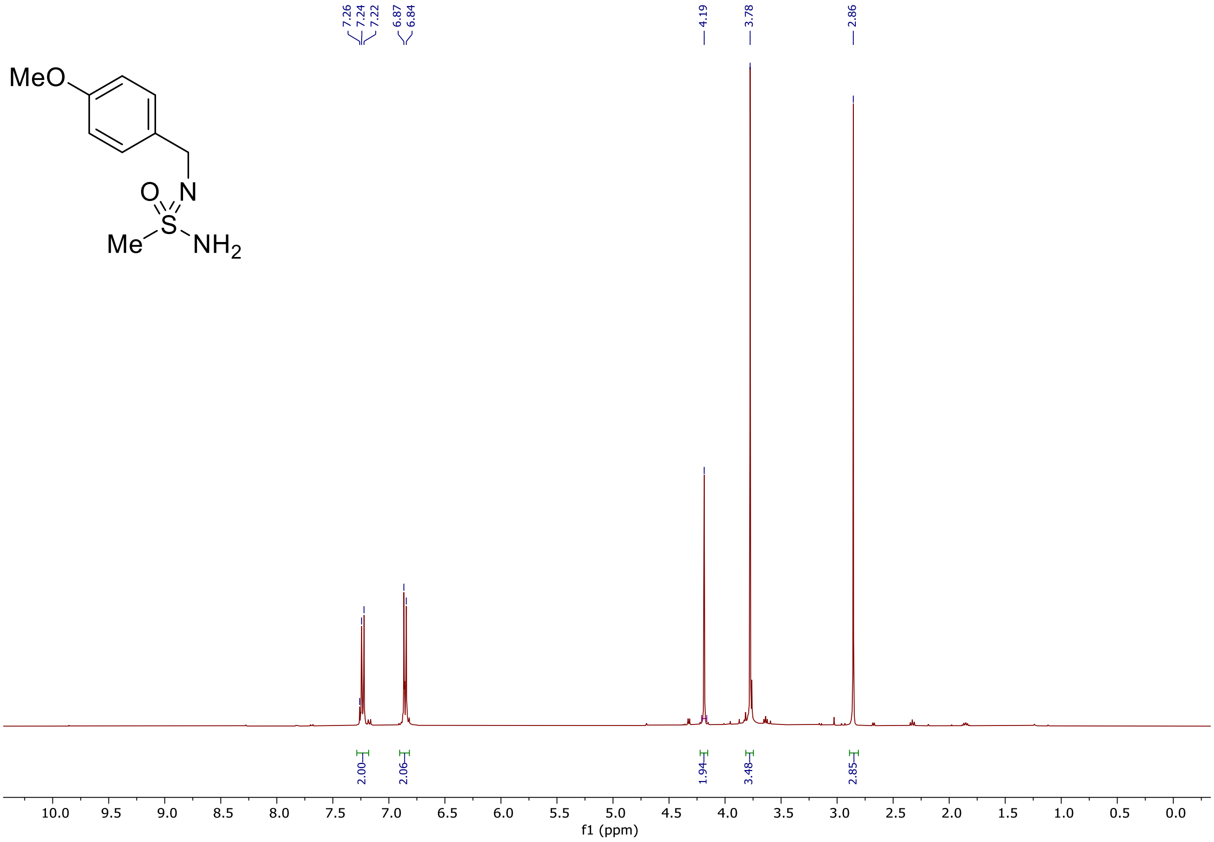

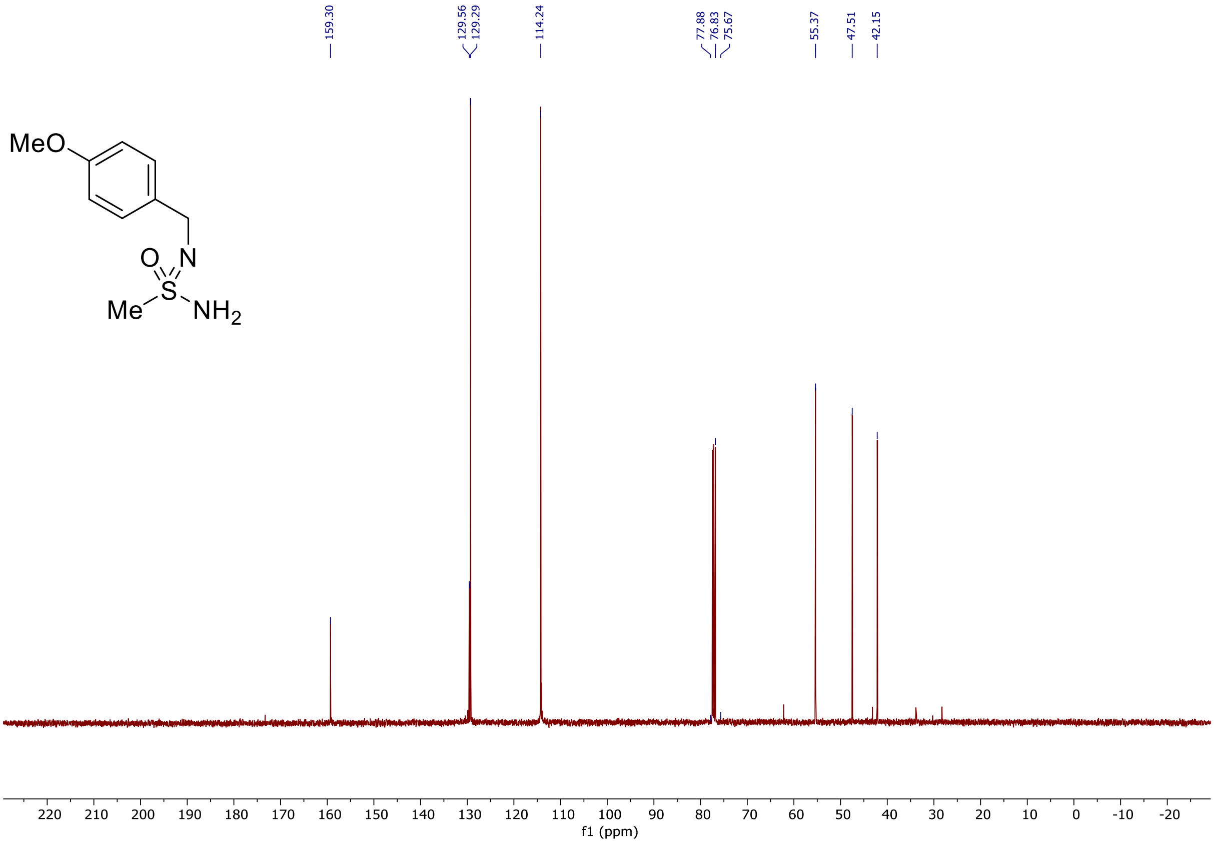

N,N'-bis(4-methoxybenzyl)-[1,1'-biphenyl]-4-sulfonimidamide (2a)

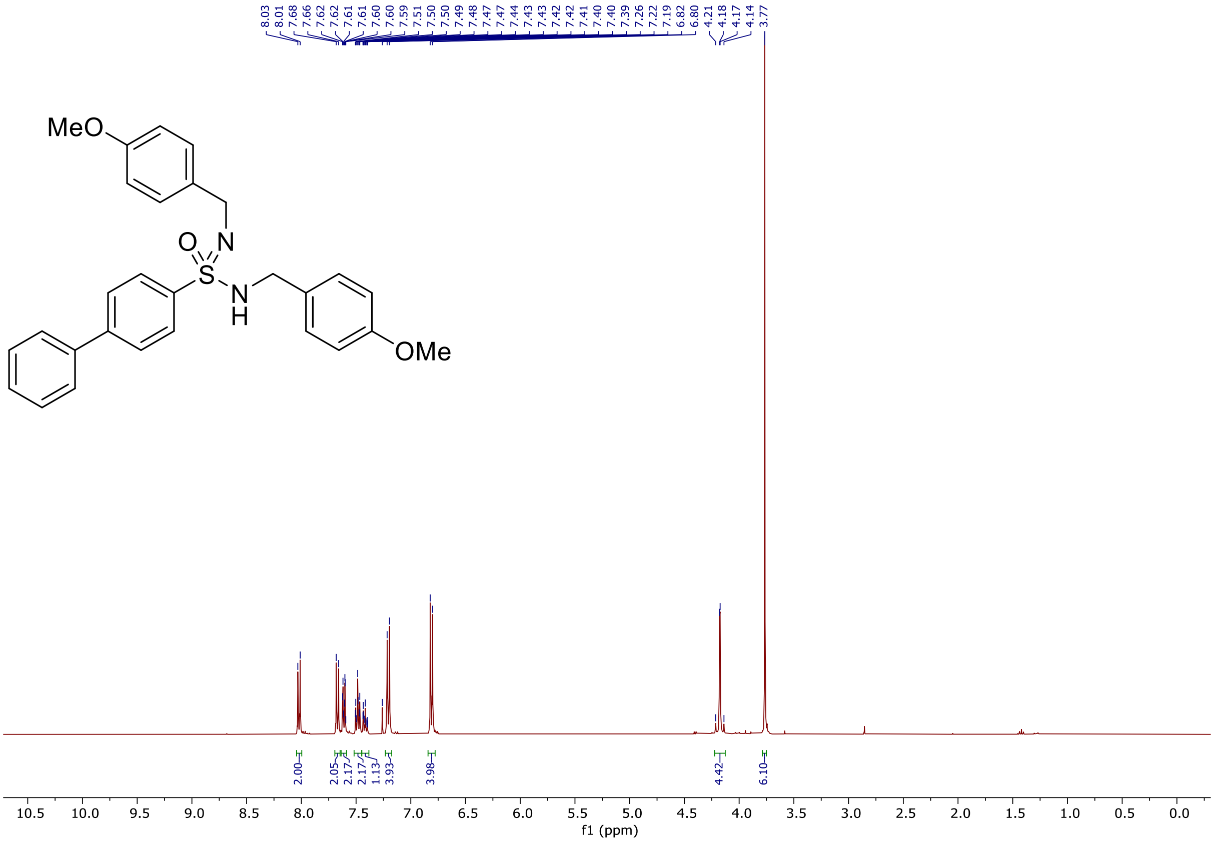

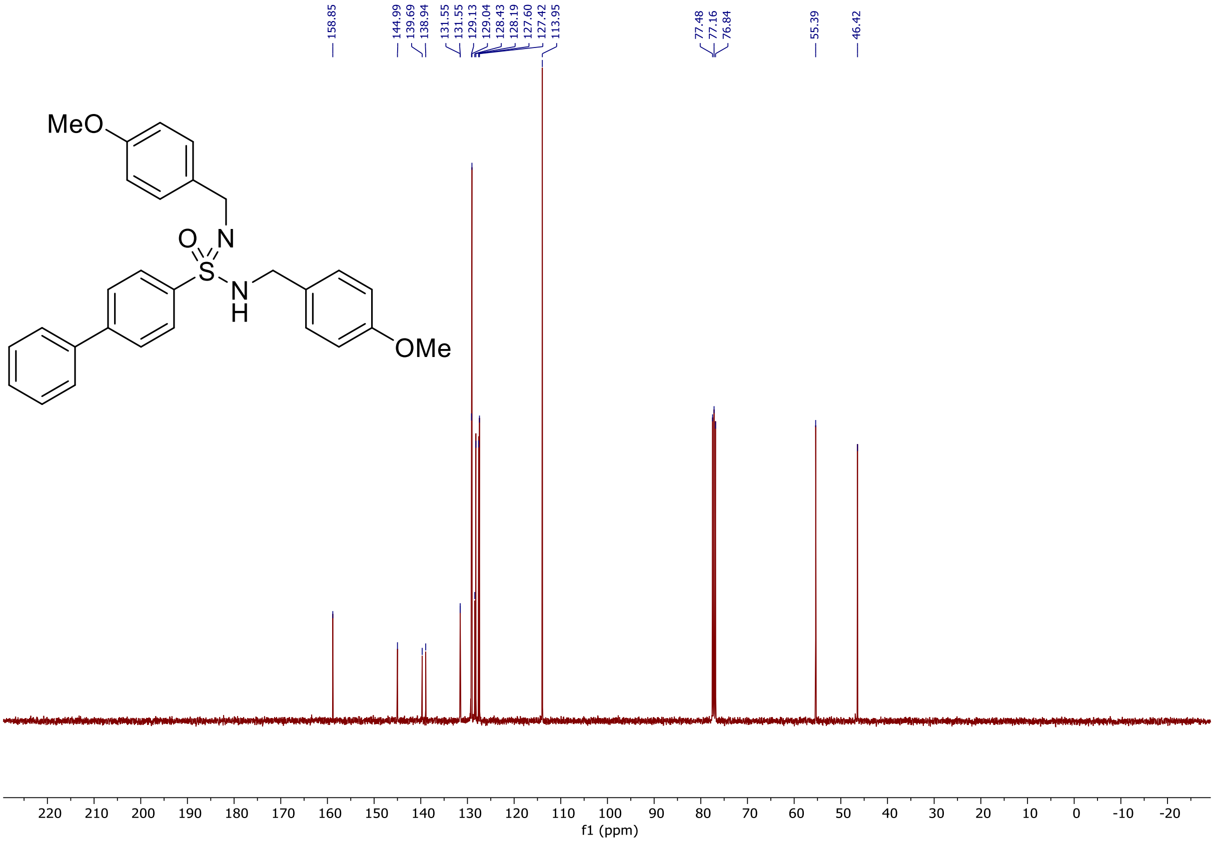

N,N'-bis(4-methoxybenzyl)-4-methylbenzenesulfonimidamide (2b)

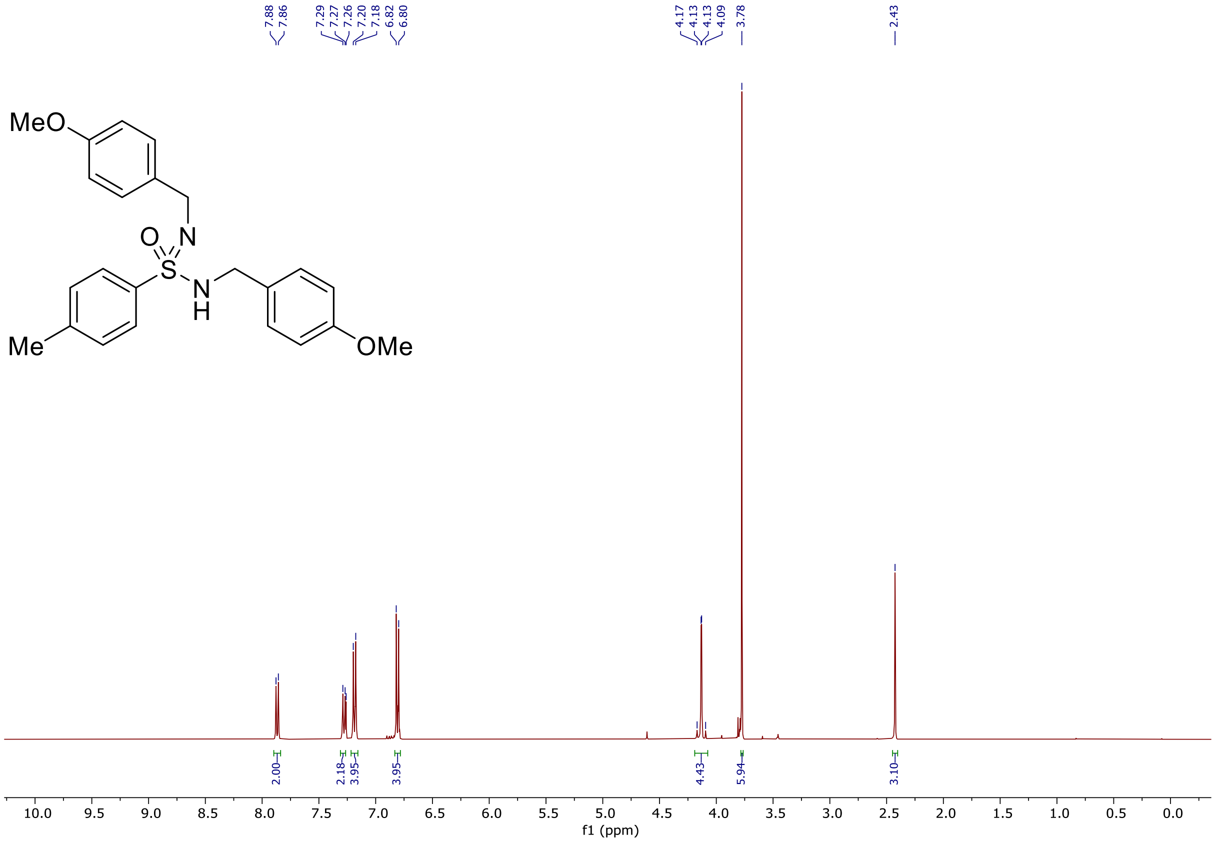

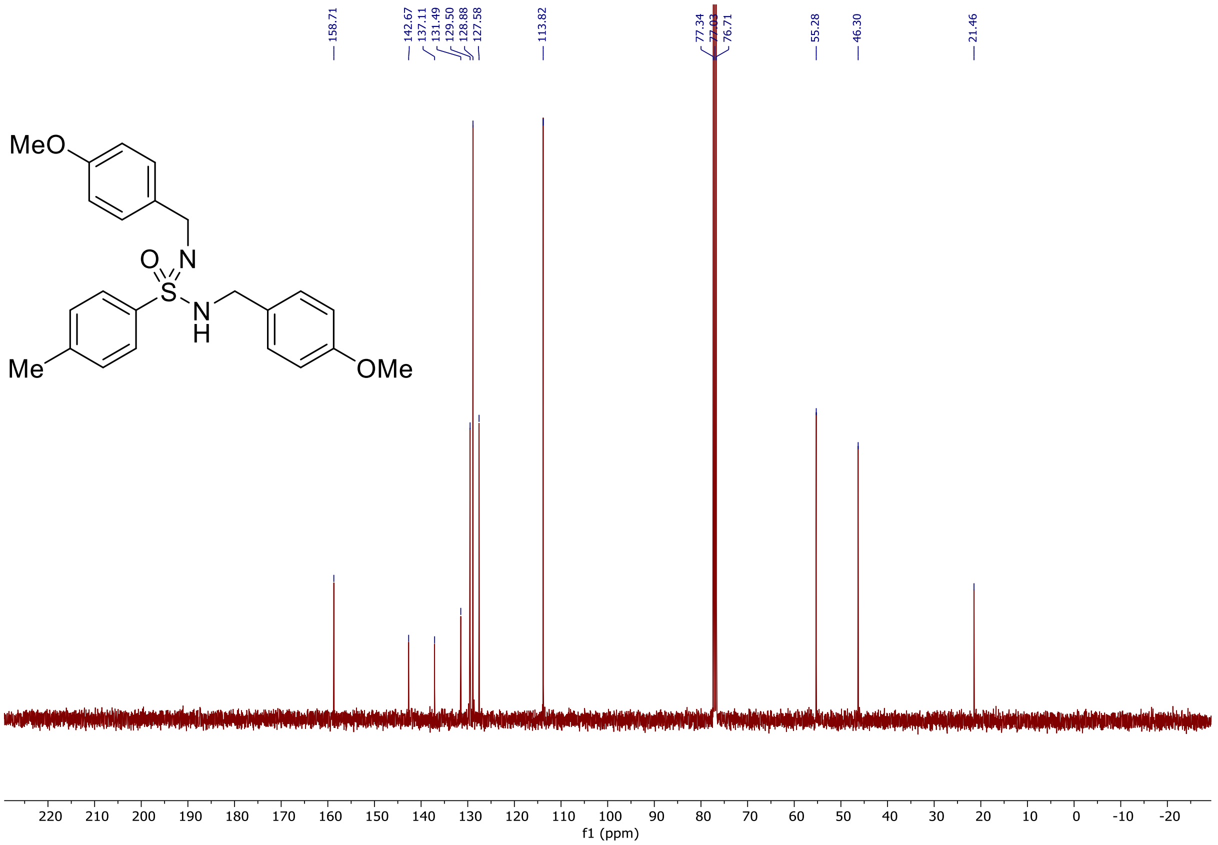

_N_,_N'_-bis(4-methoxybenzyl)benzenesulfonimidamide (2c)

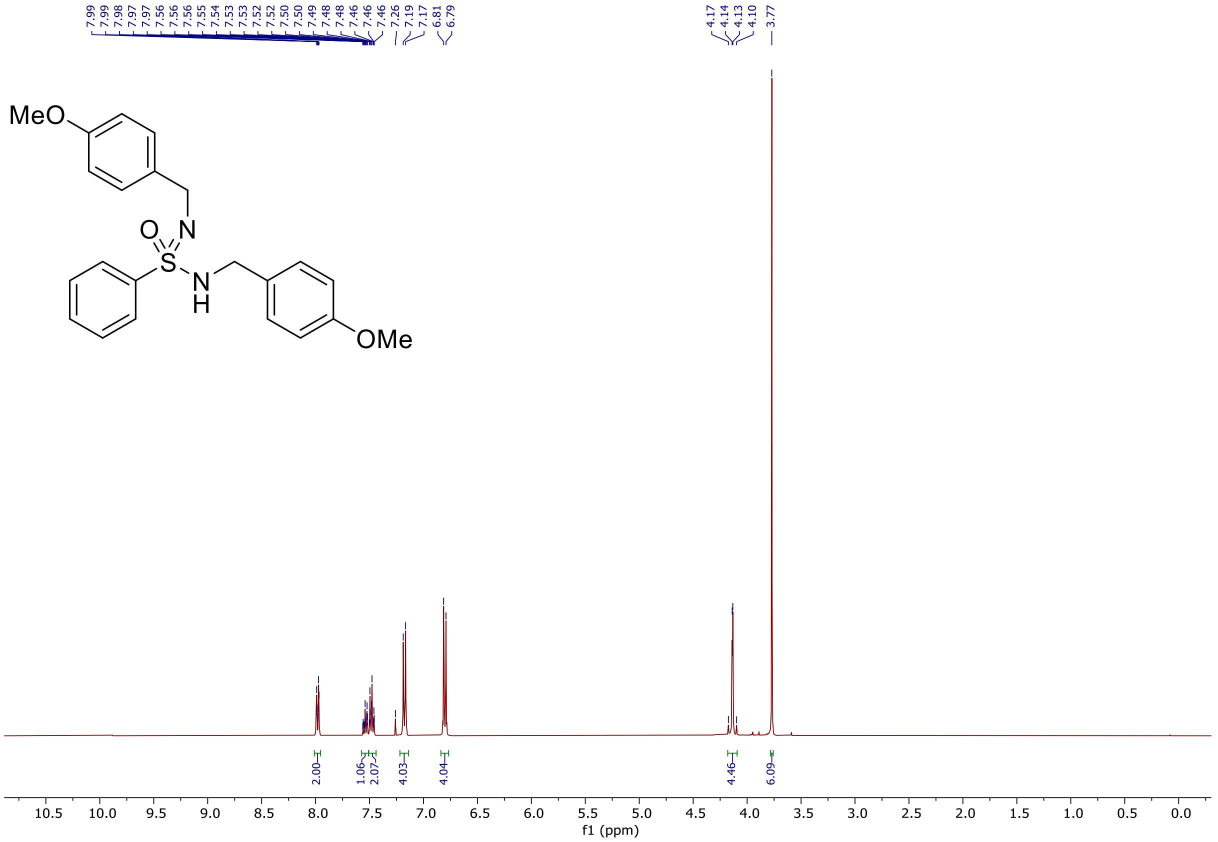

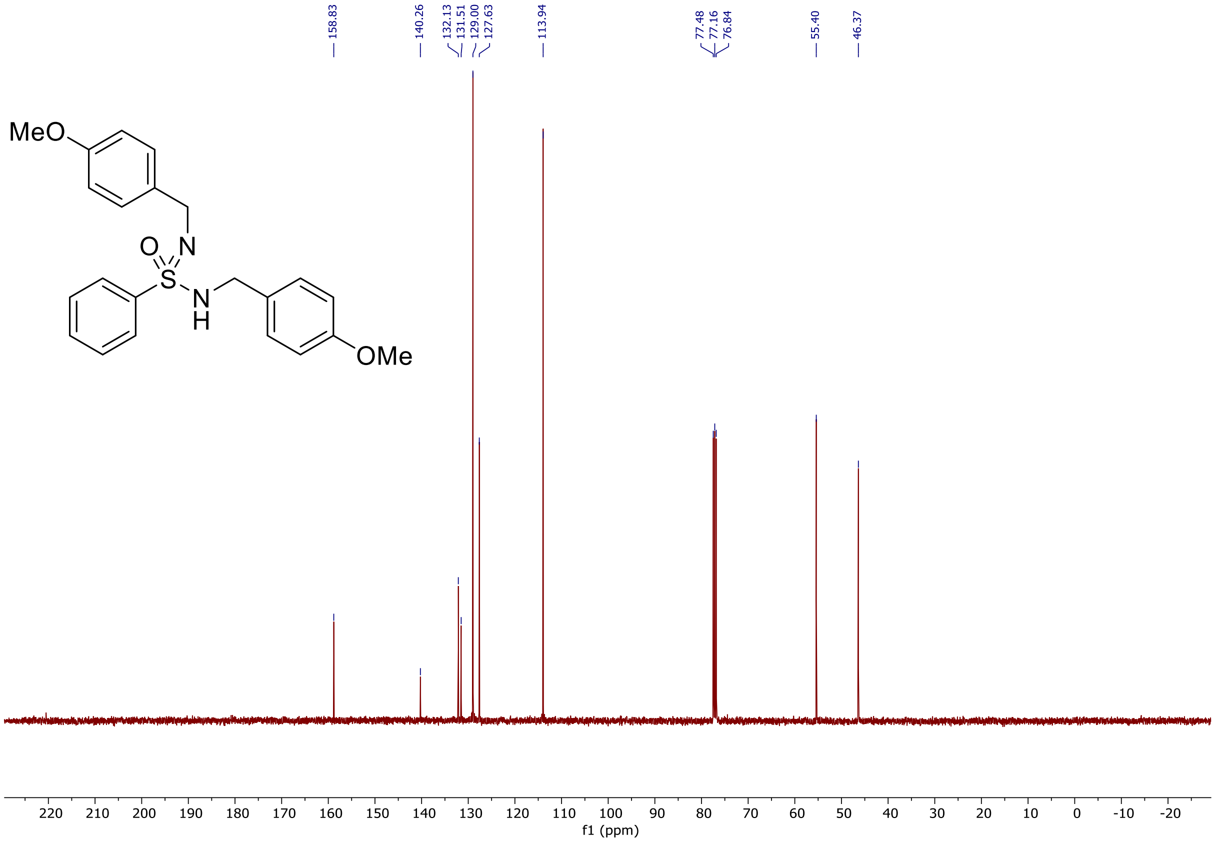

N,*N'*-bis(4-methoxybenzyl)-3-methylbenzenesulfonimidamide (2d)

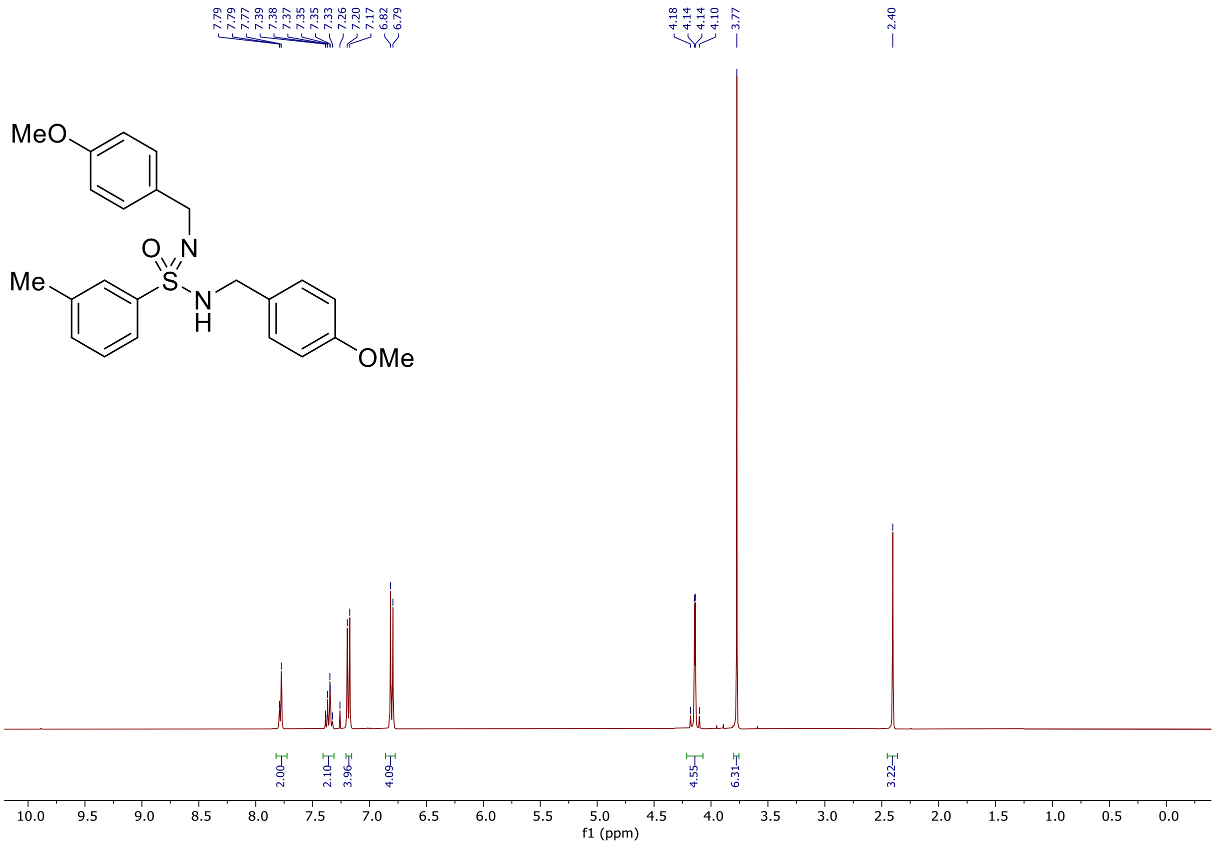

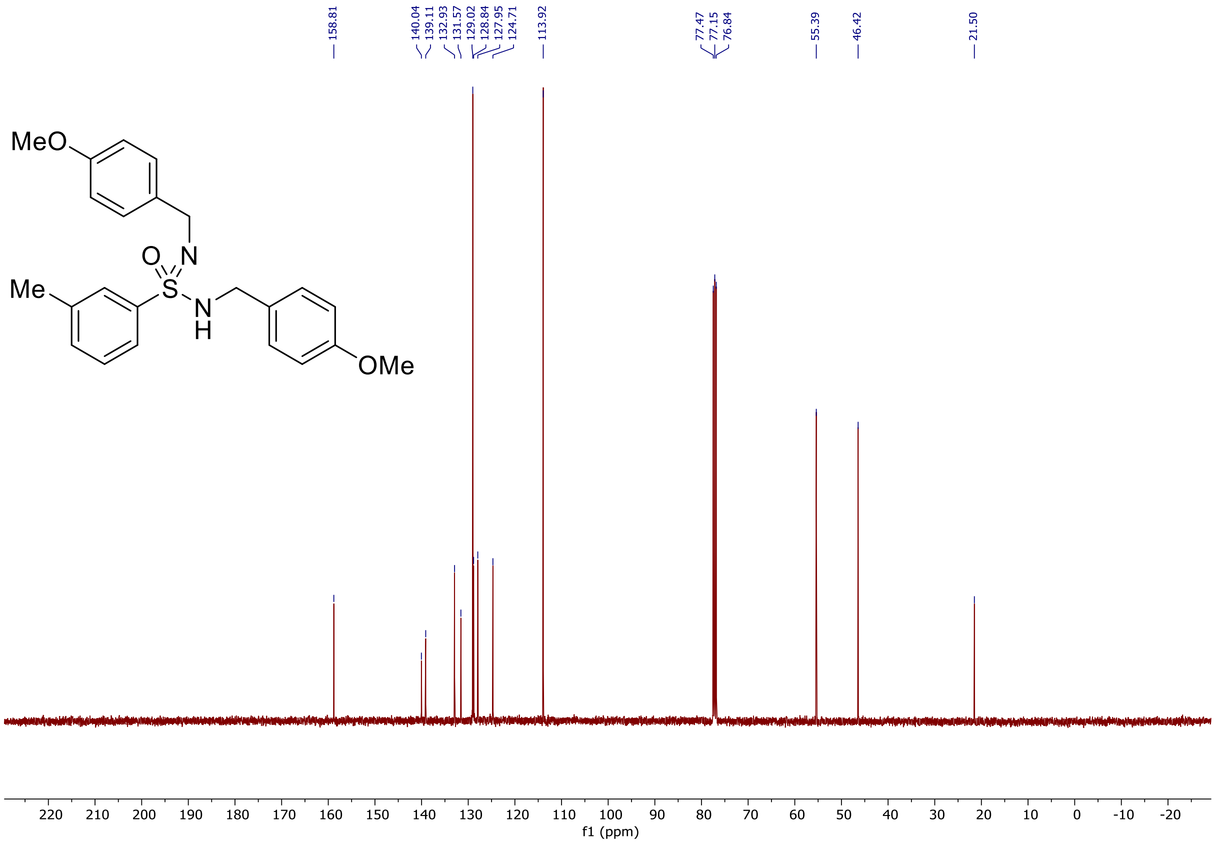

***N,N'*-bis(4-methoxybenzyl)-2-methylbenzenesulfonimidamide (2e)**

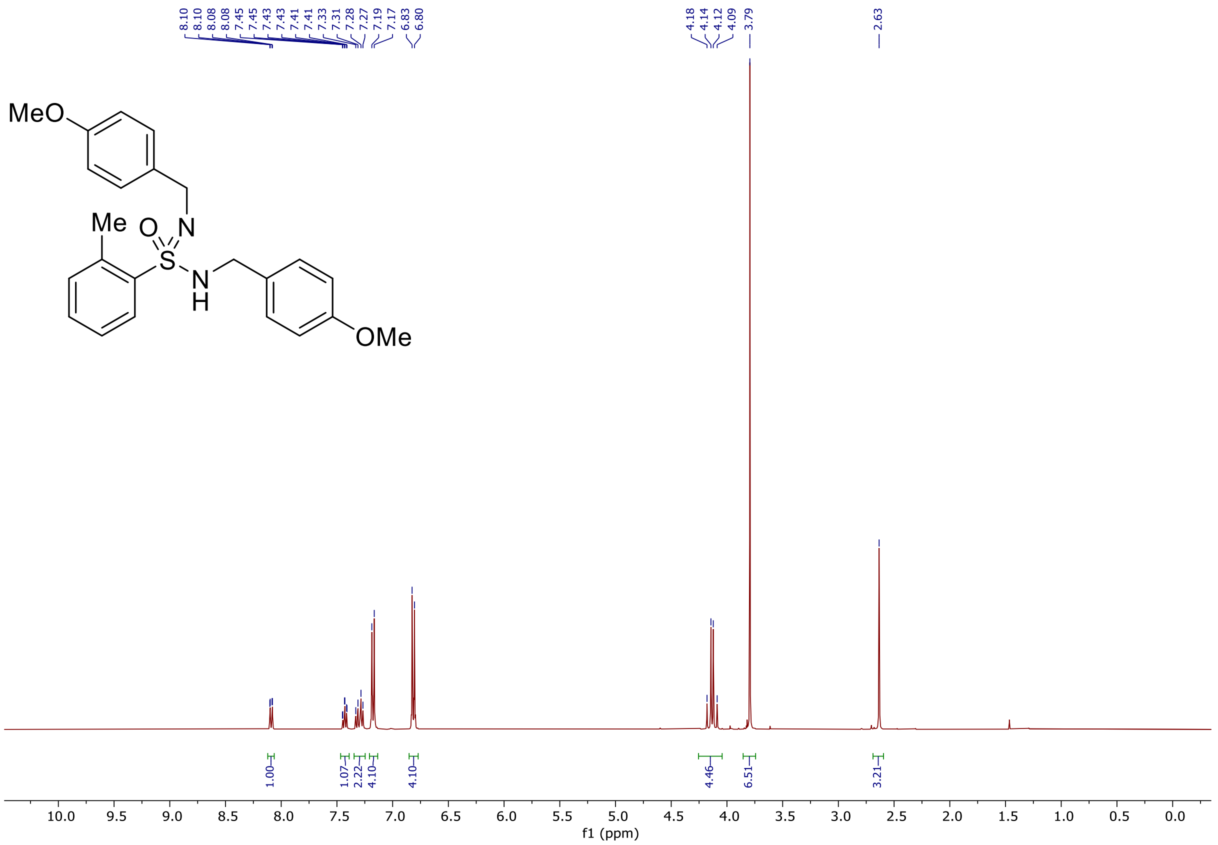

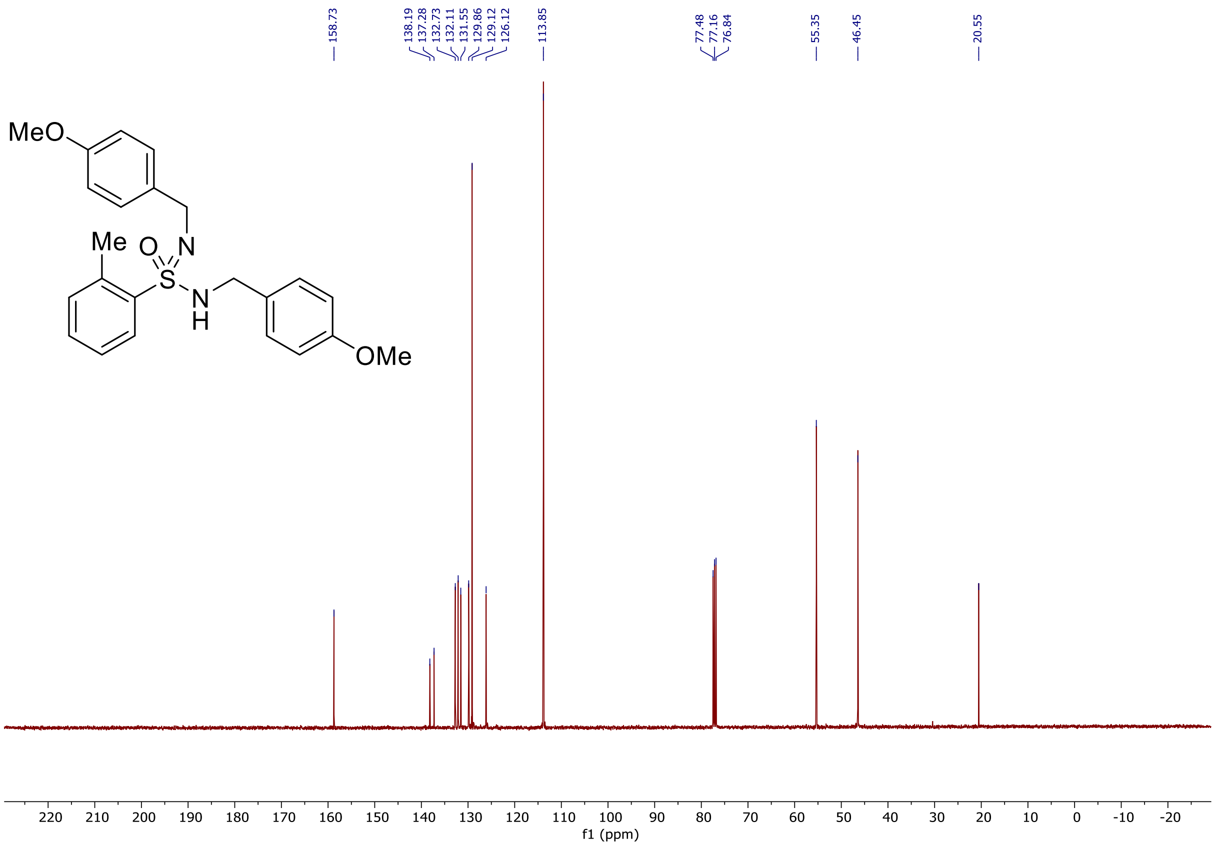

4-fluoro-*N*,*N'*-bis(4-methoxybenzyl)benzenesulfonimidamide (2f)

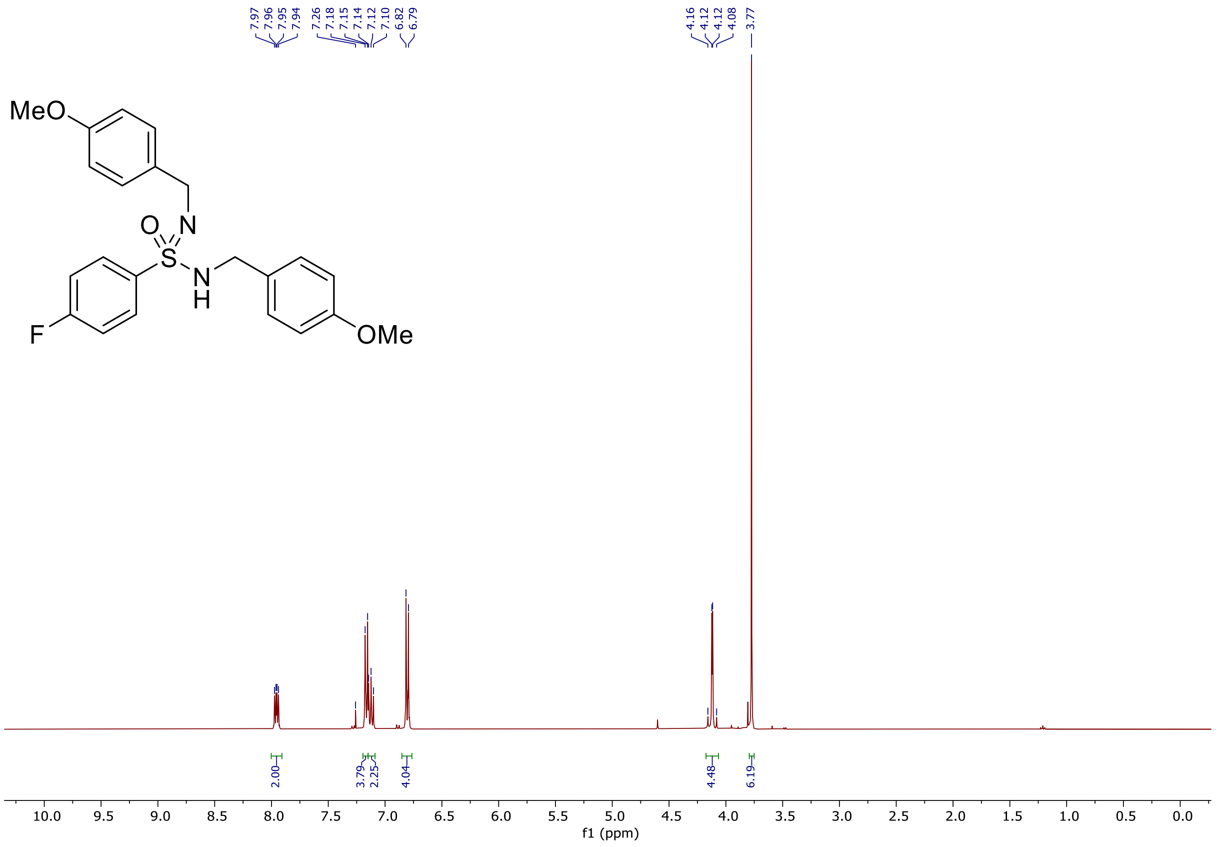

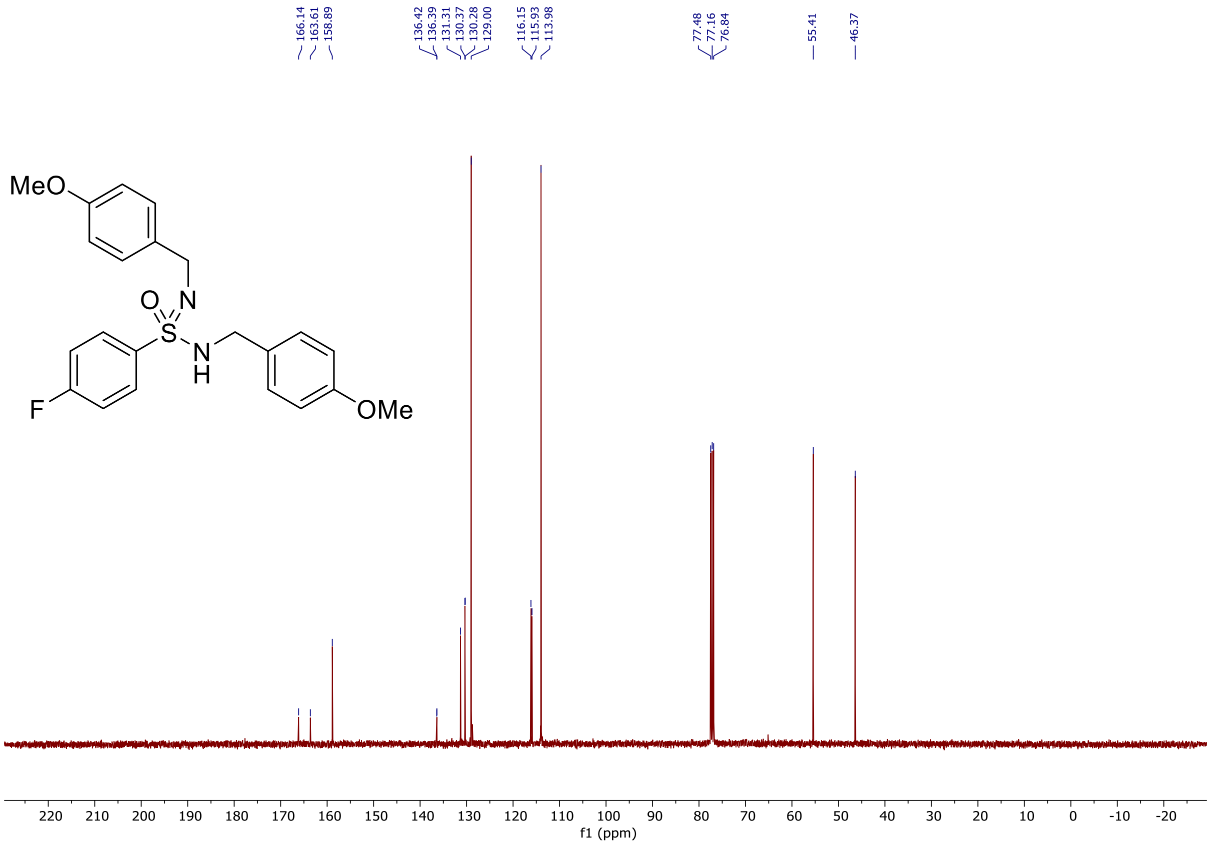

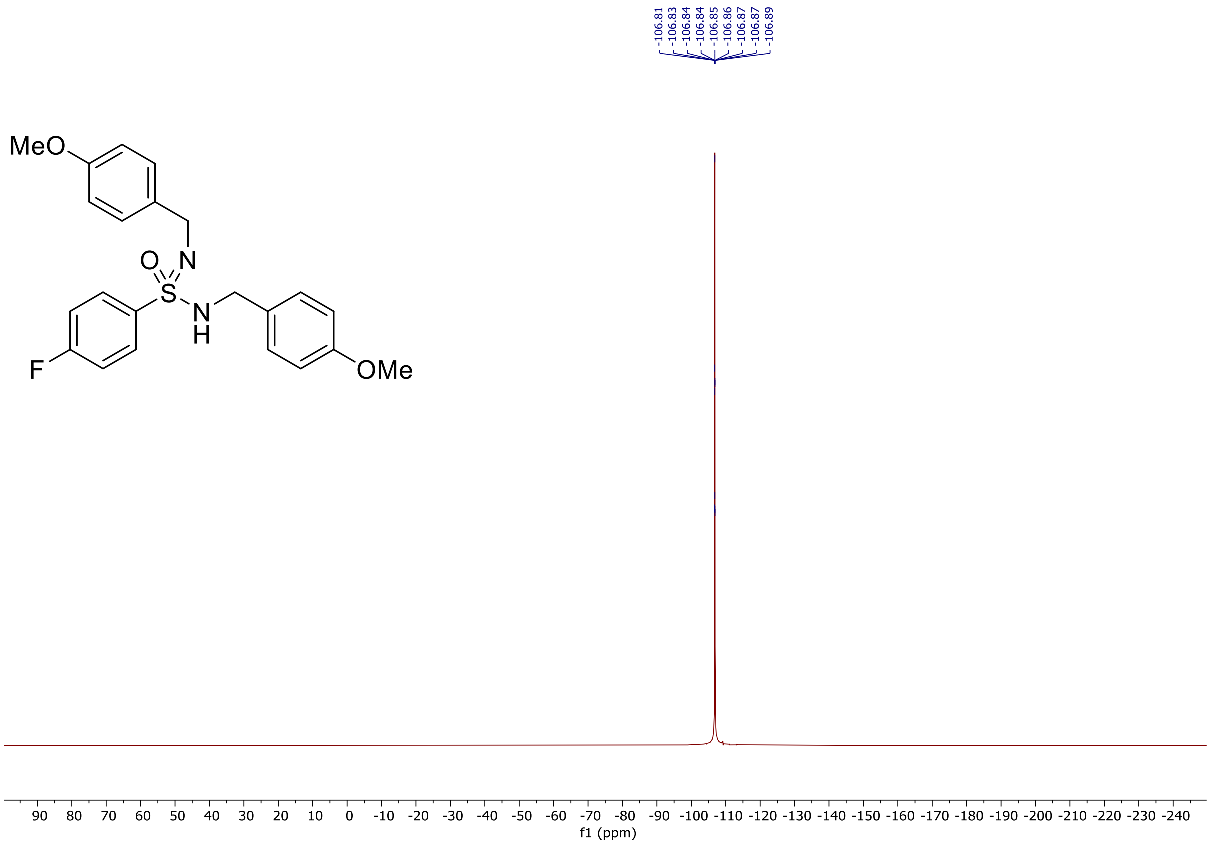
-106.81
-106.83
-106.84
-106.84
-106.85
-106.86
-106.87
-106.87
-106.89
MeO
O
N
S
N
H
F
OMe
90 80 70 60 50 40 30 20 10 0 -10 -20 -30 -40 -50 -60 -70 -80 -90 -100 -110 -120 -130 -140 -150 -160 -170 -180 -190 -200 -210 -220 -230 -240
f1 (ppm)

N,*N'*-bis(4-methoxybenzyl)thiophene-2-sulfonimidamide (2g)

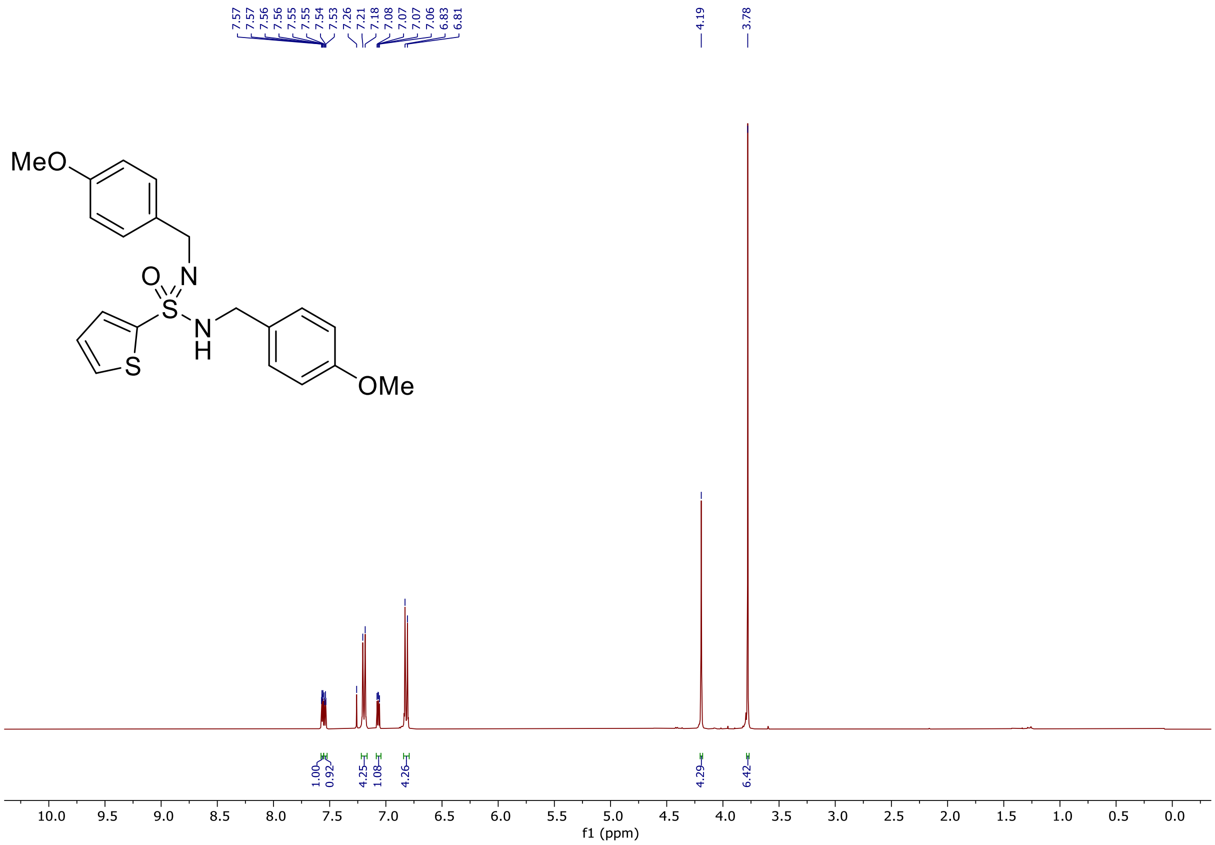

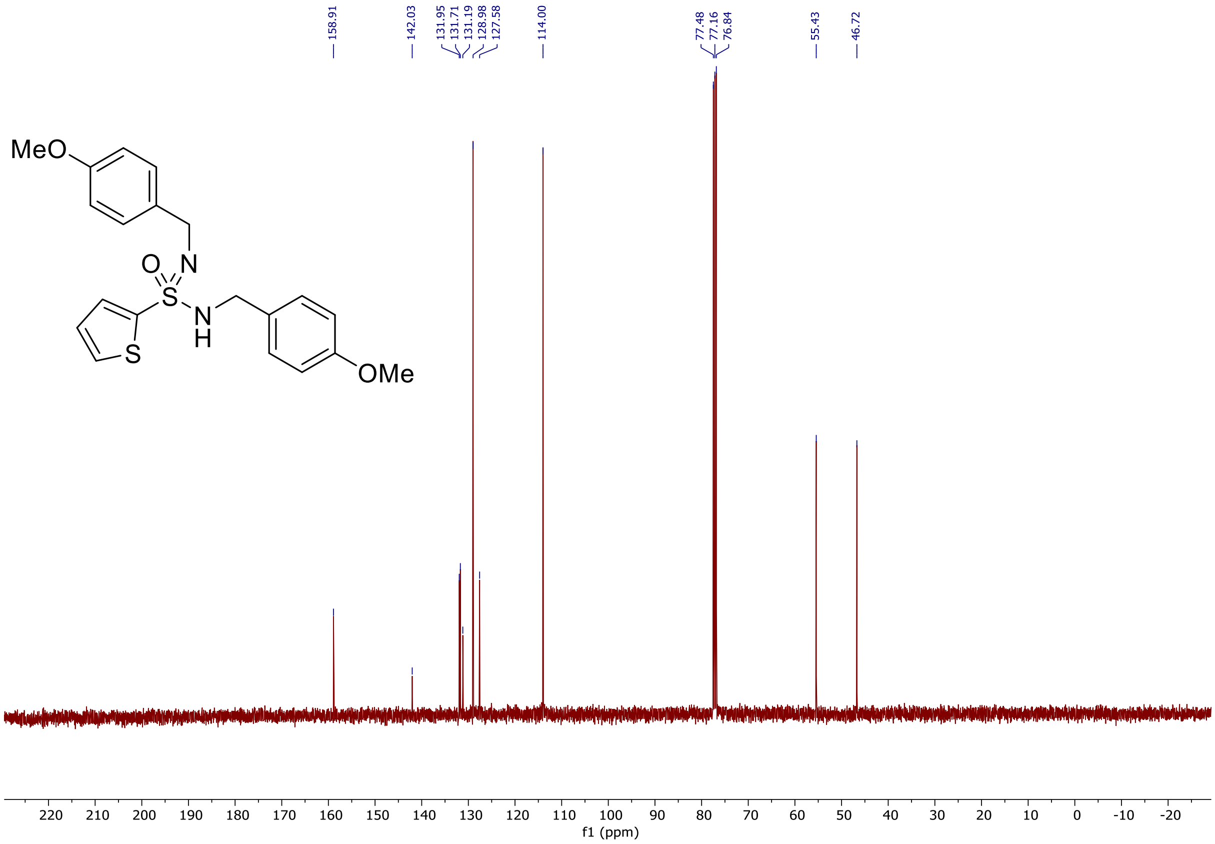

_N,N'_-bis(4-methoxybenzyl)-4-(5-(p-tolyl)-3-(trifluoromethyl)-1H-pyrazol-1-yl)benzenesulfonimidamide (2h)

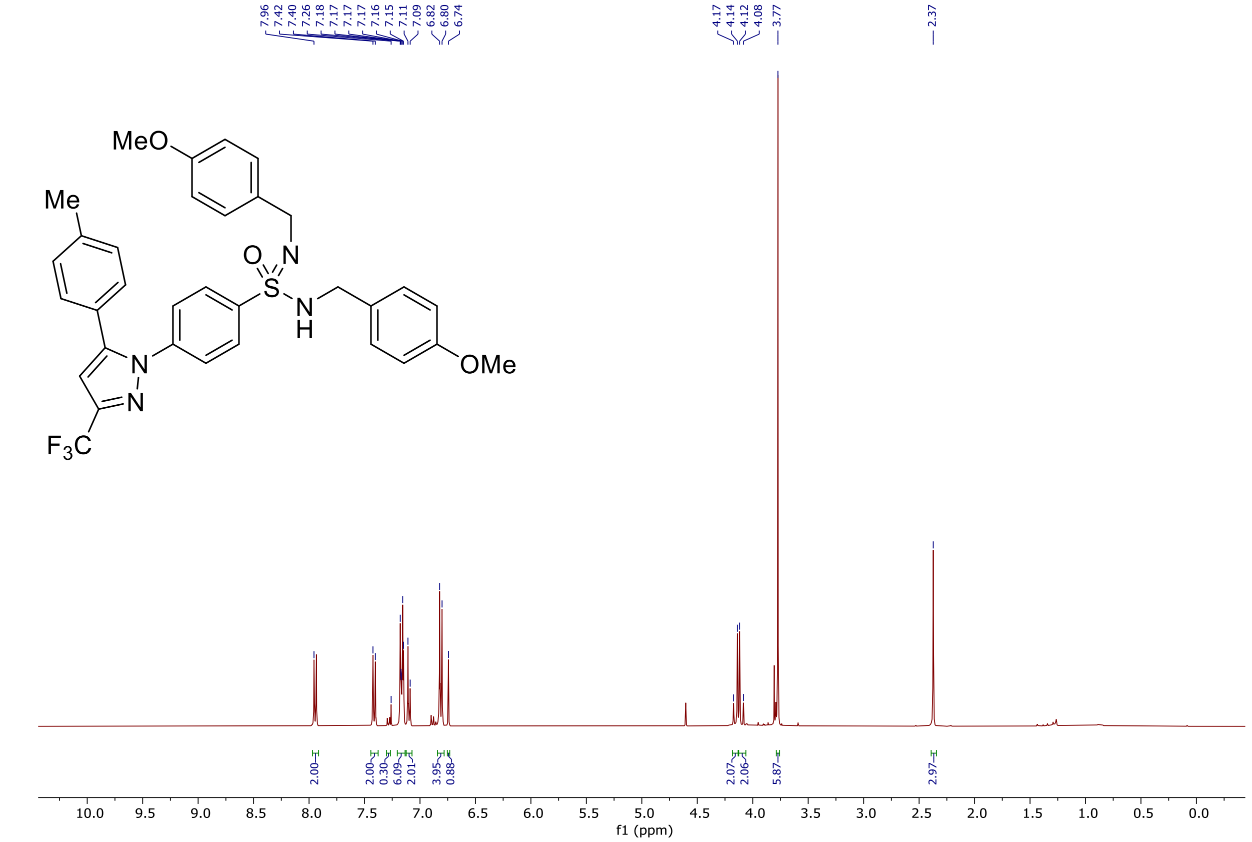

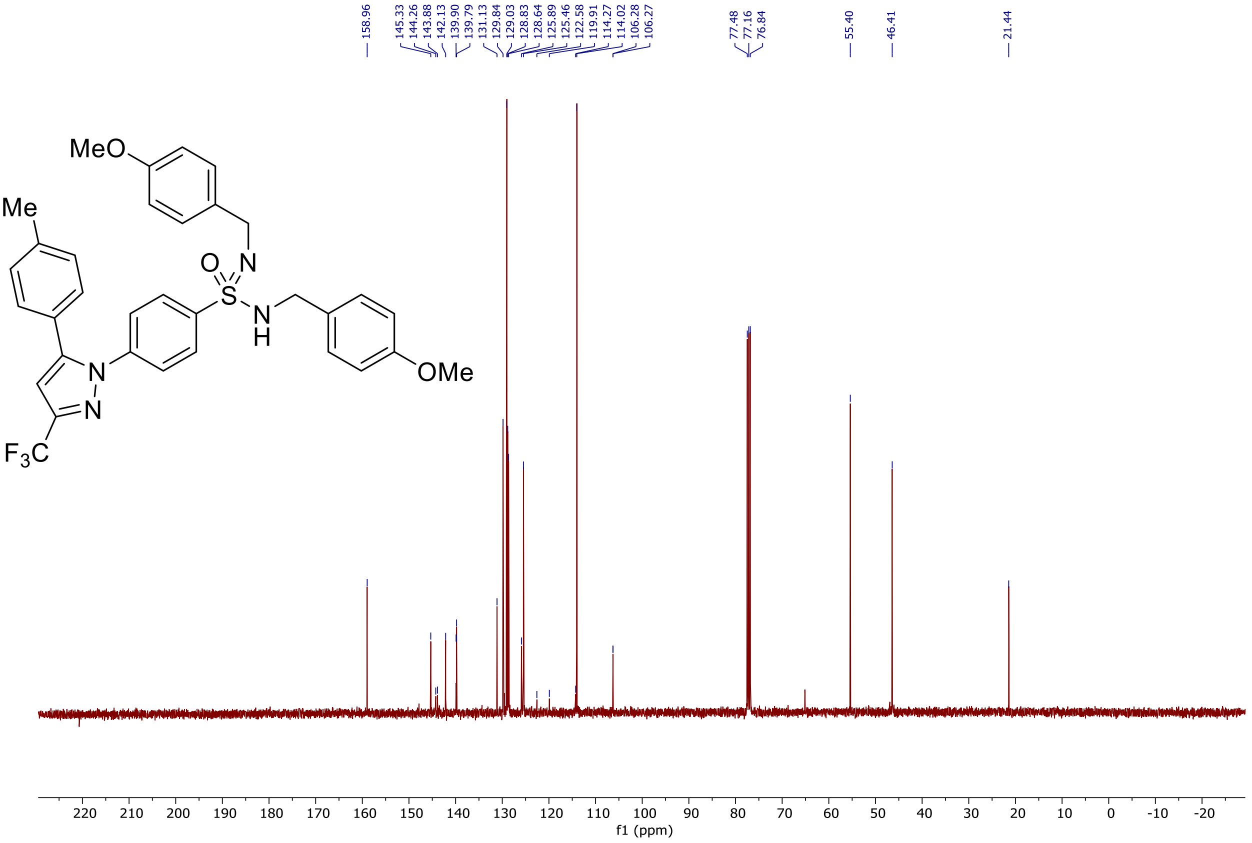

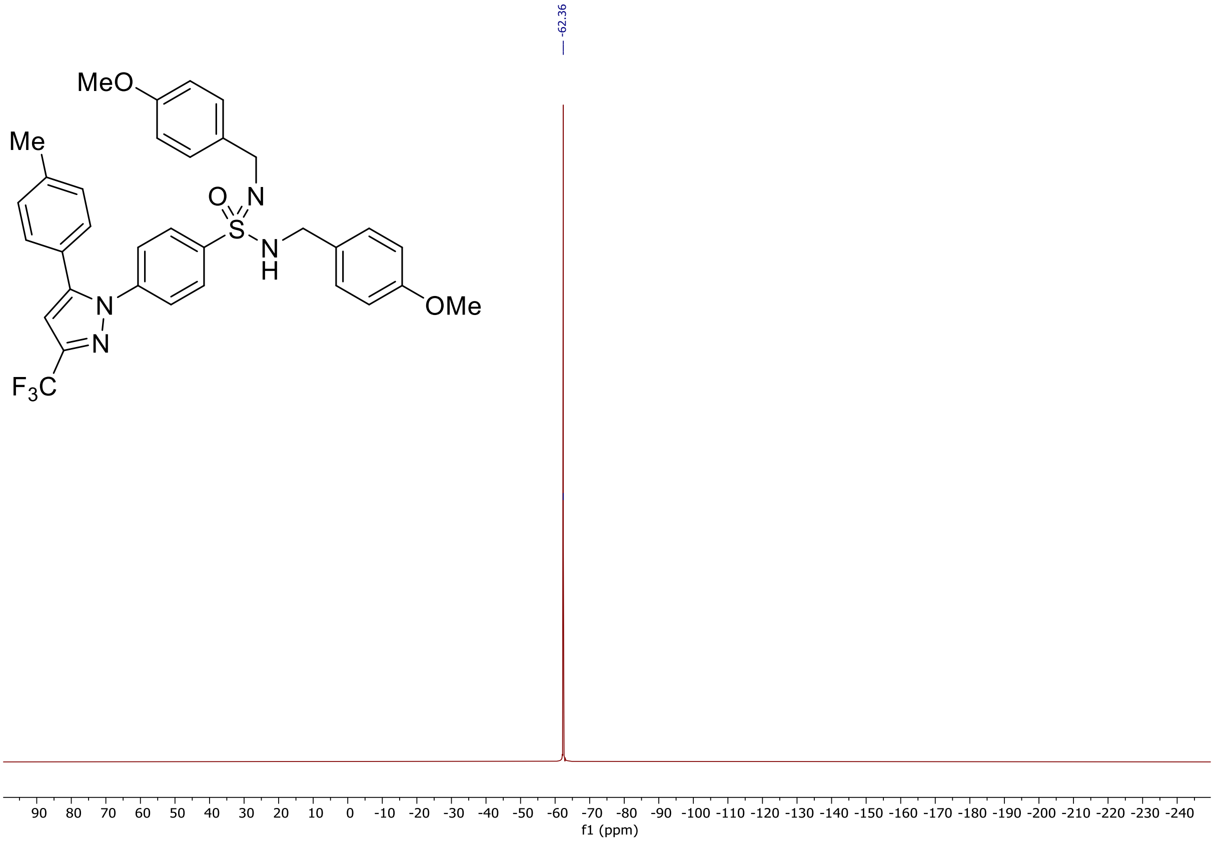
-62.36
MeO
Me
O
N
S
N
H
N
N
OMe
F3C
90 80 70 60 50 40 30 20 10 0 -10 -20 -30 -40 -50 -60 -70 -80 -90 -100 -110 -120 -130 -140 -150 -160 -170 -180 -190 -200 -210 -220 -230 -240
f1 (ppm)

N,*N'*-bis(4-methoxybenzyl)-2-methylprop-1-ene-1-sulfonimidamide (2i)

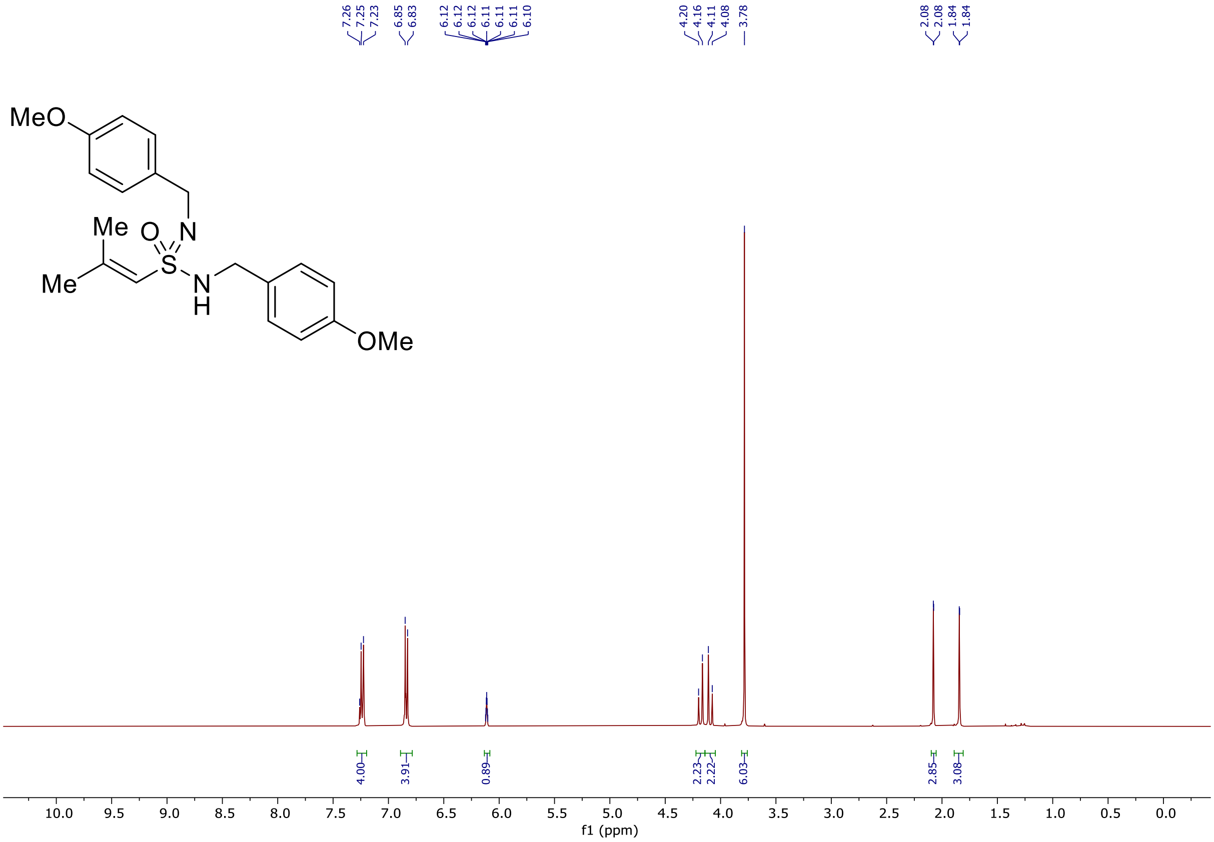

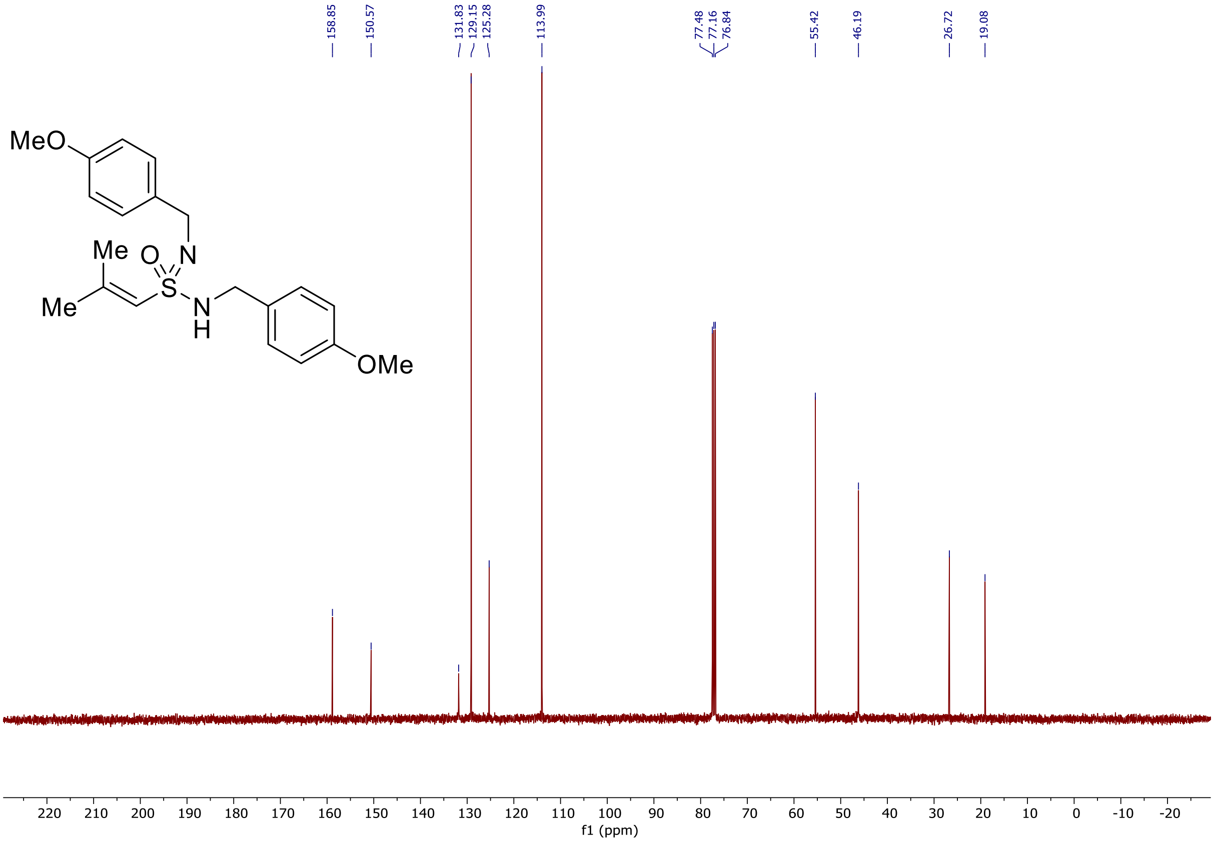

***N*,*N'*-bis(4-methoxybenzyl)methanesulfonimidamide (2j)**

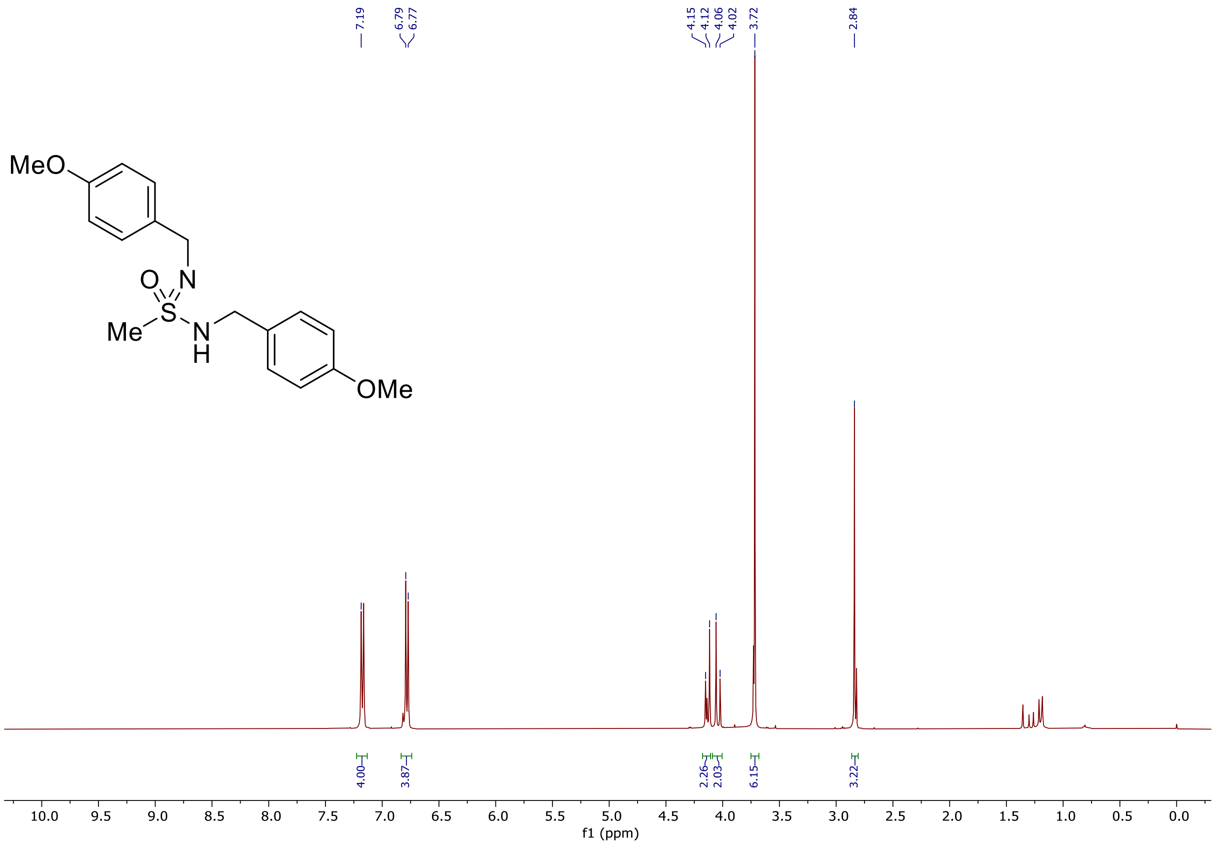

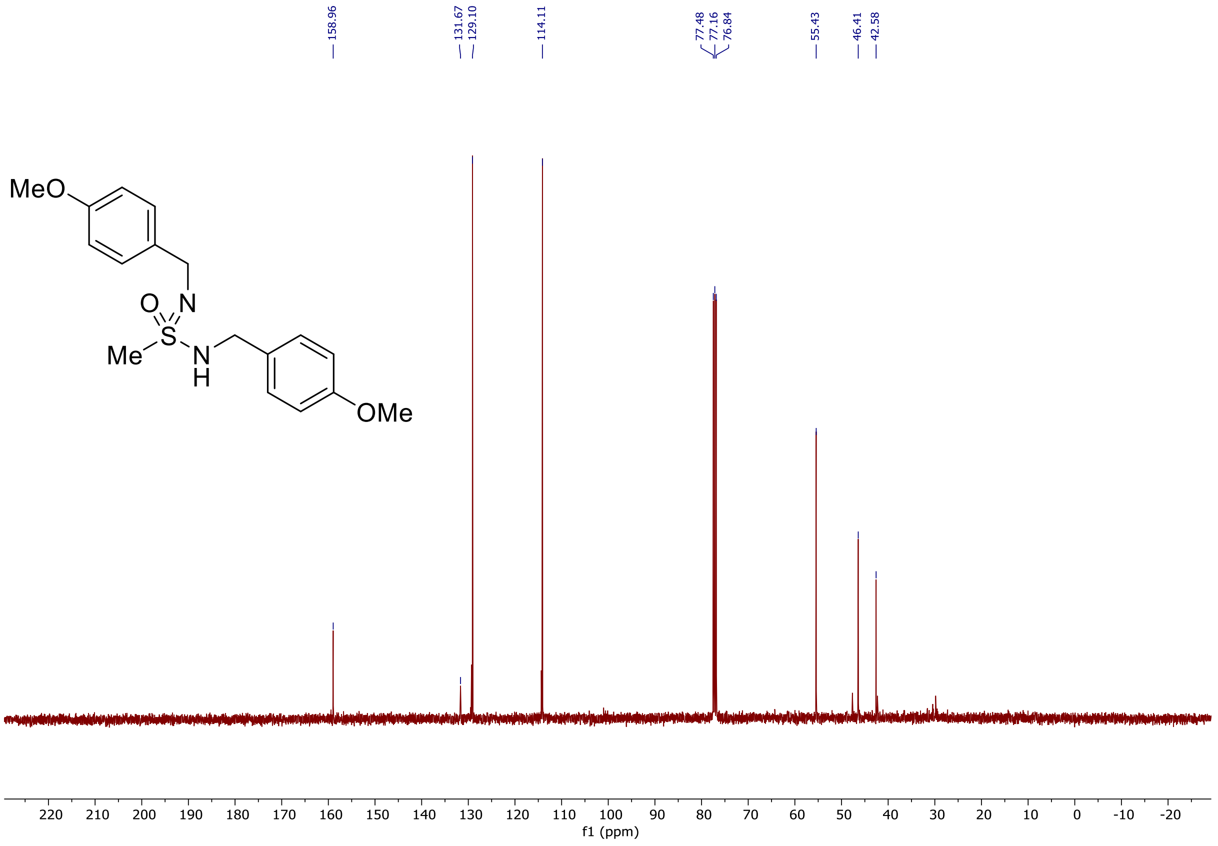

(*S*)-*N*-benzyl-*N*,*N*'-bis(4-methoxybenzyl)-[1,1'-biphenyl]-4-sulfonimidamide (3aa)

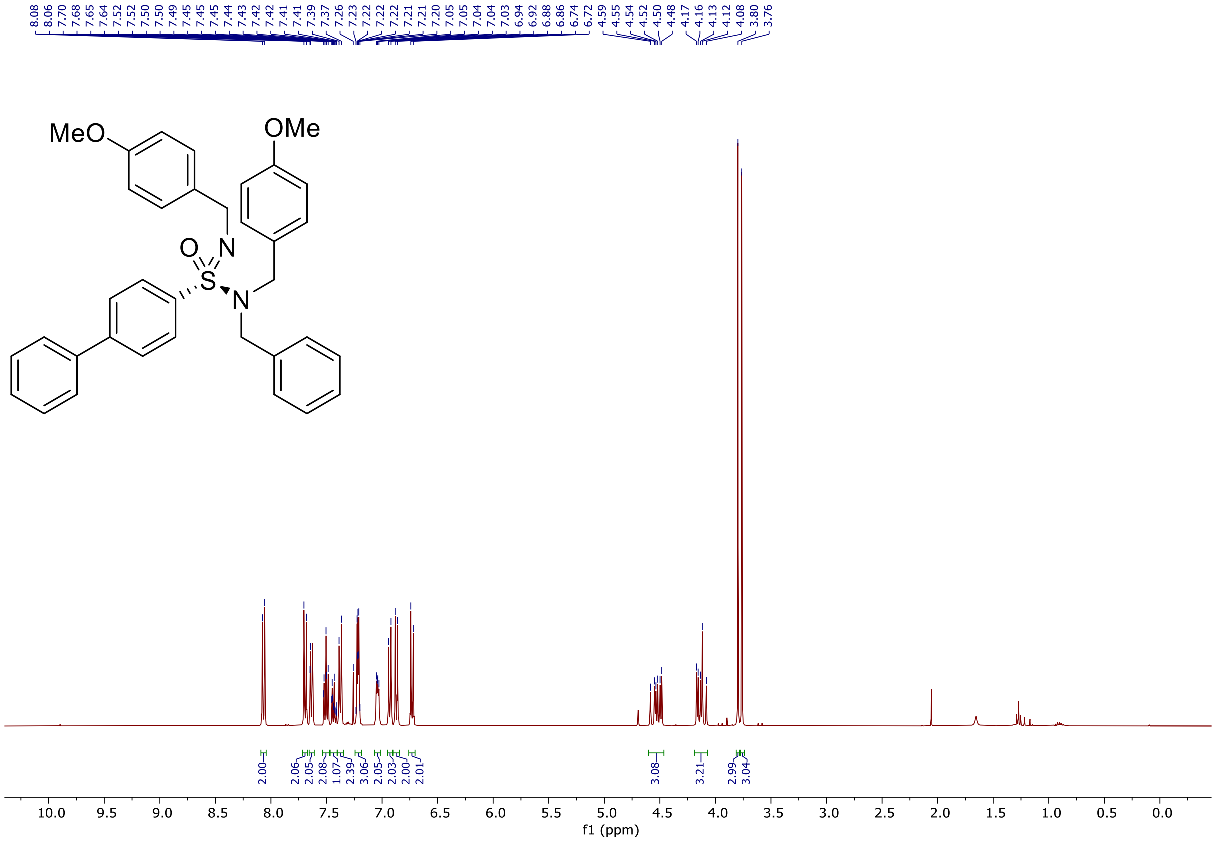

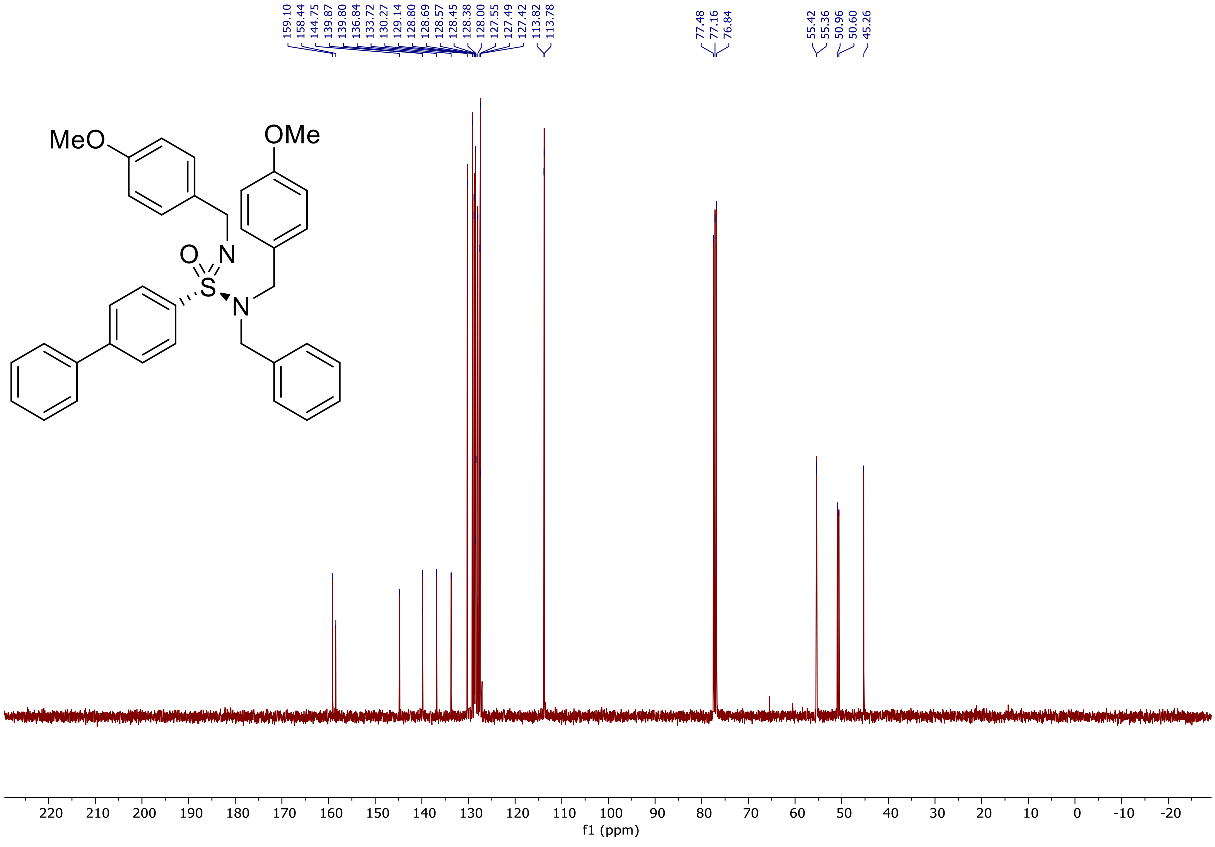

(*S*)-*N*-(4-fluorobenzyl)-*N*,*N'*-bis(4-methoxybenzyl)-[1,1'-biphenyl]-4-sulfonimidamide (3ab)

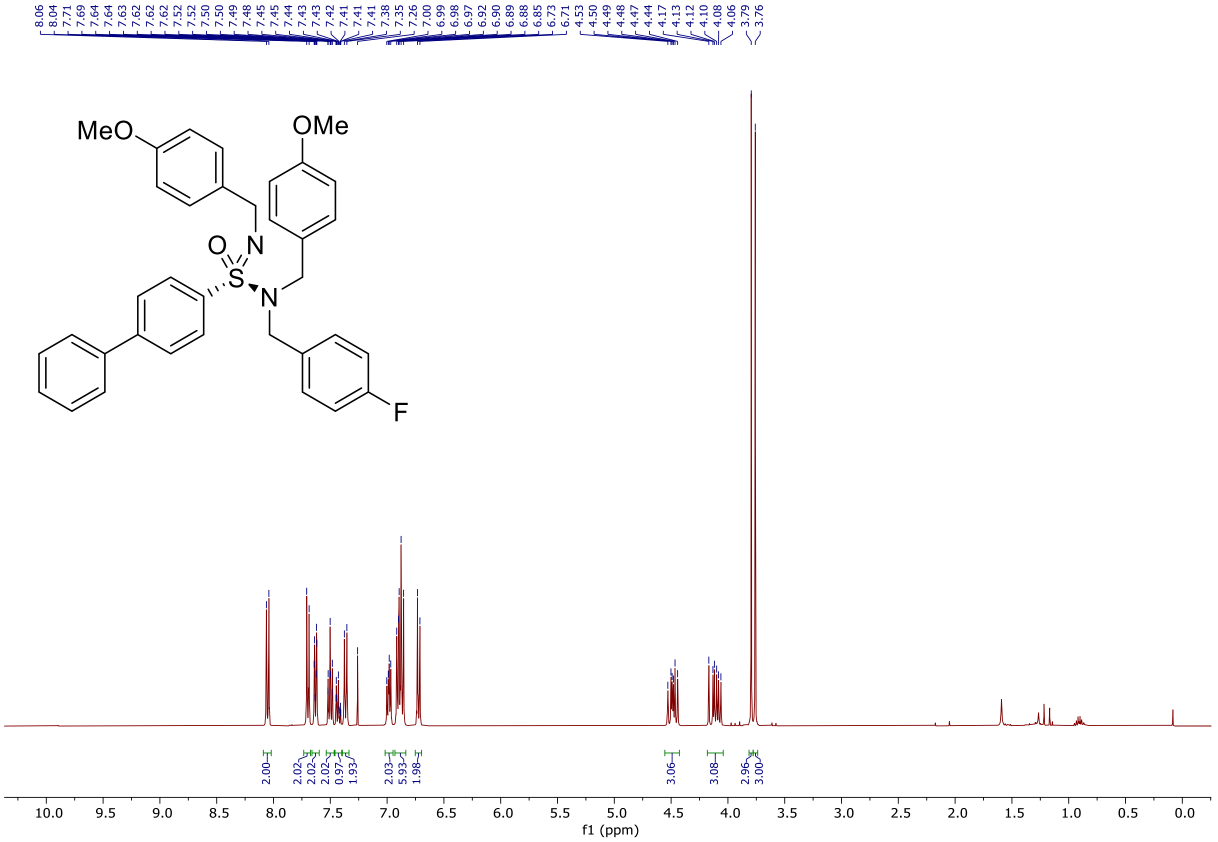

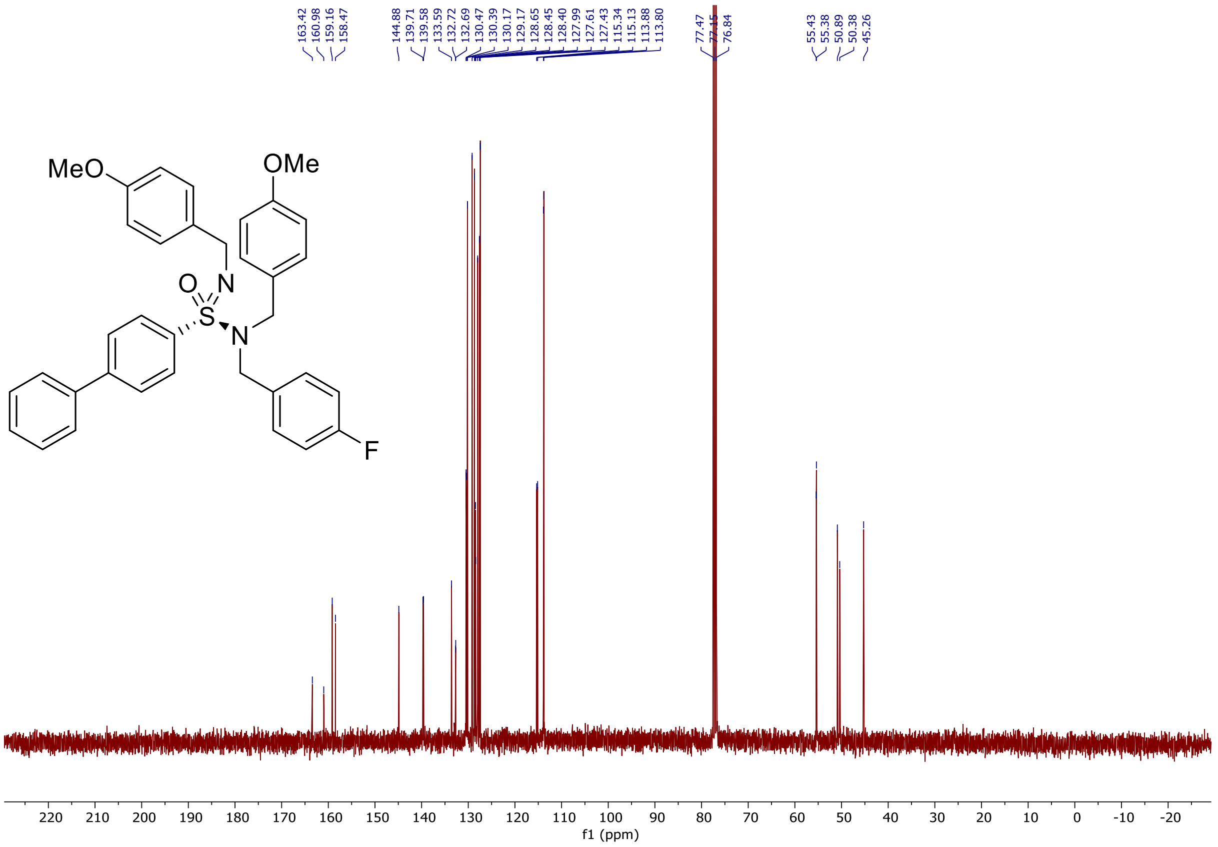

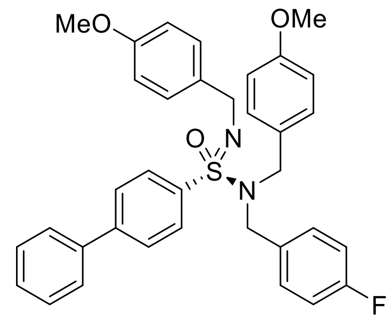

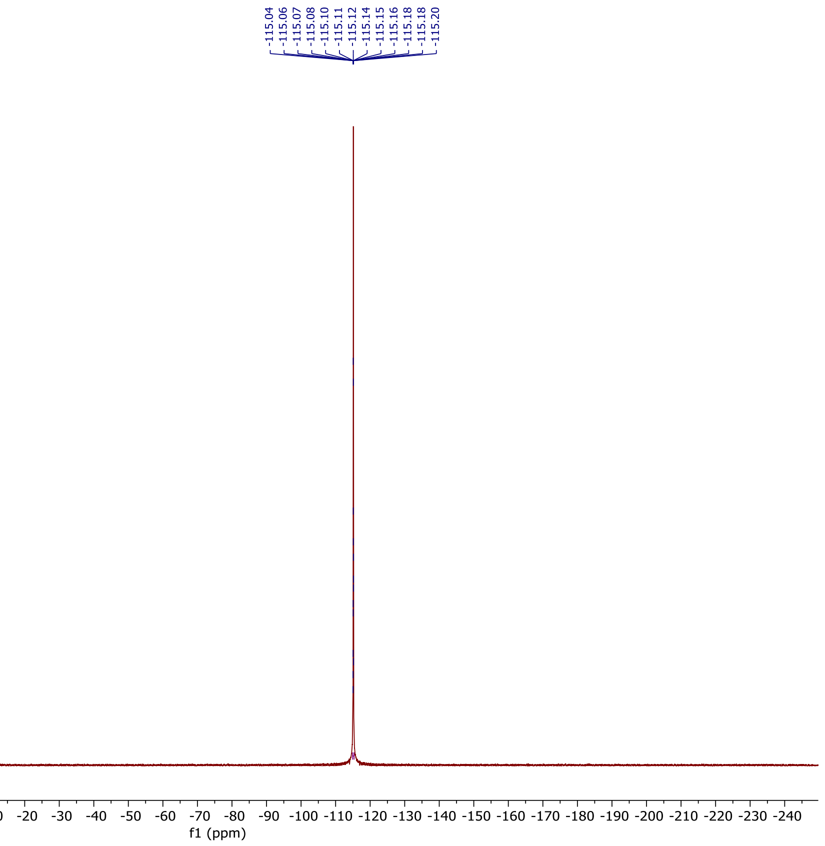
-115.04
-115.06
-115.07
-115.08
-115.10
-115.11
-115.12
-115.14
-115.15
-115.16
-115.18
-115.18
-115.20
90 80 70 60 50 40 30 20 10 0 -10 -20 -30 -40 -50 -60 -70 -80 -90 -100 -110 -120 -130 -140 -150 -160 -170 -180 -190 -200 -210 -220 -230 -240
f1 (ppm)

(*S*)-*N*,*N'*-bis(4-methoxybenzyl)-*N*-(4-(methylthio)benzyl)-[1,1'-biphenyl]-4-sulfonimidamide (3ac)

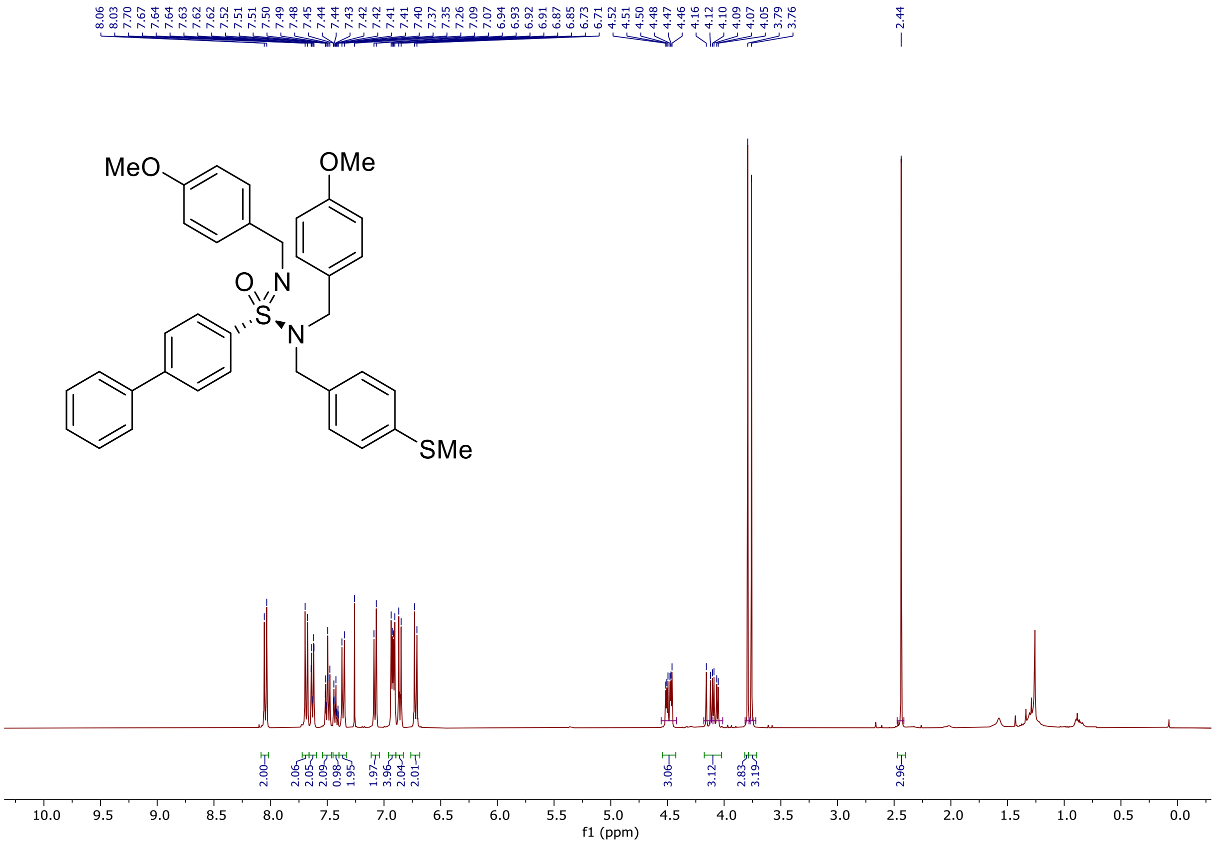

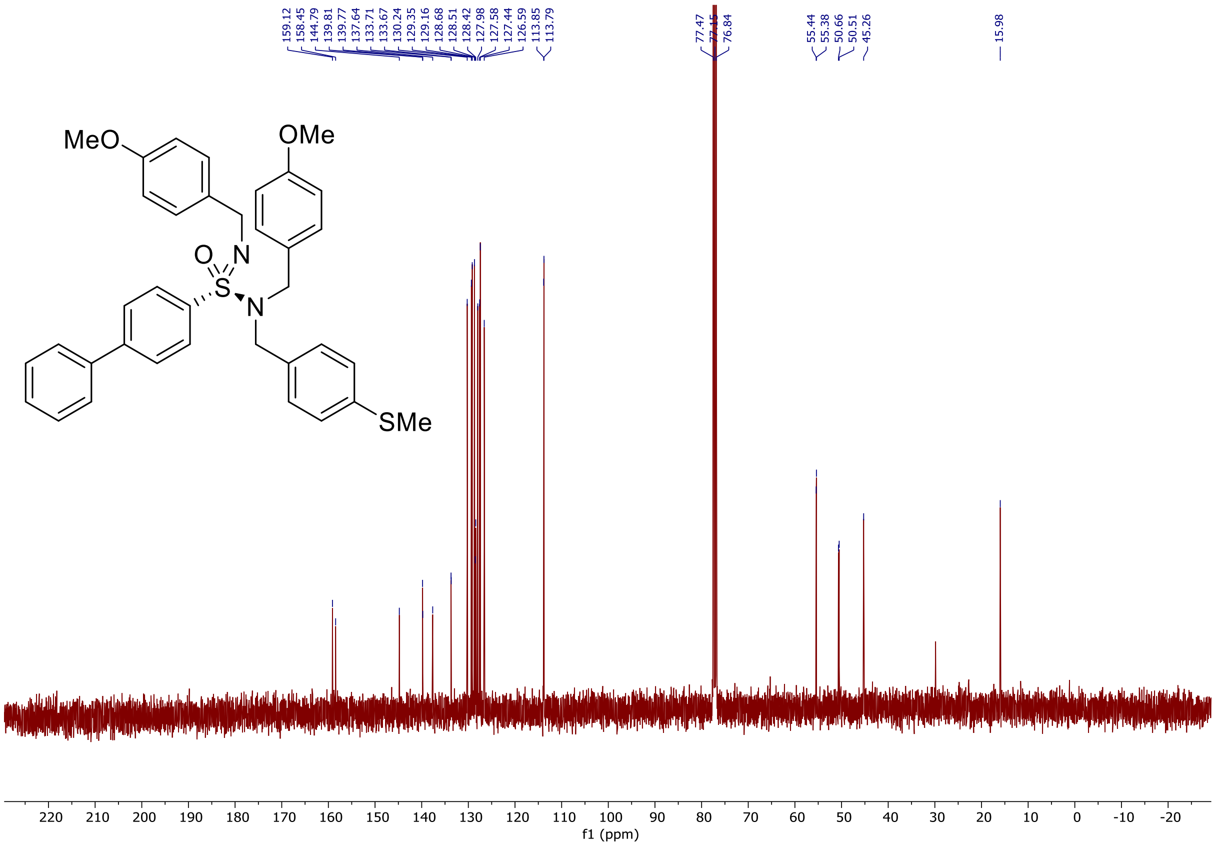

(*S*)-*N*-(4-cyanobenzyl)-*N*,*N'*-bis(4-methoxybenzyl)-[1,1'-biphenyl]-4-sulfonimidamide (3ad)

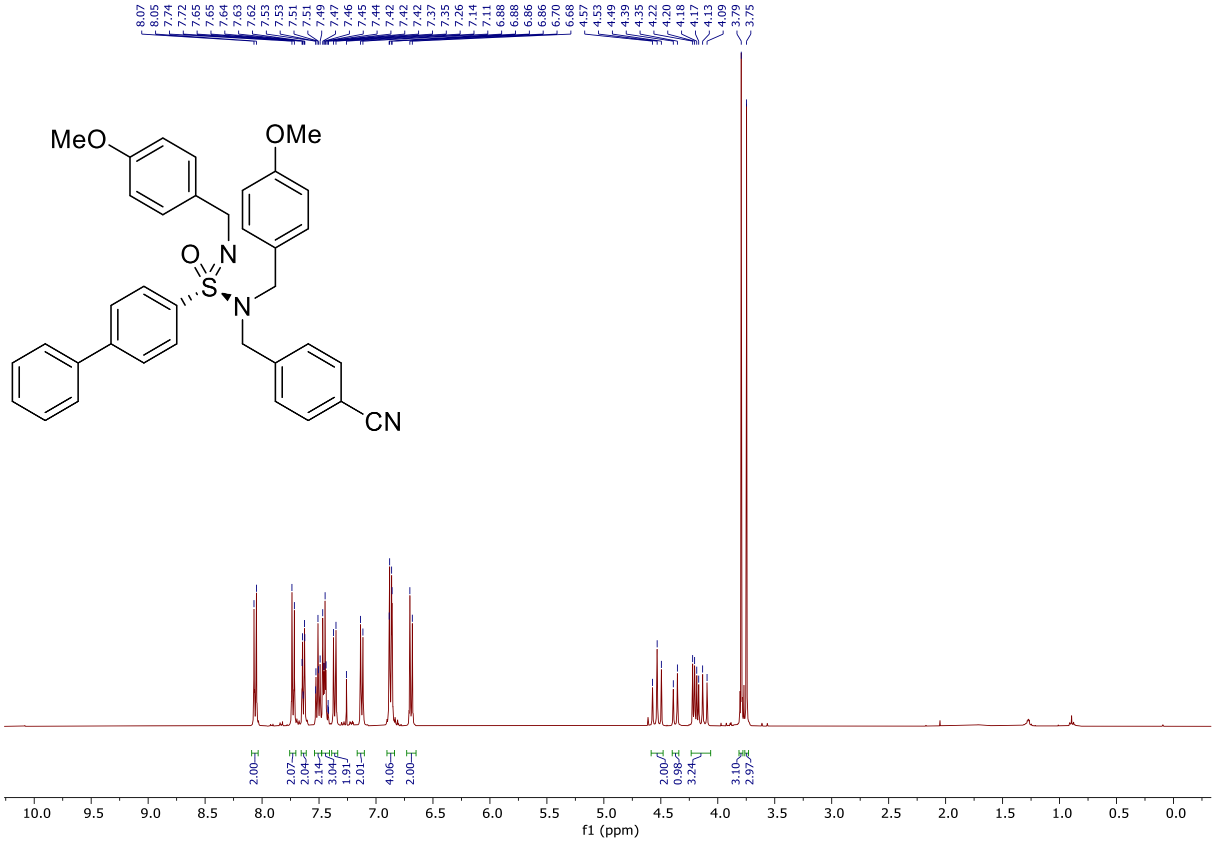

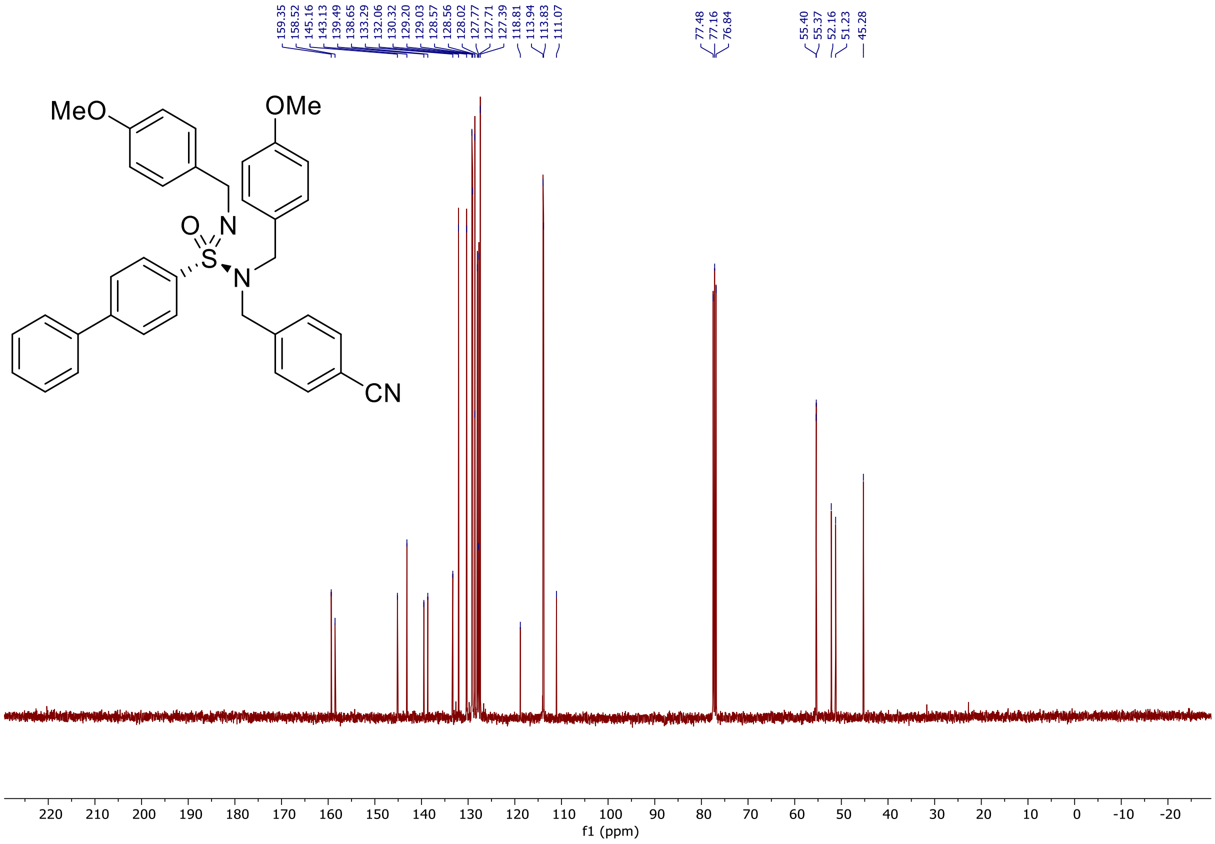

(*S*)-*N*-(3-formylbenzyl)-*N*,*N'*-bis(4-methoxybenzyl)-[1,1'-biphenyl]-4-sulfonimidamide (3ae)

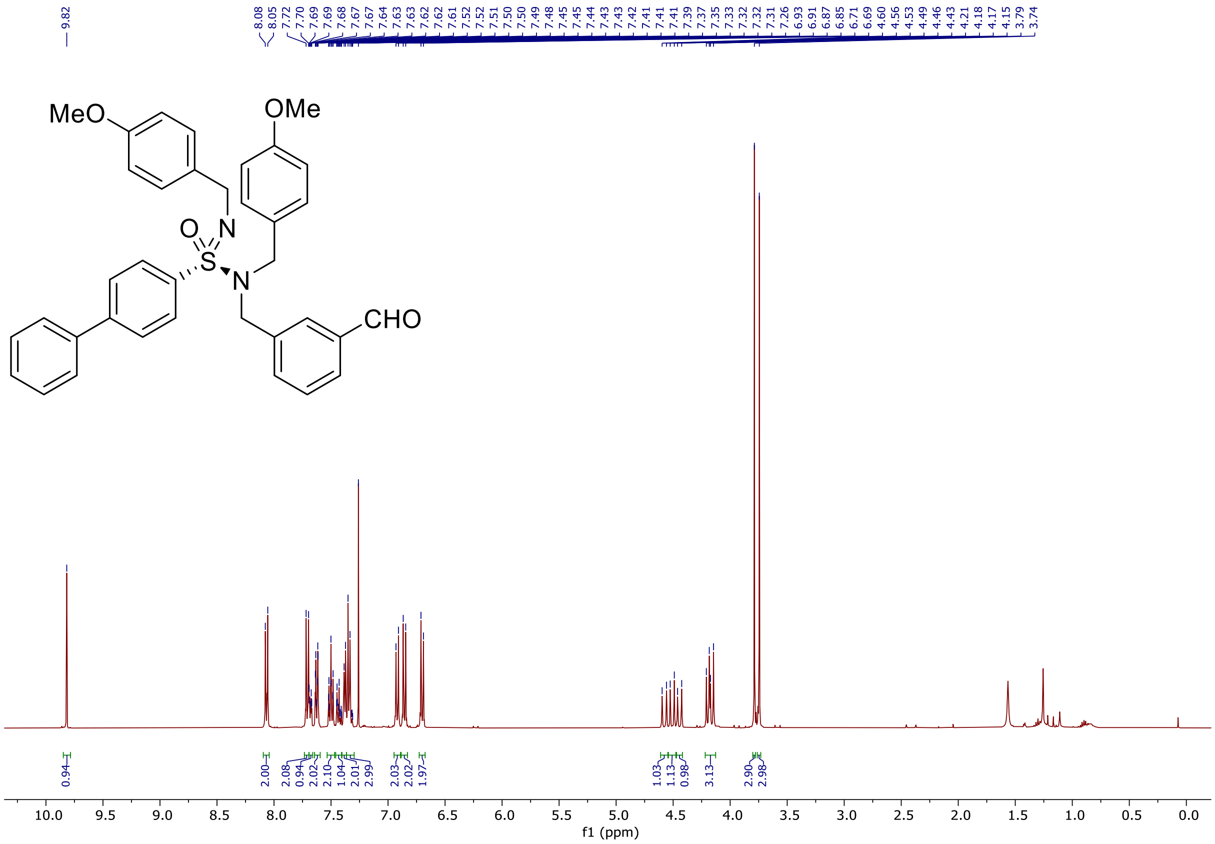

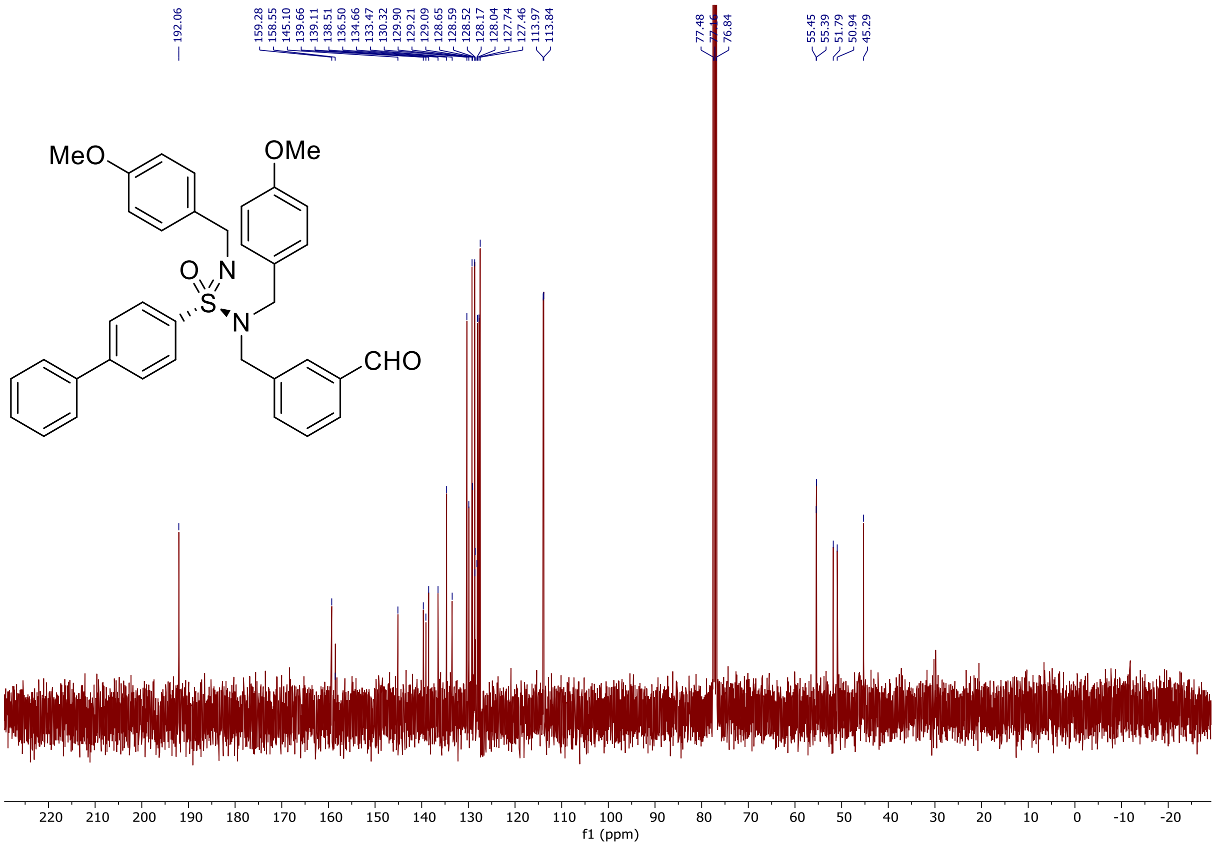

(*S*)-*N*,*N'*-bis(4-methoxybenzyl)-*N*-(naphthalen-2-ylmethyl)-[1,1'-biphenyl]-4-sulfonimidamide (3af)

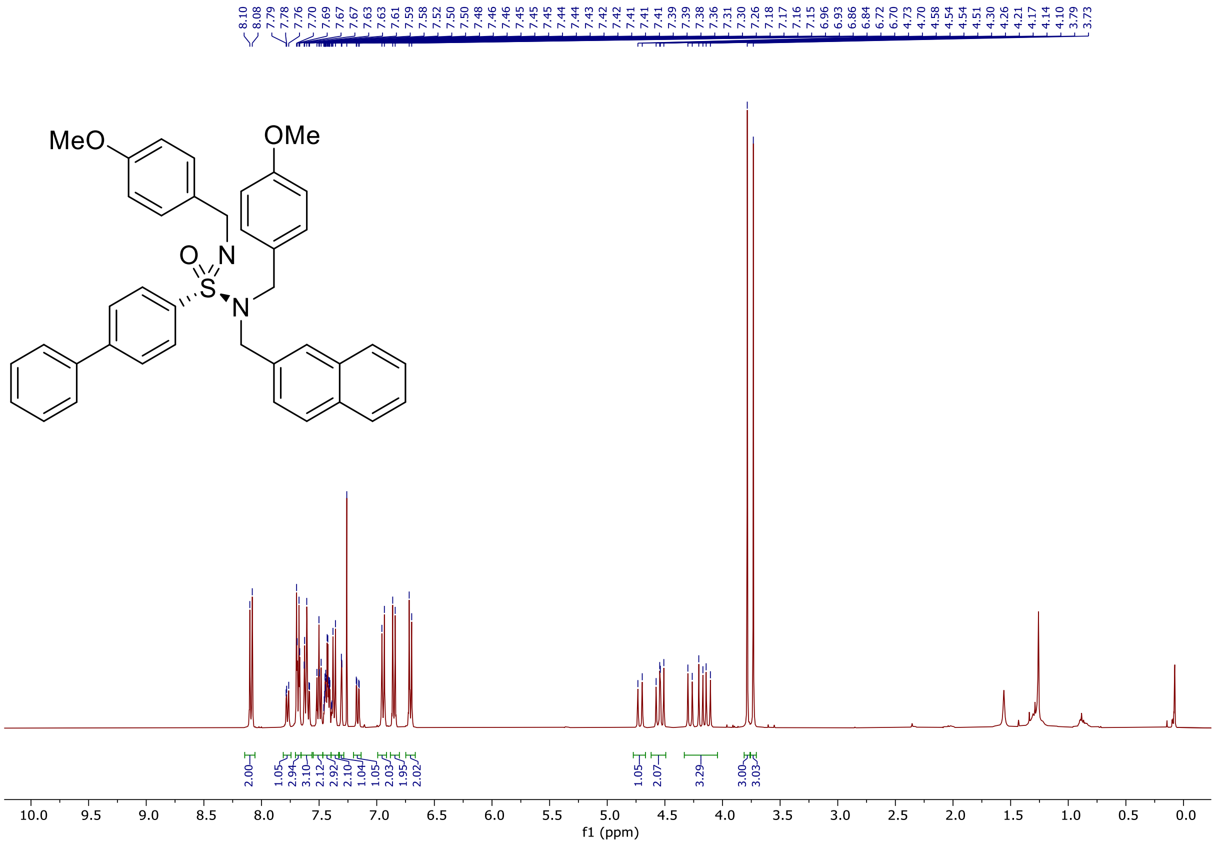

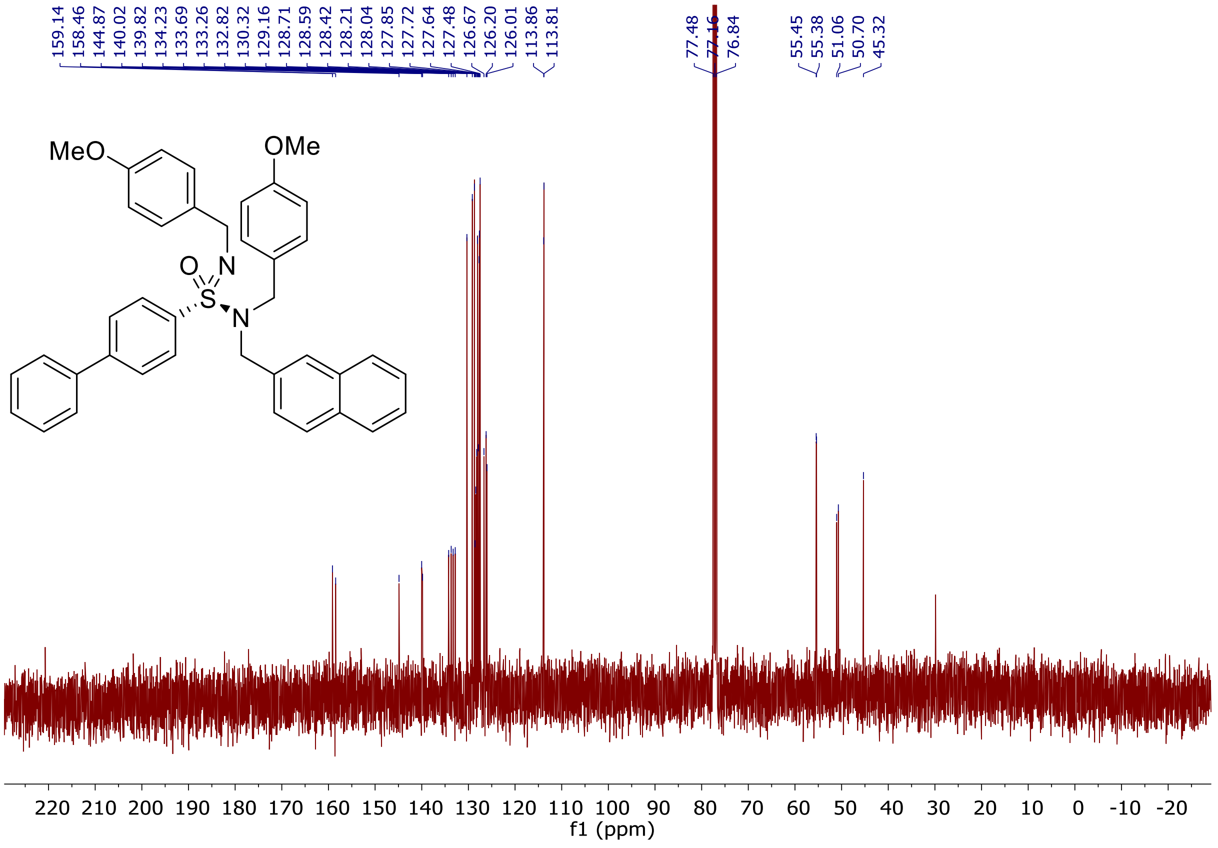

(*S*)-*N*-allyl-*N*,*N'*-bis(4-methoxybenzyl)-[1,1'-biphenyl]-4-sulfonimidamidede (3ag)

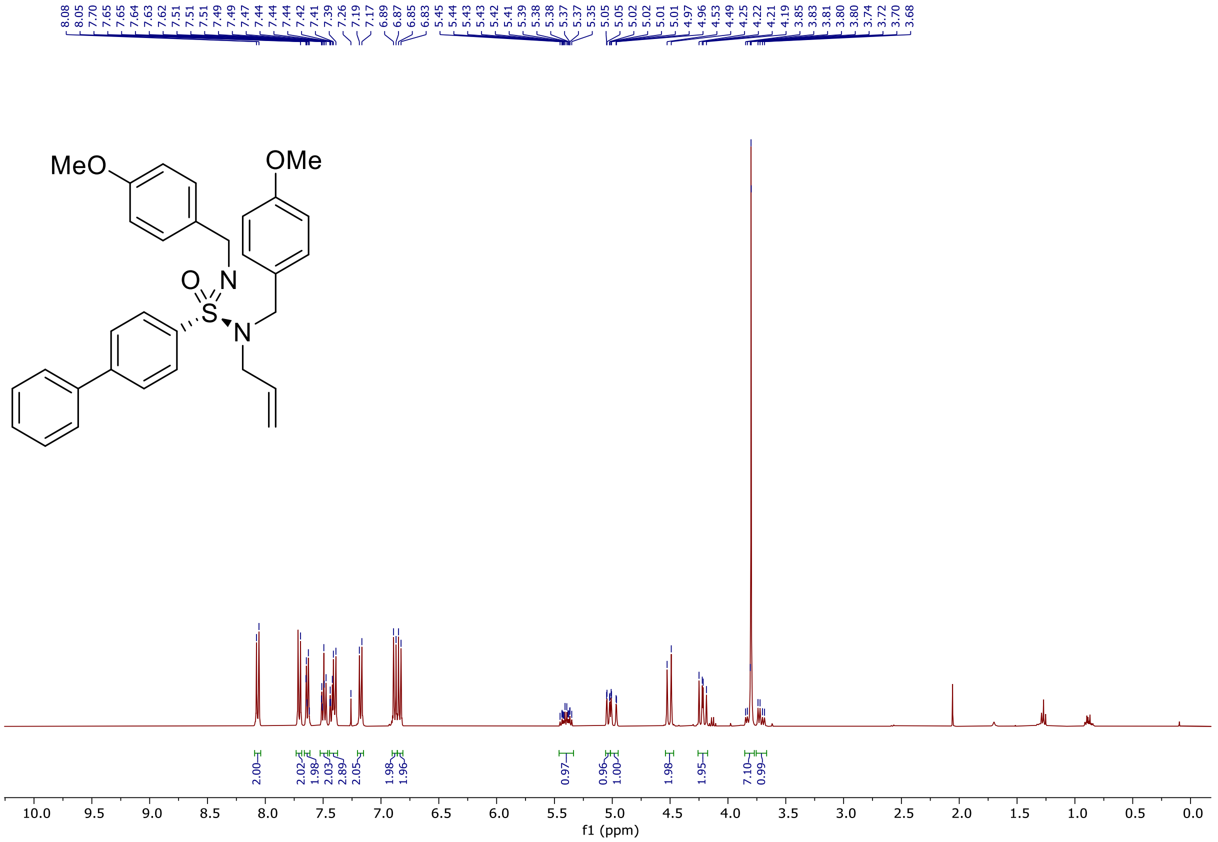

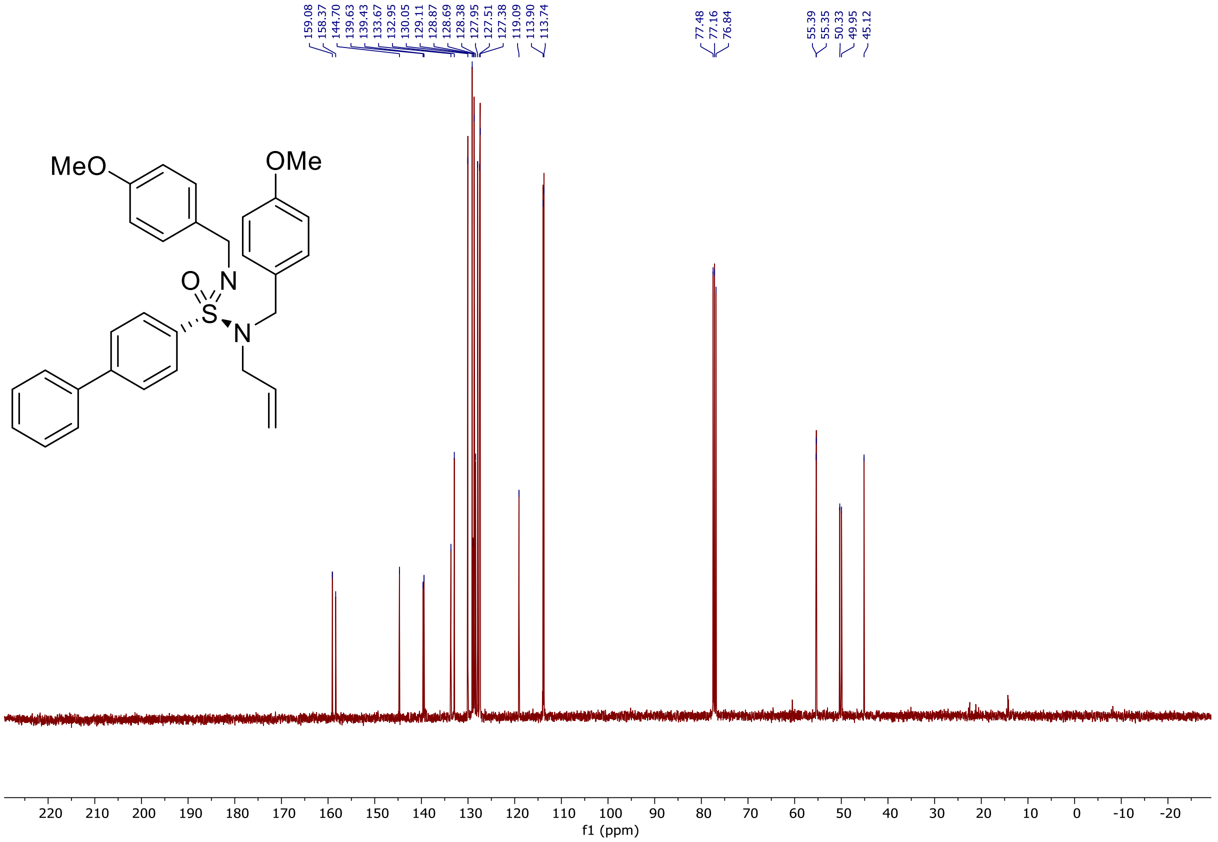

(*S*)-*N*,*N'*-bis(4-methoxybenzyl)-*N*-(2-methylallyl)-[1,1'-biphenyl]-4-sulfonimidamide (3ah)

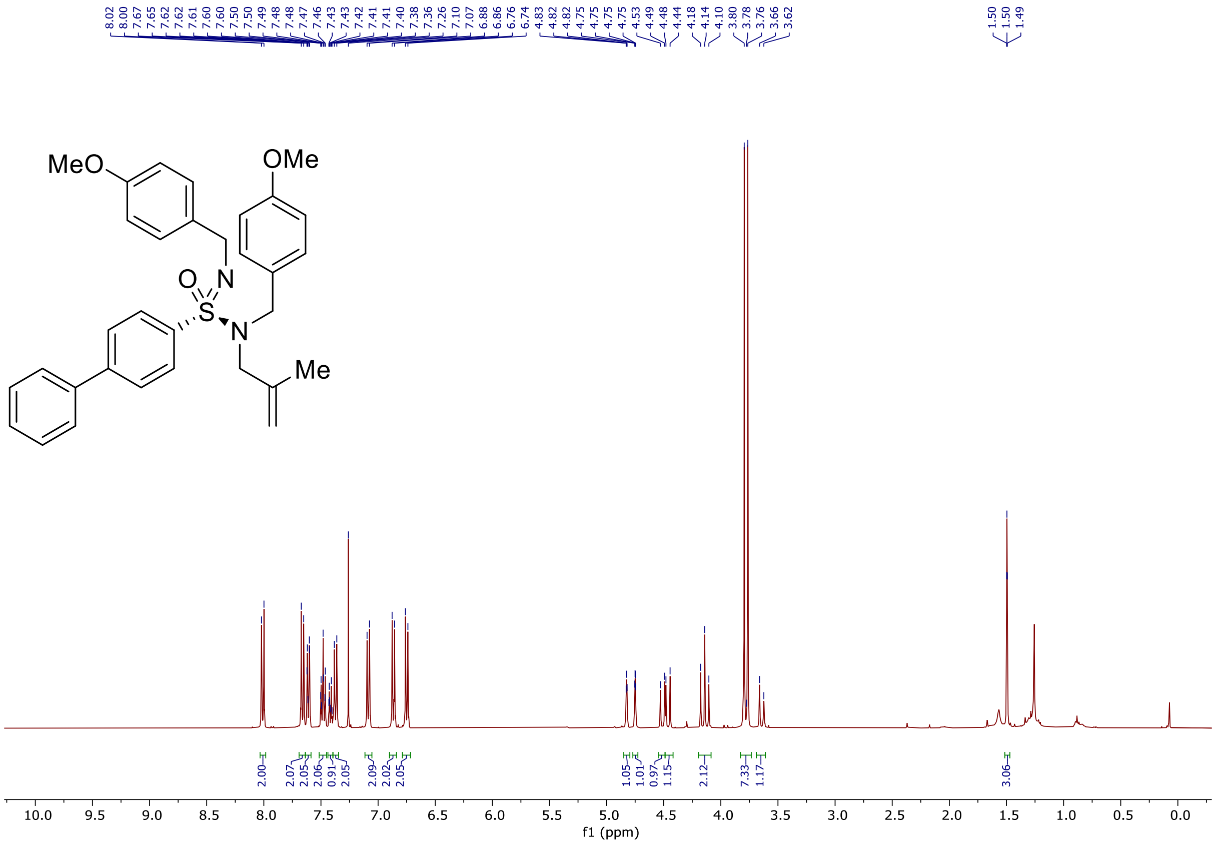

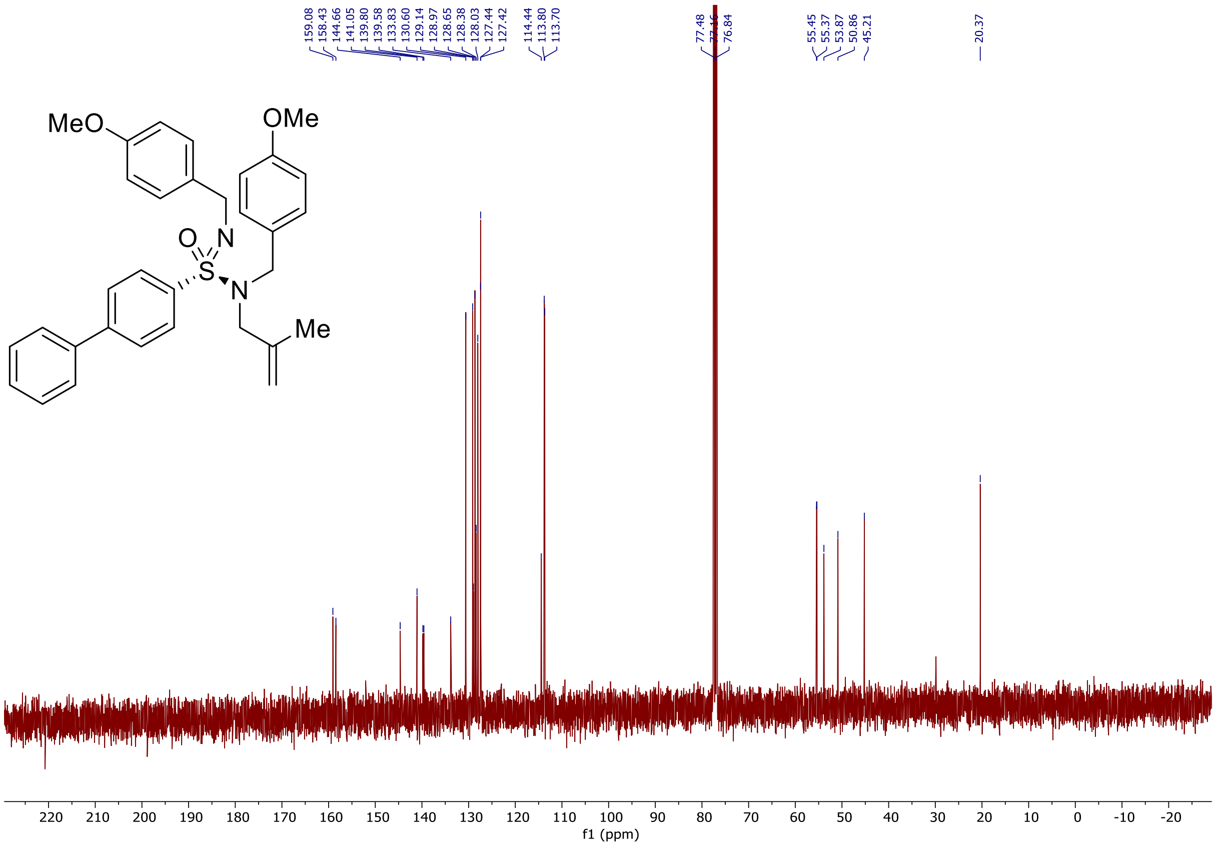

(*S*)-*N*-(cyclopropylmethyl)-*N*,*N'*-bis(4-methoxybenzyl)-[1,1'-biphenyl]-4-sulfonimidamide (3ai)

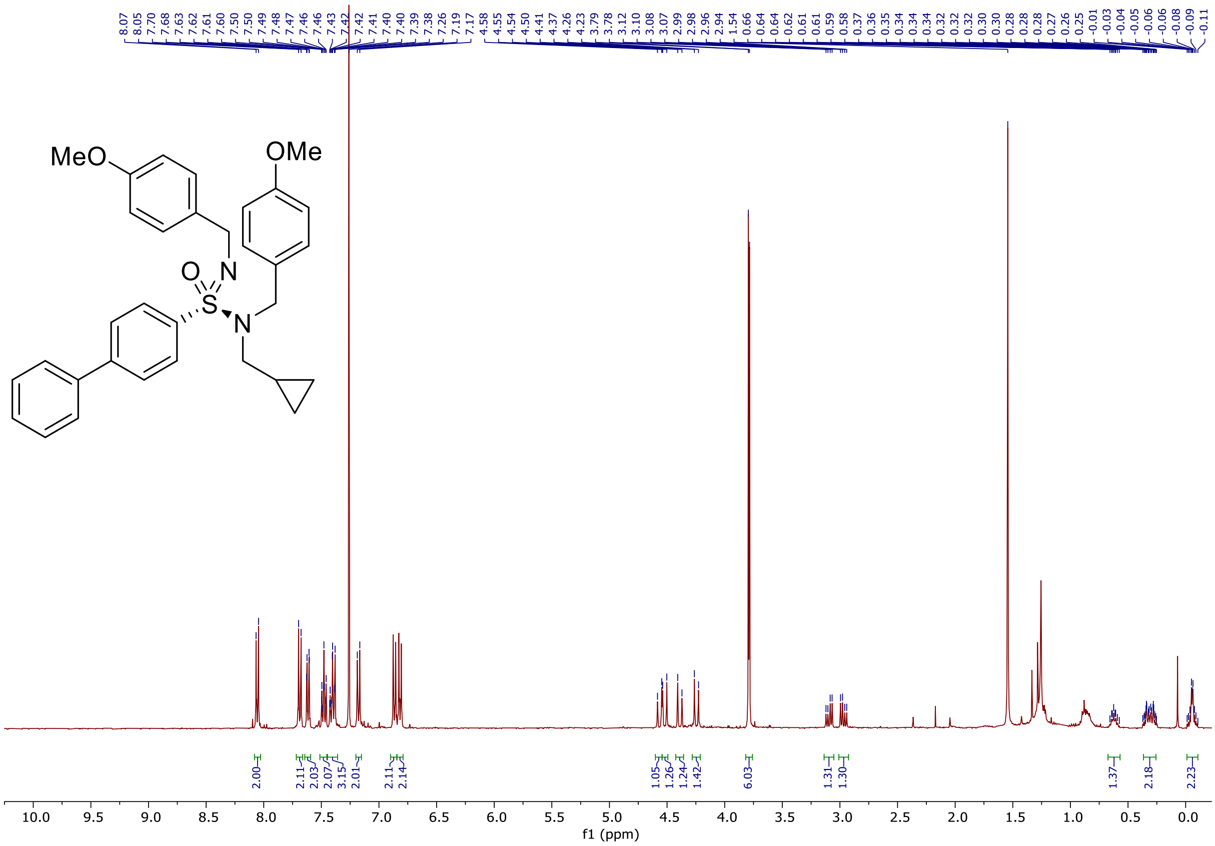

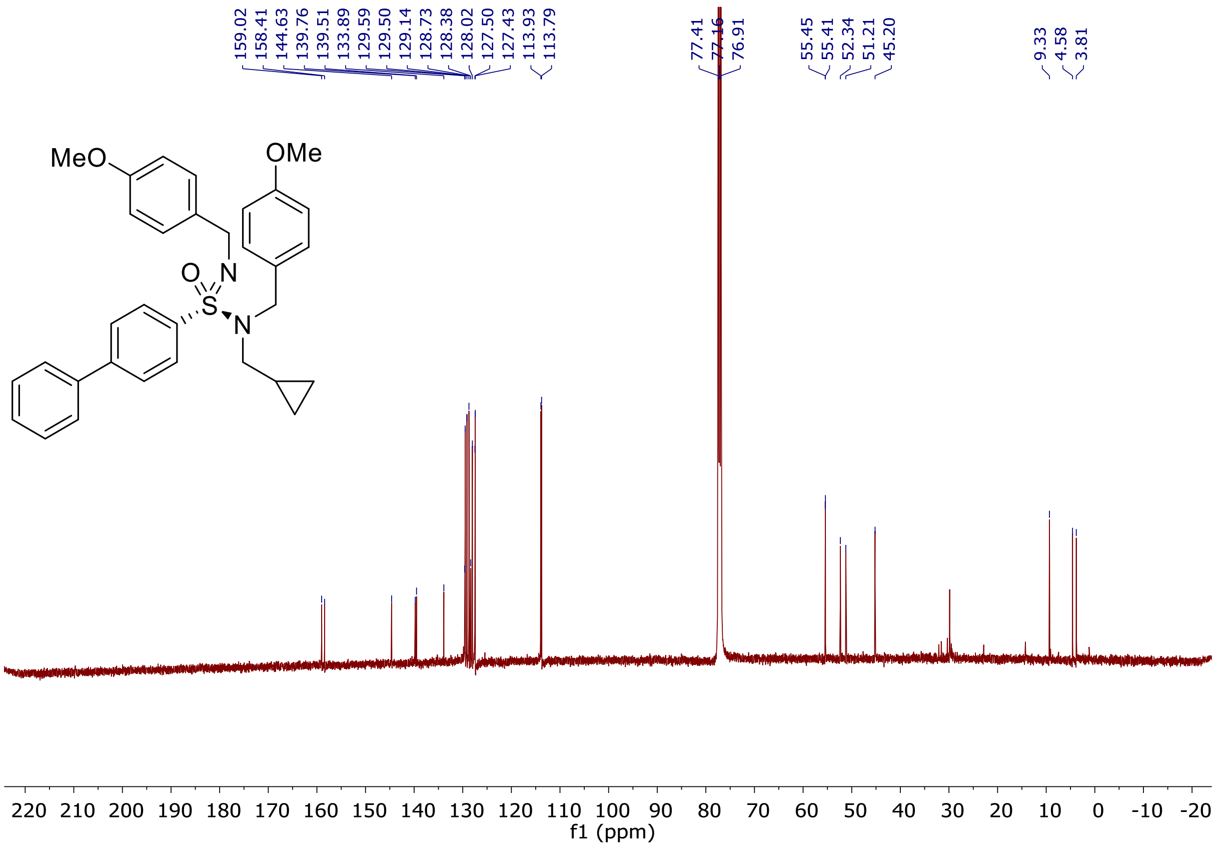

(*S*)-*N*-benzyl-*N*,*N'*-bis(4-methoxybenzyl)-4-methylbenzenesulfonimidamide (3b)

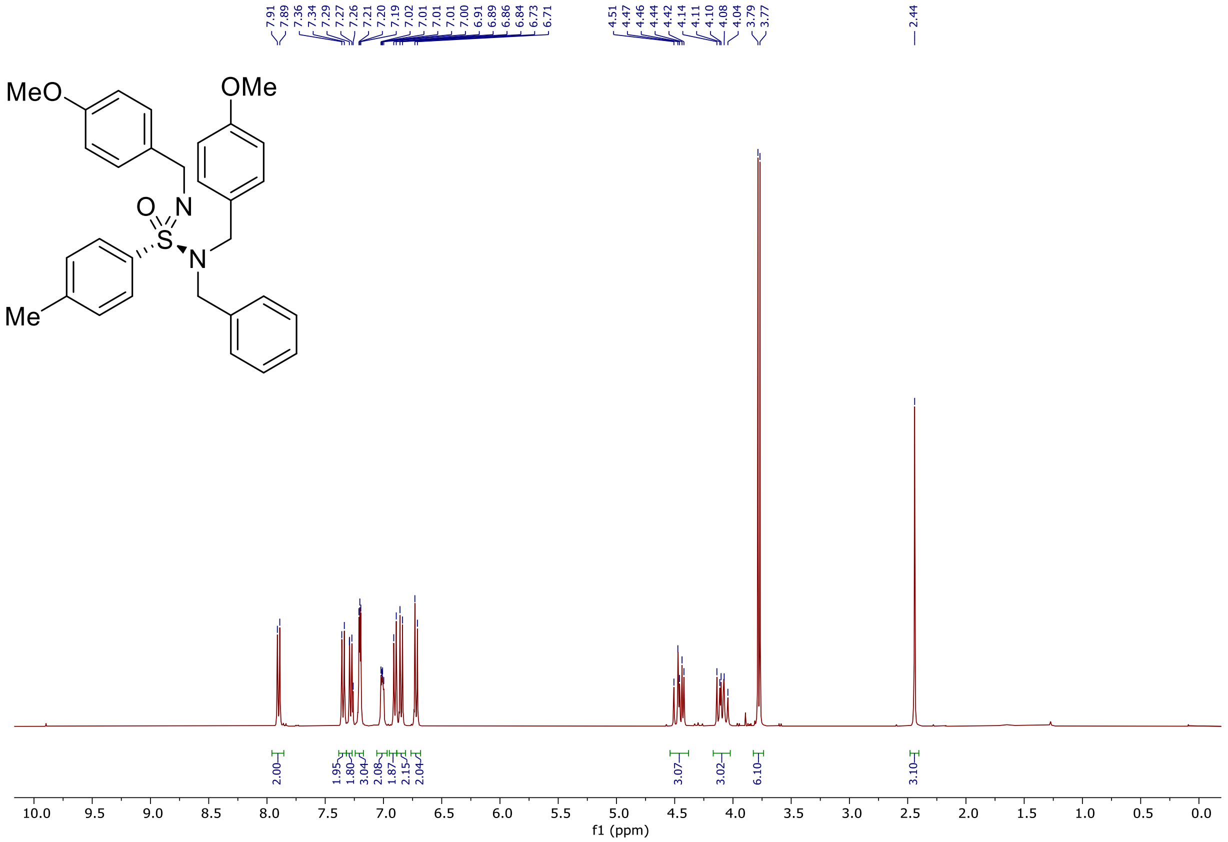

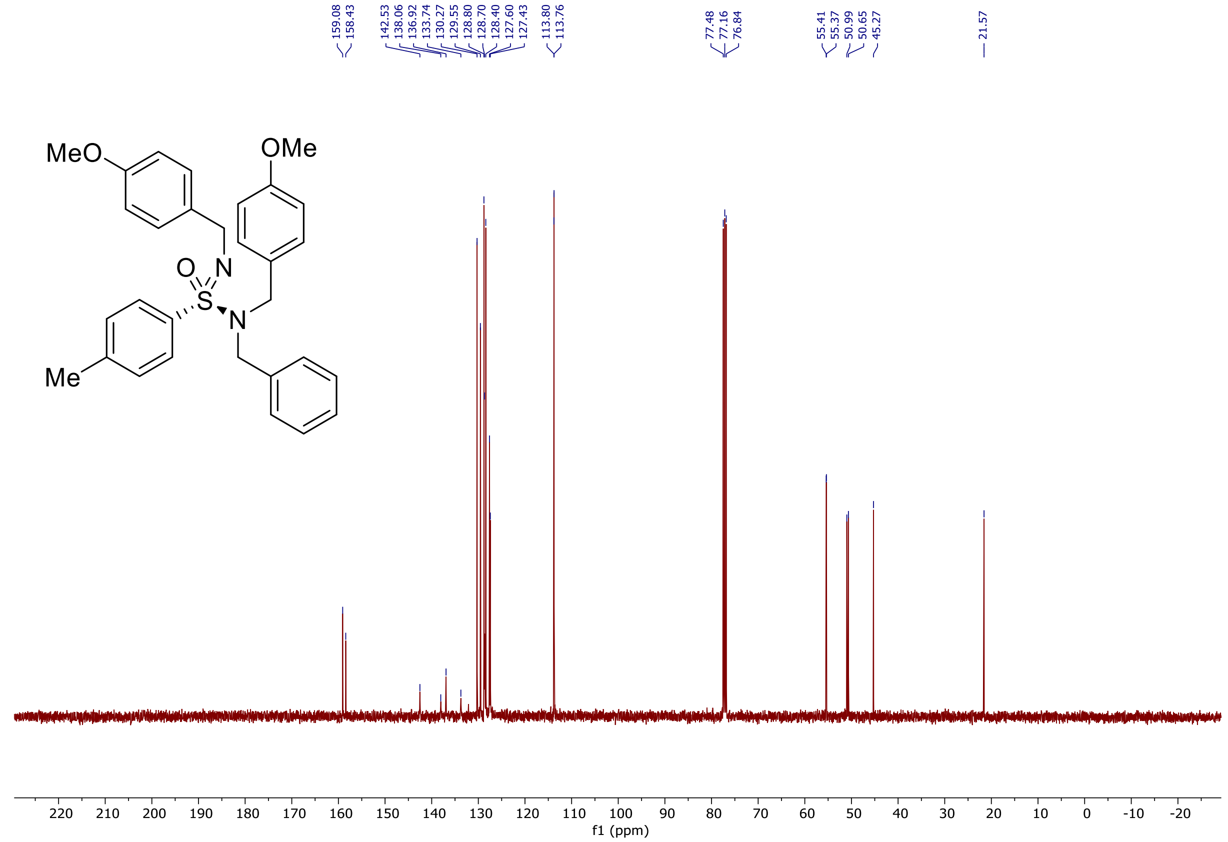

(*S*)-*N*-benzyl-*N*,*N'*-bis(4-methoxybenzyl)benzenesulfonimidamide (3c)

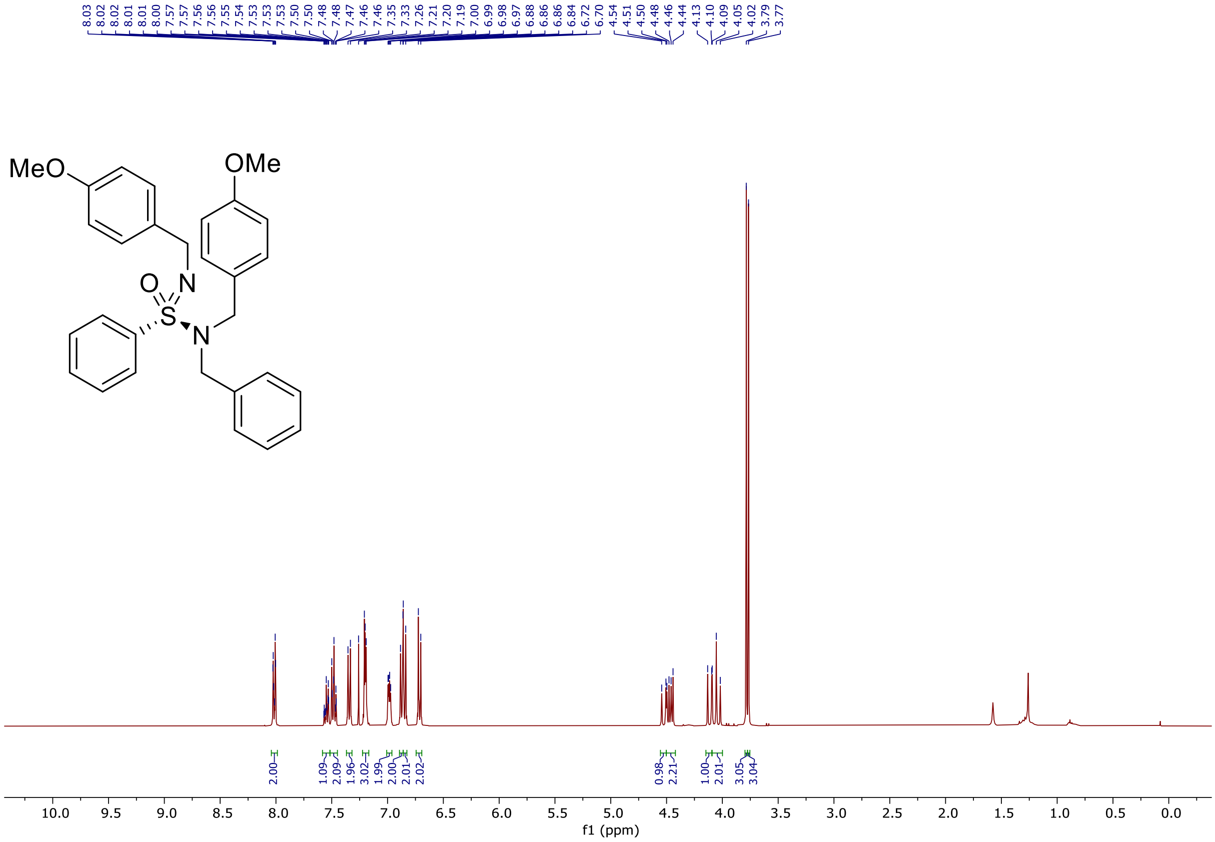

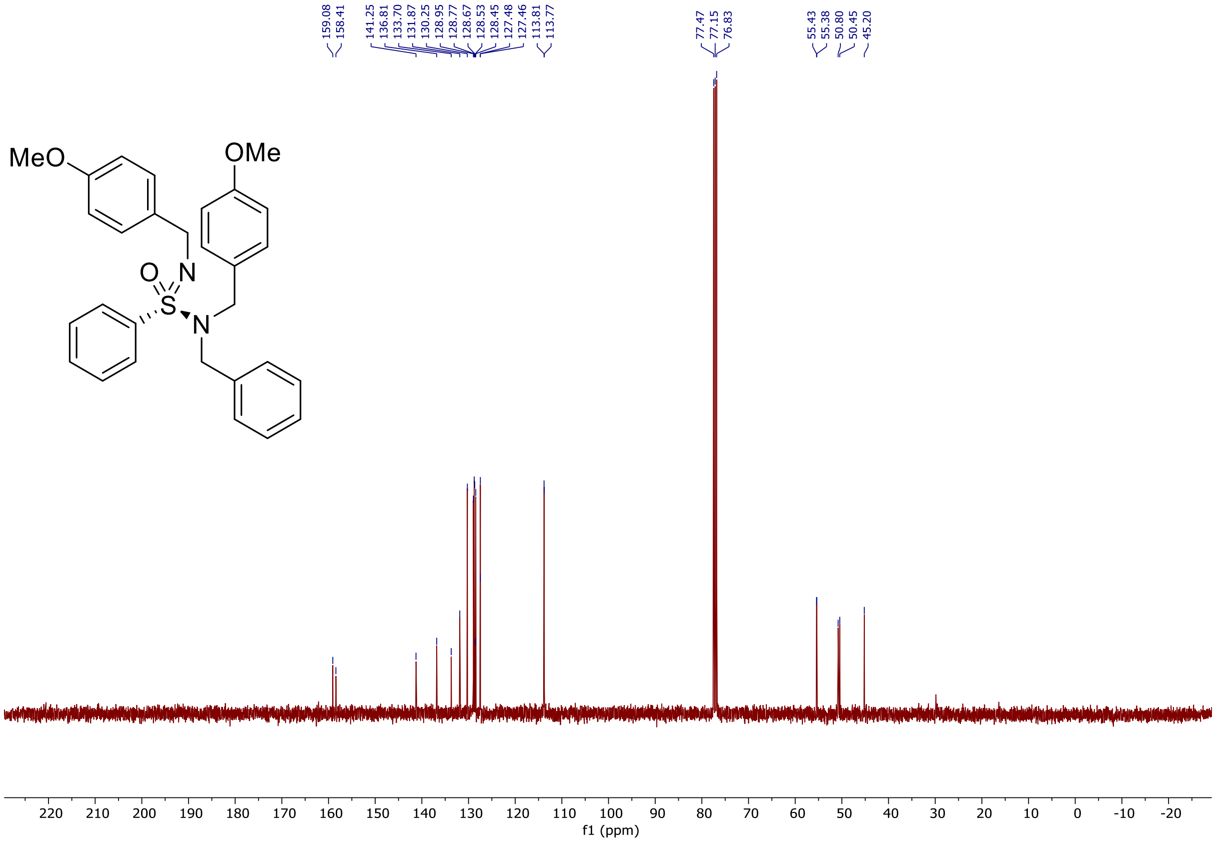

(*S*)-*N*-benzyl-*N*,*N'*-bis(4-methoxybenzyl)-3-methylbenzenesulfonimidamide (3d)

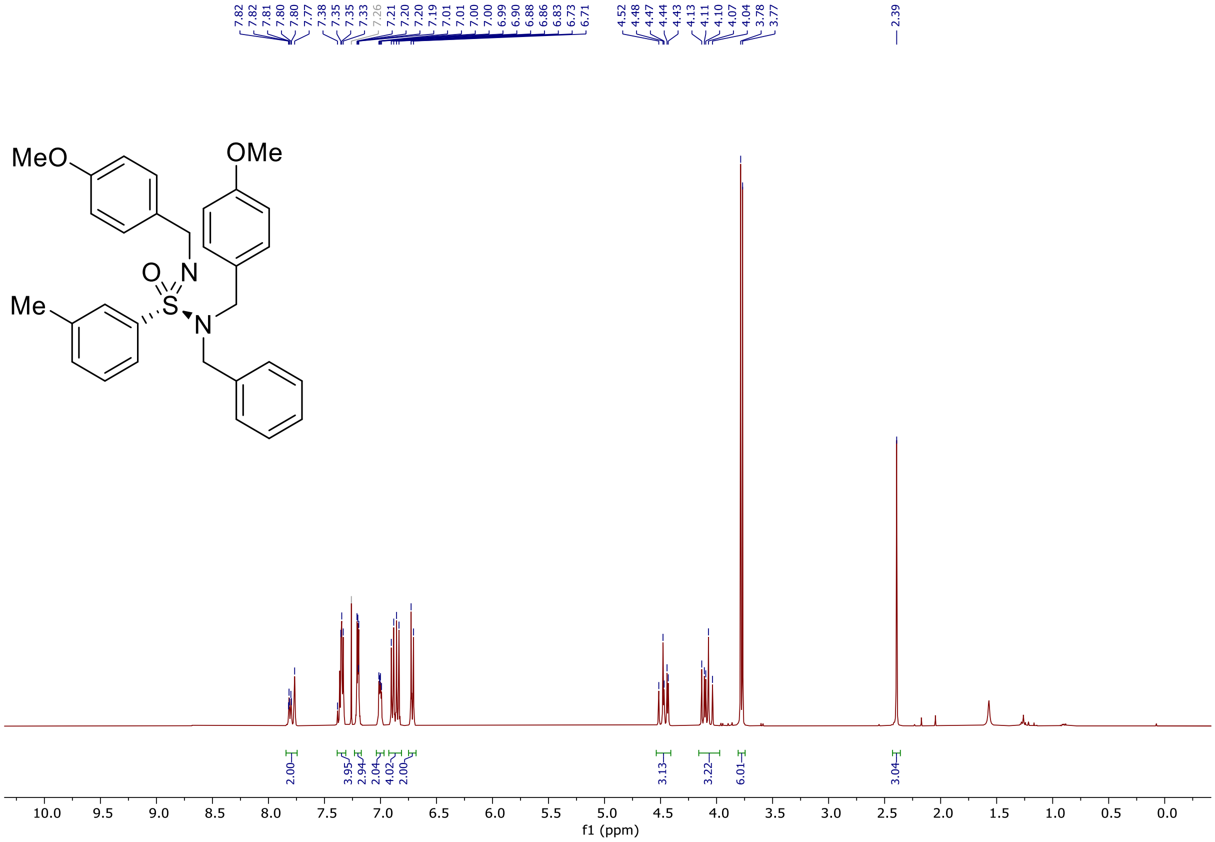

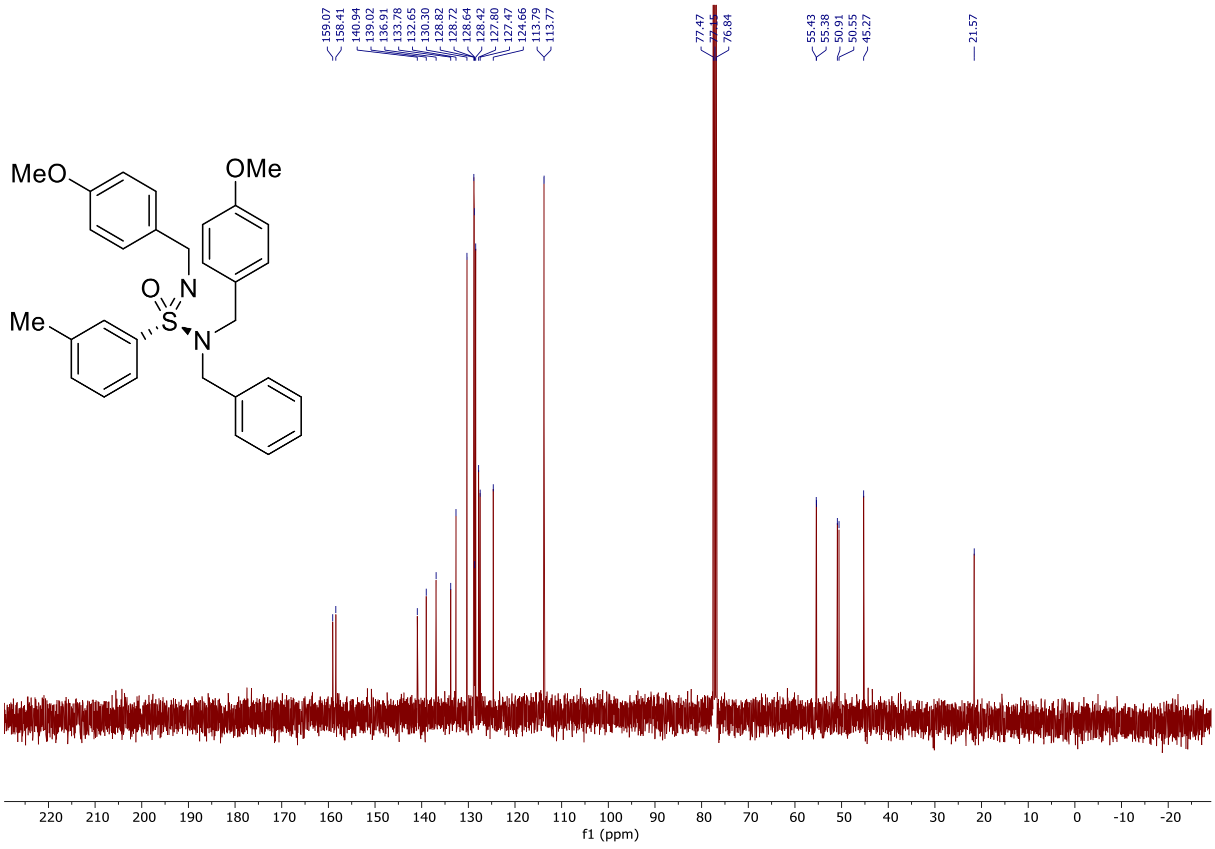

(*S*)-*N*-benzyl-*N*,*N'*-bis(4-methoxybenzyl)-2-methylbenzenesulfonimidamide (3e)

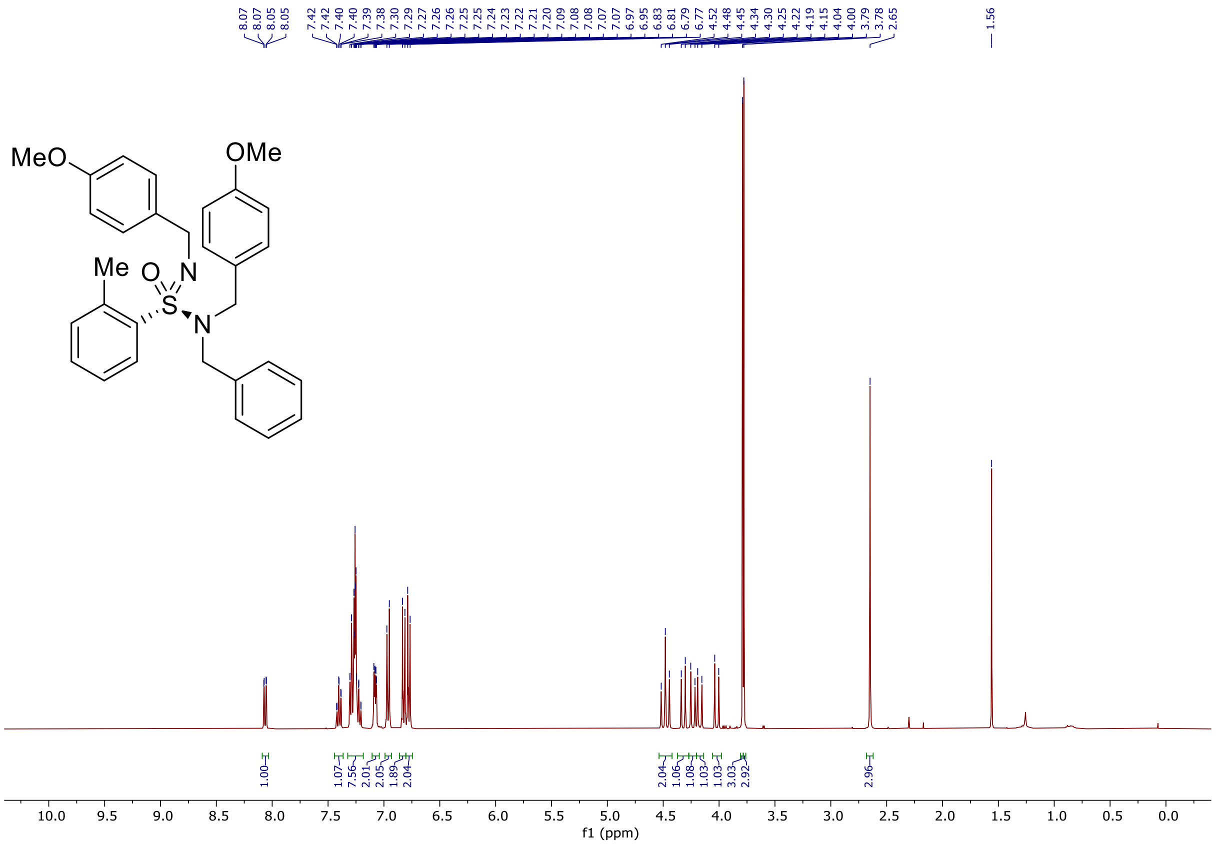

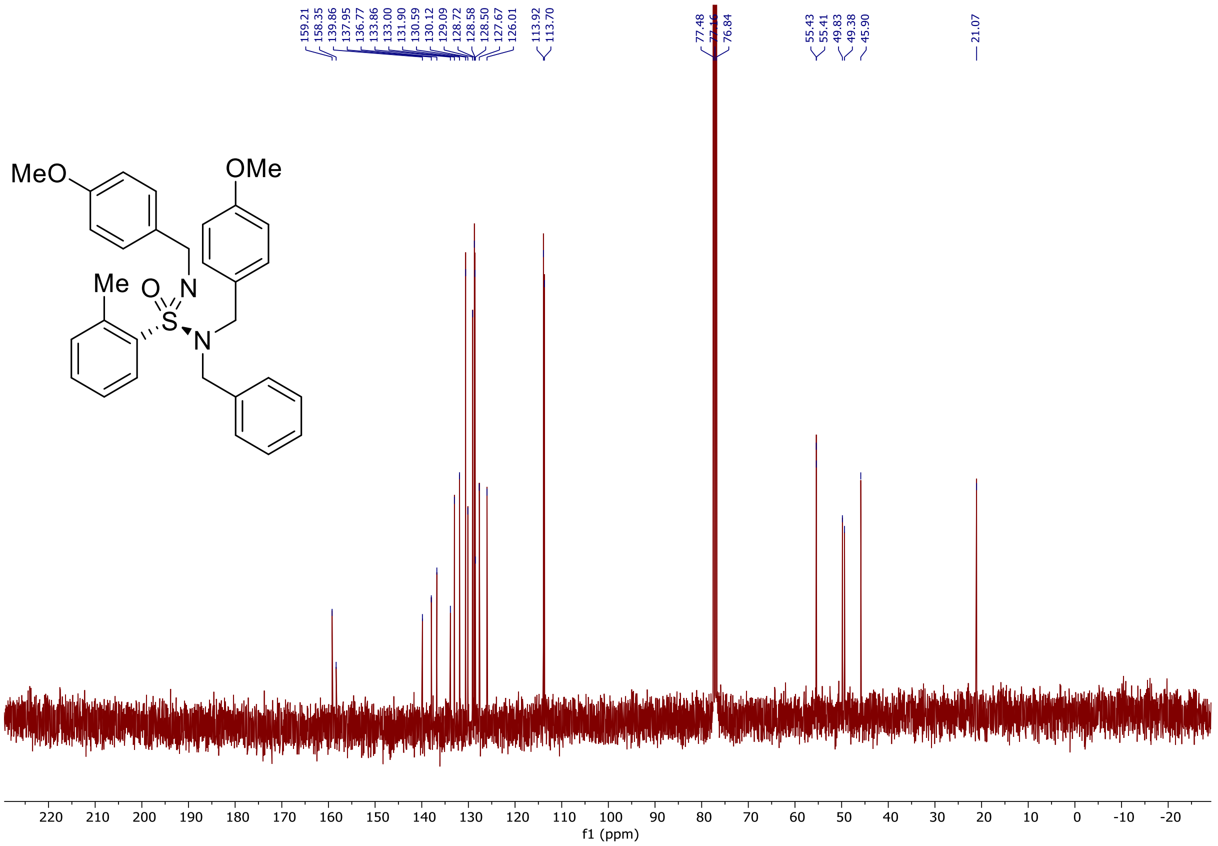

(*S*)-*N*-benzyl-4-fluoro-*N*,*N'*-bis(4-methoxybenzyl)benzenesulfonimidamide (3f)

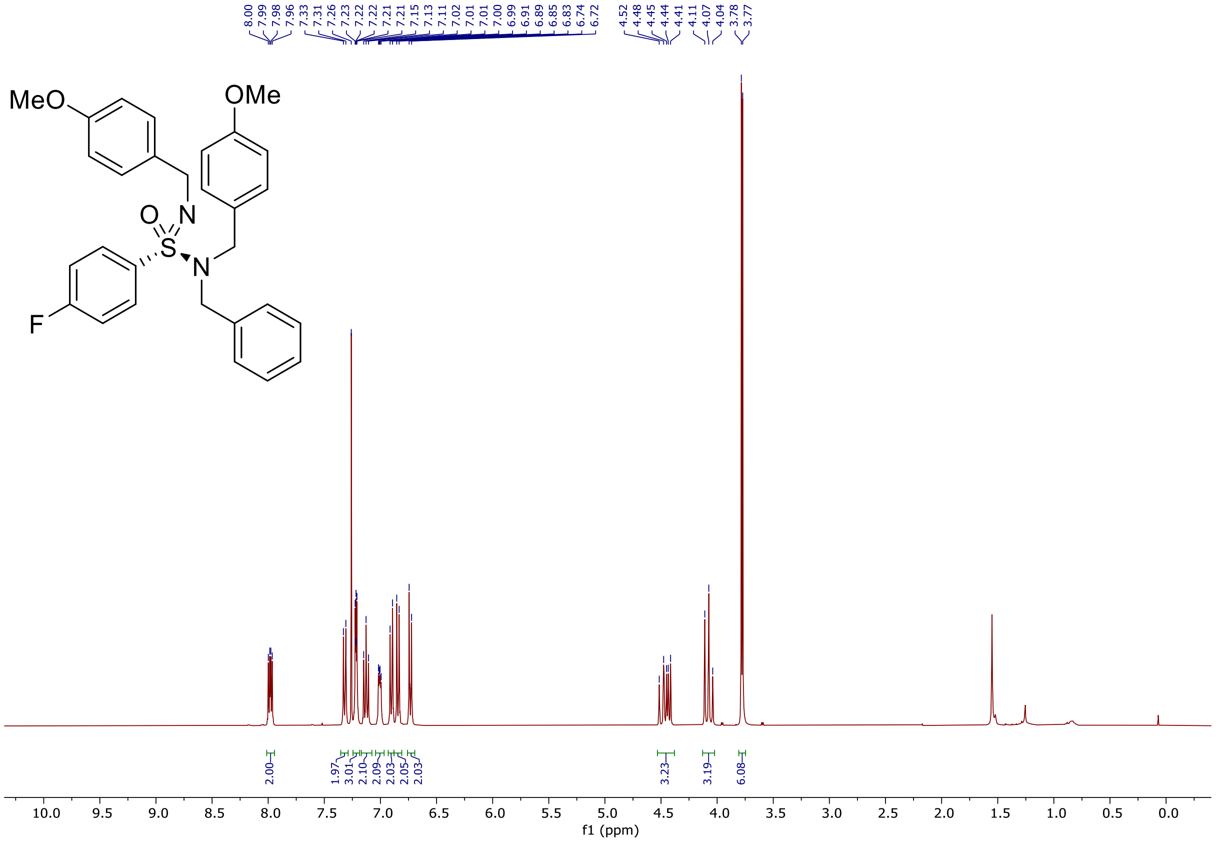

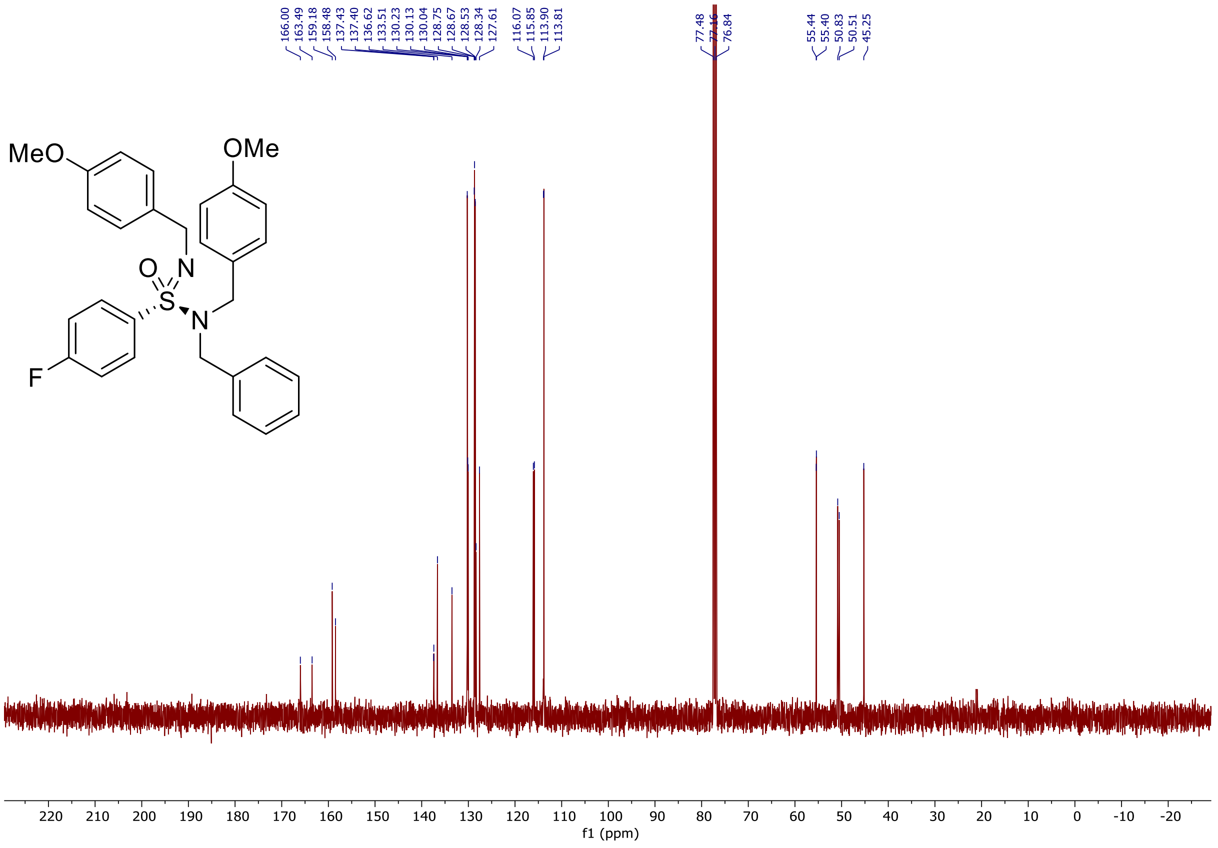

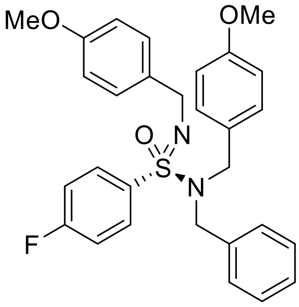

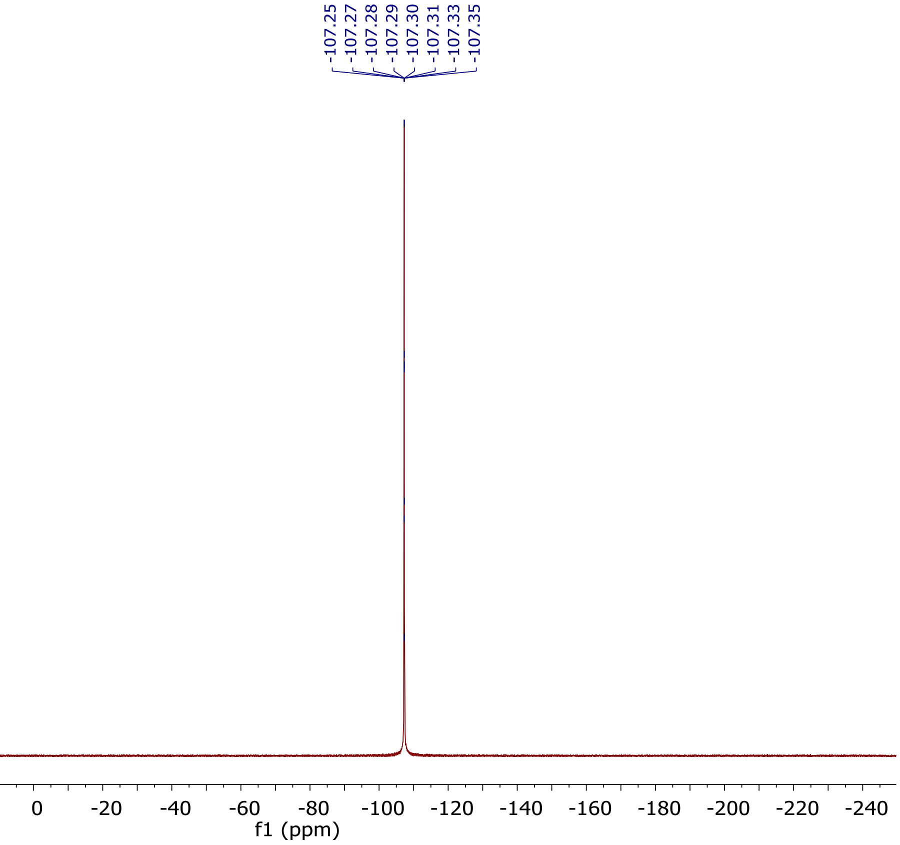

-107.25
-107.27
-107.28
-107.29
-107.30
-107.31
-107.33
-107.35
80
60
40
20
0
-20
-40
-60
-80
-100
-120
-140
-160
-180
-200
-220
-240
f1 (ppm)

(*S*)-*N*-benzyl-*N*,*N'*-bis(4-methoxybenzyl)thiophene-2-sulfonimidamide (3g)

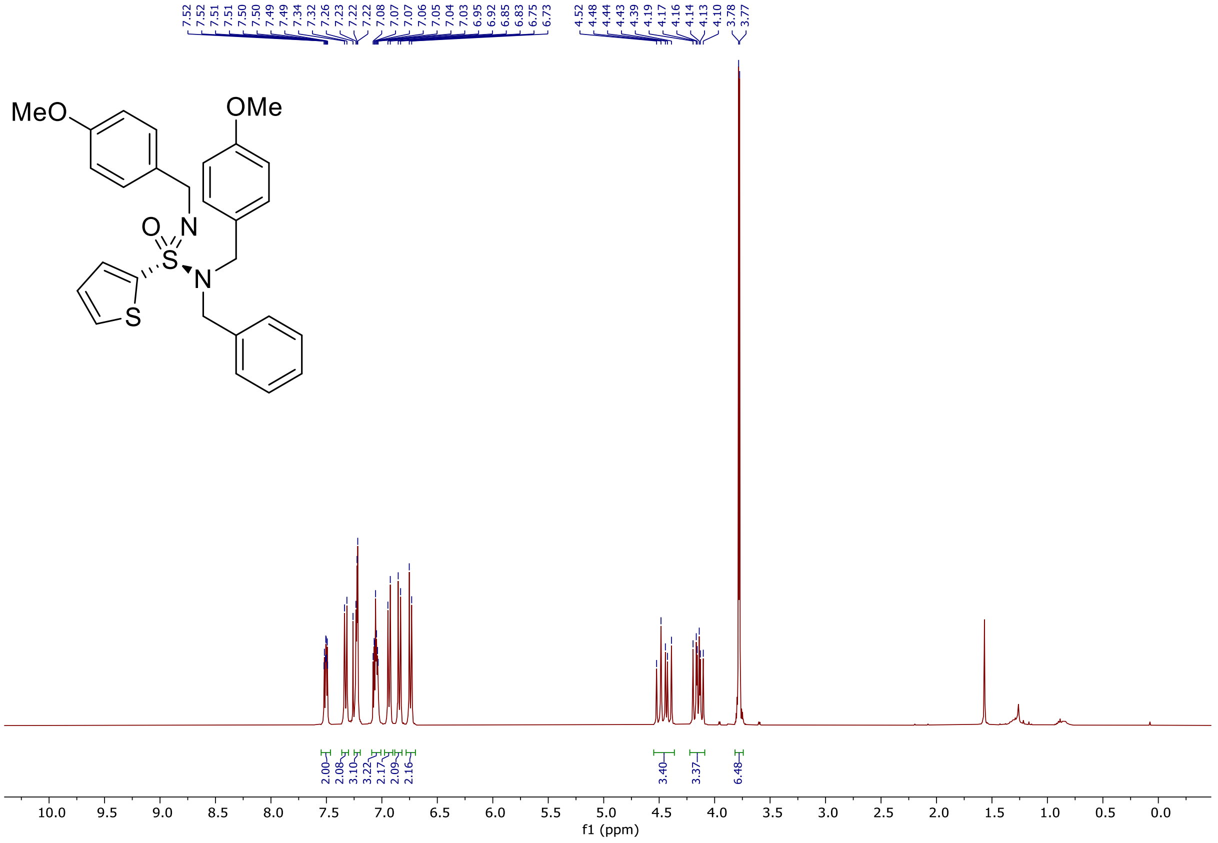

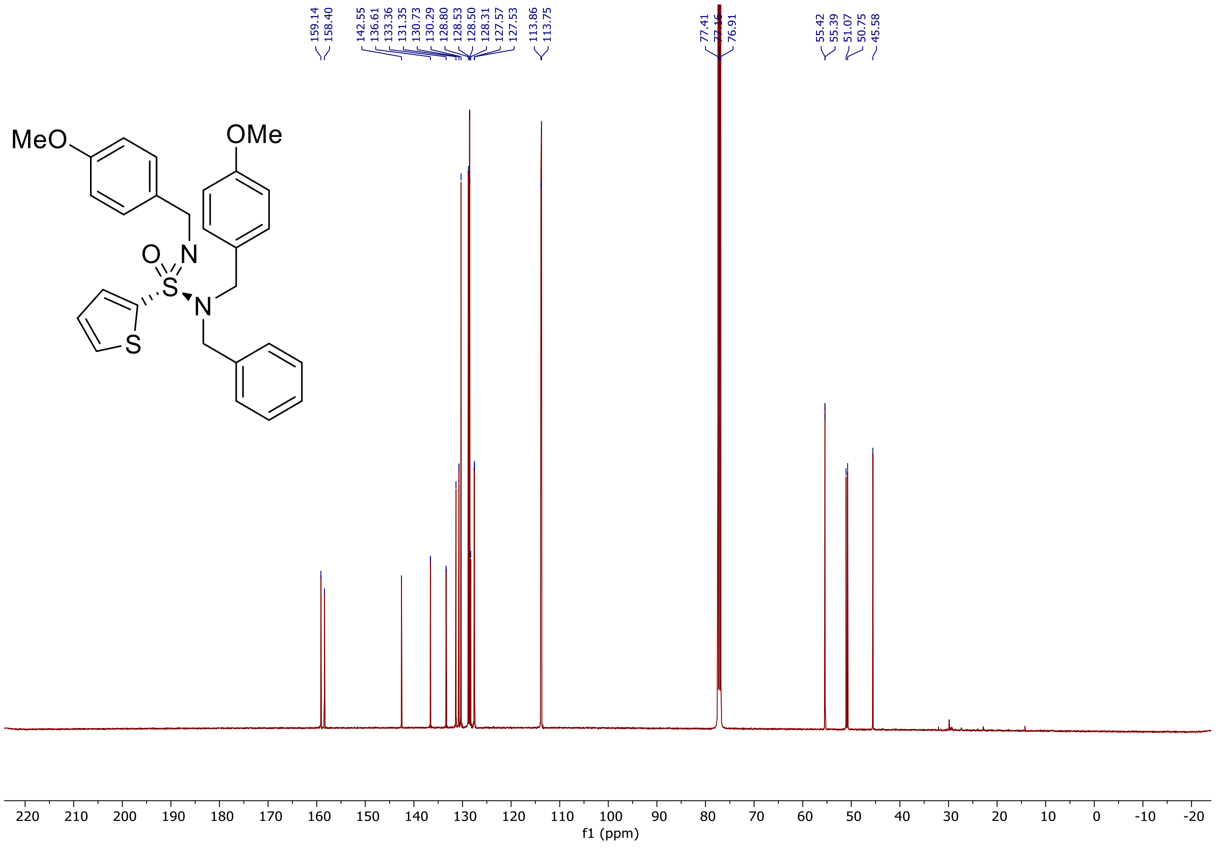

(*S*)-*N*-benzyl-*N*,*N'*-bis(4-methoxybenzyl)-4-(5-(p-tolyl)-3-(trifluoromethyl)-1H-pyrazol-1-yl)benzenesulfonimidamide (3h)

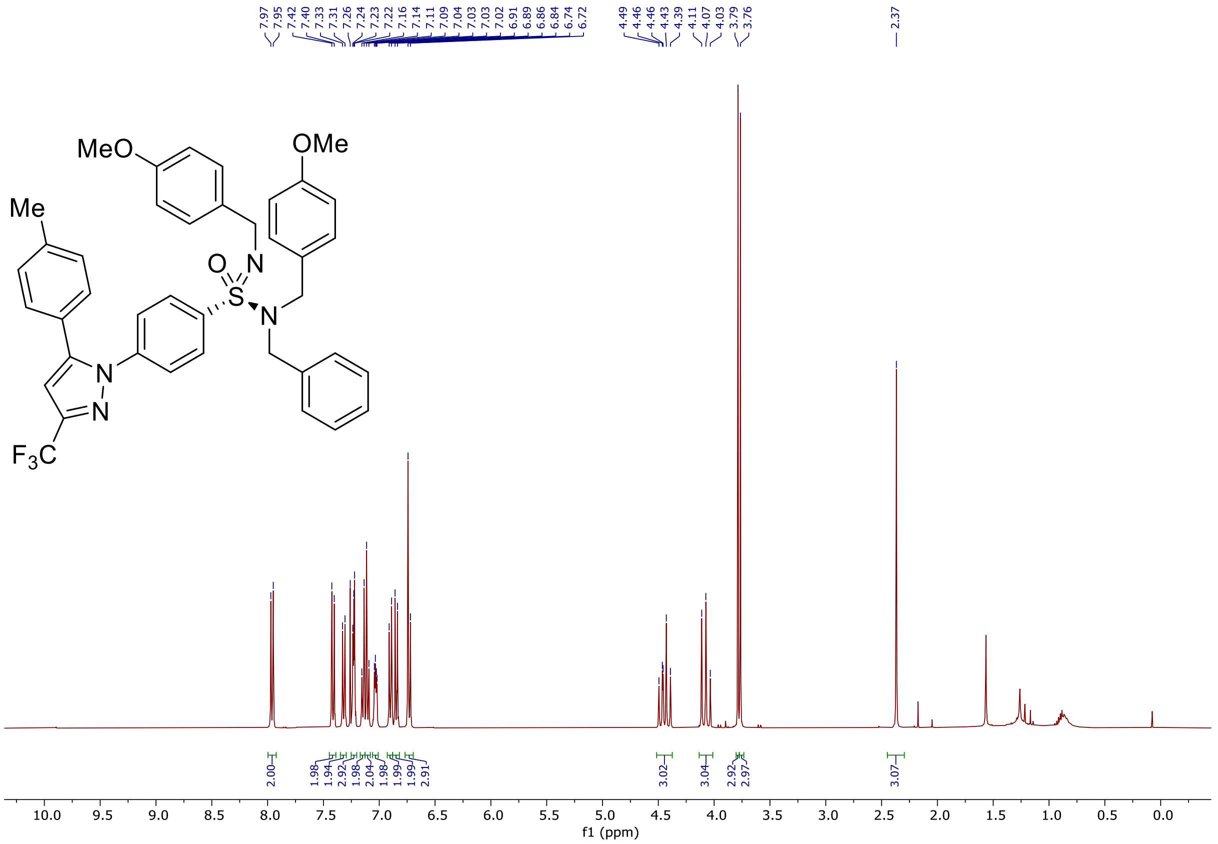

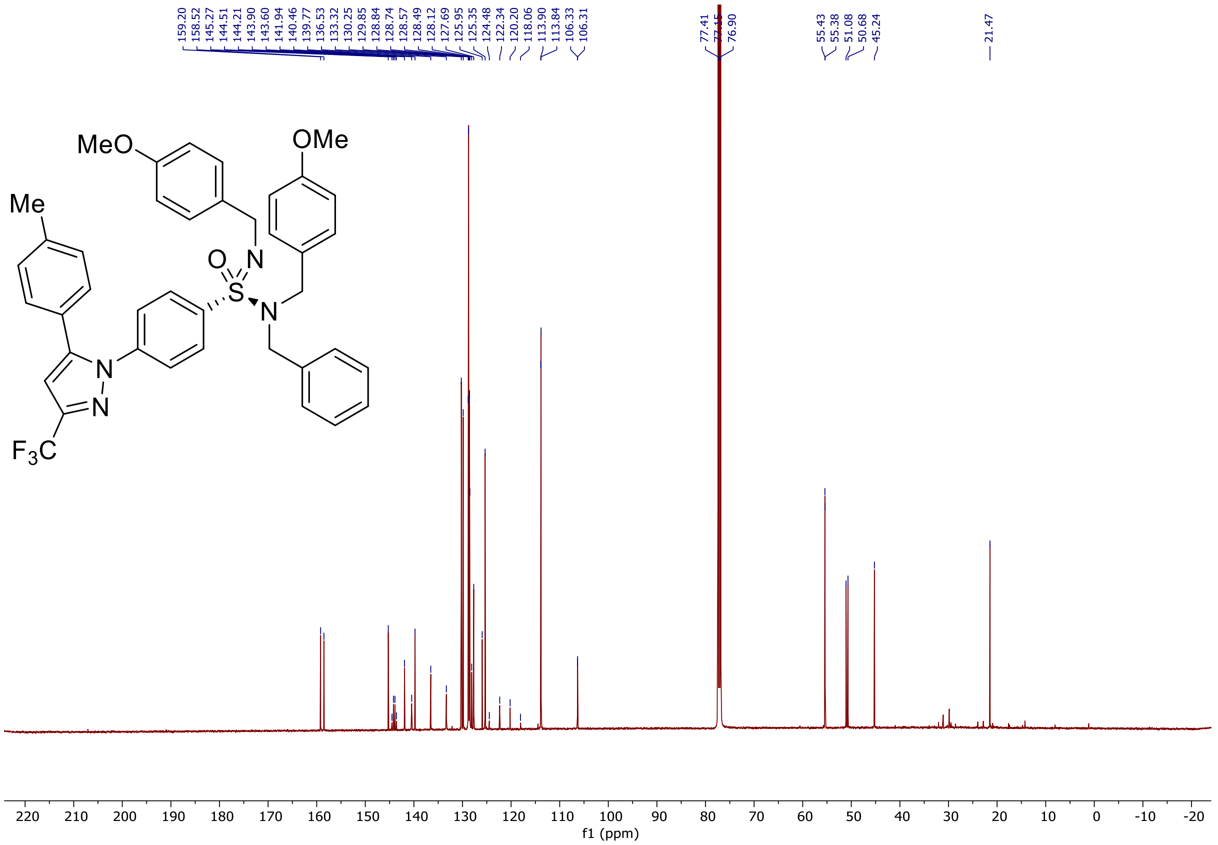

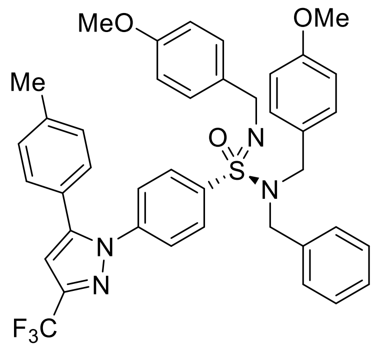
MeO
OMe
Me
O
N
S
N
N
N
F3C

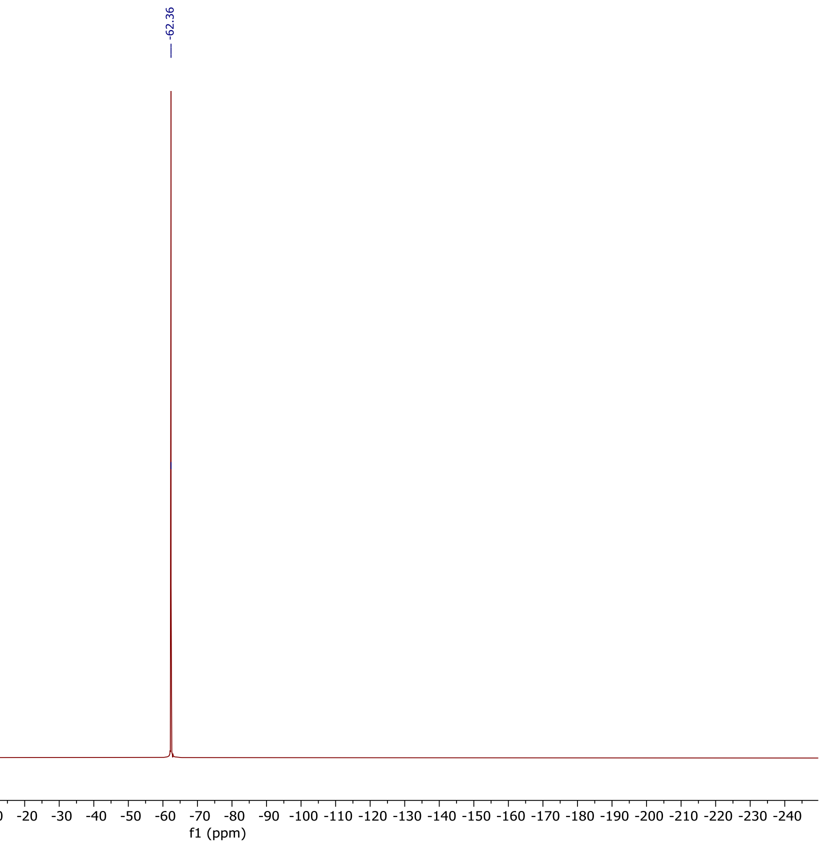
-62.36
90 80 70 60 50 40 30 20 10 0 -10 -20 -30 -40 -50 -60 -70 -80 -90 -100 -110 -120 -130 -140 -150 -160 -170 -180 -190 -200 -210 -220 -230 -240
f1 (ppm)

(*S*)-*N*-benzyl-*N*,*N'*-bis(4-methoxybenzyl)-2-methylprop-1-ene-1-sulfonimidamide (3i)

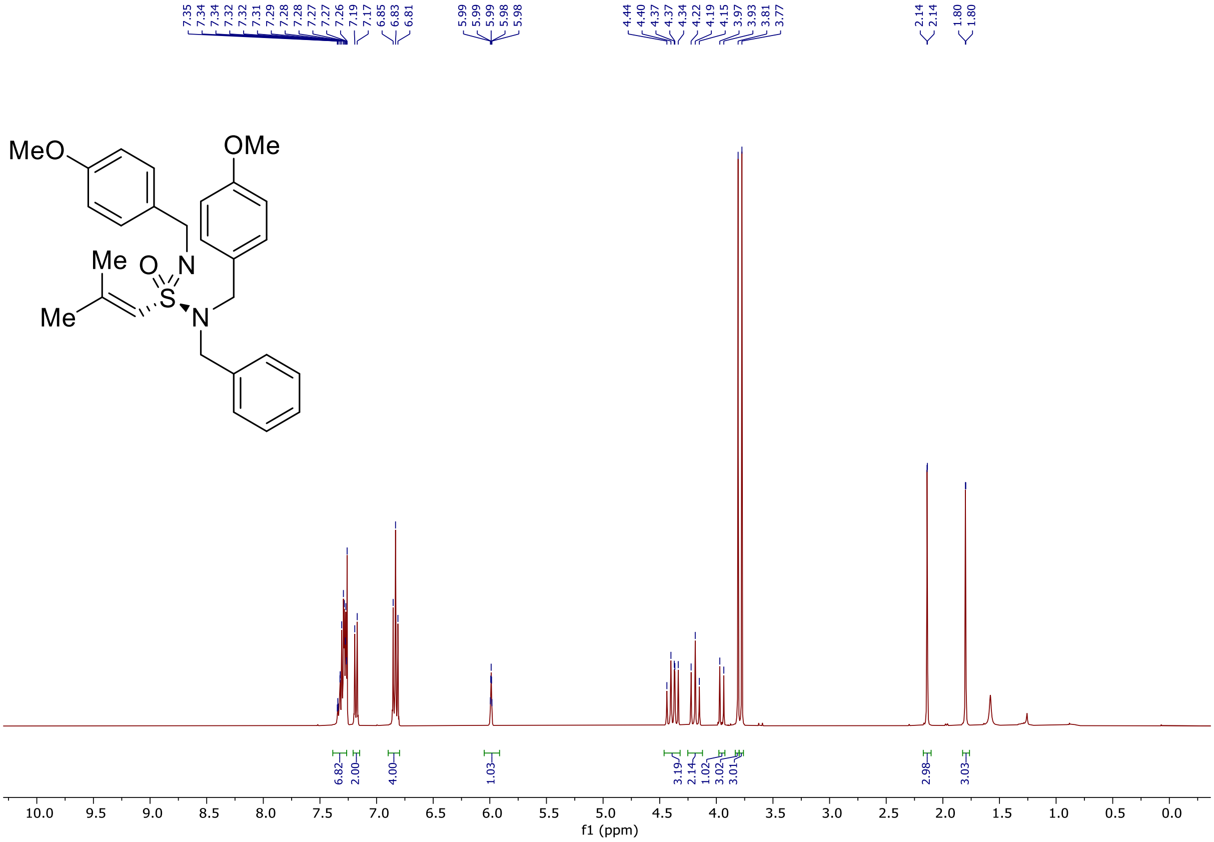

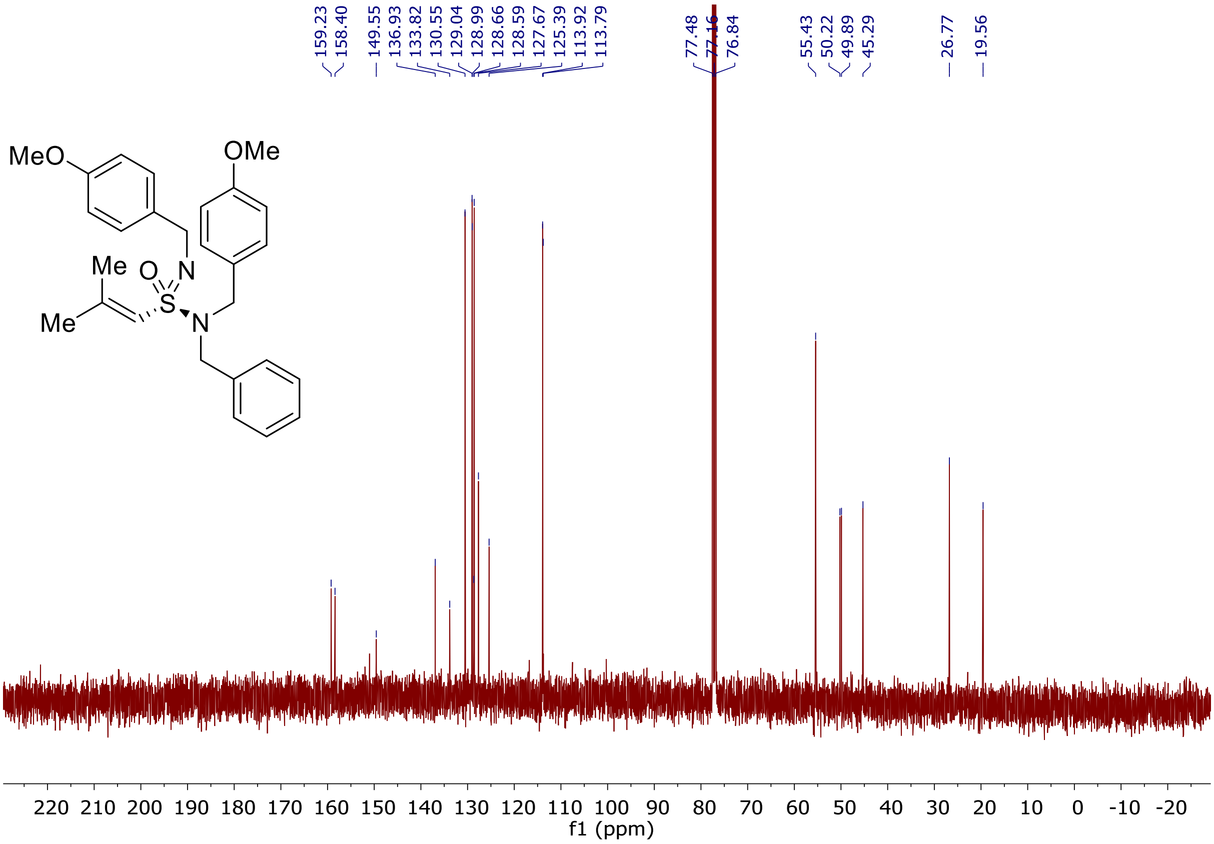

(*S*)-*N*-benzyl-*N*,*N'*-bis(4-methoxybenzyl)methanesulfonimidamide (3j)

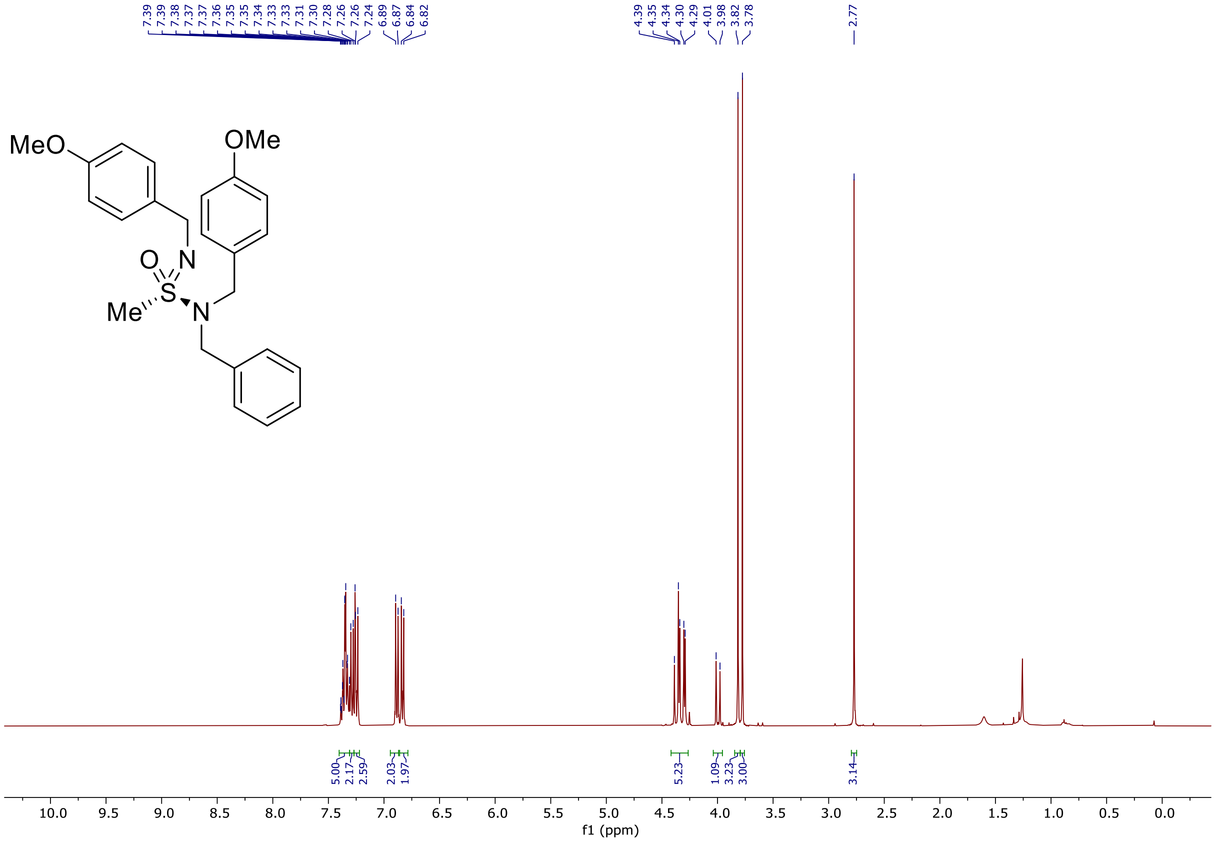

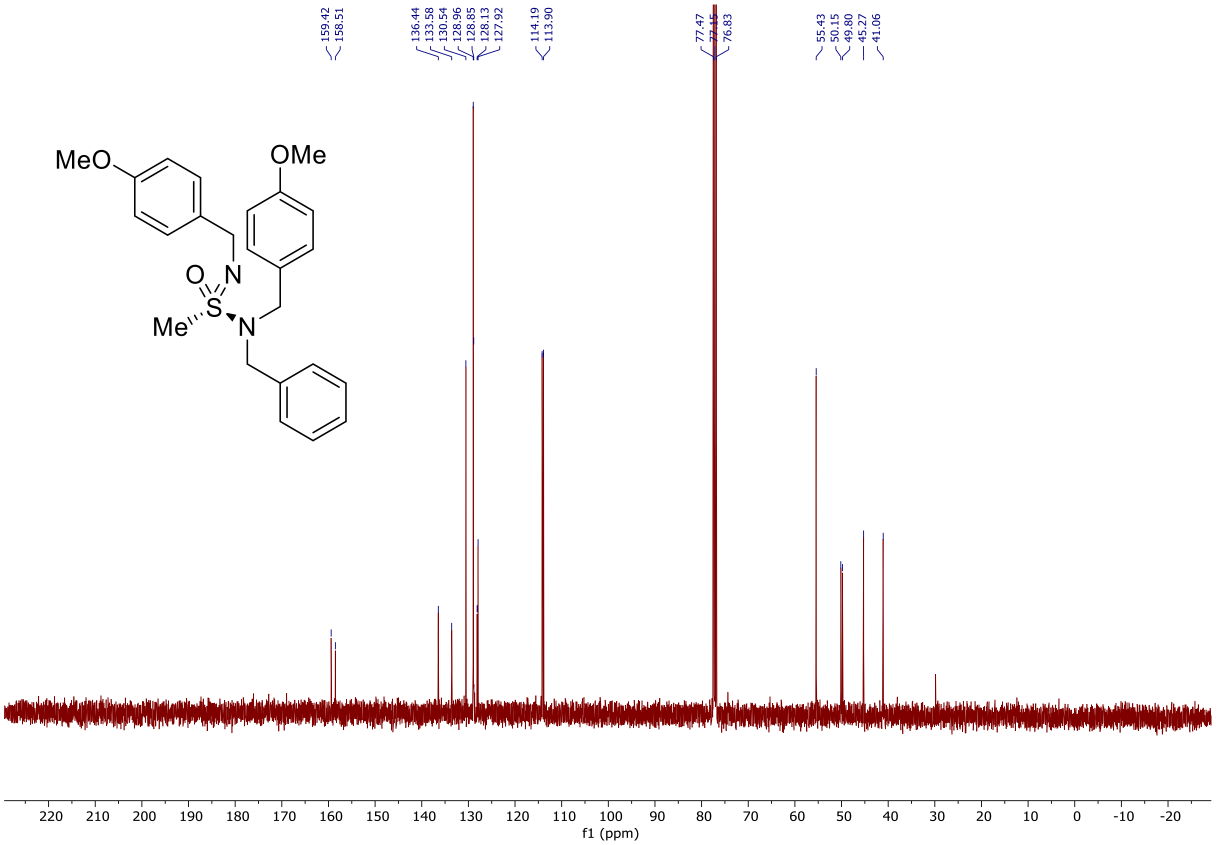

(*R*)-*N*-benzyl-*N*-(4-methoxybenzyl)-4-methylbenzenesulfonimidamide (5)

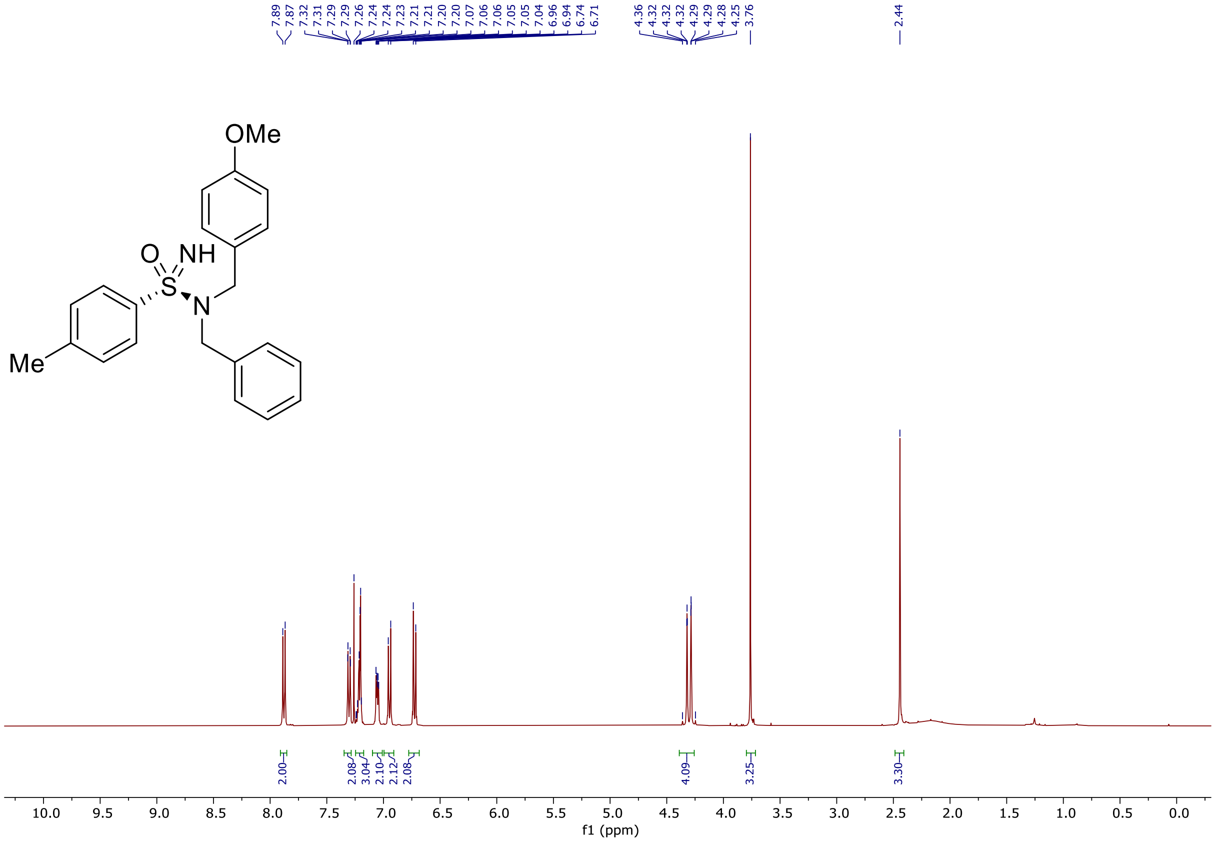

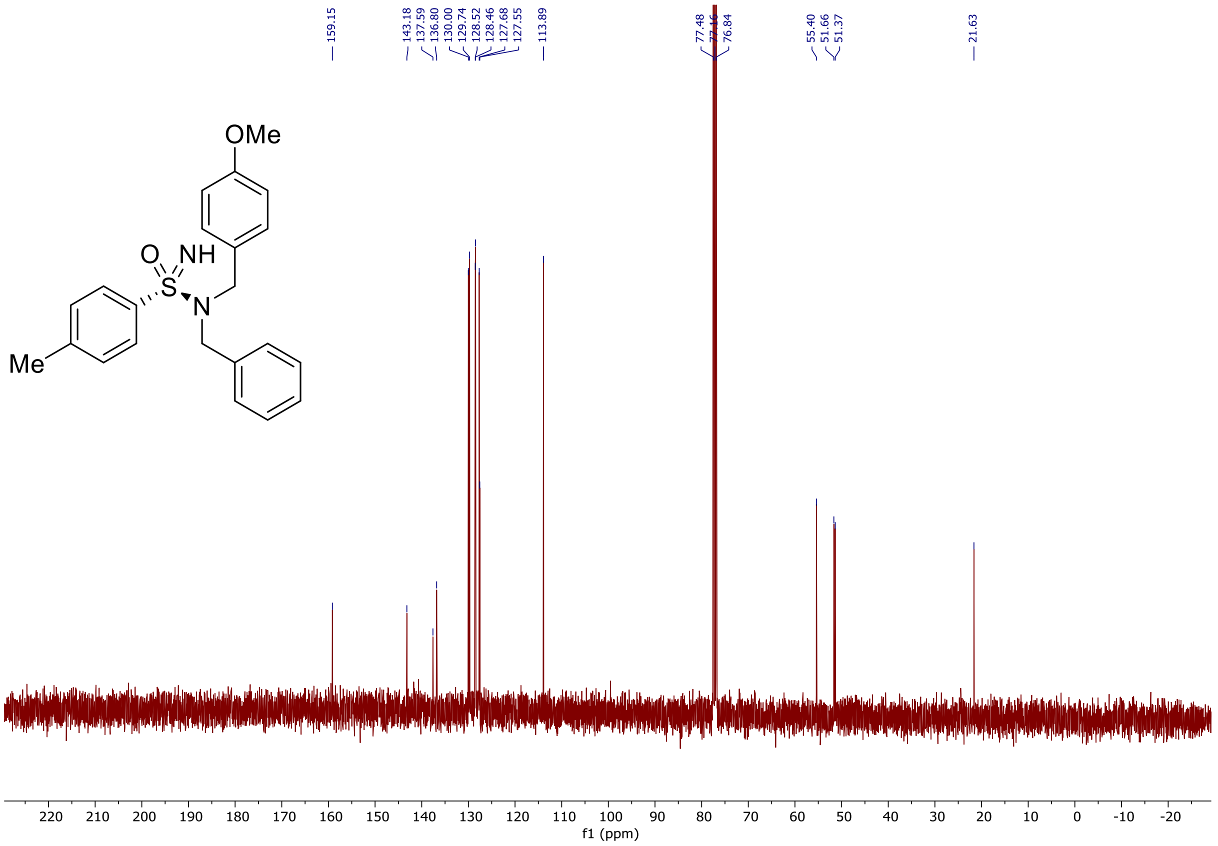

(*R*)-*N'*-benzyl-4-methylbenzenesulfonimidamide (6)

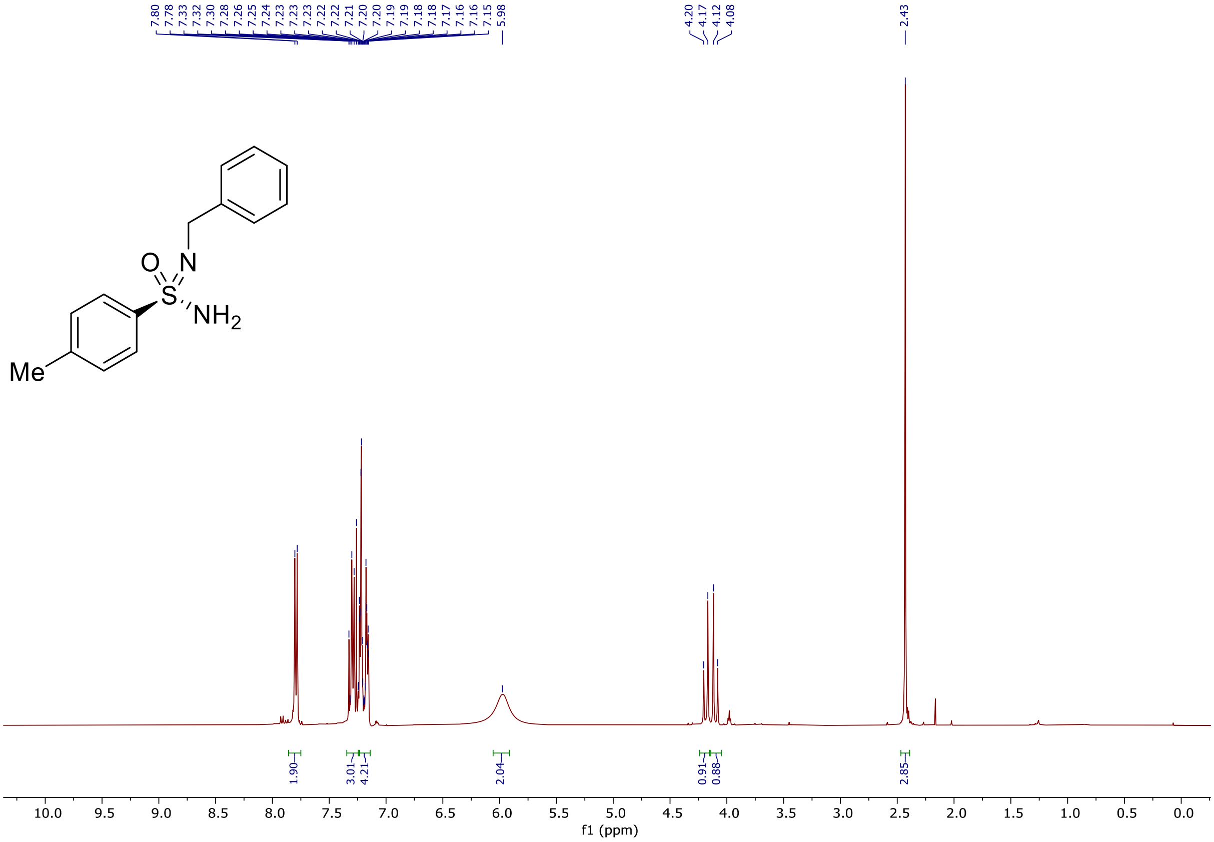

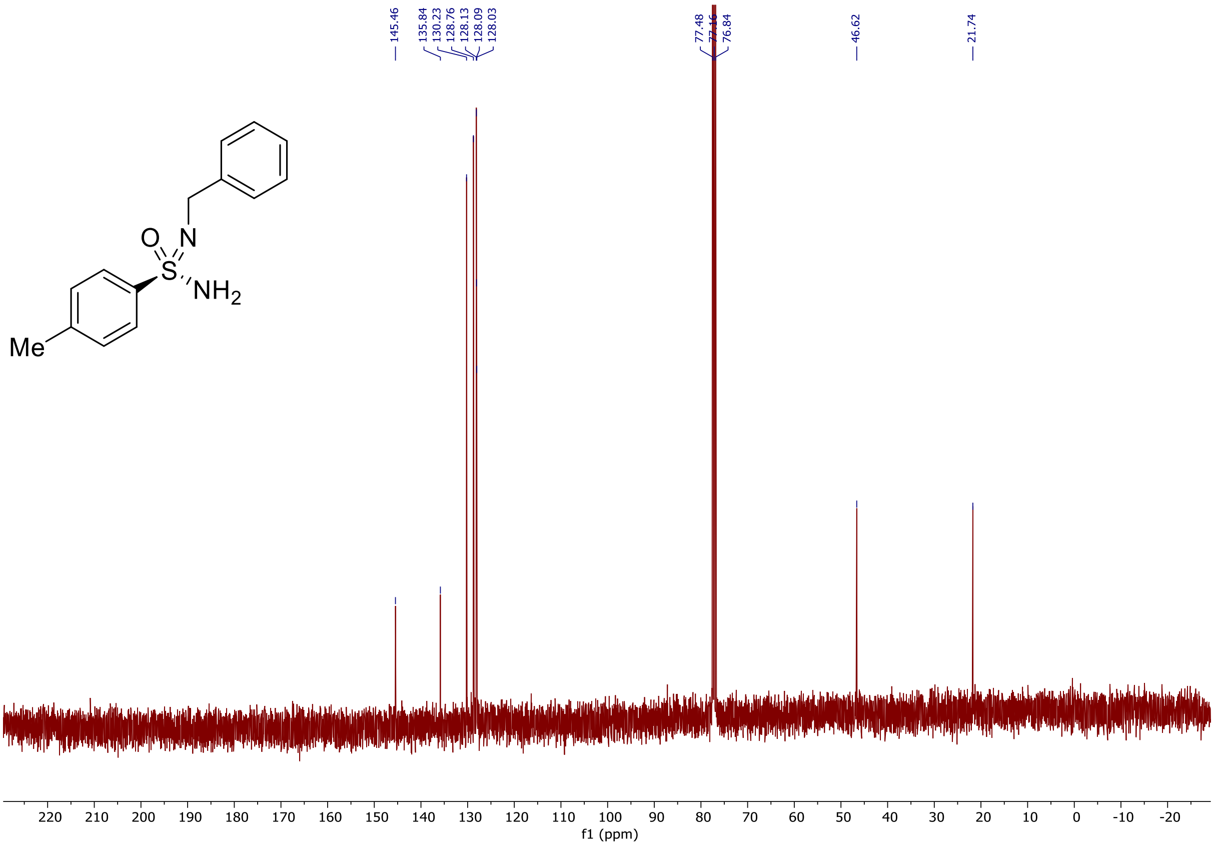

Supplement: Supplementary file 1 — Supporting Information [file ANIE-60-25680-s001.pdf]
